# Asymmetric [3 + 2] Cycloaddition to Access 3‐Pyrrolines and Their Switchable Transformations to Nine‐Membered Cyclic Sulfamidates and 2*H*‐Pyrroles

**DOI:** 10.1002/advs.202513904

**Published:** 2025-11-10

**Authors:** Seoung‐Mi Choi, Jong‐Un Park, Ju Hyun Kim

**Affiliations:** ^1^ Department of Chemistry Dongguk University Seoul 04620 South Korea; ^2^ Department of Chemistry (BK21 Four) Gyeongsang National University Jinju 52828 South Korea

**Keywords:** 3‐pyrroline, asymmetric cycloaddition, desulfonylation, ring expansion, trimethylenemethane

## Abstract

Herein, Pd‐catalyzed asymmetric [3 + 2] cycloaddition and olefin isomerization is reported to afford chiral 3‐pyrrolines using cyano‐TMM (trimethylenemethane) and cyclic sulfamidate imines. This represents a unique protocol to provide chiral *N*‐heterocycles bearing endocyclic olefins by asymmetric Pd‐TMM cycloaddition. The developed Pd‐catalyzed cycloaddition further extends to versatile synthetic transformations, offering a facile and unified approach to synthetically challenging yet valuable classes of heterocycles, including medium‐sized sulfamidate rings and 2*H*‐pyrroles, in a one‐pot operation. In particular, chemo‐switchable ring expansion and desulfonylation of sulfamidate‐fused 3‐pyrrolines are achieved by simple solvent and temperature changes under identical alkoxide base conditions.

## Graphical abstract

Pd‐catalyzed asymmetric [3 + 2] cycloaddition of cyano‐TMM with cyclic sulfamidate imines affords chiral 3‐pyrrolines bearing endocyclic olefins. Simple solvent and temperature adjustments under identical basic conditions enable chemo‐switchable one‐pot ring expansion or desulfonylation, providing a unified access to medium‐sized sulfamidates and 2*H*‐pyrroles while expanding the structural diversity and retrosynthetic utility of TMM chemistry.

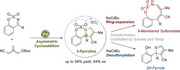

## 1 Introduction

**Figure 1:**
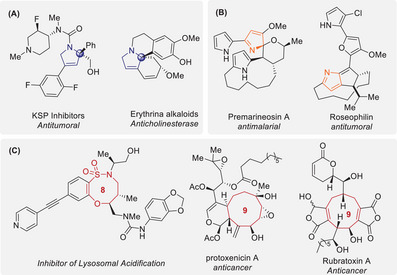
Selected Natural Products and Pharmaceuticals Containing 3‐Pyrrolines A), 2*H*‐Pyrroles B), and Medium‐Sized Rings C).

Pyrrolines are valuable *N*‐heterocyclic scaffolds widely present in naturally occurring alkaloids and pharmaceuticals.^[^ ^1^, ^2^, ^3^, ^4^ ^]^ They exist in three distinct isomeric forms, defined by the position of the double bond, and have unique biological activities. Chiral 3‐pyrrolines serve as key building blocks for kinesin spindle protein inhibitors, which are anticancer drug candidates for the treatment of prostate cancer, colorectal cancer, and other malignancies. These 3‐pyrrolines are found in natural Erythrina alkaloids, which act as anticholinesterase agents and have been investigated as potential treatments for Alzheimer's disease (**Figure** **1**A). In contrast to the well‐established enantioselective approaches used to produce other isomeric forms, only limited enantioselective synthetic methods have been developed for these compounds.^[^ ^5^, ^6^, ^7^, ^8^, ^9^, ^10^ ^]^ These include a Pd‐catalyzed asymmetric Heck reaction utilizing *N*‐protected 2‐pyrrolines^[^ ^11^ ^]^ and the chiral phosphine‐catalyzed [3 + 2] cycloaddition of *N*‐sulfonyl imines and allenoates.^[^ ^12^, ^13^, ^14^ ^]^

**Scheme 1:**
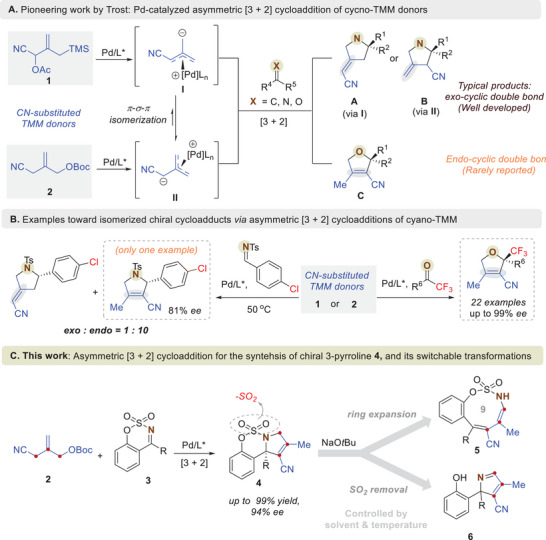
Research Background and Our Proposal.

Catalytic asymmetric cycloaddition is a highly efficient strategy for the rapid construction of complex ring systems and addressing key selectivity challenges, including chemo‐, regio‐, and stereoselectivity.^[^ ^15^, ^16^, ^17^, ^18^, ^19^, ^20^ ^]^ Since the pioneering works of Trost and Hayashi, palladium‐catalyzed asymmetric [3 + n] cycloadditions of trimethylenemethane (TMM) donors, which generate highly reactive π‐allylpalladium zwitterionic species and a broad range of dipolarophiles, have been well established.^[^ ^21^, ^22^, ^23^, ^24^, ^25^, ^26^, ^27^, ^28^, ^29^ ^]^ This strategy provides an efficient approach for synthesizing chiral carbo‐ and heterocycles bearing exo‐cyclic double bonds. However, the regioselectivity of chiral cycloadducts can present challenges when the synthetically versatile nitrile‐substituted TMM **1** is utilized. Mechanistically, TMM **1** transforms to highly nucleophilic *π*‐allylpalladium species **I**, which then undergoes *π*‐σ‐*π* isomerization to give the more stable *π*‐allylpalladium **II** (**Scheme** **1**A). In a report on the Pd‐catalyzed asymmetric [3 + 2] cycloaddition of *N*‐ sulfonyl imines and cyano‐TMM **1**, regioisomeric chiral pyrrolidines containing exocyclic double bonds (A and B in Scheme 1A) were obtained.^[^ ^30^ ^]^ The selective synthesis of these regioisomers can be achieved by tuning the rate of *π*‐σ‐*π* isomerization of the palladium complex using the electronic properties of imines and the steric effects of ligands. In 2018, the Trost group disclosed an advanced deprotonation strategy using cyano‐TMM **2**, which enhanced atom‐economy and regioselectivity due to the selective formation of stable π‐allylpalladium species **II**.^[^ ^31^ ^]^ While the catalytic asymmetric addition of cyano‐TMM donors **1** and **2** to dipolarophiles for the synthesis of methylene‐substituted heterocycles is well established, analogous transformations leading to heterocycles containing endocyclic olefins have rarely been reported (C in Scheme 1A). Screening of the asymmetric cycloadditions of cyano‐TMM **1** with *N*‐tosyl aldimines revealed only one example of chiral 3‐pyrroline formation when an electron‐deficient *p*‐chlorophenyl‐substituted aldimine was used under conditions favoring the thermodynamic product, albeit with low enantio‐ and regioselectivity (left, Scheme 1B). A general enantioselective [3 + 2] cycloaddition utilizing cyano‐TMM **2** and electron‐deficient fluorinated ketones has been reported for the synthesis of O‐heterocycles containing endocyclic double bonds. This method afforded chiral dihydrofurans with excellent enantioselectivities (right, Scheme 1B). Notably, the olefin isomerization‐driven formation of cycloadducts with endocyclic double bonds would greatly enhance the synthetic utility of Pd‐TMM cycloadditions and enable new retrosynthetic strategies that leverage TMM as a three‐atom synthon for the incorporation of a double bond into chiral ring systems.

Herein, we report this asymmetric [3 + 2] cycloaddition using cyano‐TMM **2** and cyclic sulfamidate imines **3** to produce chiral 3‐pyrrolines **4**, enabled by rapid olefin isomerization of initially formed pyrrolidines containing exo‐cyclic double bonds even at low temperatures (Scheme 1C). In the catalytic system, only 3‐pyrrolines **4** containing endo‐olefins were obtained with high efficiencies and enantioselectivities, regardless of the electronic properties of cyclic sulfamidate imines **3**. Moreover, tandem one‐pot approaches toward 9‐membered oxathiazonines **5** via chemoselective ring expansion and 2*H*‐pyrroles **6** via ring expansion, dearomative release of sulfur dioxide, and intramolecular Michael addition were achieved by adding NaO*t*Bu after Pd catalysis. Interestingly, these transformations were selectively controlled by changing the solvent and reaction temperature in the presence of the same base, which provided chemo‐switchable routes to important classes of heterocycles (Figure 1B,C). Notably, although sulfonamides represent one of the most common structural motifs in Food and Drug Administration (FDA)‐approved small‐molecule drugs, synthetic methods for medium‐sized (8–11‐membered) sulfonamides and sulfamidates remain largely underexplored.^[^ ^32^, ^33^, ^34^ ^]^

## 2 Results and Discussion

Initially, we focused on achieving asymmetric [3 + 2] cycloaddition by screening various chiral ligands in the presence of 2.5 mol% of Pd_2_(dba)_3_ catalyst at 30 °C in toluene using the cyclic sulfamidate ketimine **3a** and cyano‐TMM donor **2** as standard substrates (**Table** **1**). This revealed that only phosphoramidite ligands **L1**–**L5** were reactive, and that **L1**–**L2** with a 1,1′‐bi‐2‐naphthol back‐bone exhibited better reactivity than **L3**–**L5** with more electron‐rich backbones (Table 1, entries 1–7). 3‐Pyrroline **4a** was the exclusive product in this catalytic system, possibly due to regioselective formation of a 3‐methylene‐substituted *N*‐fused pyrrolidine followed by rapid double‐bond isomerization. When the phosphoramidite **L1** with a (*S*, *S*, *S*) motif was used, the *N*‐fused pyrrolidine **4a** was obtained with a best enantioselectivity of 78% ee and a yield of 67% (Table 1, entry 1). We have also examined the regioselectivity and stereoselectivity of this cycloaddition using cyano‐TMM donor **1** and the same catalytic system. This afforded **4a** with identical enantioselectivity, albeit with a slightly lower yield, possibly due to the formation of regioisomers (Table 1, entry 8). The reaction was also performed at −15 °C using the same catalytic system as entry 1 and a slightly greater amount of the cyano‐TMM donor **2** to enhance enantioselectivity. Both reactivity and enantioselectivity of the desired product **4a** were increased (96% yield and 87% ee) (Table 1, entry 9). Encouraged by this result, we investigated Pd catalysts further to increase stereoselectivity (Table 1, entries 10–13), and achieved a slightly better enantioselectivity of 90% ee using a CpPd(*η* ^3^‐C_3_H_5_) catalyst (Table 1, entry 13). Solvent screening at −15 °C with a CpPd(*η* ^3^‐C_3_H_5_) catalyst revealed that Methyl tert‐Butyl Ether (MTBE) enhanced the enantioselectivity to 92% ee, albeit with reduced reactivity (Table 1, entries 14–15); notably, performing the reaction at 0 °C preserved the high enantioselectivity while improving the yield to 88% (Table 1, entry 16).

**Table 1:** Reaction Optimization of Asymmetric [3 + 2] Cycloaddition.^a)^

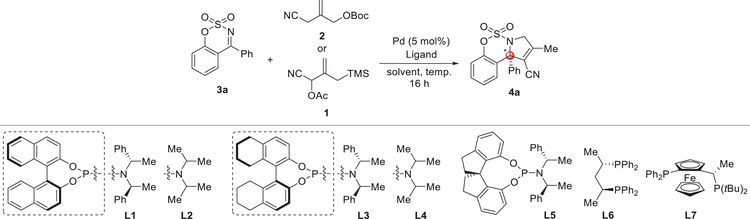						
entry	Pd	Ligand	Solvent	T [°C]	Yield [%]^b)^	ee [%]^c)^
1	Pd_2_(dba)_3_	**L1**	toluene	30	67	78
2	Pd_2_(dba)_3_	**L2**	toluene	30	31	46
3	Pd_2_(dba)_3_	**L3**	toluene	30	80	77
4	Pd_2_(dba)_3_	**L4**	toluene	30	35	35
5	Pd_2_(dba)_3_	**L5**	toluene	30	29	55
6	Pd_2_(dba)_3_	**L6**	toluene	30	N.R	−
7	Pd_2_(dba)_3_	**L7**	toluene	30	N.R	−
8^d)^	Pd_2_(dba)_3_	**L1**	toluene	30	56	78
9^e)^	Pd_2_(dba)_3_	**L1**	toluene	−15	96	87
10	[Pd(*η* ^3^‐C_3_H_5_)Cl]_2_	**L1**	toluene	−15	N.R	−
11	[Pd(π‐cinnamyl)Cl]_2_	**L1**	toluene	−15	N.R	−
12	CpPd(π‐cinnamyl)	**L1**	toluene	−15	43	78
**13** ^e)^	**CpPd(*η* ^3^‐C_3_H_5_)**	**L1**	**toluene**	**−15**	**95**	**90**
14	CpPd(*η* ^3^‐C_3_H_5_)	**L1**	DCM	−15	38	58
15	CpPd(*η* ^3^‐C_3_H_5_)	**L1**	MTBE	−15	70	92
16	CpPd(*η* ^3^‐C_3_H_5_)	**L1**	MTBE	0	88	92

^a)^ Reaction conditions: **3a** (0.1 mmol), **2** (0.1 mmol), Pd (5 mol%) based on Pd, ligand **L1**–**L5** (11 mol%) or **L6**–**L7** (5.5 mol%) in 1 mL of solvent for 16 h under Ar;

^b)^ Isolated yield;

^c)^ Determined by HPLC using a chiral stationary phase;

^d)^ **1** was used as cyano‐TMM donor instead of **2**;

^e)^ The reaction was performed with 1.5 equiv of **2**.

**Scheme 2:**
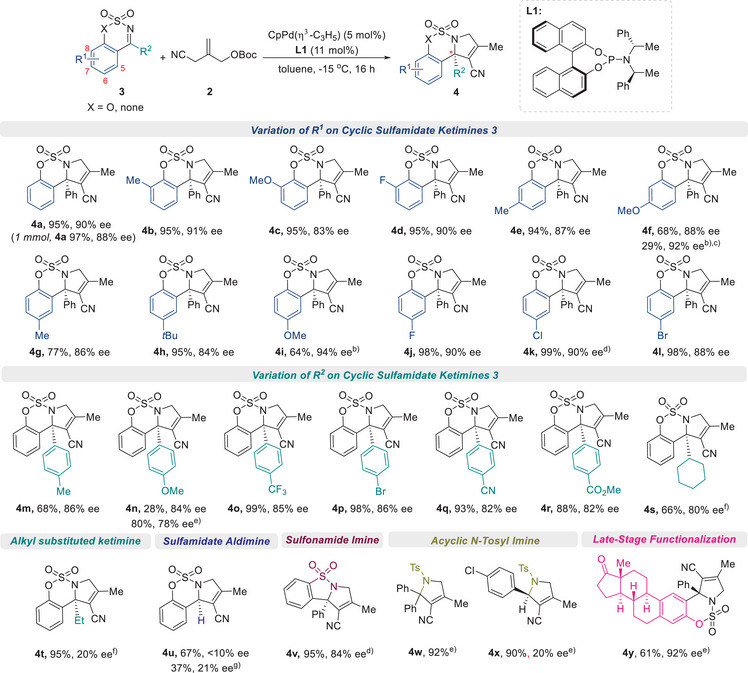
Substrate Scope for Catalytic Asymmetric [3 + 2] Cycloadditions; ^a)^Reaction conditions: **1** (0.2 mmol), **2a** (0.3 mmol), CpPd(*η* ^3^‐C_3_H_5_) (5 mol%), **L1** (11 mol%) in toluene (2 mL), ‐15 °C, 16 h under Ar; ^b)^The reaction was conducted in MTBE at r.t.; ^c)^ **4f** showed solubility‐dependent reactivity (68% yield in toluene vs lower yield with slightly higher ee in MTBE); ^d)^The reaction was conducted in MTBE at 0 °C; ^e)^The reaction was conducted at 30 °C; ^f)^The reaction was conducted at 60 °C; ^g)^Ming‐Phos ligand (**L8**) (5.5 mol%) was used; **L8**: (*R*)‐*N*‐((*S*)‐(2‐(diphenylphosphino)phenyl)(phenyl)methyl)‐*N*,2‐dimethylpropane‐2‐sulfinamide.

**Figure 2:**
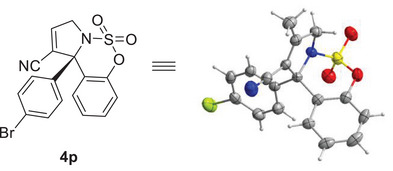
Determination of the Absolute Configuration of **4p**.

Having optimized the reaction conditions, we explored the scope of the catalytic asymmetric [3 + 2] cycloaddition/double bond isomerization cascade to construct *N*‐fused 3‐pyrrolines **4** (**Scheme** **2**). We were pleased to observe that ketimines with relatively low inherent reactivity were effectively engaged in this asymmetric TMM cycloaddition. All attempted reactions proceeded smoothly to generate sulfamidate‐fused 3‐pyrrolines **4** bearing a quaternary carbon center without forming pyrrolidines bearing exocyclic double bonds. Substitution at the 8‐position of the arene with electron‐donating groups (–Me, –OMe) or a fluoro group provided the corresponding 3‐pyrrolines **4b–4d** in high yields with excellent enantioselectivities 83%–91% ee. Similarly, substitution at the 7‐position with –Me or –OMe afforded products **4e** and **4f**, respectively, with high enantioselectivities (up to 92% ee). An investigation of 6‐substituted ketimines revealed good tolerance for both electron‐donating (–Me, –*t*Bu, –OMe) and halogen groups (–F, –Cl, –Br), with yields and enantioselectivities **4g–4l** of up to 98% and 94% ee. Notably, halogenated ketimines (**3j–3l**) were more reactive than their electron‐rich counterparts (**3g–3i**). A broad range of aryl moieties at the R^2^ position was then examined. The presence of electron‐withdrawing groups (–CF_3_, –Br, –CN, –CO_2_Me) on phenyl ketimines significantly improved yields of corresponding products **4o**–**4r** (up to 99%), while *p*‐methyl‐ and *p*‐methoxy‐substituted phenyl ketimines yielded **4m** and **4n** in lower yields and comparable levels of enantioselectivity. The low yield of compound **4n** was attributed to its limited solubility, and performing the reaction at 30 °C improved the yield at the expense of enantioselectivity. X‐ray crystallography of **4p** confirmed an *S* configuration (**Figure** **2**). This catalytic TMM cycloaddition is also applied to aryl cyclohexyl sulfamidate imine, which efficiently afforded **4s** in 66% yield and 80% ee. When the substrate bearing an ‐Et substituent at the R^2^ position on the cyclic sulfamidate imine **3** was employed, the desired product **4t** was obtained in 95 % yield but with low enantioselectivity (20 % ee). In addition, the reaction also proceeded smoothly for the corresponding aldimine substrate, selectively providing the product **4u** with low enantioselectivity. To improve the enantioselectivity, we also examined a sulfinamide phosphine ligand **L8** (Ming‐Phos), which has been reported to exhibit remarkable performance in Pd‐catalyzed asymmetric reactions,^[^ ^35^ ^]^ under otherwise identical conditions; a slightly improved enantioselectivity (21 % ee) was observed, albeit with significantly reduced yield. The low enantioselectivities observed for **4t** and **4u** are likely due to the smaller steric demand of the R^2^ substituents (–Et or –H) on the cyclic sulfamidate imine **3**, leading to reduced facial discrimination during the stereo‐determining step.^[^ ^36^, ^37^, ^38^ ^]^ This asymmetric Pd catalysis was also effectively utilized to synthesize sulfonamide‐fused 3‐pyrroline **4v**. The substrate scope was further extended to acyclic *N*‐tosyl imines. Reaction of *N*‐tosyl benzophenone imine afforded the corresponding product **4w** in 92% yield. In the case of *N*‐tosyl *p*‐chlorobenzaldimine, the cycloadduct **4x** was obtained in 90% yield with modest enantioselectivity (20% ee). Interestingly, under the optimized conditions, the reaction predominantly produced the cycloadduct bearing an exo‐cyclic methylene, which gradually underwent double‐bond isomerization to the endo‐olefin as the reaction time and temperature increased (see ESI for details). Late‐stage transformation was attempted to determine the utility of the developed methodology for synthetic applications. When the estrone‐derived imine was used as the substrate, the desired cycloadduct **4y**, which featured a 3‐pyrroline‐fused polycyclic ring, was efficiently synthesized in 61% yield with 92% ee.

The synthesized *N*‐fused 3‐pyrrolines **4** possessed a synthetically versatile sulfamidate functionality. To demonstrate the synthetic utility of the development methodology, we explored transformations further using these cycloadducts. In our previous study, the desulfonylation of *N*‐sulfamidate‐fused cycloadducts proceeded via a KO*t*Bu‐promoted E1cB mechanism to produce oxazoline.^[^ ^39^ ^]^ Accordingly, we envisioned that the base‐promoted desulfonylation of *N*‐sulfamidate‐fused 3‐pyrroline **4a** could provide access to 2*H*‐pyrrole **6a**, a challenging target due to its intrinsically unstable, non‐aromatic nature (**Table** **2**).

**Table 2:** Optimization of Ring Expansion and Desulfonylation Reactions of 3‐Pyrroline.^a)^

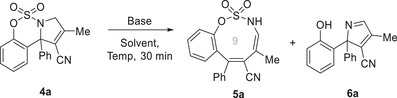					
Entry	Base [equiv.]	Solvent	Temp [°C]	5a yield [%]^b)^	6a yield [%]^b)^
1	KO*t*Bu (2)	toluene	30	−	‐
2	KO*t*Bu (2)	THF	30	31	‐
3	KO*t*Bu (2)	1,4‐dioxane	30	12	−
4	KO*t*Bu (2)	DMF	30	37	<5
5	KO*t*Bu (2)	DMSO	30	50	<5
6	KO*t*Bu (4)	THF	30	55	−
**7**	**NaO*t*Bu (4)**	**THF**	**30**	**81**	−
8	NaO*t*Am (4)	THF	30	75	−
9	NaO*t*Bu (2)	DMSO	80		26
**10**	**NaO*t*Bu (2)**	**DMF**	**80**	−	**73**
11	NaO*t*Bu (2)	1,4‐dioxane	80	−	24
12	NaO*t*Bu (2)	MeCN	80	−	44

^a)^ Reaction conditions: **3a** (0.1 mmol) and base (2 or 4 equiv.) in solvent (1 mL) for 30 min under Ar;

^b)^ Isolated yield.

To evaluate the feasibility of this transformation, we examined various solvents in the presence of excess KO*t*Bu at 30 °C using 3‐pyrroline **4a**. Interestingly, instead of the anticipated desulfonylation, a ring‐expansion occurred in certain solvents such as Tetrahydrofuran (THF), 1,4‐dioxane, Dimethylformamide (DMF), and Dimethyl sulfoxide (DMSO), leading to the formation of nine‐membered oxathiazonine **5a** (Table 2, entries 1–5). Notably, despite the broad spectrum of biological activities exhibited by medium‐sized sulfamidates and sulfonamides, synthetic preparatory methods are limited. Although a few ring‐expansion reactions with metal catalysts or under acidic conditions have been developed,^[^ ^32^, ^33^, ^34^, ^40^, ^41^, ^42^, ^43^, ^44^ ^]^ no examples of ring expansion under metal‐free conditions have been reported. The use of DMSO best facilitated the transformation to the medium‐sized ring **5a**; however, 2*H*‐pyrrole **6a** was also obtained in low yield (Table 2, entry 5). Reaction conditions in THF were further optimized to enhance the yield of oxathiazonine **5a**. Increasing the amount of KO*t*Bu to 4 equivalents improved the yield to 55% (Table 2, entry 6), and replacing KO*t*Bu with NaO*t*Bu further increased the yield to 81% and resulted in the optimal condition (Table 2, entry 7). Notably, desulfonylation leading to the formation of 2*H*‐pyrrole **6a** was observed when NaO*t*Bu (2 equiv.) was used in polar solvents at an elevated temperature (80 °C) (Table 2, entries 9–12); the optimal yield of 73% was achieved in DMF (Table 2, entry 10). These results demonstrate that transformations involving ring expansion and desulfonylation might be selectively controlled by optimizing solvent and temperature, and enable the reaction to be switched to produce the nine‐membered sulfamidate **5a** or 2*H*‐pyrrole **6a**.

**Scheme 3:**
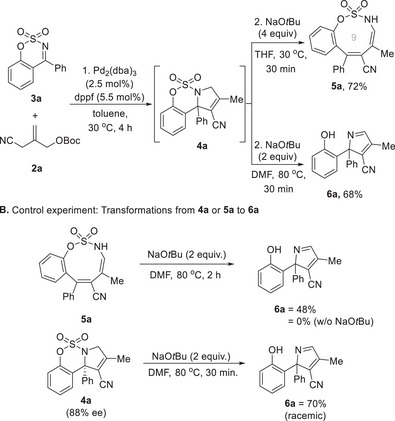
Tandem One‐Pot Approaches to Synthesize Oxathiazonine **5a** and 2*H*‐Pyrrole **6a** and the Control Experiment.

Next, we explored the possibility of a challenging tandem one‐pot approach based on integrating Pd‐catalyzed [3 + 2] cycloaddition of cyano‐TMM **2a** and **3a**, double‐bond isomerization, and base‐promoted ring expansion or desulfonylation to achieve the selective synthesis of heterocycles **5a** or **6a** (**Scheme** **3**A). When optimized reaction conditions were applied in a tandem manner, oxathiazonine **5a** and 2*H*‐pyrrole **6a** were selectively obtained in 72% and 68% yields, respectively, demonstrating effective disconnection and formation of multiple bonds in a one‐pot process. Control experiments were conducted to gain mechanistic insight (Scheme 3B). Based on the observation that two pathways could be selectively controlled by varying the reaction temperature under identical basic conditions, we hypothesized that NaO*t*Bu‐mediated ring expansion preferentially occurs at lower temperatures and is followed by desulfonylation. To test this hypothesis, the isolated 9‐membered ring **5a** was subjected to basic conditions in DMF to afford 2*H*‐pyrrole **6a** in 48% yield. To elucidate the reaction pathway of this one‐pot transformation, temperature‐ and time‐dependent NMR studies were conducted in DMF‐d_7_. Upon addition of two equivalents of NaO*t*Bu, intermediate **4a** first converts to **5a** at room temperature, which subsequently transforms into **6a** at elevated temperature (see ESI for details). When 3‐pyrroline **4a** bearing a stereogenic center with 88% ee was subjected to desulfonylation conditions, it was anticipated that the resulting 2*H*‐pyrrole **6a** would retain its enantiomeric purity if the reaction proceeded via an E1cB mechanism. However, a racemic mixture was obtained. These results support a stepwise mechanism in which ring expansion occurs first, followed by NaO*t*Bu‐promoted desulfonylation, leading to the formation of 2*H*‐pyrrole **6a** via a ring‐reconstruction pathway.

**Scheme 4:**
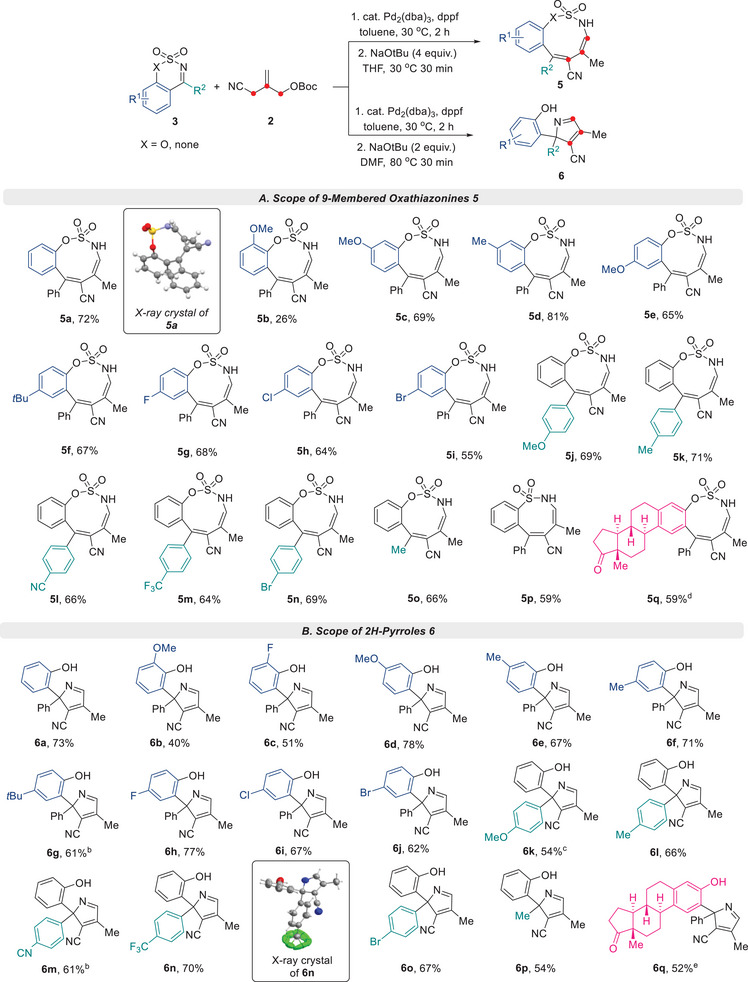
Substrate Scope for Divergent Synthesis of Nine‐Membered Sulfamidates **5** and 2*H*‐Pyrroles **6;** a)Reaction conditions: 1. **1** (0.2 mmol), **2a** (0.3 mmol), Pd_2_(dba)_3_ (2.5 mol%), dppf (11 mol%) in toluene (2 mL) for 2 h at 30 °C under Ar; **A**: 2. NaO*t*Bu (4 equiv.) in THF (2 mL) for 30 min at 30 °C; **B**: 2. NaO*t*Bu (2 equiv.) in DMF (2 mL) for 30 min at 80 °C; ^b)^NaO*t*Bu (2.5 equiv.); ^c)^NaO*t*Bu (4 equiv.); ^d)^Reactions were conducted at 40 °C; ^e)^1. Conducted at 40 °C, 2. Conducted at 90 °C.

We also examined the scope of tandem one‐pot reactions for the construction of oxathiazonines **5** (**Scheme** **4**A). Cyclic sulfamidate imines bearing –OMe at the 8‐position of the arene gave the corresponding product **5b**, but reactivity was low. On the other hand, sulfamidate imines bearing –OMe or –Me at the 8‐position of the phenyl afforded **5c** and **5d**, respectively, at yields up to 81%. Various substituents (–OMe, –*^t^* Bu, –F, –Cl, –Br) at the 6‐position of the arene of cyclic imines were also well tolerated, and provided the oxathazonines **5e**–**5i** in moderate yields. Subsequently, we investigated sulfamidate cyclic imines with various substituents at the R^2^ position. Cyclic imines bearing electron‐donating (–Me, –OMe) and electron‐withdrawing (–CN, –CF_3_, –Br) groups at the para‐position of the phenyl ring successfully produced the required products **5j**–**5n** in good yields. Furthermore, when a methyl‐substituted ketimine was used as the substrate, the corresponding oxathiazonine **5o** bearing an alkyl substituent at the R^2^ position was obtained. Notably, the transformation was successfully extended to a cyclic sulfonamide imine substrate, which led to the efficient synthesis of the eight‐membered sulfamide **5p**, thereby demonstrating the potential of this cycloaddition/ring expansion strategy for the construction of medium‐sized rings with diverse ring sizes. Late‐stage transformation using the one‐pot approach was also performed using estrone‐derived imine, and the desired polycyclic nine‐membered oxathiazonine **5q** was isolated in 59% yield.

The ability of the tandem one‐pot process to synthesize 2*H*‐pyrroles **6** using the same substrates was also investigated (Scheme 4B). Cyclic sulfamidate imines bearing various substituents with different phenyl positions were employed. The corresponding 2*H*‐pyrroles **6b**–**6j** were selectively obtained in moderate to good yields. Similarly, cyclic sulfamidate imines with different arene substituents and a methyl substituent at the R^2^ position were used. The process proceeded without any significant changes in chemoselectivity or reactivity, affording a series of 2*H*‐pyrrole products **6k**–**6p**. In addition, an estrone‐substituted 2*H*‐pyrrole **6q** was obtained in 52% yield from an estrone‐derived sulfamidate imine substrate.

**Scheme 5:**
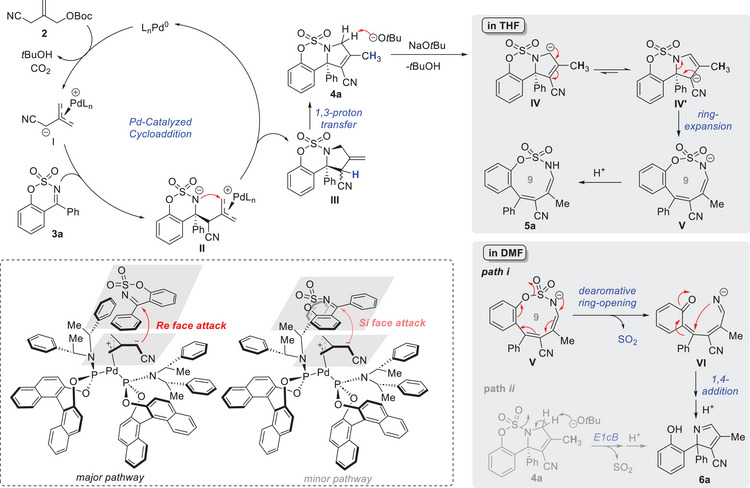
Proposed Mechanism.

Based on the results of our mechanistic study and previous reports on Pd‐catalyzed TMM‐cycloaddition,^[^ ^21^, ^22^, ^23^, ^24^, ^25^, ^26^, ^27^, ^28^, ^29^, ^30^, ^31^ ^]^ we propose the reaction mechanism shown in **Scheme** **5**. Briefly, the proposed catalytic cycle commences with the formation of a *π*‐allylpalladium zwitterionic species **I** via oxidative addition of Pd(0) species and deprotonation by *tert*‐butoxide. Enantioselective addition of the Pd‐TMM donor **I** to sulfamidate imine **3a** then generates the zwitterionic intermediate **II**, with the nucleophilic attack preferentially occurring from the *Re* face of Pd‐TMM **I** (Scheme 5). Subsequent intramolecular nucleophilic attack of the nitrogen in complex **II** on the cationic *π*‐allylpalladium species produces intermediate **III**, which undergoes a 1,3‐proton shift to yield endocyclic olefin **4a**. In the presence of *tert*‐butoxide, deprotonation at the carbon adjacent to the nitrogen generates intermediate **IV**, and in THF at room temperature, **IV** undergoes a carbanion rearrangement, favoring the formation of its more stabilized resonance structure **IV'**. This rearrangement is followed by a ring‐expansion, facilitated by double‐bond formation and C─N bond cleavage, to afford the nine‐membered product **V**. Final protonation leads to the formation of oxathiazonine **5a**.

Conversely, at elevated temperatures in DMF, the reaction proceeds via a ring expansion and desulfonylation cascade (Path *i*). Sulfur dioxide extrusion from intermediate **V**, driven by dearomative ring‐opening reaction, furnishes an *ortho*‐quinone methide intermediate **VI**. Subsequent intramolecular Michael‐type addition and protonation furnish the final 2*H*‐pyrrole product **6a**. Control experiments ruled out the possibility of a base‐promoted E1cB elimination pathway starting from 3‐pyrroline **4a** and involving sulfur dioxide extrusion and subsequent protonation (Path *ii*).

## 3 Conclusion

In summary, we developed a highly efficient method for the synthesis of chiral *N*‐fused 3‐pyrrolines via Pd‐catalyzed asymmetric [3 + 2] cycloaddition and olefin isomerization using cyano‐TMM donors and cyclic sulfamidate imines. Under Pd catalytic conditions, the asymmetric cycloaddition underwent unusually rapid olefin isomerization to provide endocyclic olefin products exclusively in yields of up to 99% and enantioselectivities of up to 94% ee. The formation of endocyclic cycloadducts significantly expands the retrosynthetic utility of Pd–TMM cycloadditions, offering a powerful strategy for constructing chiral ring systems bearing internal double bonds through the strategic incorporation of TMM as a three‐carbon synthon. Furthermore, the developed method enables switchable access to distinct heterocyclic scaffolds, including nine‐membered sulfamidates via ring expansion and 2*H*‐pyrroles via ring expansion/dearomatization‐driven sulfur dioxide release/intramolecular Michael addition cascade by one‐pot syntheses. The selective transformation pathways were precisely controlled by changing solvents and reaction temperature in the presence of NaO*t*Bu, which highlighted the versatility of this catalytic approach and the synthetic utility of sulfamidate‐fused 3‐pyrrolines. This tunable and selective synthesis of three valuable *N*‐heterocyclic scaffolds from a common set of substrates offers a promising strategy for the rapid construction of molecular libraries with potential applications in medicinal and materials science.

## 4 Experimental Section

### General Procedure for the Synthesis of Compounds **4**

Under an argon atmosphere, CpPd(allyl) (0.01 mmol, 5 mol%) and ligand **L1** (0.022 mmol, 11 mol%) were dissolved in toluene or MTBE (2.0 mL) and stirred at room temperature for ≈15 min. The resulting solution was then cooled to −15 or 0 °C and stirred for an additional 5 min. Subsequently, *N*‐sulfonyl ketimine **3** (0.2 mmol, 1.0 equiv.) and cyano‐TMM donor **2** (0.3 mmol, 1.5 equiv.) were added sequentially under an argon atmosphere. The reaction mixture was stirred at −15 or 0 °C for 16 h. Upon completion (monitored by TLC), the solvent was removed under reduced pressure, and the crude residue was purified by silica gel column chromatography to afford compound **4**. The ee value was determined by HPLC using a Daicel chiral column.

### General Procedure for the Synthesis of Oxathiazonine **5**

Under an argon atmosphere, Pd_2_(dba)_3_ (0.005 mmol, 2.5 mol%), dppf (0.011 mmol, 5.5 mol%), *N*‐sulfonyl ketimine 3 (0.2 mmol), and cyano‐TMM donor **2** (0.3 mmol, 1.5 equiv.) were dissolved in toluene (2.0 mL) and stirred at 30 °C for 2 h. Upon completion of the reaction, as determined by TLC analysis, NaO*t*Bu (0.8 mmol, 4 equiv.) and THF (2.0 mL) were added, and the resulting mixture was stirred at 30 °C for an additional 30 min. The reaction was then quenched with saturated aqueous NH_4_Cl solution, and the mixture was extracted with EtOAc (3 ×). The combined organic layers were washed with brine, dried over anhydrous MgSO_4_, filtered, and concentrated under reduced pressure. The crude product was purified by silica gel column chromatography to afford compound **5**.

### General Procedure for the Synthesis of 2*H*‐Pyrrole **6**

Under an argon atmosphere, Pd_2_(dba)_3_ (0.005 mmol, 2.5 mol%), dppf (0.011 mmol, 5.5 mol%), *N*‐sulfonyl ketimine **3** (0.2 mmol), and cyano‐TMM donor **2** (0.3 mmol, 1.5 equiv.) were dissolved in toluene (2.0 mL) and stirred at 30 °C for 2 h. Upon completion of the reaction, as determined by TLC analysis, NaO*t*Bu (0.4 mmol, 2 equiv.) and DMF (2.0 mL) were added, and the resulting mixture was stirred at 80 °C for an additional 30 min. The reaction was then quenched with saturated aqueous NH_4_Cl solution, and the mixture was extracted with EtOAc (3 ×). The combined organic layers were washed with brine, dried over anhydrous MgSO_4_, filtered, and concentrated under reduced pressure. The crude product was purified by silica gel column chromatography to afford compound **6**.

## Conflict of Interest

The authors declare no conflict of interest.

## Supporting information



Supporting Information

## Data Availability

The data that support the findings of this study are available from the corresponding author upon reasonable request. (refer to the Supporting Information for additional details). [CCDC 2 421 296, CCDC 2 421 297, CCDC 2 432 149 contain the supplementary crystallographic data for this paper. These data can be obtained free of charge from The Cambridge Crystallographic Data Centre via www.ccdc.cam.ac.uk/data_request/cif.]
