# Supplementary material for: Asymmetric [3 + 2] Cycloaddition to Access 3‐Pyrrolines and Their Switchable Transformations to Nine‐Membered Cyclic Sulfamidates and 2H‐Pyrroles
Source: Adv Sci (Weinh). 2025 Nov 10;12(47):e13904. doi: 10.1002/advs.202513904 (PMC12713028; doi:10.1002/advs.202513904)
Supplement: Supplementary file 1 — Supporting Information [file ADVS-12-e13904-s001.docx]

Supporting Information

Asymmetric [3 + 2] Cycloaddition to Access 3-Pyrrolines and Their Switchable Transformations to Nine-Membered Cyclic Sulfamidates and 2*H*-Pyrroles

Seoung-Mi Choi^[a]^, Jong-Un Park^[b]^ and Ju Hyun Kim^[a]^*

^[a]^Department of Chemistry, Dongguk University, Seoul 04620, Korea
E-mail: [juhyunkim@dgu.edu](mailto:juhyunkim@dgu.edu)

^[b]^Department of Chemistry (BK21 Four), Gyeongsang National University, Jinju 52828, Korea

**Table of contents**

1. General Information ------------------------------------------------------------------ 2

2. General Procedure for The Synthesis of **3v** --------------------------------------- 3

3. Reaction Optimization --------------------------------------------------------------- 5

4. Mechanism Study -------------------------------------------------------------------- 7

5. General Procedure for The Synthesis of **4** --------------------------------------- 11

6. Characterization of The Compounds **4** ------------------------------------------- 13

7. General procedure for the synthesis of **5** or **6** ------------------------------------ 22

8. Characterization of The Compounds **5 ------**------------------------------------- 23

9. Characterization of The Compounds **6** ------------------------------------------- 29

10. Single Crystal X-ray Diffraction Data ------------------------------------------ 35

11. References -------------------------------------------------------------------------- 41

12. NMR Spectra of All Compounds ------------------------------------------------ 42

13. HPLC Chromatograms of The Synthesized Compounds **4**------------------ 103

**1. General Information**

All reactions and manipulations were carried out under an argon atmosphere using oven-dried Schlenk techniques. The reaction flasks were flamed dried, and solvents were transferred by oven-dried syringe. Toluene and THF were distilled at UltimaOrganic Solvent purification System (Purification capacity: 400~800 L/column using ACS level or high). Commercially available chemical reagents and other anhydrous solvents from Aldrich Chemical Co., Acros Organics, Alfa Aesar, and TCI were directly used without further purification. Thin-layer Chromatography (TLC) was performed on silica gel 60 F_254_ aluminum plates (Merck). TLC plates were visualized by short-wave ultraviolet light (254 and 365 nm) and/or KMnO_4_ solution. Flash chromatography was performed on Merck silica gel (60–200 mesh) by standard techniques. The ^1^H, ^13^C and ^19^F NMR spectra were recorded on Bruker DRX-300 and Bruker DRX-500, and the chemical shifts (δ) in ^1^H, ^13^C and ^19^F NMR spectra are given in ppm relative to TMS. (CDCl_3_: δ ^1^H = 7.26 ppm, δ ^13^C = 77.16 ppm). High-resolution mass spectrometric (HRMS) analyses were performed by electrospray ionization (ESI) using a SYNAPT G2 (Waters, U.K.) and a Xevo G2-XS QTOF (Waters, Milford, MA, USA), and by electron ionization (EI) using a JEOL JMS-700 spectrometer. Enantiomeric excesses (% ee) were determined by HPLC analysis (YL9100) using appropriate commercial Daicel Chiralpak columns as stated in the experimental procedures.

CpPd(allyl)^1^ and Phosphoramidite ligands^2^ **L1**, **L3**, **L5**-**L11** was synthesized according to the previously reported procedure. Phosphoramidite ligands **L2**, **L4**, **L12**, **L13**, and Ming-Phos **L14** were purchased from Aldrich and Strem Chemical.

(11b*S*)-N,N-bis((S)-1-phenylethyl)dinaphtho[2,1-d:1',2'-f][1,3,2]dioxaphosphepin-4-amine **L1**

5-((11b*S*)-dinaphtho[2,1-d:1',2'-f][1,3,2]dioxaphosphepin-4-yl)-5H-dibenzo[b,f]azepine **L2**

(11b*S*)-N,N-diisopropyldinaphtho[2,1-d:1',2'-f][1,3,2]dioxaphosphepin-4-amine **L3**

(11b*S*)-N-((*R*)-1-phenylethyl)dinaphtho[2,1-d:1',2'-f][1,3,2]dioxaphosphepin-4-amine **L4**

(11b*S*)-N,N-dimethyldinaphtho[2,1-d:1',2'-f][1,3,2]dioxaphosphepin-4-amine **L5**

(11b*S*)-N-benzyl-N-methyldinaphtho[2,1-d:1',2'-f][1,3,2]dioxaphosphepin-4-amine **L6**

(11b*S*)-N-benzyl-N-((*S*)-1-phenylethyl)dinaphtho[2,1-d:1',2'-f][1,3,2]dioxaphosphepin-4-amine **L7**

(11b*S*)-N,N-bis((*S*)-1-phenylethyl)-8,9,10,11,12,13,14,15-octahydrodinaphtho[2,1-d:1',2'-f][1,3,2]dioxaphosphepin-4-amine **L8**

(11b*S*)-N,N-diisopropyl-8,9,10,11,12,13,14,15-octahydrodinaphtho[2,1-d:1',2'-f][1,3,2]dioxaphosphepin-4-amine **L9**

(11b*S*)-N,N-dimethyl-8,9,10,11,12,13,14,15-octahydrodinaphtho[2,1-d:1',2'-f][1,3,2]dioxaphosphepin-4-amine **L10**

(11a*R*)-N,N-Bis((*S*)-1-phenylethyl)-4,5,6,7-tetrahydrodiindeno[7,1-de:1',7'-fg][1,3,2]dioxaphosphocin-12-amine **L11**

(2*S*,4*S*)-Pentane-2,4-diylbis(diphenylphosphine), (*S*,*S*)-BDPP **L12**

(2*R*)-1-[(1*R*)-1-[Bis(1,1-dimethylethyl)phosphino]ethyl]-2-(diphenylphosphino)ferrocene **L13**

(*R*)-N-((*S*)-(2-(diphenylphosphino)phenyl)(phenyl)methyl)-N,2-dimethylpropane-

2-sulfinamide **L14**

**2. Synthesis of Starting Materials 2 and 3**

Trimethylenemethane derivative **2**^3^ and sulfamidate imine **3**^4^ were synthesized according to the previously reported procedure.

**2.1 Synthesis of Late-Stage Functionalization Starting Material 3y**

The compound **S3** was synthesized according to the previously reported procedure.^5-7^

Following a reported procedure,^4b^ **S1** (0.46 mmol) was dissolved in toluene (9 mL) in a flask, and chlorosulfonyl isocyanate (2 equiv.) was added to the reaction mixture. The flask was placed in the reaction system at 100 °C and refluxed for 12 h. The reaction mixture was dissolved in ethyl acetate. Dried by a rotary evaporator, the residue was purified by column chromatography (Hexane/EtOAc = 3/1) to obtain the product **3y** in 33% (34.4 mg) as a slightly yellow solid. R*_f_* = 0.3 (Hexane/EtOAc = 3/1), mp: 240−242 ^o^C, ^1^H NMR (500 MHz, Chloroform-*d*) δ 7.76 (dd, *J* = 8.2, 1.3 Hz, 2H), 7.71 – 7.66 (m, 1H), 7.58 (t, *J* = 7.7 Hz, 2H), 7.52 (d, *J* = 1.1 Hz, 1H), 7.13 (s, 1H), 3.12 – 3.00 (m, 2H), 2.52 (dd, *J* = 19.0, 8.8 Hz, 1H), 2.29 (td, *J* = 10.7, 4.3 Hz, 1H), 2.20 – 2.13 (m, 2H), 2.12 – 2.05 (m, 2H), 1.95 – 1.89 (m, 1H), 1.69 – 1.57 (m, 3H), 1.55 – 1.40 (m, 5H), 0.91 (s, 3H). ^13^C NMR (126 MHz, Chloroform-*d*) δ 220.2, 176.6, 152.6, 148.3, 138.2, 134.0, 133.3, 130.7, 129.0, 128.9, 119.2, 114.4, 50.4, 47.9, 43.9, 37.7, 35.9, 31.4, 30.2, 25.9, 25.7, 21.7, 13.9. HRMS (EI) *m/z* calcd for C_25_H_25_NO_4_S [M]^+^: 435.1504, Found: 435.1507.

**2.2 NMR Spectra of The Compound 3y**


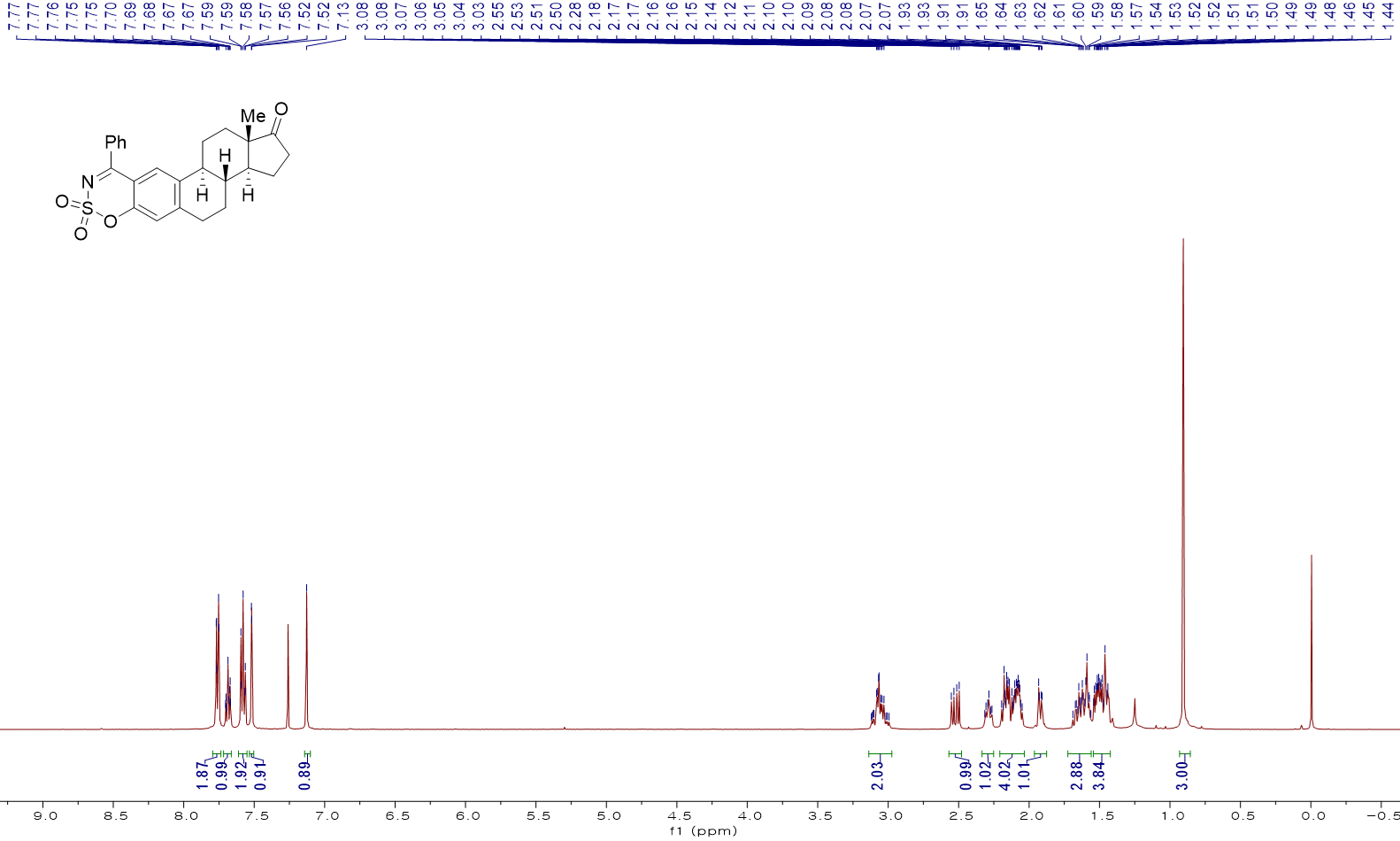


**Figure S1.** ^1^H NMR spectra of **3y**


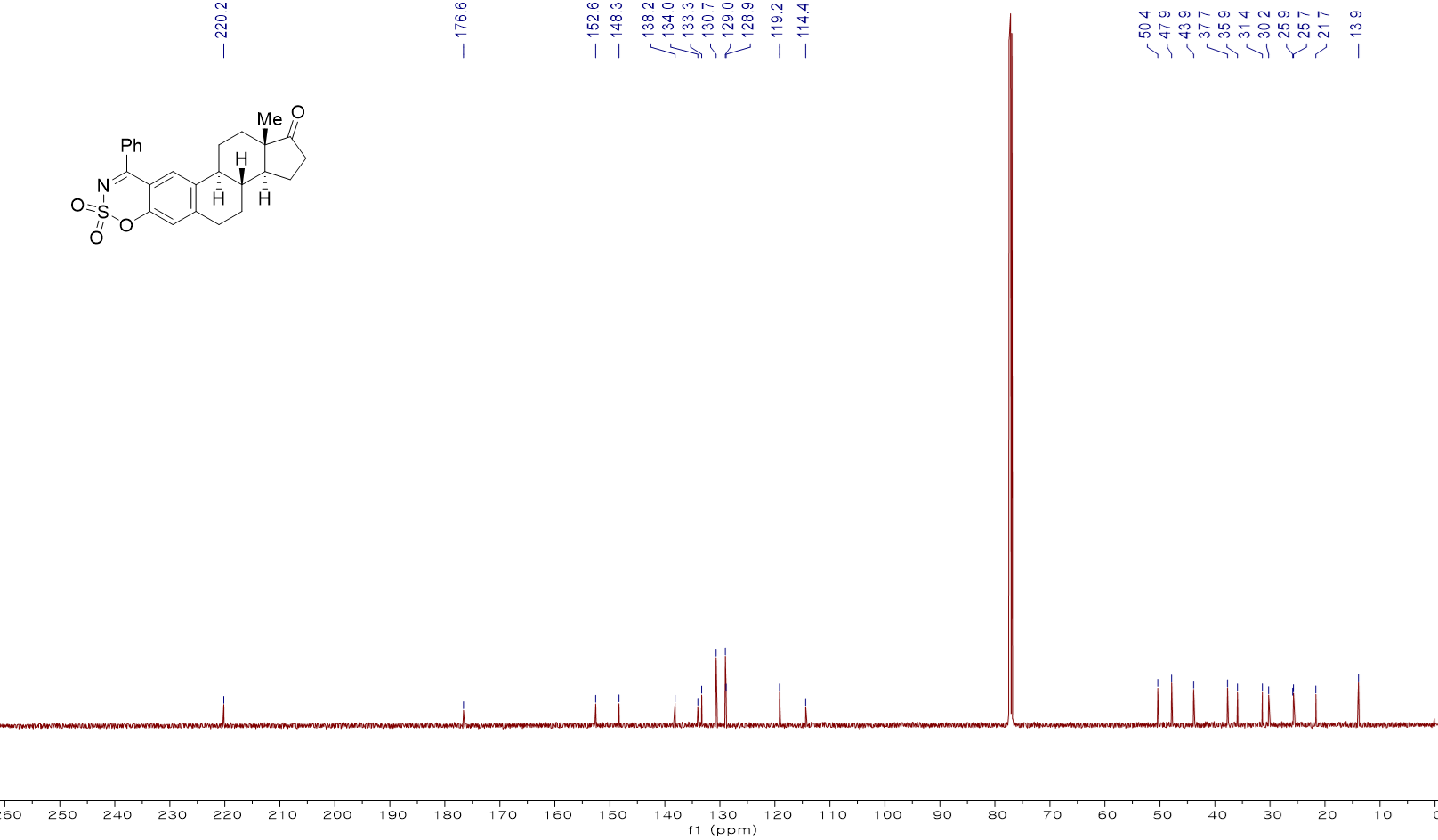


**Figure S2.** ^13^C NMR spectra of **3y**

**3. Reaction Optimization**

**Table S1.** Reaction optimization of the Pd catalyzed asymmetric cycloaddtion of *N*-sulfonyl ketimine (**3a**) with cyano-TMM donor (**1** or **2**).^a)^

| Entry | Pd  (mol%) | Ligand  (mol%) | Solvent | Temp (^o^C) | Yield **4a** (%)^b)^ | % ee^c)^ |
| --- | --- | --- | --- | --- | --- | --- |
| 1 | Pd_2_(dba)_3_ (2.5) | **L1** (11) | toluene | 30 | 67 | 78 |
| 2^d)^ | Pd_2_(dba)_3_ (2.5) | **L1** (11) | toluene | 30 | 56 | 78 |
| 2 | Pd_2_(dba)_3_ (2.5) | **L2** (11) | toluene | 30 | 55 | 39 |
| 3 | Pd_2_(dba)_3_ (2.5) | **L3** (11) | toluene | 30 | 31 | 46 |
| 4 | Pd_2_(dba)_3_ (2.5) | **L4** (11) | toluene | 30 | trace | - |
| 5 | Pd_2_(dba)_3_ (2.5) | **L5** (11) | toluene | 30 | 65 | 78 |
| 6 | Pd_2_(dba)_3_ (2.5) | **L6** (11) | toluene | 30 | 21 | 44 |
| 7 | Pd_2_(dba)_3_ (2.5) | **L7** (11) | toluene | 30 | 58 | 78 |
| 8^e)^ | Pd_2_(dba)_3_ (2.5) | **L8** (11) | toluene | 30 | 80 | 78 |
| 9 | Pd_2_(dba)_3_ (2.5) | **L9** (11) | toluene | 30 | 35 | 35 |
| 10 | Pd_2_(dba)_3_ (2.5) | **L10** (11) | toluene | 30 | 64 | 29 |
| 11 | Pd_2_(dba)_3_ (2.5) | **L11** (11) | toluene | 30 | 29 | 55 |
| 12 | Pd_2_(dba)_3_ (2.5) | **L12** (5.5) | toluene | 30 | N.R. | - |
| 13 | Pd_2_(dba)_3_ (2.5) | **L13** (5.5) | toluene | 30 | N.R. | - |
| 14^e)^ | Pd_2_(dba)_3_ (2.5) | **L1** (11) | toluene | 0 | 97 | 83 |
| 15^e)^ | Pd_2_(dba)_3_ (2.5) | **L1** (11) | toluene | -15 | 96 | 87 |
| 16 | CpPd(allyl) (5) | **L1** (11) | toluene | -15 | 74 | 90 |
| 17 | [Pd(allyl)Cl]_2_ (2.5) | **L1** (11) | toluene | -15 | N.R. | - |
| 18 | CpPd(π-cinnamyl) (2.5) | **L1** (11) | toluene | -15 | 43 | 78 |
| 19 | [Pd(π-cinnamyl)Cl]_2_ (5) | **L1** (11) | toluene | -15 | N.R. | - |
| 20^e)^ | CpPd(allyl) (5) | **L1** (11) | toluene | -15 | 95 | 90 |
| 21 | CpPd(allyl) (5) | **L1** (11) | DCM | -15 | 38 | 58 |
| 22 | CpPd(allyl) (5) | **L1** (11) | DME | -15 | 54 | 85 |
| 23 | CpPd(allyl) (5) | **L1** (11) | PhCl | -15 | 59 | 82 |
| 24 | CpPd(allyl) (5) | **L1** (11) | MTBE | -15 | 70 | 92 |
| 25 | CpPd(allyl) (5) | **L1** (11) | MTBE | 0 | 88 | 92 |
| ^a)^Reaction condition: **1a** (0.1 mmol), **2a** (0.1 mmol), Pd (5 mol% based on Pd), and ligand (11 mol%) in solvent (1.0 mL) for 16 h; ^b)^Isolated yield; ^c)^Determined by HPLC using a chiral stationary phase; ^d^^)^**1** was used instead of **2** for cyano-donor; ^e)^Reaction was performed with 1.5 equiv. of **2a**; N.R.= No Reaction; MTBE = Methyl tert-butyl ether. | | | | | | |

**4. Mechanism Study**

**4.1 NMR Experiments of Double-bond Isomerization Reaction**

To verify the formation of cycloadducts via double-bond isomerization, the cycloaddition reaction of **3y** with **2** was carried out in an NMR tube under the catalytic conditions of PdCp(allyl) (5 mol%) and **L1** (11 mol%) in toluene-d₈ at 30 ℃ for 2 h. The reaction mixture was then monitored by ¹H NMR spectroscopy. The NMR tube was subsequently maintained at 30, 60, and 80 ℃ for different periods, and ¹H NMR spectra were recorded after each interval (Figure S3). In the spectra, the H_b_ proton signal of compound **4x′** was detected at 30 ℃, indicating its initial formation. Upon increasing the temperature to 60 ℃ and 80 ℃, the H_b_ proton of **4x** gradually disappeared while the H_a_ signal of **4x** emerged, providing clear evidence that the endo-cyclic compound was formed through a double-bond isomerization process.

**
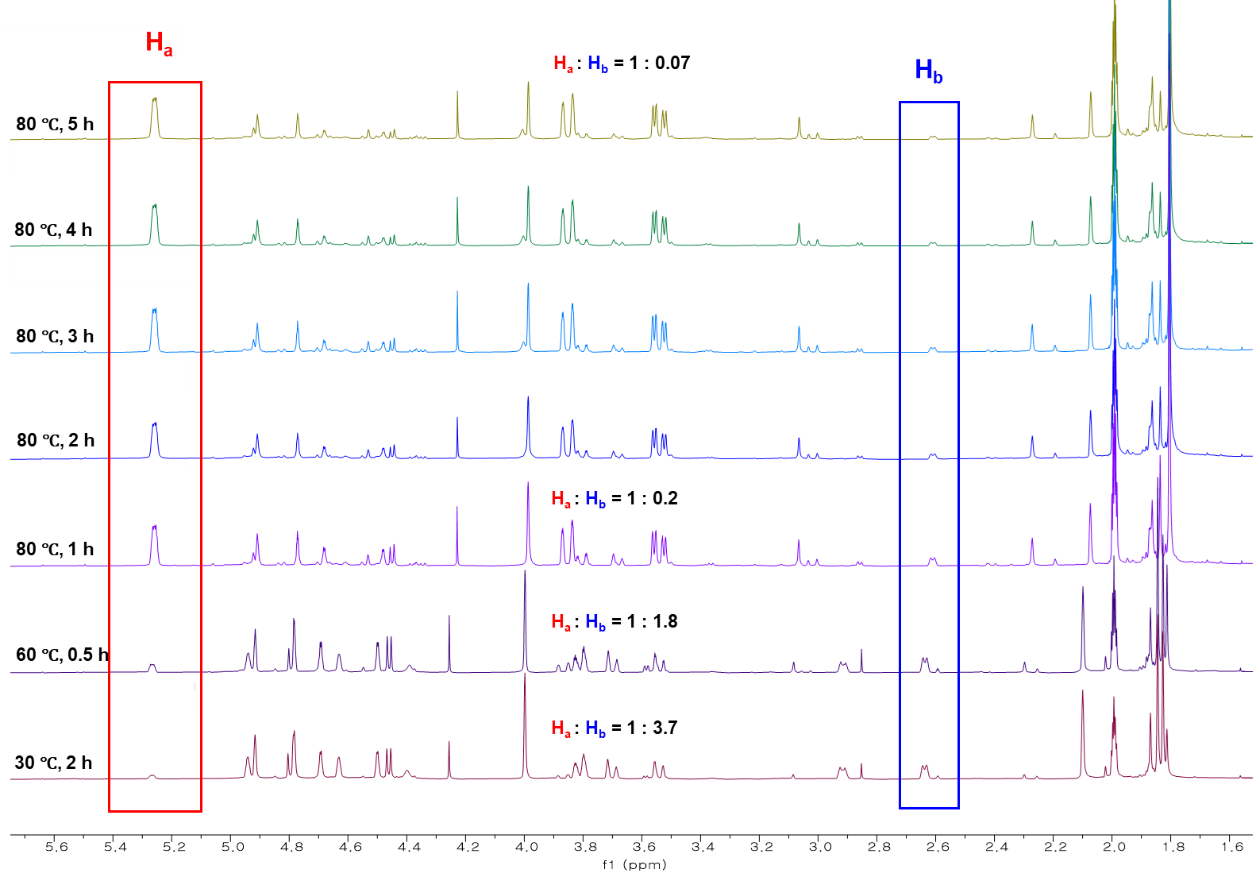
**

**Figure S3.** ^1^H NMR spectra of double-bond isomerization in Toluene-d_8_

**4.2 Procedure of The Synthesis of Compound 6a for Mechanism Study**

Under an argon atmosphere, 3-pyrroline **4a** or oxathiazonine **5a** (0.10 mmol, 1.0 equiv.) and NaO*t*Bu (19.2 mg, 0.20 mmol, 2.0 equiv.) were dissolved in anhydrous DMF (1.0 mL). The reaction mixture was stirred at 80 ℃. Upon completion, the reaction was quenched with a saturated aqueous solution of NH₄Cl, and the resulting mixture was extracted with EtOAc (3 × 5 mL). The combined organic layers were washed with brine, dried over anhydrous MgSO₄, filtered, and concentrated under reduced pressure. The crude residue was purified by flash column chromatography on silica gel to afford 2*H*-pyrrole **6a** in 48% and 70% yield, respectively.

(determined by HPLC analysis Daicel Chiralpak AD-H, eluent: n-hexane/iPrOH=95/5, flow rate: 1.0 mL/min, λ = 254 nm).

**4.3 HPLC Spectra of 2*H*-Pyrrole 6a**


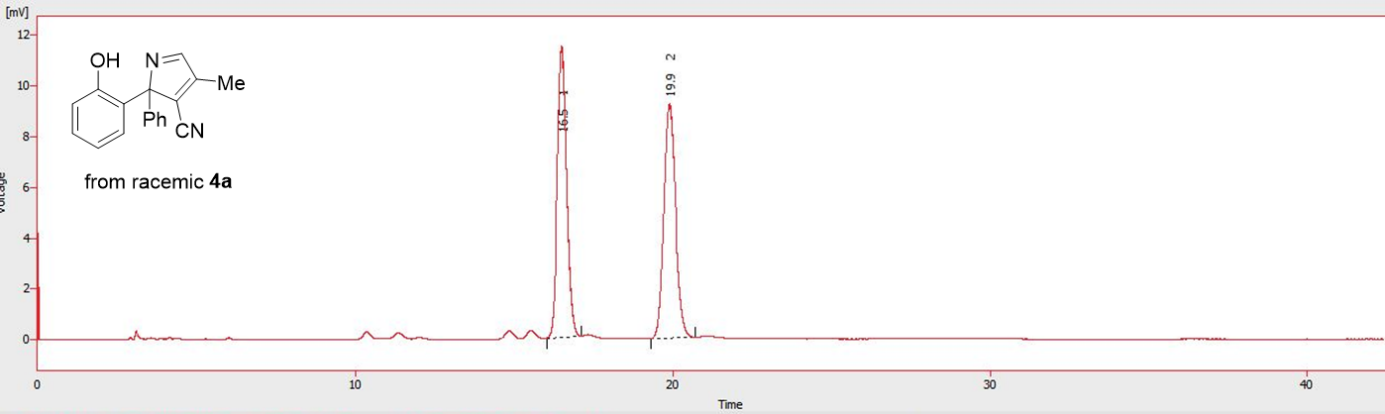


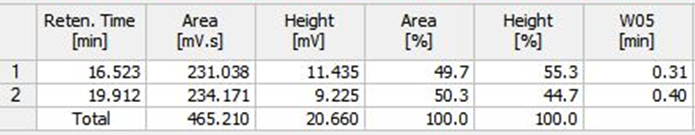


**Figure S4**. For **6a** from racemic **4a**


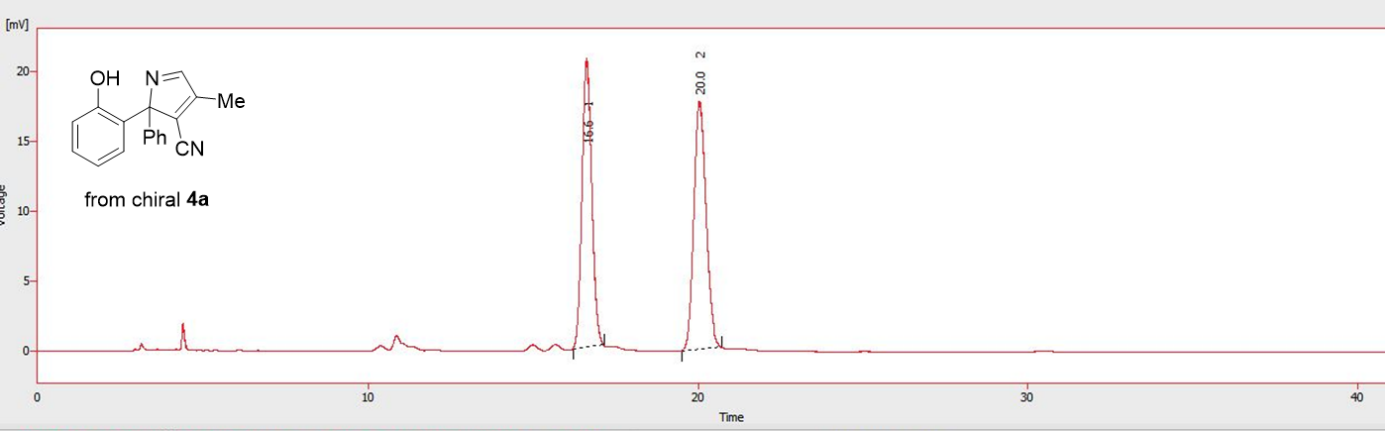


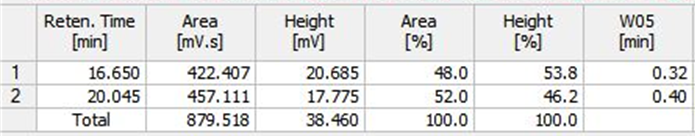


**Figure S5.** For **6a** from chiral **4a**

**4.4 NMR Experiments of Desulfonylation Reaction**

To investigate whether desulfonylation occurs via **5a** as an intermediate, the desulfonylation reaction of **4a** with NaO*^t^*Bu in DMF-d_7_ was conducted and analyzed by ^1^H NMR. To the NMR tube, **4a** (0.05 mmol, 1 equiv) and NaO*^t^*Bu (2 equiv.) were dissolved in DMF-d_7_. The NMR tube was maintained at room temperature, 30 ℃, 50 ℃, and 80 ℃ for different periods of time, and ¹H NMR spectra were recorded after each time. The results are shown in Figure S6. In the NMR spectra, we confirmed the H_b_ proton of compound **5a** and observed its formation at room temperature. When the temperature was raised to 50 ℃ and 80 ℃, the disappearance of the H_b_ proton of **5a** and the concomitant appearance of the H_c_ proton of **6a** were observed, thereby providing evidence that the transformation of **4a** into **6a** occurs through **5a** as an intermediate.

**
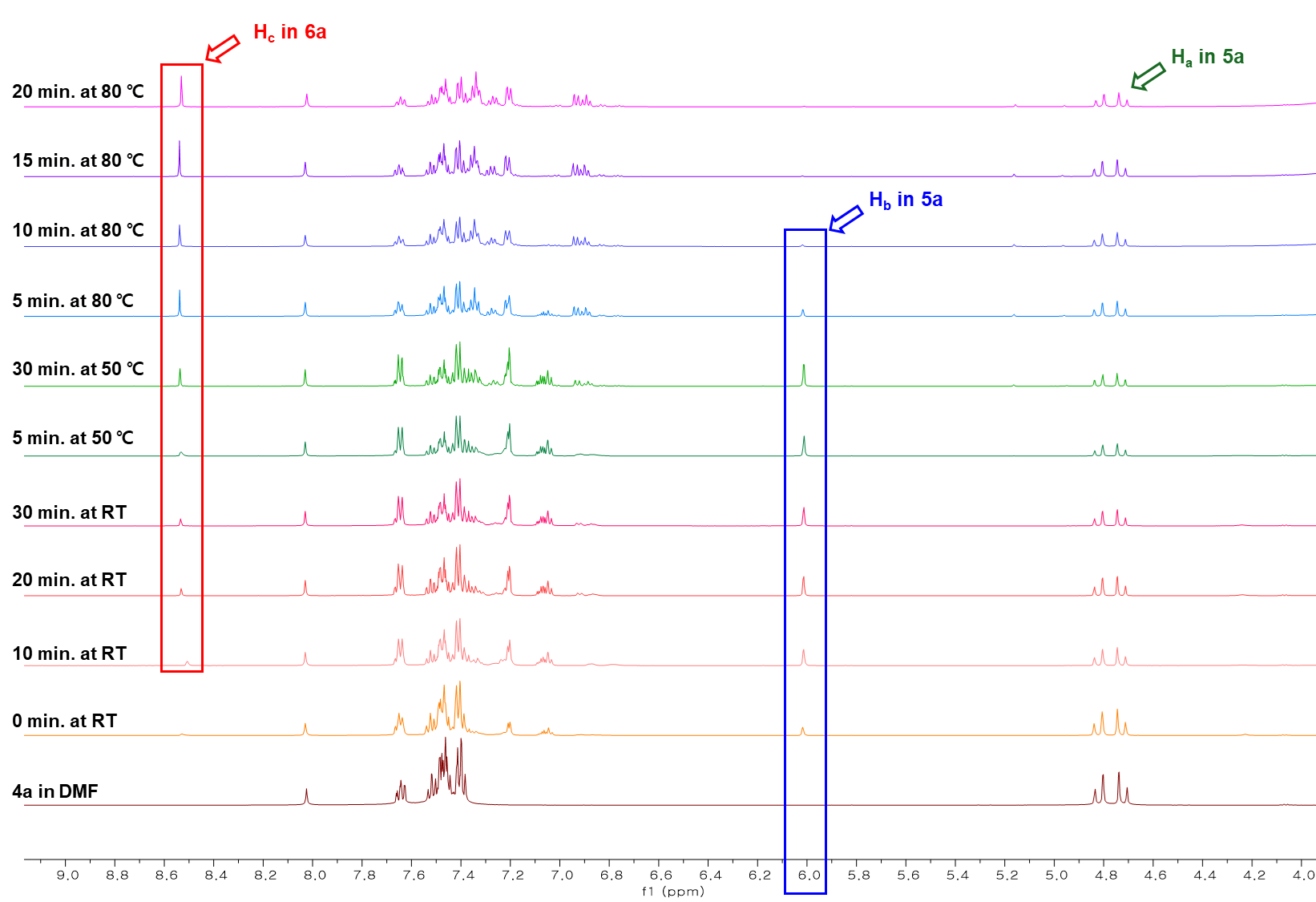
**

**Figure S6.** ^1^H NMR spectra of the desulfonylation reaction of **4a** in DMF-d_7_

**5. General Procedure for The Synthesis of 4**

**5.1. General Procedure for Preparation of The Chiral Products**

Under an argon atmosphere, CpPd(allyl) (2.1 mg, 0.01 mmol, 5 mol%) and ligand **L1** (5.94 mg, 0.022 mmol, 11 mol%) were dissolved in Toluene or MTBE (2.0 mL), and stirred at room temperature for approximately 15 min. Then, the mixture was stirred at -15 °C or 0 ^o^C for 5 min. *N*-sulfonyl ketimine **3** (0.2 mmol) and cyano-TMM donor **2** (59.2 mg, 0.3 mmol, 1.5 equiv.) were added sequentially under an argon atmosphere. The reaction mixture was stirred at -15 ℃ or 0 ℃ for 16 h. After the reaction was completed as determined by TLC analysis, the solvent was evaporated under reduced pressure, and the residue was purified by silica gel column chromatography to afford **4.** The enantiomeric excess (% ee) was recorded by HPLC (Daicel Chiralpak).

**5.2. General Procedure for Preparation of the Racemic Products**

Under an argon atmosphere, Pd_2_(dba)_3_ (2.2 mg, 0.0025 mmol, 2.5 mol%) and dppf (3.0 mg, 0.0055 mmol, 5.5 mol%) were dissolved in Toluene (1.0 mL), and stirred at room temperature for approximately 15 min. Then, the mixture was stirred at -15 ℃ for 5 min. *N*-sulfonyl ketimine **3** (0.1 mmol) and cyano-TMM donor **2** (29.6 mg, 0.15 mmol, 1.5 equiv.) were added sequentially under argon atmosphere. The reaction mixture was stirred at 30 ℃ for 2 h. After reaction completed as determined by TLC ananlysis, the solvent was evaporated under reduced pressure and the residue was purified by silica gel column chromatography to afford **4.**

**5.3 Procedure for the 1 mmol Scale Synthesis of 4a**

Under an argon atmosphere, CpPd(allyl) (10.6 mg, 0.05 mmol, 5 mol%) and ligand **L1** (59.4 mg, 0.11 mmol, 11 mol%) were dissolved in Toluene (10.0 mL), and stirred at room temperature for approximately 15 min. Then, the mixture was stirred at -15 ℃ for 5 min. *N*-sulfonyl ketimine **3** (1 mmol) and cyano-TMM donor **2** (295.8 mg, 1.5 mmol, 1.5 equiv.) were added sequentially under an argon atmosphere. The reaction mixture was stirred at -15 ℃ for 24 h. After the reaction was completed as determined by TLC analysis, the solvent was evaporated under reduced pressure, and the residue was purified by silica gel column chromatography to afford **4a.** The desired product **4a** was formed in 97% (0.328 g) yield as a white solid, 88% ee. (determined by HPLC analysis Daicel Chiralpak AD-H, eluent: n-hexane/iPrOH=99/1, flow rate: 1.0 mL/min, λ = 254 nm).

**6. Characterization of The Compounds 4a‒4y**

**(*S*)-(2-methyl-10b-phenyl-3,10b-dihydrobenzo[e]pyrrolo[1,2-c][1,2,3]oxathiazine-1-carbonitrile 5,5-dioxide (4a)**

White solid (64.2 mg, yield: 95%); mp: 214−216 ℃; purification by silica gel chromatography (ethyl acetate: *n*-hexane= 1:5, R*_f_* = 0.4); 90% ee, determined by HPLC analysis [Daicel Chiralpak AD-H, *n*-hexane/*i*-PrOH = 99/1, 1.0 mL/min, λ = 254 nm, t_major_ = 33.87 min, t_minor_ = 31.62 min]; ^1^H NMR (300 MHz, Chloroform-*d*) δ 7.50 – 7.42 (m, 2H), 7.42 – 7.36 (m, 3H), 7.31 (dddd, *J* = 7.5, 4.6, 2.8, 1.3 Hz, 3H), 7.17 (dd, *J* = 8.2, 1.4 Hz, 1H), 4.60 – 4.51 (m, 1H), 4.49 – 4.41 (m, 1H), 2.18 (d, *J* = 1.2 Hz, 3H).; ^13^C NMR (75 MHz, Chloroform-*d*) δ 153.4, 150.0, 139.0, 130.7, 129.5, 128.9, 128.7, 128.4, 126.0, 121.6, 120.0, 115.0, 113.4, 80.1, 60.0, 14.6.; HRMS (EI) *m/z* calcd for C_18_H_14_N_2_O_3_S [M]^+^: 338.0725, Found: 338.0723.

**(*S*)-2,7-dimethyl-10b-phenyl-3,10b-dihydrobenzo[e]pyrrolo[1,2-c][1,2,3]oxathiazine-1-carbonitrile 5,5-dioxide (4b)**

White solid (66.9 mg, yield: 95%); mp: 155−157 ℃; purification by silica gel chromatography (ethyl acetate: *n*-hexane= 1:5, R*_f_* = 0.35); 91% ee, determined by HPLC analysis [Daicel Chiralpak AD-H, *n*-hexane/*i*-PrOH = 95/5, 1.0 mL/min, λ = 254 nm, t_major_ = 11.35 min, t_minor_ = 9.94 min]; ^1^H NMR (300 MHz, Chloroform-*d*) δ 7.41 – 7.36 (m, 3H), 7.34 – 7.27 (m, 4H), 7.18 (dd, *J* = 8.3, 7.0 Hz, 1H), 4.59 – 4.41 (m, 2H), 2.36 (s, 3H), 2.17 (d, *J* = 1.2 Hz, 3H).; ^13^C NMR (75 MHz, Chloroform-*d*) δ 153.2, 148.5, 139.3, 132.0, 129.4, 128.6, 128.5, 126.4, 125.2, 121.4, 115.1, 113.4, 80.3, 77.6, 77.4, 77.2, 76.7, 60.0, 16.0, 14.6.; HRMS (EI) *m/z* calcd for C_19_H_16_N_2_O_3_S [M]^+^: 352.0882, Found: 352.0881.

**(*S*)-7-methoxy-2-methyl-10b-phenyl-3,10b-dihydrobenzo[e]pyrrolo[1,2-c][1,2,3]oxathiazine-1-carbonitrile 5,5-dioxide (4c)**

White solid (69.9 mg, yield: 95%); mp: 189−191 ℃; purification by silica gel chromatography (ethyl acetate: *n*-hexane= 1:5, R*_f_* = 0.35); 83% ee, determined by HPLC analysis [Daicel Chiralpak AD-H, *n*-hexane/*i*-PrOH = 99/1, 1.0 mL/min, λ = 254 nm, t_major_ = 74.44 min, t_minor_ = 66.72 min]; ^1^H NMR (300 MHz, Chloroform-*d*) δ 7.37 (qd, *J* = 3.5, 2.9, 1.8 Hz, 3H), 7.35 – 7.29 (m, 2H), 7.22 (t, *J* = 8.1 Hz, 1H), 7.05 – 6.98 (m, 2H), 4.50 (qd, *J* = 16.6, 1.2 Hz, 2H), 3.93 (s, 3H), 2.16 (d, *J* = 1.1 Hz, 3H).; ^13^C NMR (75 MHz, Chloroform-*d*) δ 153.5, 149.6, 139.7, 138.9, 129.4, 128.6, 128.5, 125.5, 119.8, 114.8, 113.4, 112.7, 80.3, 77.6, 77.4, 77.2, 76.7, 60.0, 56.4, 14.6.; HRMS (EI) *m/z* calcd for C_19_H_16_N_2_O_4_S [M]^+^: 368.0831, Found: 368.0829.

**(*S*)-7-fluoro-2-methyl-10b-phenyl-3,10b-dihydrobenzo[e]pyrrolo[1,2-c][1,2,3]oxathiazine-1-carbonitrile 5,5-dioxide (4d)**

White solid (67.6 mg, yield: 95%); mp: 224−226 ℃; purification by silica gel chromatography (ethyl acetate: *n*-hexane= 1:5, R*_f_* = 0.3); 90% ee, determined by HPLC analysis [Daicel Chiralpak AD-H, *n*-hexane/*i*-PrOH = 95/5, 1.0 mL/min, λ = 254 nm, t_major_ = 15.54 min, t_minor_ = 14.99 min]; ^1^H NMR (300 MHz, CDCl_3_) δ 7.40 (dt, *J* = 5.5, 1.9 Hz, 3H), 7.31 (dtd, *J* = 7.8, 4.0, 2.2 Hz, 2H), 7.24 (s, 2H), 4.52 (qd, *J* = 16.6, 1.3 Hz, 2H), 2.18 (s, 3H).; ^13^C NMR (75 MHz, Chloroform-*d*) δ 153.7, 151.7 (d, *J* = 251.3 Hz), 138.84 (d, *J* = 14.3 Hz), 138.5, 129.6, 128.8, 128.4, 125.5 (d, *J* = 7.5 Hz), 123.7 (d, *J* = 3.8 Hz), 123.6, 117.4 (d, *J* = 17.3 Hz), 114.6, 113.2, 80.3 (d, *J* = 2.3 Hz), 60.0, 14.6.; ^19^F NMR (376 MHz, Chloroform-*d*) δ -130.7; HRMS (EI) *m/z* calcd for C_18_H_13_F N_2_O_3_S [M]^+^: 356.0631, Found: 356.0627.

**(*S*)-2,8-dimethyl-10b-phenyl-3,10b-dihydrobenzo[e]pyrrolo[1,2-c][1,2,3]oxathiazine-1-carbonitrile 5,5-dioxide (4e)**

White solid (66.2 mg, yield: 94%); mp: 235−237 ℃; purification by silica gel chromatography (ethyl acetate: *n*-hexane= 1:5, R*_f_* = 0.3); 87% ee, determined by HPLC analysis [Daicel Chiralpak AD-H, *n*-hexane/*i*-PrOH = 95/5, 1.0 mL/min, λ = 254 nm, t_major_ = 10.42 min, t_minor_ = 9.18 min]; ^1^H NMR (300 MHz, Chloroform-*d*) δ 7.41 – 7.29 (m, 6H), 7.15 – 7.09 (m, 1H), 6.98 (t, *J* = 1.2 Hz, 1H), 4.57 – 4.36 (m, 2H), 2.41 (s, 3H), 2.16 (d, *J* = 1.1 Hz, 3H).; ^13^C NMR (75 MHz, Chloroform-*d*) δ 153.1, 149.8, 141.4, 139.1, 129.4, 128.6, 128.5, 128.4, 126.9, 120.1, 118.5, 115.1, 113.4, 80.0, 77.6, 77.2, 76.7, 59.9, 21.2, 14.6.; HRMS (EI) *m/z* calcd for C_19_H_16_N_2_O_3_S [M]^+^: 352.0882, Found: 352.0878.

**(*S*)-8-methoxy-2-methyl-10b-phenyl-3,10b-dihydrobenzo[e]pyrrolo[1,2-c][1,2,3]oxathiazine-1-carbonitrile 5,5-dioxide (4f)**

White solid (50.1 mg, yield: 29%); mp: 209−211 ℃; purification by silica gel chromatography (ethyl acetate: *n*-hexane= 1:5, R*_f_* = 0.25); 92% ee, determined by HPLC analysis [Daicel Chiralpak AD-H, *n*-hexane/*i*-PrOH = 90/10, 1.0 mL/min, λ = 254 nm, t_major_ = 15.79 min, t_minor_ = 13.55 min]; ^1^H NMR (300 MHz, Chloroform-*d*) δ 7.35 (ddd, *J* = 18.3, 7.1, 3.9 Hz, 6H), 6.86 (dd, *J* = 8.9, 2.6 Hz, 1H), 6.67 (d, *J* = 2.6 Hz, 1H), 4.59 – 4.49 (m, 1H), 4.43 (d, *J* = 16.5 Hz, 1H), 3.84 (s, 3H), 2.15 (s, 3H).; ^13^C NMR (75 MHz, Chloroform-*d*) δ 161.0, 152.8, 150.8, 139.2, 129.5, 129.4, 128.6, 128.4, 115.2, 113.4, 113.3, 112.7, 104.6, 79.8, 59.9, 55.9, 14.5.; HRMS (EI) *m/z* calcd for C_19_H_16_N_2_O_4_S [M]^+^: 368.0831, Found: 368.0831.

**(*S*)-2,9-dimethyl-10b-phenyl-3,10b-dihydrobenzo[e]pyrrolo[1,2-c][1,2,3]oxathiazine-1-carbonitrile 5,5-dioxide (4g)**

White solid (62.0 mg, yield: 77%); mp: 173−176 ℃; purification by silica gel chromatography (ethyl acetate: *n*-hexane= 1:5, R*_f_* = 0.45); 86% ee, determined by HPLC analysis [Daicel Chiralpak AD-H, *n*-hexane/*i*-PrOH = 95/5, 1.0 mL/min, λ = 254 nm, t_major_ = 8.33 min, t_minor_ = 9.04 min]; ^1^H NMR (300 MHz, Chloroform-*d*) δ 7.42 – 7.37 (m, 3H), 7.35 – 7.30 (m, 2H), 7.26 – 7.21 (m, 2H), 7.05 (d, *J* = 9.0 Hz, 1H), 4.57 – 4.38 (m, 2H), 2.36 (s, 3H), 2.17 (d, *J* = 1.2 Hz, 3H).; ^13^C NMR (75 MHz, Chloroform-*d*) δ 153.4, 147.9, 139.1, 135.9, 131.3, 129.4, 128.8, 128.7, 128.5, 121.1, 119.6, 115.0, 113.4, 80.1, 77.6, 77.2, 76.7, 59.9, 21.1, 14.6.; HRMS (EI) *m/z* calcd for C_19_H_16_N_2_O_3_S [M]^+^: 352.0882, Found: 352.0879.

**(*S*)-9-(*tert*-butyl)-2-methyl-10b-phenyl-3,10b-dihydrobenzo[e]pyrrolo[1,2-c][1,2,3]oxathiazine-1-carbonitrile 5,5-dioxide (4h)**

White solid (75.0 mg, yield: 95%); mp: 89−91 ℃; purification by silica gel chromatography (ethyl acetate: *n*-hexane= 1:5, R*_f_* = 0.45); 84% ee, determined by HPLC analysis [Daicel Chiralpak AD-H, *n*-hexane/*i*-PrOH = 95/5, 1.0 mL/min, λ = 254 nm, t_major_ = 8.09 min, t_minor_ = 11.16 min]; ^1^H NMR (300 MHz, Chloroform-*d*) δ 7.49 – 7.43 (m, 2H), 7.41 – 7.37 (m, 3H), 7.34 – 7.29 (m, 2H), 7.09 (dd, *J* = 8.0, 1.0 Hz, 1H), 4.57 – 4.42 (m, 2H), 2.17 (d, *J* = 1.1 Hz, 3H), 1.30 (s, 9H).; ^13^C NMR (75 MHz, Chloroform-*d*) δ 153.0, 149.2, 147.7, 139.2, 129.4, 128.7, 128.4, 127.6, 126.1, 120.5, 119.2, 115.2, 113.5, 80.4, 77.6, 77.2, 76.7, 60.0, 34.9, 31.3, 14.6.; HRMS (EI) *m/z* calcd for C_22_H_22_N_2_O_3_S [M]^+^: 394.1351, Found: 394.1354.

**(*S*)-9-methoxy-2-methyl-10b-phenyl-3,10b-dihydrobenzo[e]pyrrolo[1,2-c][1,2,3]oxathiazine-1-carbonitrile 5,5-dioxide (4i)**

White solid (47.2 mg, yield: 64%); mp: 213−215 ℃; purification by silica gel chromatography (ethyl acetate: *n*-hexane= 1:5, R*_f_* = 0.35); 94% ee, determined by HPLC analysis [Daicel Chiralpak AD-H, *n*-hexane/*i*-PrOH = 90/10, 1.0 mL/min, λ = 254 nm, t_major_ = 7.29 min, t_minor_ = 9.17 min]; ^1^H NMR (300 MHz, Chloroform-*d*) δ 7.43 – 7.37 (m, 3H), 7.36 – 7.30 (m, 2H), 7.09 (d, *J* = 9.0 Hz, 1H), 7.00 – 6.91 (m, 2H), 4.57 – 4.38 (m, 2H), 3.76 (s, 3H), 2.18 (s, 3H).; ^13^C NMR (75 MHz, Chloroform-*d*) δ 156.9, 153.5, 143.6, 138.9, 129.5, 128.7, 128.5, 122.1, 120.8, 117.2, 114.8, 113.6, 112.3, 80.3, 77.6, 77.2, 76.7, 60.0, 56.0, 14.7.; HRMS (EI) *m/z* calcd for C_19_H_16_N_2_O_4_S [M]^+^: 368.0831, Found: 368.0828.

**(*S*)-9-fluoro-2-methyl-10b-phenyl-3,10b-dihydrobenzo[e]pyrrolo[1,2-c][1,2,3]oxathiazine-1-carbonitrile 5,5-dioxide (4j)**

White solid (69.8 mg, yield: 98%); mp: 160−162 ℃; purification by silica gel chromatography (ethyl acetate: *n*-hexane= 1:5, R*_f_* = 0.25); 90% ee, determined by HPLC analysis [Daicel Chiralpak AD-H, *n*-hexane/*i*-PrOH = 97/3, 1.0 mL/min, λ = 254 nm, t_major_ = 20.38 min, t_minor_ = 18.71 min]; ^1^H NMR (300 MHz, Chloroform-*d*) δ 7.41 (h, *J* = 2.5 Hz, 3H), 7.31 (ddd, *J* = 6.4, 2.7, 1.4 Hz, 2H), 7.21 – 7.13 (m, 3H), 4.60 – 4.38 (m, 2H), 2.18 (d, *J* = 1.2 Hz, 3H).; ^13^C NMR (75 MHz, Chloroform-*d*) δ 159.2 (d, *J* = 246.0 Hz), 153.9, 145.9 (d, *J* = 2.3 Hz), 138.4, 129.7, 128.8, 128.3, 123.2 (d, *J* = 6.6 Hz), 121.5 (d, *J* = 8.3 Hz), 117.9 (d, *J* = 23.3 Hz), 115.4 (d, *J* = 24.8 Hz), 114.4, 113.1, 80.0 (d, *J* = 1.5 Hz), 60.0, 14.6.; ^19^F NMR (376 MHz, Chloroform-*d*) δ -113.3.; HRMS (EI) *m/z* calcd for C_18_H_13_FN_2_O_3_S [M]^+^: 356.0631, Found: 356.0634.

**(*S*)-9-chloro-2-methyl-10b-phenyl-3,10b-dihydrobenzo[e]pyrrolo[1,2-c][1,2,3]oxathiazine-1-carbonitrile 5,5-dioxide (4k)**

White solid (69.9 mg, yield: 99%); mp: 138−141 ℃; purification by silica gel chromatography (ethyl acetate: *n*-hexane= 1:5, R*_f_* = 0.25); 90% ee, determined by HPLC analysis [Daicel Chiralpak AD-H, *n*-hexane/*i*-PrOH = 97/3, 1.0 mL/min, λ = 254 nm, t_major_ = 12.60 min, t_minor_ = 11.42 min]; ^1^H NMR (300 MHz, Chloroform-*d*) δ 7.47 – 7.39 (m, 5H), 7.34 – 7.28 (m, 2H), 7.12 (d, *J* = 8.7 Hz, 1H), 4.57 – 4.40 (m, 2H), 2.18 (s, 3H).; ^13^C NMR (75 MHz, Chloroform-*d*) δ 153.8, 148.5, 138.3, 131.3, 130.9, 129.7, 128.9, 128.4, 128.3, 123.2, 121.3, 114.5, 113.0, 79.9, 77.6, 77.2, 76.7, 59.9, 14.7.; HRMS (EI) *m/z* calcd for C_18_H_13_ClN_2_O_3_S [M]^+^: 372.0335, Found: 372.0337.

**(*S*)-9-bromo-2-methyl-10b-phenyl-3,10b-dihydrobenzo[e]pyrrolo[1,2-c][1,2,3]oxathiazine-1-carbonitrile 5,5-dioxide (4l)**

White solid (81.8 mg, yield: 98%); mp: 130−132 ℃; purification by silica gel chromatography (ethyl acetate: *n*-hexane= 1:5, R*_f_* = 0.25); 88% ee, determined by HPLC analysis [Daicel Chiralpak AD-H, *n*-hexane/*i*-PrOH = 98/2, 1.0 mL/min, λ = 254 nm, t_major_ = 12.53 min, t_minor_ = 11.60 min]; ^1^H NMR (300 MHz, Chloroform-*d*) δ 7.61 – 7.54 (m, 2H), 7.42 (dt, *J* = 4.3, 1.6 Hz, 3H), 7.34 – 7.27 (m, 2H), 7.06 (d, *J* = 8.6 Hz, 1H), 4.59 – 4.39 (m, 2H), 2.18 (s, 3H).; ^13^C NMR (75 MHz, Chloroform-*d*) δ 153.8, 149.0, 138.3, 133.8, 131.3, 129.7, 128.9, 128.3, 123.5, 121.6, 118.6, 114.5, 113.0, 79.8, 59.9, 14.6.; HRMS (EI) *m/z* calcd for C_18_H_13_BrN_2_O_3_S [M]^+^: 415.9830, Found: 415.9832.

**(*S*)-2-methyl-10b-(p-tolyl)-3,10b-dihydrobenzo[e]pyrrolo[1,2-c][1,2,3]oxathiazine-1-carbonitrile 5,5-dioxide** **(4m)**

White solid (49.3 mg, yield: 68%); mp: 81−83 ℃; purification by silica gel chromatography (ethyl acetate: *n*-hexane= 1:5, R*_f_* = 0.4); 86% ee, determined by HPLC analysis [Daicel Chiralpak AD-H, *n*-hexane/*i*-PrOH = 90/10, 1.0 mL/min, λ = 254 nm, t_major_ = 9.80 min, t_minor_ = 8.54 min]; ^1^H NMR (300 MHz, Chloroform-*d*) δ 7.45 (ddd, *J* = 12.9, 7.6, 1.7 Hz, 2H), 7.30 (td, *J* = 7.6, 1.3 Hz, 1H), 7.23 – 7.13 (m, 5H), 4.53 (dd, *J* = 16.6, 1.4 Hz, 1H), 4.44 (dd, *J* = 16.5, 1.2 Hz, 1H), 2.37 (s, 3H), 2.16 (s, 3H).; ^13^C NMR (75 MHz, Chloroform-*d*) δ 153.2, 150.0, 139.5, 136.1, 130.6, 129.4, 128.9, 128.3, 125.9, 121.8, 119.9, 115.0, 113.4, 80.0, 59.9, 21.3, 14.6.; HRMS (EI) *m/z* calcd for C_19_H_16_N_2_O_3_S [M]^+^: 352.0882, Found: 352.0878.

**(*S*)-10b-(4-methoxyphenyl)-2-methyl-3,10b-dihydrobenzo[e]pyrrolo[1,2-c][1,2,3]oxathiazine-1-carbonitrile 5,5-dioxide (4n)**

White solid (20.6 mg, yield: 28%); mp: 103−104 ℃; purification by silica gel chromatography (ethyl acetate: *n*-hexane= 1:5, R*_f_* = 0.35); 84% ee, determined by HPLC analysis [Daicel Chiralpak AD-H, *n*-hexane/*i*-PrOH = 90/10, 1.0 mL/min, λ = 254 nm, t_major_ = 15.62 min, t_minor_ = 12.25 min]; ^1^H NMR (300 MHz, Chloroform-*d*) δ 7.45 (ddd, *J* = 13.4, 7.6, 1.7 Hz, 2H), 7.30 (td, *J* = 7.6, 1.3 Hz, 1H), 7.23 – 7.13 (m, 3H), 6.93 – 6.86 (m, 2H), 4.56 – 4.39 (m, 2H), 3.81 (s, 3H), 2.16 (d, *J* = 1.2 Hz, 3H).; ^13^C NMR (75 MHz, Chloroform-*d*) δ 160.3, 153.1, 149.9, 131.0, 130.6, 129.9, 128.9, 125.9, 121.9, 119.9, 115.1, 114.0, 113.5, 80.0, 77.6, 77.2, 76.7, 59.8, 55.4, 14.7.; HRMS (EI) *m/z* calcd for C_19_H_16_N_2_O_4_S [M]^+^: 368.0831, Found: 368.0835.

**(*S*)-2-methyl-10b-(4-(trifluoromethyl)phenyl)-3,10b-dihydrobenzo[e]pyrrolo[1,2-c][1,2,3]oxathiazine-1-carbonitrile 5,5-dioxide (4o)**

White solid (79.6 mg, yield: 98%); mp: 99−101 ℃; purification by silica gel chromatography (ethyl acetate: *n*-hexane= 1:5, R*_f_* = 0.3); 85% ee, determined by HPLC analysis [Daicel Chiralpak AD-H, *n*-hexane/*i*-PrOH = 90/10, 1.0 mL/min, λ = 254 nm, t_major_ = 9.79 min, t_minor_ = 7.13 min]; ^1^H NMR (300 MHz, Chloroform-*d*) δ 7.66 (d, *J* = 8.3 Hz, 2H), 7.46 (ddd, *J* = 8.3, 7.0, 1.6 Hz, 4H), 7.34 (ddd, *J* = 8.2, 7.2, 1.3 Hz, 1H), 7.19 (dd, *J* = 8.2, 1.3 Hz, 1H), 4.62 – 4.52 (m, 1H), 4.47 (dd, *J* = 16.6, 1.2 Hz, 1H), 2.19 (d, *J* = 1.2 Hz, 3H).; ^13^C NMR (75 MHz, Chloroform-*d*) δ 154.3, 150.0, 142.9, 131.7, 131.3, 131.1, 129.0, 128.6, 126.2, 125.75 (q, *J* = 3.8f Hz)., 122.1, 120.8, 120.2, 114.4, 113.1, 79.5, 77.6, 77.2, 76.7, 60.0, 14.7; ^19^F NMR (376 MHz, Chloroform-*d*) δ -62.7.; HRMS (EI) *m/z* calcd for C_19_H_13_F_3_N_2_O_3_S [M]^+^: 406.0599, Found: 406.0596.

**(*S*)-10b-(4-bromophenyl)-2-methyl-3,10b-dihydrobenzo[e]pyrrolo[1,2-c][1,2,3]oxathiazine-1-carbonitrile 5,5-dioxide (4p)**

White solid (81.8 mg, yield: 98%); mp: 101−102 ℃; purification by silica gel chromatography (ethyl acetate: *n*-hexane= 1:5, R*_f_* = 0.35); 86% ee, determined by HPLC analysis [Daicel Chiralpak AD-H, *n*-hexane/*i*-PrOH = 90/10, 1.0 mL/min, λ = 254 nm, t_major_ = 15.99 min, t_minor_ = 9.99 min]; ^1^H NMR (300 MHz, Chloroform-*d*) δ 7.54 – 7.42 (m, 4H), 7.32 (ddd, *J* = 8.5, 7.0, 1.3 Hz, 1H), 7.21 – 7.14 (m, 3H), 4.57 – 4.39 (m, 2H), 2.17 (d, *J* = 1.1 Hz, 3H).; ^13^C NMR (75 MHz, Chloroform-*d*) δ 153.9, 149.9, 138.1, 131.9, 130.9, 130.2, 128.6, 126.1, 124.0, 121.1, 120.1, 114.5, 113.2, 79.6, 77.6, 77.4, 77.2, 76.7, 59.9, 14.7.; HRMS (EI) *m/z* calcd for C_18_H_13_BrN_2_O_3_S [M]^+^: 415.9830, Found: 415.9832.

**(*S*)-10b-(4-cyanophenyl)-2-methyl-3,10b-dihydrobenzo[e]pyrrolo[1,2-c][1,2,3]oxathiazine-1-carbonitrile 5,5-dioxide (4q)**

White solid (67.5 mg, yield: 93%); mp: 131−133 ℃; purification by silica gel chromatography (ethyl acetate: *n*-hexane= 1:5, R*_f_* = 0.4); 82% ee, determined by HPLC analysis [Daicel Chiralpak AD-H, *n*-hexane/*i*-PrOH = 95/5, 1.0 mL/min, λ = 254 nm, t_major_ = 28.62 min, t_minor_ = 25.69 min]; ^1^H NMR (300 MHz, Chloroform-*d*) δ 7.72 – 7.66 (m, 2H), 7.53 – 7.41 (m, 4H), 7.34 (ddd, *J* = 8.1, 7.2, 1.3 Hz, 1H), 7.19 (dd, *J* = 8.3, 1.2 Hz, 1H), 4.62 – 4.42 (m, 2H), 2.19 (d, *J* = 1.2 Hz, 3H).; ^13^C NMR (75 MHz, Chloroform-*d*) δ 154.7, 149.9, 143.9, 132.5, 131.2, 129.3, 128.4, 126.3, 120.3, 120.2, 118.2, 114.1, 113.4, 112.9, 79.3, 77.6, 77.2, 76.7, 59.9, 14.7.; HRMS (EI) *m/z* calcd for C_19_H_13_N_3_O_3_S [M]^+^: 363.0678, Found: 363.0677.

**Methyl (*S*)-4-(1-cyano-2-methyl-5,5-dioxidobenzo[e]pyrrolo[1,2-c][1,2,3]oxathiazin-10b(3*H*)-yl)benzoate (4r)**

White solid (69.7 mg, yield: 88%); mp: 163−165 ℃; purification by silica gel chromatography (ethyl acetate: *n*-hexane= 1:5, R*_f_* = 0.4); 81% ee, determined by HPLC analysis [Daicel Chiralpak AD-H, *n*-hexane/*i*-PrOH = 90/10, 1.0 mL/min, λ = 254 nm, t_major_ = 27.27 min, t_minor_ = 19.17 min]; ^1^H NMR (300 MHz, Chloroform-*d*) δ 8.08 – 8.03 (m, 2H), 7.51 – 7.43 (m, 2H), 7.42 – 7.36 (m, 2H), 7.33 (ddd, *J* = 8.4, 7.0, 1.3 Hz, 1H), 7.18 (dd, *J* = 8.6, 1.3 Hz, 1H), 4.59 – 4.44 (m, 2H), 3.92 (s, 3H), 2.18 (d, *J* = 1.1 Hz, 3H).; ^13^C NMR (75 MHz, Chloroform-*d*) δ 166.4, 154.0, 149.9, 143.6, 131.1, 130.9, 129.9, 128.6, 128.5, 126.1, 120.9, 120.1, 114.5, 113.1, 79.6, 77.6, 77.4, 77.2, 76.7, 59.9, 52.4, 14.7.; HRMS (EI) *m/z* calcd for C_20_H_16_N_2_O_5_S [M]^+^: 396.0780, Found: 396.0777.

**(*S*)-10b-cyclohexyl-2-methyl-3,10b-dihydrobenzo[e]pyrrolo[1,2-c][1,2,3]oxathiazine-1-carbonitrile 5,5-dioxide (4s)**

White solid (22.7 mg, yield: 66%); mp: 77−79 ℃; purification by silica gel chromatography (ethyl acetate: *n*-hexane= 1:7, R*_f_* = 0.45); 80% *ee*, determined by HPLC analysis [Daicel Chiralpak AD-H, *n*-hexane/*i*-PrOH = 96/4, 1.0 mL/min, λ = 254 nm, t_major_ = 7.8 min, t_minor_ = 8.7 min]; ^1^H NMR (500 MHz, Chloroform-*d*) δ 7.50 (dd, *J* = 7.3, 2.6 Hz, 1H), 7.33 (dtd, *J* = 26.8, 7.5, 2.1 Hz, 2H), 7.07 (dd, *J* = 8.1, 2.2 Hz, 1H), 4.68 (dd, *J* = 17.5, 3.2 Hz, 1H), 4.30 (dd, *J* = 17.6, 3.2 Hz, 1H), 2.30 (s, 1H), 2.05 (d, *J* = 3.2 Hz, 3H), 1.90 – 1.85 (m, 1H), 1.80 – 1.55 (m, 5H), 1.53 – 1.15 (m, 7H).; ^13^C NMR (126 MHz, Chloroform-*d*) δ 152.2, 150.1, 130.1, 126.7, 126.3, 122.8, 120.0, 113.9, 113.3, 82.5, 62.5, 45.7, 26.9, 26.7, 26.5, 26.4, 26.1, 14.2.; HRMS (ESI) *m/z* calcd for C_18_H_20_N_2_NaO_3_S^+^ [M+Na]^+^: 367.1087, Found: 367.1092.

**(*S*)-10b-ethyl-2-methyl-3,10b-dihydrobenzo[e]pyrrolo[1,2-c][1,2,3]oxathiazine-1-carbonitrile 5,5-dioxide (4t)**

White solid (27.6 mg, yield: 95%); mp: 149−151 ℃; purification by silica gel chromatography (Acetone: *n*-hexane= 1:5, R*_f_* = 0.5); 20% *ee*, determined by HPLC analysis [Daicel Chiralpak AD-H, *n*-hexane/*i*-PrOH = 94/6, 0.6 mL/min, λ = 254 nm, t_major_ = 14.5 min, t_minor_ = 15.1 min]; ^1^H NMR (500 MHz, Chloroform-*d*) δ 7.54 (dd, *J* = 7.9, 1.6 Hz, 1H), 7.31 (dtd, *J* = 32.9, 7.9, 1.8 Hz, 2H), 7.05 (dd, *J* = 8.0, 1.4 Hz, 1H), 4.47 (dd, *J* = 16.5, 1.5 Hz, 1H), 4.33 (dd, *J* = 16.5, 1.2 Hz, 1H), 2.45 (dq, *J* = 14.8, 7.4 Hz, 1H), 2.28 (dq, *J* = 14.4, 7.1 Hz, 1H), 2.06 (s, 3H), 1.04 (t, *J* = 7.3 Hz, 3H).; ^13^C NMR (126 MHz, Chloroform-*d*) δ 153.3, 148.6, 130.1, 126.5, 126.5, 124.8, 119.6, 113.3, 112.8, 79.0, 60.5, 31.1, 14.6, 7.9.; HRMS (EI) *m/z* calcd for C_14_H_14_N_2_O_3_S [M]^+^: 290.0725, Found: 290.0688.

**(*S*)-2-methyl-3,10b-dihydrobenzo[e]pyrrolo[1,2-c][1,2,3]oxathiazine-1-carbonitrile 5,5-dioxide (4u)**

White solid (18.1 mg, yield: 69%); mp: 191−193 ℃; purification by silica gel chromatography (ethyl acetate: *n*-hexane= 1:7, R*_f_* = 0.4); 6% *ee*, determined by HPLC analysis [Daicel Chiralpak AD-H, *n*-hexane/*i*-PrOH = 97/3, 1.0 mL/min, λ = 254 nm, t_major_ = 19.61 min, t_minor_ = 21.24 min]; ^1^H NMR (500 MHz, Chloroform-*d*) δ 7.5 (d, *J* = 7.77 Hz, 1H), 7.4 (t, *J* = 8.05 Hz, 1H), 7.3 (t, *J* = 7.55 Hz, 1H), 7.1 (d, *J* = 8.19 Hz, 1H), 5.9 (d, *J* = 4.65 Hz, 1H), 4.5 (dd, *J* = 16.23, 4.77 Hz, 1H), 4.3 (d, *J* = 16.23 Hz, 1H), 2.1 (s, 3H).; ^13^C NMR (126 MHz, Chloroform-*d*) δ 155.1, 149.7, 130.2, 126.5, 126.4, 119.9, 119.7, 113.4, 109.0, 67.9, 59.6, 14.6.; HRMS (EI) *m/z* calcd for C_19_H_14_N_2_O_2_S [M]^+^: 262.0412, Found: 262.0414.

**(*S*)-2-methyl-9b-phenyl-3,9b-dihydrobenzo[d]pyrrolo[1,2-b]isothiazole-1-carbonitrile 5,5-dioxide** **(4v)**

White solid (61.8 mg, yield: 95%); mp: 175−177 ℃; purification by silica gel chromatography (ethyl acetate: *n*-hexane= 1:5, R*_f_* = 0.25); 84% ee, determined by HPLC analysis [Daicel Chiralpak AD-H, *n*-hexane/*i*-PrOH = 90/10, 1.0 mL/min, λ = 254 nm, t_major_ = 11.09 min, t_minor_ = 8.65 min]; ^1^H NMR (300 MHz, Chloroform-*d*) δ 7.85 (dd, *J* = 7.4, 1.2 Hz, 1H), 7.74 – 7.59 (m, 3H), 7.49 – 7.43 (m, 2H), 7.42 – 7.34 (m, 3H), 4.78 (dd, *J* = 18.0, 1.2 Hz, 1H), 4.20 (dd, *J* = 17.9, 1.4 Hz, 1H), 2.15 (s, 3H).; ^13^C NMR (75 MHz, Chloroform-*d*) δ 157.4, 139.7, 139.6, 134.6, 134.1, 130.5, 129.3, 129.2, 126.4, 125.0, 122.0, 114.1, 111.5, 82.3, 77.6, 77.2, 76.7, 56.9, 14.6.; HRMS (EI) *m/z* calcd for C_19_H_14_N_2_O_2_S [M]^+^: 322.0776, Found: 322.0777.

**4-methyl-2,2-diphenyl-1-tosyl-2,5-dihydro-1*H*-pyrrole-3-carbonitrile (4w)**

White solid (38.1 mg, yield: 92%); mp: 214−216 ℃; purification by silica gel chromatography (ethyl acetate: *n*-hexane= 1:7, R*_f_* = 0.43); ^1^H NMR (500 MHz, Chloroform-*d*) δ 7.37 – 7.22 (m, 10H), 6.82 (d, *J* = 8.02 Hz, 2H), 6.60 (d, *J* = 8.29 Hz, 2H), 4.31 (s, 2H), 2.19 (s, 3H), 1.93 (s, 3H).; ^13^C NMR (126 MHz, Chloroform-*d*) δ 150.6, 143.0, 139.0, 136.2, 129.2, 129.1, 128.4, 128.3, 126.9, 116.9, 113.4, 81.9, 58.5, 21.5, 14.5.; HRMS (EI) *m/z* calcd for C_25_H_22_N_2_O_2_S [M]^+^: 414.1402, Found: 414.1405.

**(*S*)-2-(4-chlorophenyl)-4-methyl-1-tosyl-2,5-dihydro-1*H*-pyrrole-3-carbonitrile (4x)**

White solid (0.05 mmol scale, 16.7 mg, yield: 90%); mp: 72−74 ℃; purification by silica gel chromatography (ethyl acetate: *n*-hexane= 1:5, R*_f_* = 0.3); 20% *ee*, determined by HPLC analysis [Daicel Chiralpak AD-H, *n*-hexane/*i*-PrOH = 90/10, 1.0 mL/min, λ = 254 nm, t_major_ = 30.8 min, t_minor_ = 25.8 min]; ^1^H NMR (500 MHz, Chloroform-*d*) δ 7.52 – 7.46 (m, 2H), 7.31 – 7.26 (m, 2H), 7.24 (d, *J* = 7.97 Hz, 2H), 7.20 – 7.15 (m, 2H), 5.50 (dd, *J* = 5.39, 2.75 Hz, 1H), 4.42 – 4.26 (m, 2H), 2.42 (s, 3H), 2.02 (d, *J* = 1.70 Hz, 3H).; ^13^C NMR (126 MHz, Chloroform-*d*) δ 153.0, 144.4, 136.4, 134.9, 134.6, 130.0, 129.1, 128.7, 127.4, 112.9, 110.5, 69.6, 58.6, 21.7, 14.5.; HRMS (ESI) *m/z* calcd for C_19_H_18_ClN_2_O_2_S^+^ [M+H]^+^: 373.0772, Found: 373.0777.

**(3a*R*,3b*S*,12a*S*,13b*R*,15a*R*)-11,15a-dimethyl-1-oxo-12a-phenyl-2,3,3a,3b,4,5,10,12a,13b,14,15,15a-dodecahydro-1*H*-cyclopenta[7,8]phenanthro[3,2-e]pyrrolo[1,2-c][1,2,3]oxathiazine-12-carbonitrile 8,8-dioxide** **(4y)**

White solid (62.2 mg, yield: 64%); mp: 197−199 ℃; purification by silica gel chromatography (ethyl acetate: *n*-hexane= 1:3, R*_f_* = 0.4); 92% ee, determined by HPLC analysis [Daicel Chiralpak IA, *n*-hexane/*i*-PrOH = 93/7, 2.0 mL/min, λ = 254 nm, t_minor_ = 15.98 min, t_major_ = 20.32 min]; ^1^H NMR (500 MHz, Chloroform-*d*) δ 7.43 – 7.37 (m, 3H), 7.35 – 7.28 (m, 3H), 6.89 (d, *J* = 4.2 Hz, 1H), 4.50 (d, *J* = 16.6 Hz, 1H), 4.43 (d, *J* = 16.7 Hz, 1H), 2.95 (tt, *J* = 12.4, 6.3 Hz, 2H), 2.50 (ddd, *J* = 18.9, 8.8, 5.6 Hz, 1H), 2.35 – 2.18 (m, 3H), 2.15 (d, *J* = 4.6 Hz, 3H), 2.13 – 2.03 (m, 2H), 1.90 (td, *J* = 8.8, 8.2, 3.1 Hz, 1H), 1.60 (td, *J* = 13.0, 12.5, 5.8 Hz, 2H), 1.54 – 1.40 (m, 4H), 0.90 (d, *J* = 15.6 Hz, 3H).; ^13^C NMR (126 MHz, Chloroform-*d*) δ 220.5, 220.5, 152.9, 152.9, 147.8, 147.8, 140.1, 140.0, 139.2, 139.1, 138.1, 129.4, 128.7, 128.7, 128.5, 128.4, 125.9, 125.6, 119.4, 119.4, 118.5, 118.3, 115.2, 115.2, 113.6, 113.5, 80.2, 59.9, 59.9, 50.4, 50.4, 48.0, 47.9, 44.3, 44.0, 38.1, 37.9, 35.9, 35.9, 31.5, 31.4, 29.4, 29.2, 26.3, 26.1, 25.7, 25.6, 21.7, 21.7, 14.7, 14.6, 13.9, 13.9.; HRMS (EI) *m/z* calcd for C_30_H_30_N_2_O_4_S [M]^+^: 514.1926, Found: 514.1930.

**7. General Procedure for The Synthesis of 5 or 6**

Under an argon atmosphere, Pd_2_(dba)_3_ (4.6 mg, 0.005 mmol, 2.5 mol%), dppf (6.1 mg, 0.011 mmol, 5.5 mol%), *N*-sulfonyl ketimine **3** (0.2 mmol), and cyano-TMM donor **2** (59.2 mg, 0.3 mmol, 1.5 equiv.) were dissolved in toluene (2.0 mL). The mixture was stirred at 30 ℃ for 2 h. After reaction completed as determined by TLC analysis, NaO*t*Bu (76.9 mg, 0.8 mmol, 4 equiv. or 38.4 mg, 0.4 mmol, 2 equiv.), THF or DMF (2.0 mL) was added and stirred at 30 ℃ or 80 ℃ for 30 min. The reaction was quenched with saturated NH_4_Cl solution, and the mixture was extracted three times with EtOAc. The combined organic layers were washed with brine, dried with anhydrous MgSO_4_, and the solvents evaporated to dryness. The residue was purified by silica gel column chromatography to afford oxathiazonine **5** or 2*H*-pyrrole **6**.

**8. Characterization of The Compounds 5**

**(4Z,6E)-5-methyl-7-phenyl-3*H*-benzo[h][1,2,3]oxathiazonine-6-carbonitrile 2,2-dioxide (5a)**

White solid (48.7 mg, yield: 72%); mp: 207−208 ℃; purification by silica gel chromatography (ethyl acetate: *n*-hexane= 1:5, R*_f_* = 0.5; ^1^H NMR (300 MHz, Acetone-*d*_6_) δ 10.01 (s, 1H), 8.52 – 8.42 (m, 2H), 8.23 (dtd, *J* = 6.6, 4.7, 4.1, 2.1 Hz, 4H), 8.18 – 8.12 (m, 2H), 8.09 (dd, *J* = 7.9, 1.9 Hz, 1H), 6.73 (d, *J* = 1.7 Hz, 1H), 2.51 (d, *J* = 1.6 Hz, 3H); ^13^C NMR (75 MHz, Acetone-*d*_6_) δ 155.4, 149.2, 136.9, 132.3, 131.8, 130.2, 129.9, 129.0, 128.6, 128.5, 127.3, 125.2, 124.1, 116.2, 111.7, 17.7.; HRMS (ESI) *m/z* calcd for C_15_H_19_N_2_O_3_S [M]^+^: 339.0837, Found: 339.0834.

**(4Z,6E)-10-methoxy-5-methyl-7-phenyl-3*H*-benzo[h][1,2,3]oxathiazonine-6-carbonitrile 2,2-dioxide (5b)**

White solid (19.2 mg, yield: 26%); mp: 206−208 ℃; purification by silica gel chromatography (ethyl acetate: *n*-hexane= 1:5, R*_f_* = 0.25); ^1^H NMR (500 MHz, Acetone-*d*_6_) δ 9.16 (s, 1H), 7.71 – 7.64 (m, 2H), 7.45 (dd, *J* = 5.0, 1.9 Hz, 3H), 7.32 (t, *J* = 8.0 Hz, 1H), 7.17 (dd, *J* = 8.4, 1.5 Hz, 1H), 6.85 (dd, *J* = 7.7, 1.5 Hz, 1H), 6.03 (d, *J* = 1.7 Hz, 1H), 3.88 (s, 3H), 1.82 (d, *J* = 1.6 Hz, 3H).; ^13^C NMR (126 MHz, Acetone-*d*_6_) δ 156.3, 154.7, 138.9, 138.0, 134.1, 130.9, 129.4, 129.2, 128.7, 125.6, 120.7, 117.1, 114.4, 112.5, 56.6, 18.3.; HRMS (EI) *m/z* calcd for C_19_H_16_N_2_O_4_S [M]^+^: 368.0831, Found: 368.0833.

**(4Z,6E)-10-methoxy-5-methyl-7-phenyl-3*H*-benzo[h][1,2,3]oxathiazonine-6-carbonitrile 2,2-dioxide (5c)**

White solid (50.8 mg, yield: 69%); mp: 225−226 ℃; purification by silica gel chromatography (ethyl acetate: *n*-hexane= 1:5, R*_f_* = 0.35); ^1^H NMR (300 MHz, Acetone-*d*_6_) δ 9.21 (s, 1H), 7.74 – 7.65 (m, 2H), 7.47 (dt, *J* = 4.6, 2.9 Hz, 3H), 7.24 – 7.17 (m, 1H), 6.95 (d, *J* = 8.1 Hz, 2H), 6.06 – 5.93 (m, 1H), 3.86 (s, 3H), 1.76 (d, *J* = 1.6 Hz, 3H); ^13^C NMR (75 MHz, Acetone-*d*_6_) δ 161.4, 156.3, 150.9, 138.4, 133.5, 131.0, 130.4, 129.5, 129.4, 124.9, 124.8, 117.3, 114.0, 112.8, 111.6, 56.2, 18.6; HRMS (ESI) *m/z* calcd for C_19_H_17_N_2_O_4_S [M+H]^+^: 369.0909, Found: 369.0919.

**(4Z,6E)-5,10-dimethyl-7-phenyl-3*H*-benzo[h][1,2,3]oxathiazonine-6-carbonitrile 2,2-dioxide (5d)**

White solid (57.2 mg, yield: 81%); mp: 224−226 ℃; purification by silica gel chromatography (ethyl acetate: *n*-hexane= 1:5, R*_f_* = 0.35); ^1^H NMR (300 MHz, Acetone-*d*_6_) δ 7.75 – 7.66 (m, 2H), 7.47 (dt, *J* = 4.7, 2.9 Hz, 3H), 7.25 – 7.14 (m, 3H), 6.01 – 5.94 (m, 1H), 2.38 (s, 3H), 1.76 (d, *J* = 1.6 Hz, 3H).; ^13^C NMR (75 MHz, Acetone-*d*_6_) δ 156.5, 150.0, 141.4, 138.1, 133.2, 131.1, 129.9, 129.6, 129.5, 129.4, 128.9, 126.5, 125.1, 117.2, 112.6, 21.0, 18.6.; HRMS (ESI) *m/z* calcd for C_19_H_17_N_2_O_3_S [M+H]^+^: 353.0960, Found: 353.0966.

**(4Z,6E)-9-methoxy-5-methyl-7-phenyl-3*H*-benzo[h][1,2,3]oxathiazonine-6-carbonitrile 2,2-dioxide (5e)**

White solid (47.9 mg, yield: 65%); mp: 221−222 ℃; purification by silica gel chromatography (ethyl acetate: *n*-hexane= 1:5, R*_f_* = 0.35); ^1^H NMR (300 MHz, Acetone-*d*_6_) δ 9.16 (s, 1H), 7.71 (dt, *J* = 5.7, 3.5 Hz, 2H), 7.51 – 7.43 (m, 3H), 7.26 (d, *J* = 1.3 Hz, 2H), 7.12 (s, 1H), 5.97 (q, *J* = 1.7 Hz, 1H), 2.35 (s, 3H), 1.77 (d, *J* = 1.6 Hz, 3H).; ^13^C NMR (75 MHz, Acetone-*d*_6_) δ 156.4, 148.0, 138.3, 137.9, 133.0, 132.4, 131.3, 131.1, 130.0, 129.5, 129.4, 125.8, 125.1, 117.1, 112.5, 20.8, 18.6.; HRMS (ESI) *m/z* calcd for C_19_H_17_N_2_O_4_S [M+H]^+^: 369.09090, Found: 369.0919.

**(4Z,6E)-9-(tert-butyl)-5-methyl-7-phenyl-3H-benzo[h][1,2,3]oxathiazonine-6-carbonitrile 2,2-dioxide (5f)**

White solid (51.2 mg, yield: 67%); mp: 207−208 ℃; purification by silica gel chromatography (ethyl acetate: *n*-hexane= 1:5, R*_f_* = 0.35); ^1^H NMR (300 MHz, Acetone-*d*_6_) δ 9.17 (s, 1H), 7.78 – 7.70 (m, 2H), 7.53 – 7.44 (m, 4H), 7.39 (d, *J* = 2.5 Hz, 1H), 7.30 (d, *J* = 8.6 Hz, 1H), 5.96 (q, *J* = 1.6 Hz, 1H), 1.76 (d, *J* = 1.6 Hz, 3H), 1.34 (s, 9H).; ^13^C NMR (75 MHz, Acetone-*d*_6_) δ 156.7, 151.2, 147.8, 137.8, 133.1, 132.2, 131.1, 129.5, 129.4, 127.4, 127.0, 125.4, 125.0, 117.2, 112.3, 35.3, 31.4, 18.5.; HRMS (ESI) *m/z* calcd for C_22_H_23_FN_2_O_3_S [M+H]^+^: 395.1429, Found: 395.1430.

**(4Z,6E)-9-fluoro-5-methyl-7-phenyl-3*H*-benzo[h][1,2,3]oxathiazonine-6-carbonitrile 2,2-dioxide (5g)**

White solid (48.4 mg, yield: 68%); mp: 211−213 ℃; purification by silica gel chromatography (ethyl acetate: *n*-hexane= 1:5, R*_f_* = 0.45); ^1^H NMR (300 MHz, Acetone-*d*_6_) δ 9.25 (s, 1H), 7.74 (dd, *J* = 6.8, 3.0 Hz, 2H), 7.54 – 7.41 (m, 4H), 7.32 – 7.22 (m, 2H), 6.03 (d, *J* = 1.6 Hz, 1H), 1.81 (d, *J* = 1.6 Hz, 3H).; ^13^C NMR (75 MHz, Acetone-*d*_6_) δ 161.28 (d, *J* = 247.3 Hz)., 154.7, 146.30 (d, *J* = 3.0 Hz)., 137.2, 134.5, 134.3, 133.1, 131.2, 129.6, 129.3 161.2 (d, *J* = 247.3 Hz), 146.3 (d, *J* = 3.0 Hz), 128.09 (d, *J* = 9.4 Hz), 125.1, 117.43 (d, *J* = 23.5 Hz), 116.8, 116.4 (d, *J* = 24.8 Hz), 113.1, 18.4.; ^19^F NMR (376 MHz, Acetone-*d*_6_) δ -115.3.; HRMS (ESI) *m/z* calcd for C_18_H_14_FN_2_O_3_S [M+H]^+^: 357.0709, Found: 357.0708.

**(4Z,6E)-9-chloro-5-methyl-7-phenyl-3*H*-benzo[h][1,2,3]oxathiazonine-6-carbonitrile 2,2-dioxide (5h)**

White solid (47.6 mg, yield: 64%); mp: 231−233 ℃; purification by silica gel chromatography (ethyl acetate: *n*-hexane= 1:5, R*_f_* = 0.35); ^1^H NMR (300 MHz, Acetone-*d*_6_) δ 9.33 (s, 1H), 7.77 – 7.69 (m, 2H), 7.55 – 7.39 (m, 6H), 6.03 (q, *J* = 1.6 Hz, 1H), 1.80 (d, *J* = 1.6 Hz, 3H).; ^13^C NMR (75 MHz, Acetone-*d*_6_) δ 154.6, 148.9, 137.3, 134.4, 133.3, 133.1, 131.3, 130.8, 129.6, 129.4, 129.3, 127.9, 125.0, 116.8, 113.2, 18.5.; HRMS (ESI) *m/z* calcd for C_18_H_14_ClN_2_O_3_S [M+H]^+^: 373.0414, Found: 373.0414.

**(4Z,6E)-9-bromo-5-methyl-7-phenyl-3*H*-benzo[h][1,2,3]oxathiazonine-6-carbonitrile 2,2-dioxide (5i)**

White solid (45.7 mg, yield: 55%); mp: 206−209 ℃; purification by silica gel chromatography (ethyl acetate: *n*-hexane= 1:5, R*_f_* = 0.35); ^1^H NMR (300 MHz, Acetone-*d*_6_) δ 9.33 (s, 1H), 7.77 – 7.69 (m, 2H), 7.67 (dd, *J* = 8.7, 2.5 Hz, 1H), 7.61 (d, *J* = 2.5 Hz, 1H), 7.50 (dt, *J* = 4.7, 3.0 Hz, 3H), 7.37 (d, *J* = 8.7 Hz, 1H), 6.03 (q, *J* = 1.6 Hz, 1H), 1.80 (d, *J* = 1.6 Hz, 3H).; ^13^C NMR (75 MHz, Acetone-*d*_6_) δ 154.5, 149.4, 137.3, 134.8, 133.9, 133.3, 132.3, 131.3, 129.6, 129.4, 128.1, 125.1, 120.8, 116.8, 113.2, 18.5.; HRMS (ESI) *m/z* calcd for C_18_H_14_BrN_2_O_3_S [M+H]^+^: 416.9909, Found: 416.9910.

**(4Z,6E)-7-(4-methoxyphenyl)-5-methyl-3*H*-benzo[h][1,2,3]oxathiazonine-6-carbonitrile 2,2-dioxide (5j)**

White solid (50.8 mg, yield: 69%); mp: 224−225 ℃; purification by silica gel chromatography (ethyl acetate: *n*-hexane= 1:5, R*_f_* = 0.55); ^1^H NMR (300 MHz, Acetone-*d*_6_) δ 9.18 (s, 1H), 7.70 – 7.63 (m, 2H), 7.52 – 7.45 (m, 1H), 7.43 – 7.36 (m, 2H), 7.33 – 7.27 (m, 1H), 7.05 – 6.98 (m, 2H), 5.95 (q, *J* = 1.6 Hz, 1H), 3.85 (s, 3H), 1.73 (d, *J* = 1.6 Hz, 3H).; ^13^C NMR (75 MHz, Acetone-*d*_6_) δ 162.4, 155.7, 150.2, 133.4, 133.0, 131.3, 130.7, 130.1, 129.8, 128.1, 126.1, 124.9, 117.6, 114.8, 109.9, 55.9, 18.6.; HRMS (ESI) *m/z* calcd for C_19_H_17_N_2_O_4_S [M+H]^+^: 369.0909, Found: 369.0920.

**(4Z,6E)-5-methyl-7-(p-tolyl)-3*H*-benzo[h][1,2,3]oxathiazonine-6-carbonitrile 2,2-dioxide (5k)**

White solid (50.0 mg, yield: 71%); mp: 228−229 ℃; purification by silica gel chromatography (ethyl acetate: *n*-hexane= 1:5, R*_f_* = 0.55); ^1^H NMR (300 MHz, Acetone-*d*_6_) δ 9.21 (s, 1H), 7.65 – 7.58 (m, 2H), 7.51 – 7.44 (m, 1H), 7.39 (ddt, *J* = 6.9, 3.6, 1.6 Hz, 2H), 7.33 – 7.25 (m, 3H), 5.96 (q, *J* = 1.6 Hz, 1H), 2.36 (s, 3H), 1.74 (d, *J* = 1.6 Hz, 3H).; 13C NMR (75 MHz, Acetone-*d*_6_) δ 156.2, 150.1, 141.6, 134.9, 133.3, 132.9, 130.7, 130.1, 129.9, 129.4, 128.1, 126.1, 124.9, 117.3, 111.5, 21.3, 18.5.; HRMS (ESI) *m/z* calcd for C_19_H_17_N_2_O_3_S [M+H]^+^: 353.0960, Found: 353.0962.

**(4Z,6E)-7-(4-cyanophenyl)-5-methyl-3*H*-benzo[h][1,2,3]oxathiazonine-6-carbonitrile 2,2-dioxide (5l)**

White solid (47.9 mg, yield: 66%); mp: 235−236 ℃; purification by silica gel chromatography (ethyl acetate: *n*-hexane= 1:5, R*_f_* = 0.4; ^1^H NMR (300 MHz, Acetone-*d*_6_) δ 9.29 (s, 1H), 7.93 (s, 4H), 7.55 – 7.48 (m, 1H), 7.46 – 7.39 (m, 3H), 6.01 (q, *J* = 1.6 Hz, 1H), 1.77 (d, *J* = 1.6 Hz, 3H).; ^13^C NMR (75 MHz, Acetone-*d*_6_) δ 154.5, 150.1, 142.1, 133.4, 132.6, 131.7, 131.3, 130.1, 129.8, 128.4, 126.3, 125.3, 118.8, 116.5, 115.2, 114.4, 18.3.; HRMS (ESI) *m/z* calcd for C_19_H_14_N_3_O_3_S [M+H]^+^: 364.0756, Found: 364.0760.

**(4Z,6E)-5-methyl-7-(4-(trifluoromethyl)phenyl)-3*H*-benzo[h][1,2,3]oxathiazo nine-6-carbonitrile 2,2-dioxide (5m)**

White solid (52.1 mg, yield: 64%); mp: 220−222 ℃; purification by silica gel chromatography (ethyl acetate: *n*-hexane= 1:5, R*_f_* = 0.4; ^1^H NMR (300 MHz, Acetone-*d*_6_) δ 9.30 (s, 1H), 8.01 – 7.93 (m, 2H), 7.86 (d, *J* = 8.5 Hz, 2H), 7.55 – 7.48 (m, 1H), 7.45 – 7.39 (m, 3H), 6.02 (q, *J* = 1.6 Hz, 1H), 1.78 (d, *J* = 1.6 Hz, 3H).; ^13^C NMR (75 MHz, Acetone-*d*_6_) δ 154.8, 150.1, 141.8, 132.7, 132.2, 132.0, 131.8, 131.2, 130.1, 129.8, 128.4, 126.8, 126.54 (q, *J* = 3.8 Hz)., 126.3, 125.2, 123.2, 116.6, 114.9, 18.4.; ^19^F NMR (376 MHz, Acetone-*d*_6_) δ -63.4.; HRMS (ESI) *m/z* calcd for C_19_H_13_F_3_N_3_O_3_S [M+H]^+^: 407.0677, Found: 407.0674.

**(4Z,6E)-7-(4-bromophenyl)-5-methyl-3*H*-benzo[h][1,2,3]oxathiazonine-6-carbonitrile 2,2-dioxide (5n)**

White solid (57.4 mg, yield: 69%); mp: 201−203 ℃; purification by silica gel chromatography (ethyl acetate: *n*-hexane= 1:5, R*_f_* = 0.45; ^1^H NMR (300 MHz, Acetone-*d*_6_) δ 9.25 (s, 1H), 7.72 – 7.63 (m, 4H), 7.53 – 7.46 (m, 1H), 7.44 – 7.34 (m, 3H), 5.98 (q, *J* = 1.6 Hz, 1H), 1.75 (d, *J* = 1.6 Hz, 3H).; ^13^C NMR (75 MHz, Acetone-*d*_6_) δ 155.0, 150.1, 137.0, 132.9, 132.7, 132.2, 131.3, 131.0, 129.9, 128.3, 126.2, 125.1, 125.0, 116.9, 113.3, 18.4.HRMS (ESI) *m/z* calcd for C_18_H_14_BrN_2_O_3_S [M+H]^+^: 416.9909, Found: 416.9906.

**(4Z,6E)-5,7-dimethyl-3*H*-benzo[h][1,2,3]oxathiazonine-6-carbonitrile 2,2-dioxide (5o)**

White solid (36.5 mg, yield: 66%); mp: 214−216 ℃; purification by silica gel chromatography (ethyl acetate: *n*-hexane= 1:5, R*_f_* = 0.49; ^1^H NMR (500 MHz, Acetone-*d*_6_) δ 9.03 (s, 1H), 7.47 – 7.40 (m, 1H), 7.38 – 7.31 (m, 2H), 7.23 (dd, *J* = 7.6, 1.7 Hz, 1H), 5.88 (s, 1H), 2.44 (s, 3H), 1.62 (d, *J* = 1.6 Hz, 3H).; ^13^C NMR (126 MHz, Acetone-*d*_6_) δ 156.0, 149.7, 133.4, 132.0, 130.5, 128.7, 128.2, 125.8, 125.0, 116.3, 113.1, 25.3, 18.6.; HRMS (EI) *m/z* calcd for C_13_H_12_N_2_O_3_S [M]^+^: 276.0569, Found: 276.0572.

**(3Z,5E)-4-methyl-6-phenyl-2*H*-benzo[g][1,2]thiazocine-5-carbonitrile 1,1-dioxide (5p)**

Orange solid (19.0 mg, yield: 59%); mp: 159−161 ℃; purification by silica gel chromatography (ethyl acetate: *n*-hexane= 1:5, R*_f_* = 0.13; ^1^H NMR (500 MHz, Chloroform-*d*) δ 8.05 (dd, *J* = 6.0, 3.3 Hz, 1H), 7.56 (dd, *J* = 5.8, 3.3 Hz, 2H), 7.51 – 7.47 (m, 2H), 7.39 (dd, *J* = 5.2, 1.9 Hz, 3H), 7.04 (dd, *J* = 5.7, 3.3 Hz, 1H), 6.50 (d, *J* = 8.0 Hz, 1H), 5.74 (d, *J* = 6.2 Hz, 1H), 1.86 (d, *J* = 1.4 Hz, 3H).; ^13^C NMR (126 MHz, Chloroform-*d*) δ 156.2, 138.9, 137.8, 134.8, 133.4, 130.1, 130.1, 129.8, 128.7, 127.9, 123.3, 117.0, 110.7, 29.8, 21.3.^;^ HRMS (EI) *m/z* calcd for C_18_H_14_N_2_O_2_S [M]^+^: 322.0776, Found: 322.0777.

**(3a*S*,3b*R*,10Z,12E,14b*S*,16a*S*)-11,16a-dimethyl-1-oxo-13-phenyl-1,2,3,3a,3b,4,5,9,14b,15,16,16a-dodecahydrocyclopenta[7,8]phenanthro[3,2-h][1,2,3]oxathiazonine-12-carbonitrile 8,8-dioxide (5q)**

White solid (48.6 mg, yield: 50%); mp: 198−200 ℃; purification by silica gel chromatography (diethyl ether: *n*-hexane= 1:1, R*_f_* = 0.45); ^1^H NMR (500 MHz, Chloroform-*d*) δ 7.65 – 7.54 (m, 2H), 7.38 (d, *J* = 6.1 Hz, 3H), 7.13 (s, 1H), 6.98 (s, 0H), 6.80 (s, 1H), 5.89 (d, *J* = 7.4 Hz, 1H), 3.05 – 2.83 (m, 2H), 2.50 (dd, *J* = 19.4, 8.7 Hz, 1H), 2.23 (q, *J* = 13.5, 11.2 Hz, 2H), 2.13 (dd, *J* = 19.0, 9.2 Hz, 1H), 2.03 (q, *J* = 10.2, 8.5 Hz, 2H), 1.94 – 1.87 (m, 1H), 1.71 (d, *J* = 13.3 Hz, 3H), 1.67 – 1.35 (m, 7H), 0.88 (d, *J* = 19.5 Hz, 3H).; ^13^C NMR (126 MHz, Chloroform-*d*) δ 221.1, 157.3, 157.1, 147.1, 147.0, 139.4, 139.3, 139.3, 139.2, 136.5, 136.4, 132.8, 132.8, 130.7, 130.6, 128.9, 128.6, 128.5, 128.4, 128.3, 125.6, 125.1, 124.9, 124.9, 123.9, 123.8, 116.6, 110.0, 109.8, 50.4, 50.3, 48.0, 48.0, 44.2, 44.0, 37.8, 35.9, 31.5, 31.4, 29.8, 29.3, 29.2, 26.1, 26.0, 25.7, 21.6, 19.3, 19.1, 14.0, 13.9.; HRMS (EI) *m/z* calcd for C_30_H_30_N_2_O_4_S [M]^+^: 514.1926, Found: 514.1924.

**9. Characterization of The Compounds 6**

**2-(2-hydroxyphenyl)-4-methyl-2-phenyl-2*H*-pyrrole-3-carbonitrile (6a)**

White solid (38.9 mg, yield: 73%); mp: 108−110 ℃; purification by silica gel chromatography (ethyl acetate: *n*-hexane= 1:5, R*_f_* = 0.4; ^1^H NMR (300 MHz, Chloroform-*d*) δ 9.28 (s, 1H), 8.14 (s, 1H), 7.56 (dd, *J* = 8.1, 1.6 Hz, 1H), 7.22 (dd, *J* = 6.3, 2.7 Hz, 4H), 7.06 – 6.96 (m, 2H), 6.92 – 6.83 (m, 2H), 2.25 (s, 3H).; ^13^C NMR (75 MHz, Chloroform-*d*) δ 165.1, 156.1, 150.5, 135.9, 134.9, 130.5, 129.1, 128.8, 127.7, 125.8, 123.7, 120.0, 118.5, 114.7, 92.3, 12.6.; HRMS (ESI) *m/z* calcd for C_18_H_15_N_2_O [M+H]^+^: 275.1184, Found: 275.1195.

**2-(2-hydroxy-3-methoxyphenyl)-4-methyl-2-phenyl-2*H*-pyrrole-3-carbonitrile (6b)**

White solid (24.3 mg, yield: 40%); mp: 119−120 ℃; purification by silica gel chromatography (ethyl acetate: *n*-hexane= 1:5, R*_f_* = 0.4; ^1^H NMR (300 MHz, Chloroform-*d*) δ 8.65 (s, 1H), 8.23 (s, 1H), 7.30 – 7.25 (m, 3H), 7.18 – 7.11 (m, 3H), 6.95 – 6.81 (m, 2H), 3.85 (s, 3H), 2.32 (s, 3H).; ^13^C NMR (75 MHz, Chloroform-*d*) δ 165.1, 150.3, 149.2, 145.7, 136.0, 135.7, 129.0, 128.6, 126.1, 123.9, 120.1, 119.6, 114.8, 112.2, 92.3, 56.2, 12.6.; HRMS (ESI) *m/z* calcd for C_18_H_15_N_2_O [M]^+^: 304.1212, Found: 304.1212.

**2-(3-fluoro-2-hydroxyphenyl)-4-methyl-2-phenyl-2*H*-pyrrole-3-carbonitrile (6c)**

White solid (29.8 mg, yield: 51%); mp: 131−132 ℃; purification by silica gel chromatography (ethyl acetate: *n*-hexane= 1:5, R*_f_* = 0.35; ^1^H NMR (300 MHz, Chloroform-*d*) δ 9.44 (s, 1H), 8.27 (s, 1H), 7.40 (dd, *J* = 7.9, 1.4 Hz, 1H), 7.33 – 7.27 (m, 3H), 7.16 – 7.05 (m, 3H), 6.88 (td, *J* = 8.1, 5.0 Hz, 1H), 2.35 (s, 3H).; ^13^C NMR (75 MHz, Chloroform-*d*) δ 165.5, 152.9 (d, *J* = 245.0 Hz), 150.6, 144.8 (d, *J* = 12.6 Hz), 135.8, 134.6, 129.2, 129.0, 125.9 (d, *J* = 2.2 Hz), 125.8, 122.9 (d, *J* = 3.6 Hz), 119.6 (d, *J* = 7.2 Hz), 117.1 (d, *J* = 18.5 Hz), 114.6, 92.2, 12.7.; ^19^F NMR (376 MHz, Chloroform-*d*) δ -134.5.; HRMS (ESI) *m/z* calcd for C_18_H_13_FN_2_O [M]^+^: 292.1012, Found: 292.1012.

**2-(2-hydroxy-4-methoxyphenyl)-4-methyl-2-phenyl-2*H*-pyrrole-3-carbonitrile (6d)**

White solid (29.8 mg, yield: 51%); mp: 153−154 ℃; purification by silica gel chromatography (ethyl acetate: *n*-hexane= 1:5, R*_f_* = 0.4; ^1^H NMR (300 MHz, Chloroform-*d*) δ 9.39 (s, 1H), 8.22 (s, 1H), 7.51 (d, *J* = 9.3 Hz, 1H), 7.32 – 7.27 (m, 3H), 7.08 – 7.04 (m, 2H), 6.54 – 6.48 (m, 2H), 3.78 (s, 3H), 2.34 (s, 3H).; ^13^C NMR (75 MHz, Chloroform-*d*) δ 164.8, 161.5, 157.6, 150.3, 136.2, 135.3, 129.1, 128.8, 128.4, 125.8, 116.1, 114.7, 106.1, 103.7, 92.1, 55.4, 12.6.; HRMS (ESI) *m/z* calcd for C_19_H_17_N_2_O_2_ [M+H]^+^: 305.1290, Found: 305.1299.

**2-(2-hydroxy-4-methylphenyl)-4-methyl-2-phenyl-2*H*-pyrrole-3-carbonitrile (6e)**

White solid (38.6 mg, yield: 67%); mp: 136−139 ℃; purification by silica gel chromatography (ethyl acetate: *n*-hexane= 1:5, R*_f_* = 0.35; ^1^H NMR (300 MHz, Chloroform-*d*) δ 9.21 (s, 1H), 8.22 (s, 1H), 7.48 (d, *J* = 7.7 Hz, 1H), 7.31 – 7.26 (m, 3H), 7.10 – 7.05 (m, 2H), 6.79 – 6.73 (m, 2H), 2.34 (s, 3H), 2.31 (s, 3H).; ^13^C NMR (75 MHz, Chloroform-*d*) δ 164.9, 156.0, 150.3, 140.9, 136.2, 135.2, 129.1, 128.8, 127.6, 125.9, 120.9, 120.8, 119.1, 114.7, 92.3, 21.2, 12.6.; HRMS (ESI) *m/z* calcd for C_19_H_17_N_2_O [M+H]^+^: 289.1341, Found: 289.1353.

**2-(2-hydroxy-5-methylphenyl)-4-methyl-2-phenyl-2*H*-pyrrole-3-carbonitrile (6f)**

White solid (40.9 mg, yield: 71%); mp: 169−170 ℃; purification by silica gel chromatography (ethyl acetate: *n*-hexane= 1:4, R*_f_* = 0.4; ^1^H NMR (300 MHz, Chloroform-*d*) δ 9.08 (s, 1H), 8.22 (s, 1H), 7.40 (d, *J* = 2.1 Hz, 1H), 7.33 – 7.27 (m, 3H), 7.14 – 7.06 (m, 3H), 6.86 (d, *J* = 8.2 Hz, 1H), 2.34 (d, *J* = 1.4 Hz, 6H).; ^13^C NMR (75 MHz, Chloroform-*d*) δ 165.0, 153.8, 150.4, 136.0, 135.0, 131.0, 129.2, 129.1, 128.8, 128.1, 125.8, 123.4, 118.3, 114.7, 92.4, 20.7, 12.6.; HRMS (ESI) *m/z* calcd for C_19_H_17_N_2_O [M+H]^+^: 289.1341, Found: 289.1353.

**2-(5-(tert-butyl)-2-hydroxyphenyl)-4-methyl-2-phenyl-2*H*-pyrrole-3-carbonitrile (6g)**

White solid (40.2 mg, yield: 61%); mp: 148−150 ℃; purification by silica gel chromatography (ethyl acetate: *n*-hexane= 1:3, R*_f_* = 0.25; ^1^H NMR (300 MHz, Chloroform-*d*) δ 9.25 (s, 1H), 8.22 (s, 1H), 7.71 (d, *J* = 2.3 Hz, 1H), 7.37 – 7.28 (m, 4H), 7.14 – 7.05 (m, 2H), 6.91 (d, *J* = 8.5 Hz, 1H), 2.35 (s, 3H), 1.37 (s, 9H).; ^13^C NMR (75 MHz, Chloroform-*d*) δ 165.0, 153.6, 150.4, 142.8, 136.0, 135.2, 129.1, 128.7, 127.4, 125.8, 125.0, 122.7, 118.0, 114.8, 92.8, 34.6, 31.6, 12.6.; HRMS (ESI) *m/z* calcd for C_22_H_23_N_2_O [M+H]^+^: 331.1810, Found: 331.1812

**2-(5-fluoro-2-hydroxyphenyl)-4-methyl-2-phenyl-2*H*-pyrrole-3-carbonitrile (6h)**

White solid (45.0 mg, yield: 77%); mp: 127−129 ℃; purification by silica gel chromatography (ethyl acetate: *n*-hexane= 1:4, R*_f_* = 0.4; ^1^H NMR (300 MHz, Chloroform-*d*) δ 9.08 (s, 1H), 8.26 (s, 1H), 7.37 – 7.29 (m, 4H), 7.08 (dd, *J* = 6.5, 3.0 Hz, 2H), 7.04 – 6.96 (m, 1H), 6.88 (dd, *J* = 9.0, 5.0 Hz, 1H), 2.35 (s, 3H).; ^13^C NMR (75 MHz, Chloroform-*d*) δ 165.6, 156.2 (d, *J* = 238.6 Hz), 152.3, 152.3, 150.7, 135.5, 134.4, 129.2, 129.0, 125.7, 124.21 (d, *J* = 7.0 Hz), 119.26 (d, *J* = 7.9 Hz), 116.88 (d, *J* = 22.4 Hz), 114.40 (d, *J* = 25.2 Hz), 114.2, 91.8, 12.6.; ^19^F NMR (376 MHz, Chloroform-*d*) δ -123.7.; HRMS (ESI) *m/z* calcd for C_18_H_14_FN_2_O [M+H]^+^: 293.1090, Found: 293.1104.

**2-(5-chloro-2-hydroxyphenyl)-4-methyl-2-phenyl-2*H*-pyrrole-3-carbonitrile (6i)**

White solid (41.3 mg, yield: 67%); mp: 99−101 ℃; purification by silica gel chromatography (ethyl acetate: *n*-hexane= 1:3, R*_f_* = 0.4; ^1^H NMR (300 MHz, Chloroform-*d*) δ 9.27 (s, 1H), 8.26 (s, 1H), 7.56 (d, *J* = 2.5 Hz, 1H), 7.33 – 7.29 (m, 3H), 7.25 (dd, *J* = 8.7, 2.4 Hz, 1H), 7.11 – 7.05 (m, 2H), 6.87 (d, *J* = 8.7 Hz, 1H), 2.35 (s, 3H).; ^13^C NMR (75 MHz, Chloroform-*d*) δ 165.6, 155.0, 150.7, 135.6, 134.3, 130.4, 129.3, 129.1, 127.3, 125.8, 125.1, 124.8, 119.8, 114.3, 91.7, 12.7.; HRMS (ESI) *m/z* calcd for C_18_H_14_ClN_2_O [M+H]^+^: 309.0795, Found: 307.0793.

**2-(5-bromo-2-hydroxyphenyl)-4-methyl-2-phenyl-2*H*-pyrrole-3-carbonitrile (6j)**

White solid (43.6 mg, yield: 62%); mp: 111−112 ℃; purification by silica gel chromatography (ethyl acetate: *n*-hexane= 1:3, R*_f_* = 0.4; ^1^H NMR (300 MHz, Chloroform-*d*) δ 9.29 (s, 1H), 8.25 (s, 1H), 7.69 (d, *J* = 2.4 Hz, 1H), 7.39 (dd, *J* = 8.7, 2.4 Hz, 1H), 7.33 – 7.28 (m, 3H), 7.07 (dd, *J* = 6.6, 3.0 Hz, 2H), 6.82 (d, *J* = 8.7 Hz, 1H), 2.35 (s, 3H).; ^13^C NMR (75 MHz, Chloroform-*d*) δ 165.6, 155.5, 150.8, 135.5, 134.3, 133.4, 130.1, 129.3, 129.1, 125.8, 125.7, 120.3, 114.3, 111.9, 91.7, 12.7.; HRMS (ESI) *m/z* calcd for C_18_H_14_BrN_2_O [M+H]^+^: 353.0290, Found: 353.0288.

**2-(2-hydroxyphenyl)-2,4-dimethyl-2*H*-pyrrole-3-carbonitrile (6k)**

White solid (22.9 mg, yield: 54%); mp: 99−101 ℃; purification by silica gel chromatography (ethyl acetate: *n*-hexane= 1:5, R*_f_* = 0.38; ^1^H NMR (500 MHz, Chloroform-*d*) δ 9.86 (s, 1H), 8.04 (s, 1H), 7.54 (dd, *J* = 7.8, 1.6 Hz, 1H), 7.21 (ddd, *J* = 8.5, 7.4, 1.6 Hz, 1H), 6.93 (dd, *J* = 8.2, 1.3 Hz, 1H), 6.86 (td, *J* = 7.6, 1.3 Hz, 1H), 2.33 (s, 3H), 1.74 (s, 3H).; ^13^C NMR (126 MHz, Chloroform-*d*) δ 163.4, 155.3, 150.4, 135.4, 129.9, 126.5, 124.1, 120.0, 118.4, 114.9, 87.9, 25.1, 12.6.; HRMS (EI) *m/z* calcd for C_13_H_12_N_2_O [M]^+^: 212.0950, Found: 212.0949.

**2-(2-hydroxyphenyl)-2-(4-methoxyphenyl)-4-methyl-2*H*-pyrrole-3-carbonitrile (6l)**

White solid (32.8 mg, yield: 54%); mp: 124−125 ℃; purification by silica gel chromatography (ethyl acetate: *n*-hexane= 1:3, R*_f_* = 0.4; ^1^H NMR (300 MHz, Chloroform-*d*) δ 9.31 (s, 1H), 8.20 (s, 1H), 7.59 (dd, *J* = 8.2, 1.4 Hz, 1H), 7.32 – 7.26 (m, 1H), 6.99 – 6.92 (m, 4H), 6.80 (d, *J* = 8.9 Hz, 2H), 3.75 (s, 3H), 2.34 (s, 3H).; ^13^C NMR (75 MHz, Chloroform-*d*) δ 164.8, 159.9, 156.2, 150.1, 136.4, 130.5, 127.7, 127.2, 126.6, 123.9, 120.0, 118.6, 114.8, 114.6, 92.2, 55.4, 12.6.; HRMS (ESI) *m/z* calcd for C_19_H_17_N_2_O_2_ [M+H]^+^: 305.1290, Found: 305.1293.

**2-(2-hydroxyphenyl)-4-methyl-2-(p-tolyl)-2*H*-pyrrole-3-carbonitrile (6m)**

White solid (38.0 mg, yield: 66%); mp: 141−143 ℃; purification by silica gel chromatography (ethyl acetate: *n*-hexane= 1:5, R*_f_* = 0.4; ^1^H NMR (300 MHz, Chloroform-*d*) δ 9.37 (s, 1H), 8.29 (s, 1H), 7.67 (dd, *J* = 8.1, 1.6 Hz, 1H), 7.39 – 7.33 (m, 1H), 7.16 (d, *J* = 8.2 Hz, 2H), 7.05 – 6.98 (m, 4H), 2.40 (s, 3H), 2.37 (s, 3H).; ^13^C NMR (75 MHz, Chloroform-*d*) δ 164.9, 156.2, 150.2, 138.7, 136.2, 131.9, 130.5, 129.9, 127.7, 125.7, 123.9, 120.0, 118.6, 114.8, 92.3, 21.2, 12.6.; HRMS (ESI) *m/z* calcd for C_19_H_16_N_2_O [M]^+^: 288.1263, Found: 288.1262.

**2-(4-cyanophenyl)-2-(2-hydroxyphenyl)-4-methyl-2*H*-pyrrole-3-carbonitrile (6n)**

White solid (36.5 mg, yield: 61%); mp: 174−175 ℃; purification by silica gel chromatography (ethyl acetate: *n*-hexane= 1:5, R*_f_* = 0.4; ^1^H NMR (300 MHz, Chloroform-*d*) δ 9.02 (s, 1H), 8.31 (s, 1H), 7.60 – 7.54 (m, 3H), 7.32 (td, *J* = 7.7, 1.6 Hz, 1H), 7.20 (d, *J* = 8.5 Hz, 2H), 7.00 – 6.91 (m, 2H), 2.37 (s, 3H).; ^13^C NMR (75 MHz, Chloroform-*d*) δ 166.1, 156.1, 151.4, 140.6, 135.1, 132.9, 131.2, 127.5, 126.7, 122.9, 120.4, 119.0, 118.3, 114.2, 112.8, 91.6, 12.8.; HRMS (ESI) *m/z* calcd for C_19_H_13_N_3_O [M+H]^+^: 300.1137, Found: 300.1139.

**2-(2-hydroxyphenyl)-4-methyl-2-(4-(trifluoromethyl)phenyl)-2*H*-pyrrole-3-carbonitrile (6o)**

White solid (47.9 mg, yield: 70%); mp: 126−127 ℃; purification by silica gel chromatography (ethyl acetate: *n*-hexane= 1:5, R*_f_* = 0.4; ^1^H NMR (300 MHz, Chloroform-*d*) δ 9.14 (s, 1H), 8.30 (s, 1H), 7.58 (dd, *J* = 13.6, 7.7 Hz, 3H), 7.32 (td, *J* = 7.7, 1.6 Hz, 1H), 7.21 (d, *J* = 8.2 Hz, 2H), 6.97 (t, *J* = 7.6 Hz, 2H), 2.37 (s, 3H).; ^13^C NMR (75 MHz, Chloroform-*d*) δ 165.8, 156.1, 151.1, 139.3, 135.5, 131.2, 131.0, 130.8, 127.6, 126.3, 126.2 (q, *J* = 3.7 Hz)., 123.3, 120.3, 118.9, 114.3, 91.8, 12.7.; ^19^F NMR (376 MHz, Chloroform-*d*) δ -62.8.; HRMS (ESI) *m/z* calcd for C_19_H_13_F_3_N_2_O [M]^+^: 342.0980, Found: 342.0984.

**2-(4-bromophenyl)-2-(2-hydroxyphenyl)-4-methyl-2*H*-pyrrole-3-carbonitrile (6p)**

White solid (47.2 mg, yield: 67%); mp: 126−128 ℃; purification by silica gel chromatography (ethyl acetate: *n*-hexane= 1:5, R*_f_* = 0.4; ^1^H NMR (300 MHz, Chloroform-*d*) δ 9.16 (s, 1H), 8.25 (s, 1H), 7.57 (dd, *J* = 8.1, 1.6 Hz, 1H), 7.44 – 7.38 (m, 2H), 7.34 – 7.27 (m, 1H), 6.98 – 6.91 (m, 4H), 2.35 (s, 3H).; ^13^C NMR (75 MHz, Chloroform-*d*) δ 165.3, 156.0, 150.6, 135.6, 134.0, 132.2, 130.7, 127.5, 127.4, 123.2, 123.0, 120.1, 118.7, 114.3, 91.7, 12.6.; HRMS (ESI) *m/z* calcd for C_18_H_14_BrN_2_O [M+H]^+^: 353.0290, Found: 353.0294.

**2-((8R,9S,13S,14S)-3-hydroxy-13-methyl-17-oxo-7,8,9,11,12,13,14,15,16,17-decahydro-6*H*-cyclopenta[a]phenanthren-2-yl)-4-methyl-2-phenyl-2*H*-pyrrole-3-carbonitrile (6q)**

White solid (25.5 mg, yield: 23%); mp: 177−179 ℃; purification by silica gel chromatography (Acetone: *n*-hexane= 1:5, R*_f_* = 0.3); ^1^H NMR (500 MHz, Chloroform-*d*) δ 9.13 (d, *J* = 63.5 Hz, 1H), 8.22 (d, *J* = 1.6 Hz, 1H), 7.55 (dd, *J* = 9.1, 1.0 Hz, 1H), 7.29 (td, *J* = 4.5, 2.4 Hz, 3H), 7.12 – 7.06 (m, 2H), 6.69 (d, *J* = 9.6 Hz, 1H), 2.99 – 2.74 (m, 2H), 2.56 – 2.42 (m, 2H), 2.35 (d, *J* = 8.5 Hz, 3H), 2.20 – 1.93 (m, 4H), 1.71 – 1.37 (m, 7H), 0.92 (d, *J* = 38.5 Hz, 3H).; ^13^C ^13^C NMR (126 MHz, Chloroform-*d*) δ 221.0, 164.8, 153.8, 150.5, 150.2, 139.2, 136.2, 136.1, 135.2, 131.6, 131.4, 129.1, 128.8, 125.9, 125.8, 125.1, 125.0, 121.3, 121.1, 118.4, 118.3, 114.9, 92.7, 92.6, 50.5, 48.2, 48.1, 44.4, 44.2, 38.5, 38.4, 36.0, 31.7, 29.8, 29.4, 29.2, 26.6, 26.0, 21.7, 14.1, 13.9, 12.7.; HRMS (EI) *m/z* calcd for C_30_H_30_N_2_O_2_ [M]^+^: 450.2307, Found: 450.2310.

**10. Single Crystal X-ray Diffraction Data**

**10.1. X-ray Crystallographic Data of 4p (CCDC No. 2421296)**Single-crystal sample (**4p**) was prepared in Methanol (Figure S7).


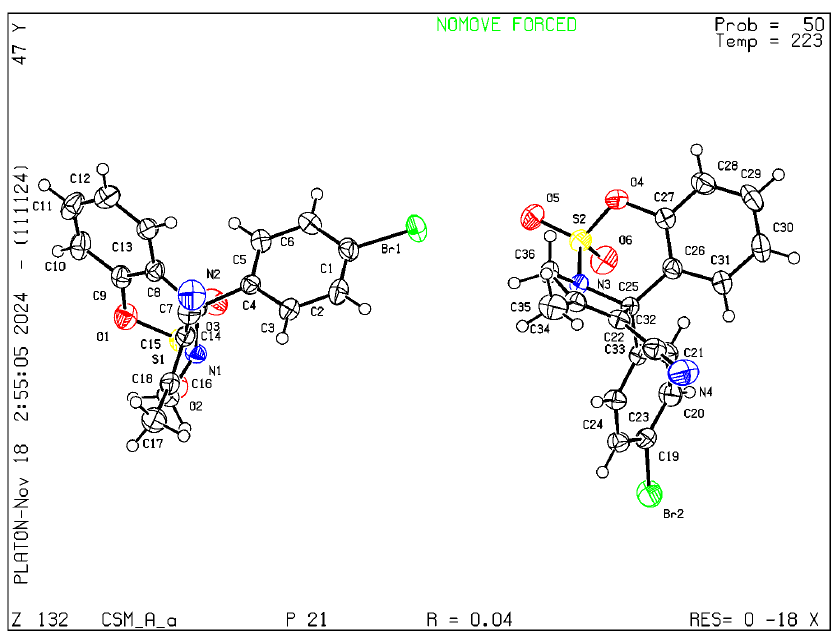


**Figure S7**. The Ellipsoid (50% probability) Plot of **4p**.

**Table S2**. Crystal Data and Structure Refinement for **4p**.

Identification code **4p**

Empirical formula C18 H13 Br N2 O3 S

Formula weight 417.27

Temperature 223(2) K

Wavelength 0.71073 Å

Crystal system Monoclinic

Space group P2**_1_**

Unit cell dimensions a = 9.6352(11) Å α= 90°.

b = 14.1568(14) Å β= 106.740(4)°.

c = 13.2500(15) Å γ = 90°.

Volume 1730.8(3) Å3

Z 4

Density (calculated) 1.601 Mg/m3

Absorption coefficient 2.515 mm-1

F(000) 840

Crystal size 0.180 x 0.153 x 0.139 mm3

Theta range for data collection 2.155 to 28.346°.

Index ranges -12<=h<=12, -18<=k<=18, -17<=l<=17

Reflections collected 36418

Independent reflections 8556 [R(int) = 0.0717]

Completeness to theta = 25.242° 100.0 %

Absorption correction Semi-empirical from equivalents

Max. and min. transmission 0.7457 and 0.5938

Refinement method Full-matrix least-squares on F2

Data / restraints / parameters 8556 / 1 / 453

Goodness-of-fit on F2 0.986

Final R indices [I>2sigma(I)] R1 = 0.0422, wR2 = 0.0913

R indices (all data) R1 = 0.0657, wR2 = 0.1012

Absolute structure parameter 0.040(6)

Extinction coefficient n/a

Largest diff. peak and hole 0.465 and -0.470 e.Å-3

**10.2. X-ray Crystallographic Data of 5a (CCDC No. 2432149)**

Single-crystal sample (**5a**) was prepared in DCM:Hexane (Figure S8).


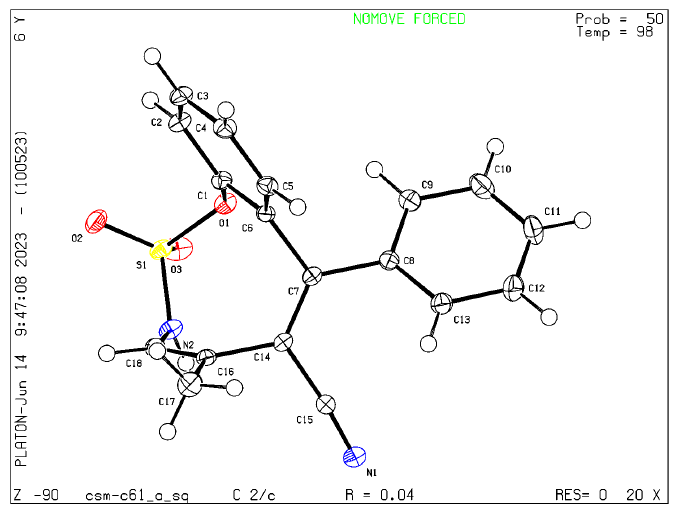


**Figure S8**. The Ellipsoid (50% probability) Plot of **5a**.

**Table S3**. Crystal Data and Structure Refinement for **5a**.

Identification code **5a**

Empirical formula C20 H20 Cl N2 O2 S

Formula weight 387.89

Temperature 98(2) K

Wavelength 0.71073 Å

Crystal system Monoclinic

Space group C2/c

Unit cell dimensions a = 26.9240(4) Å α= 90°.

b = 11.2837(2) Å β= 95.7320(10)°.

c = 12.0041(2) Å γ = 90°.

Volume 3628.64(10) Å3

Z 8

Density (calculated) 1.420 Mg/m3

Absorption coefficient 0.343 mm-1

F(000) 1624

Crystal size 0.285 x 0.200 x 0.152 mm3

Theta range for data collection 1.958 to 28.352°.

Index ranges -35<=h<=35, -15<=k<=15, -15<=l<=16

Reflections collected 33880

Independent reflections 4518 [R(int) = 0.0321]

Completeness to theta = 25.242° 99.9 %

Absorption correction Semi-empirical from equivalents

Max. and min. transmission 0.7457 and 0.7142

Refinement method Full-matrix least-squares on F2

Data / restraints / parameters 4518 / 0 / 217

Goodness-of-fit on F2 1.038

Final R indices [I>2sigma(I)] R1 = 0.0421, wR2 = 0.1203

R indices (all data) R1 = 0.0470, wR2 = 0.1247

Extinction coefficient n/a

Largest diff. peak and hole 0.750 and -0.732 e.Å-3

**10.3. X-ray Crystallographic Data of 6n (CCDC No. 2421297)**

Single-crystal sample (**6n**) was prepared in DCM:Hexane (Figure S9).


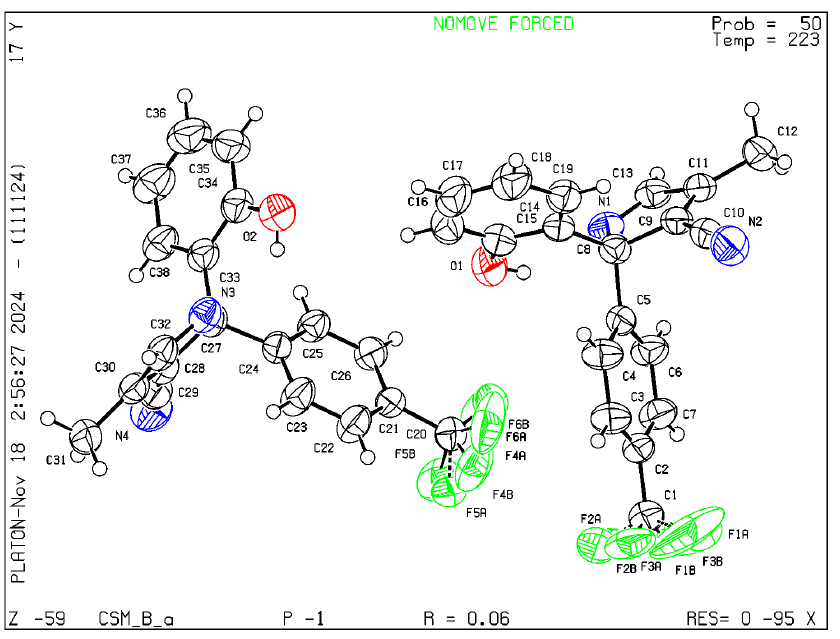


**Figure S9**. The Ellipsoid (50% probability) Plot of **6n**.

**Table S4.** Crystal Data and Structure Refinement for **6n**.

Identification code **6n**

Empirical formula C19 H13 F3 N2 O

Formula weight 342.31

Temperature 223(2) K

Wavelength 0.71073 Å

Crystal system Triclinic

Space group P-1

Unit cell dimensions a = 10.444(6) Å α= 81.243(19)°.

b = 11.192(6) Å β= 73.59(2)°.

c = 15.332(8) Å γ = 77.60(2)°.

Volume 1671.1(16) Å3

Z 4

Density (calculated) 1.361 Mg/m3

Absorption coefficient 0.108 mm-1

F(000) 704

Crystal size 0.143 x 0.142 x 0.085 mm3

Theta range for data collection 1.872 to 28.661°.

Index ranges -13<=h<=13, -14<=k<=14, -20<=l<=20

Reflections collected 71734

Independent reflections 8372 [R(int) = 0.1328]

Completeness to theta = 25.242° 100.0 %

Absorption correction Semi-empirical from equivalents

Max. and min. transmission 0.7457 and 0.6880

Refinement method Full-matrix least-squares on F2

Data / restraints / parameters 8372 / 0 / 509

Goodness-of-fit on F2 1.004

Final R indices [I>2sigma(I)] R1 = 0.0609, wR2 = 0.1518

R indices (all data) R1 = 0.1854, wR2 = 0.2071

Extinction coefficient n/a

Largest diff. peak and hole 0.206 and -0.237 e.Å-3

**11. References**

[1] Y. Tatsuno, T. Yoshida, Seiotsuka, N. Al-Salem, B. L. Shaw, "(η3-Allyl)Palladium(II) Complexes," *Inorg. Synth.* vol. 19 (1979): 220.

[2] R. Ardkhean, P. M. C. Roth, R. M. Maksymowicz, A. Curran, Q. Peng, R. S. Paton, S. P. Fletcher, "Enantioselective conjugate addition catalyzed by a copper-phosphoramidite complex: Computational and experimental exploration of asymmetric induction," *ACS Catal*. vol. 7 (2017): 6729.

[3] a) B. M. Trost, G. Meta, "Enantioselective Palladium-Catalyzed [3+2] Cycloaddition of Trimethylenemethane and Fluorinated Ketones," *Angew. Chem. Int. Ed*. vol. 57 (2018): 12333; b) B. M. Trost, S. M. Silverman, J. P. Stambuli, "Development of an Asymmetric Trimethylenemethane Cycloaddition Reaction: Application in the Enantioselective Synthesis of Highly Substituted Carbocycles," *J. Am. Chem. Soc*. vol. 133 (2011): 19483.

[4] a) S. M. Choi, K. D. Kim, J.-U. Park, Z. Xuan, J. H. Kim, "Pd-catalyzed [3 + 2] cycloaddition of cyclic ketimines and trimethylenemethanes toward *N*-fused pyrrolidines bearing a quaternary carbon," *RSC Adv*. vol. 12 (2022): 785; b) Z. Tong, Z. Tang, C.-T. Au, R. Qiu, "Nickel-Catalyzed Decarbonyloxidation of 3-Aryl Benzofuran-2(*3H*)-ones to 2-Hydroxybenzophenones," *J. Org. Chem*. vol. 85 (2020): 8533.

[5] Ø. W. Akselsen, T. V. Hansen, "*ortho*-Formylation of estrogens. Synthesis of the anti-cancer agent 2-methoxyestradiol," *Tetrahedron* vol. 67 (2011): 7738.

[6] L. Zhao, X. Wang, Q. Qiang, X. Zhao, F. Liu, S. Lu, Z.-Q. Rong, "Ruthenium-Catalysed Asymmetric Intramolecular Isomerization/Esterification Reaction: Direct Synthesis of Chiral Dihydrocoumarins," *Chin. J. Chem*. vol. 42 (2024): 1828.

[7] J.-S. Tian, Y. He, Z.-Y. Gao, X. Liu, S.-F. Dong, P. Wu, T.-P. Loh, "Water-Tolerant ortho-Acylation of Phenols," *Org. Lett*. vol. 23 (2021): 6594.

**12. NMR Spectra of All Compounds**


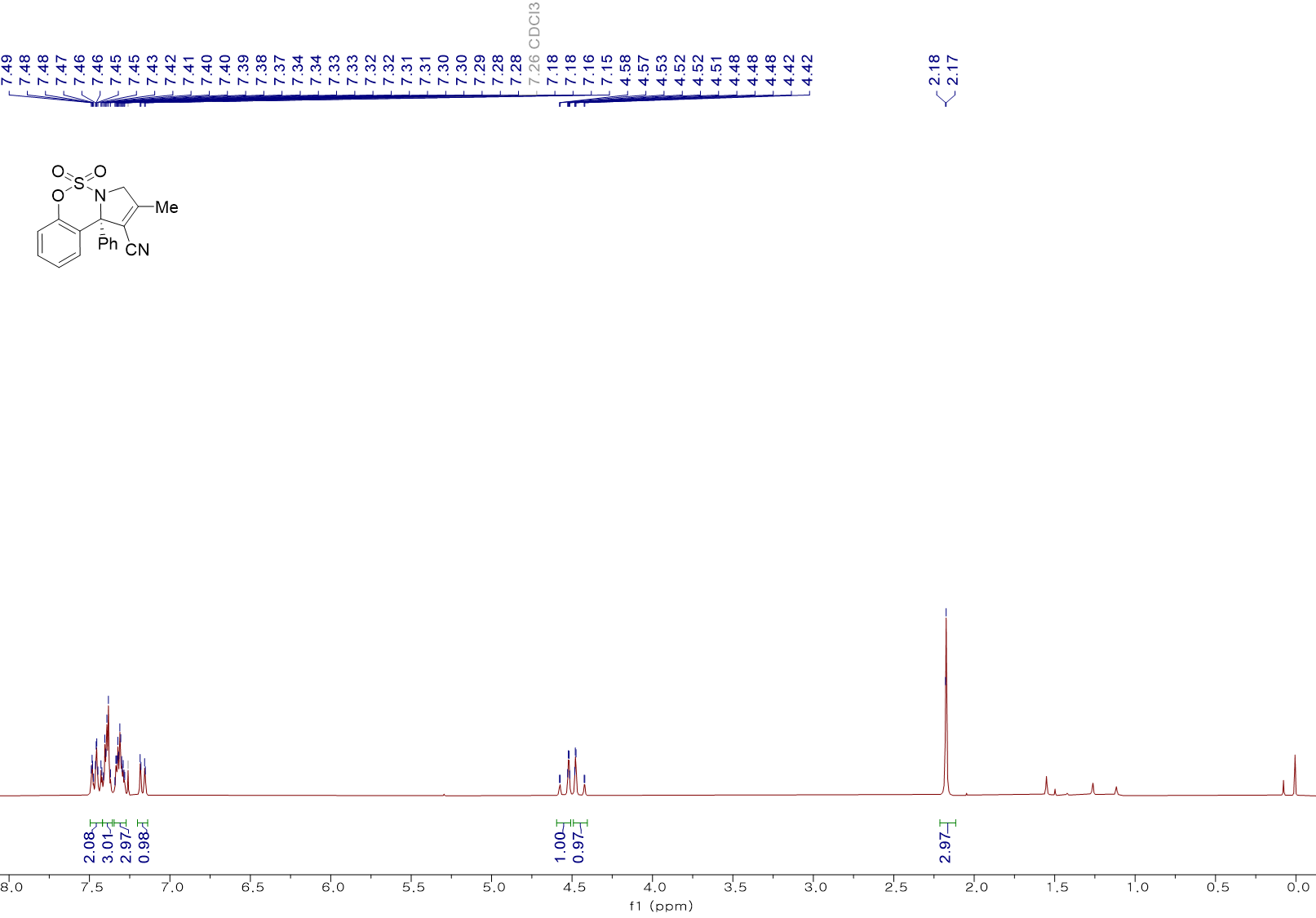


^1^H NMR spectrum of **4a**


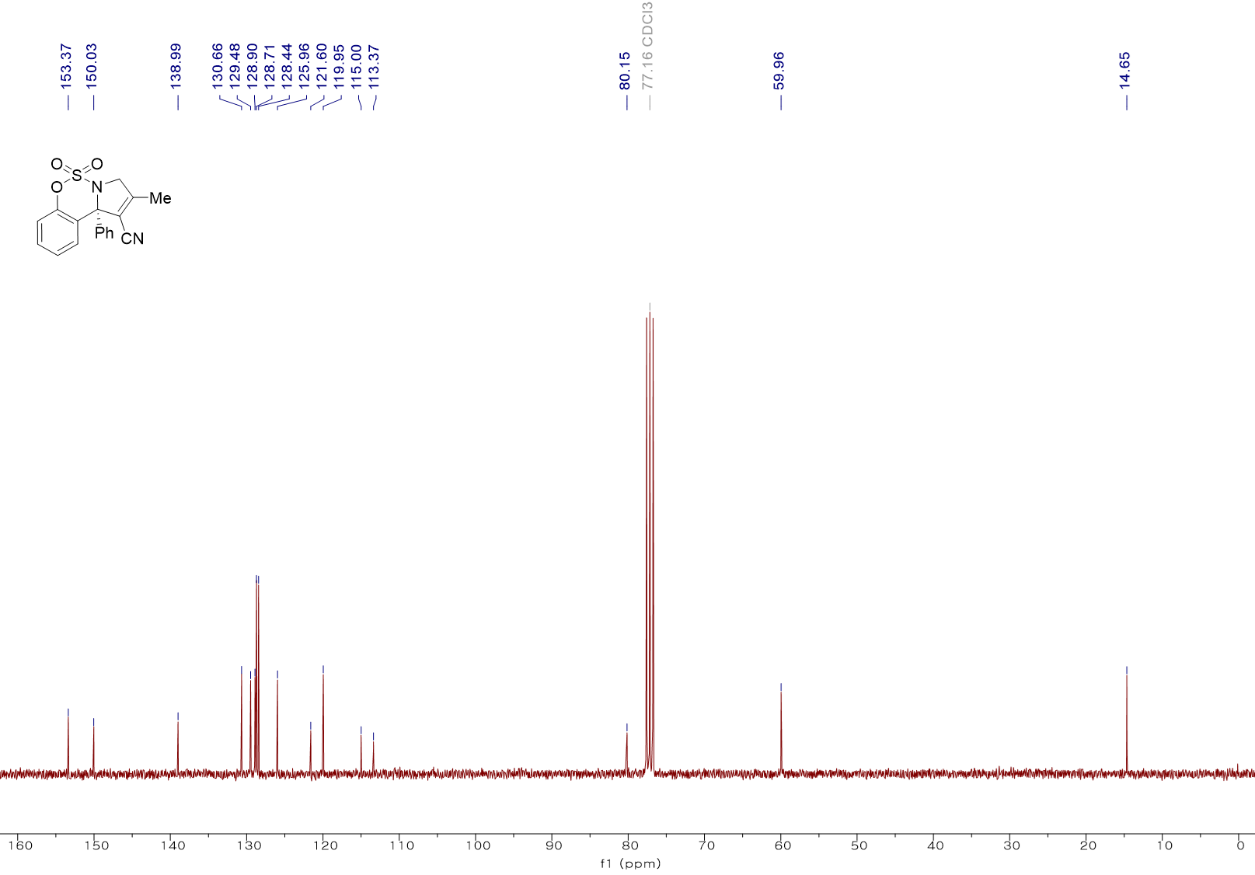


^13^C NMR spectrum of **4a**


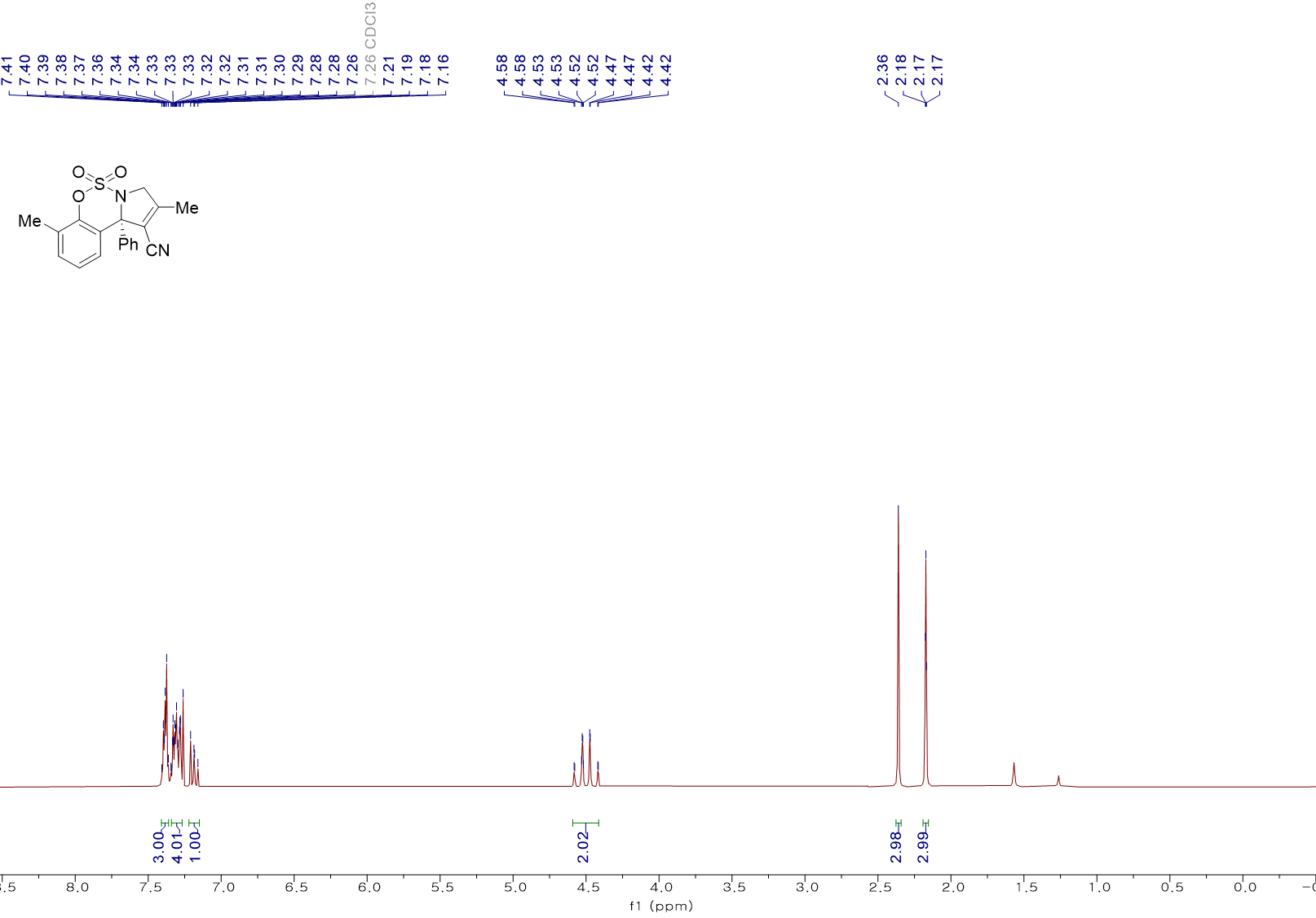


^1^H NMR spectrum of **4b**


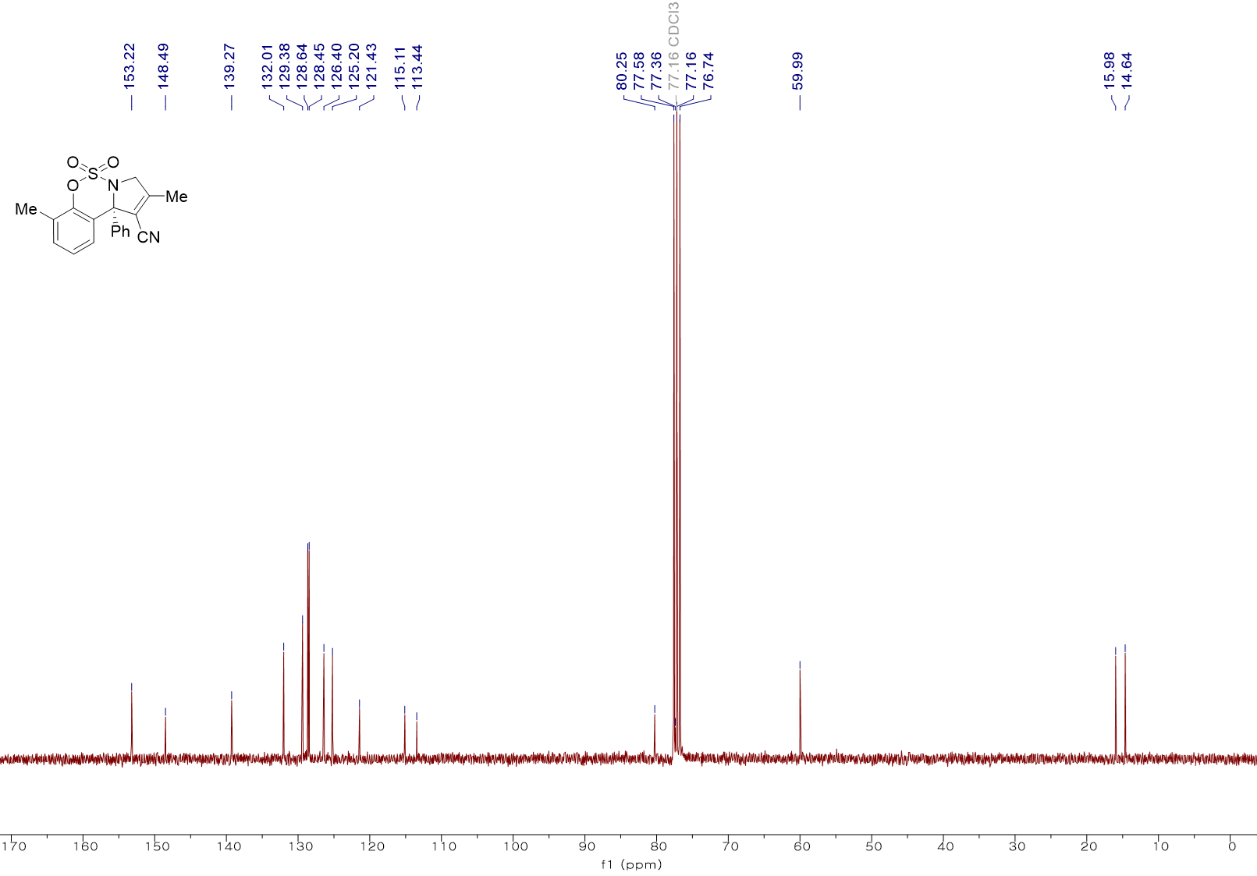


^13^C NMR spectrum of **4b**


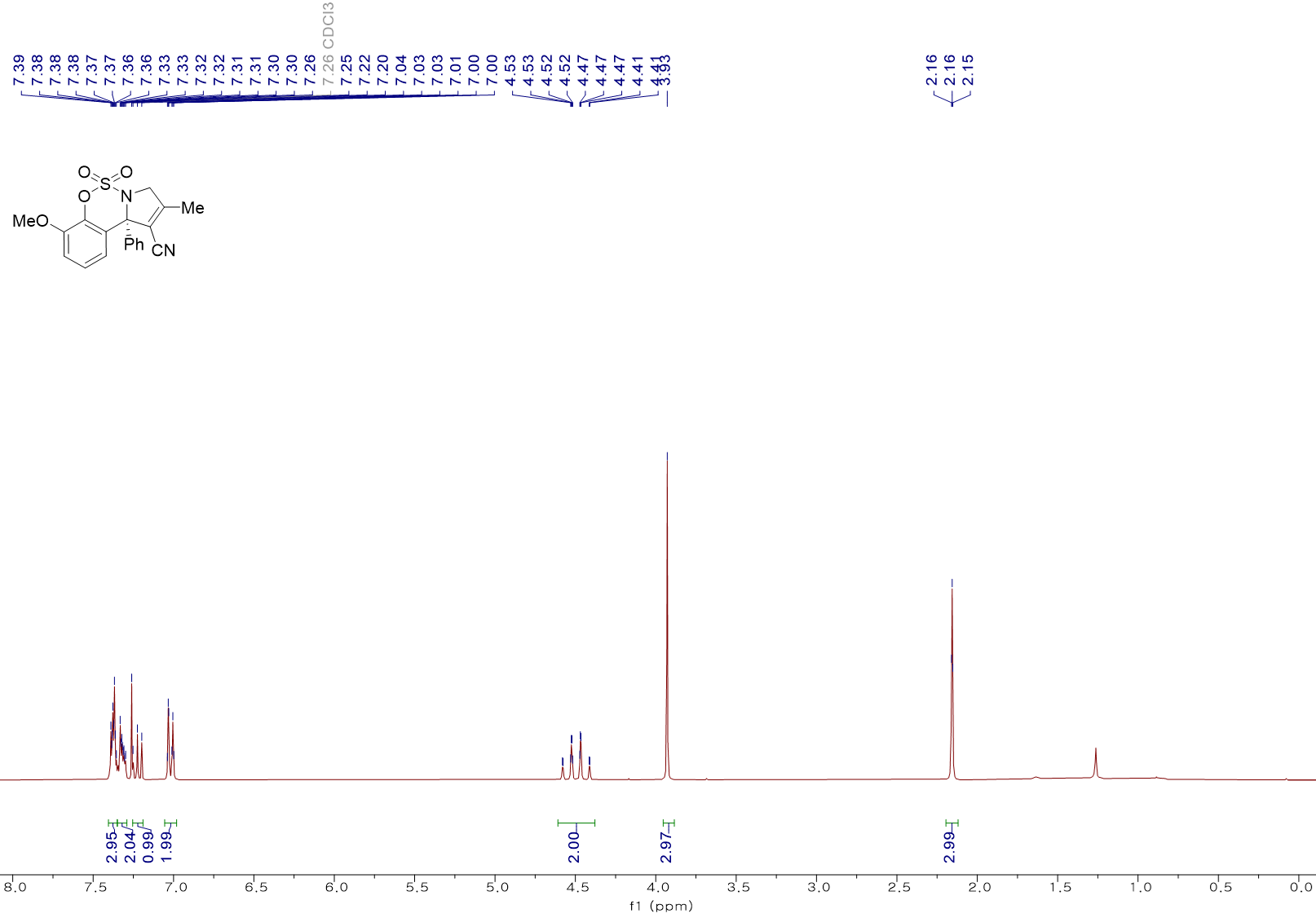


^1^H NMR spectrum of **4c**


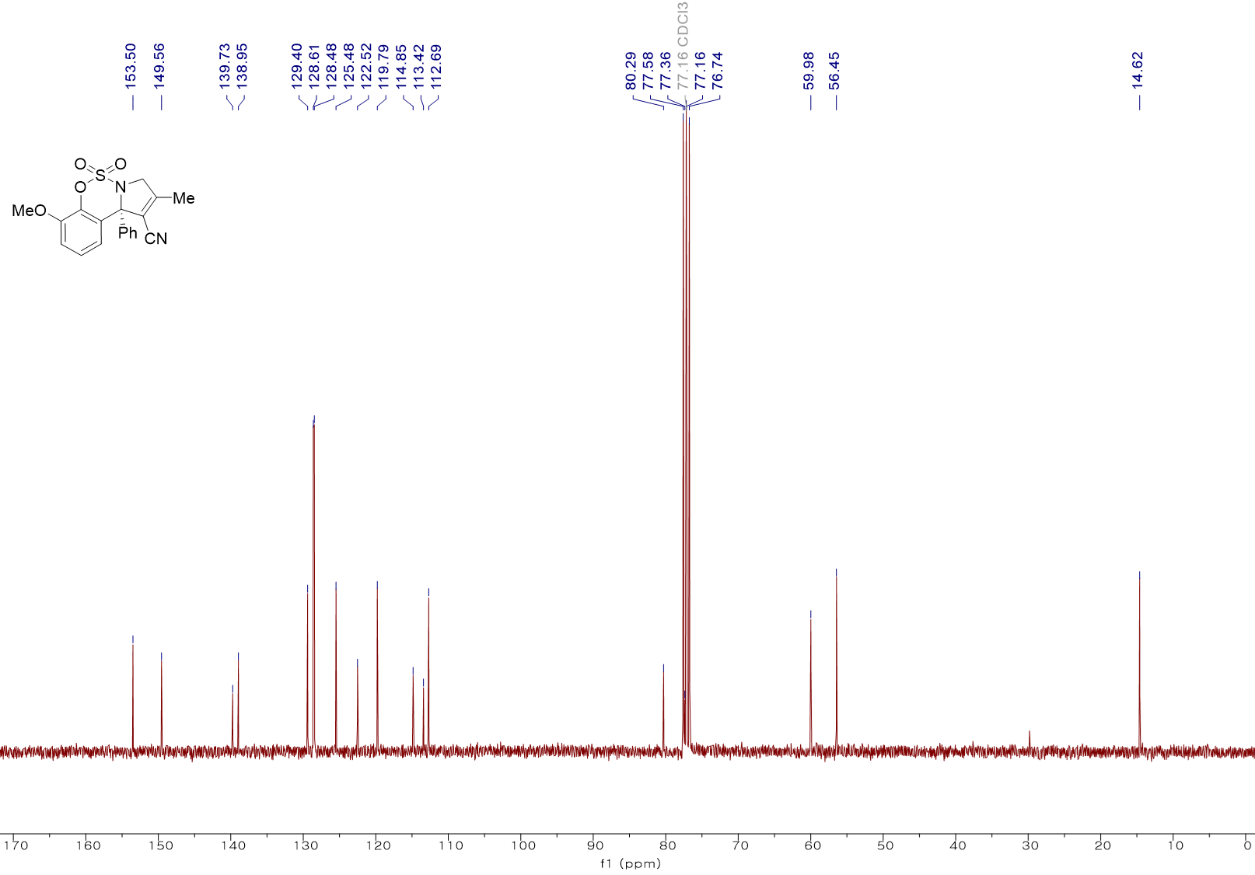


^13^C NMR spectrum of **4c**


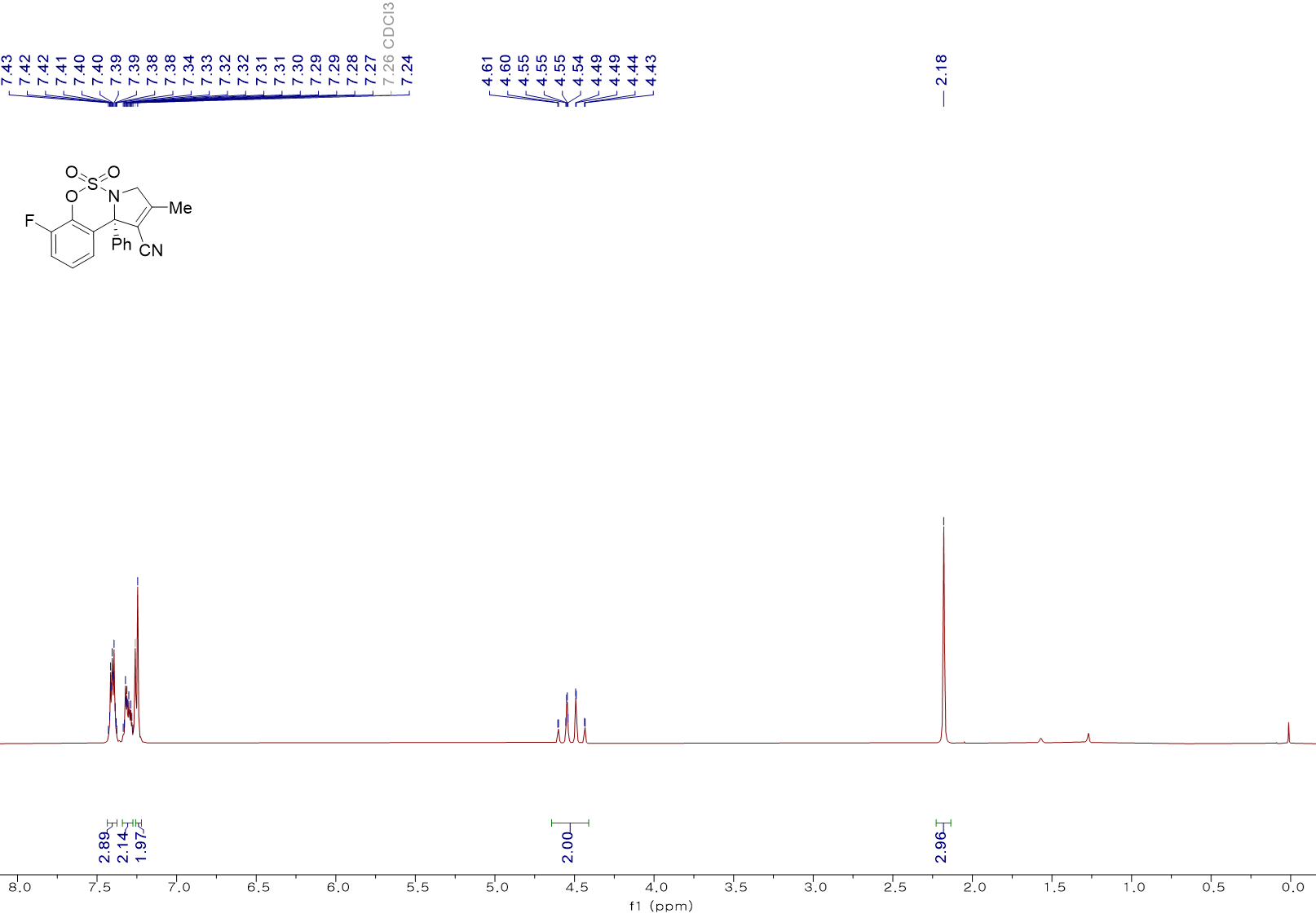


^1^H NMR spectrum of **4d**


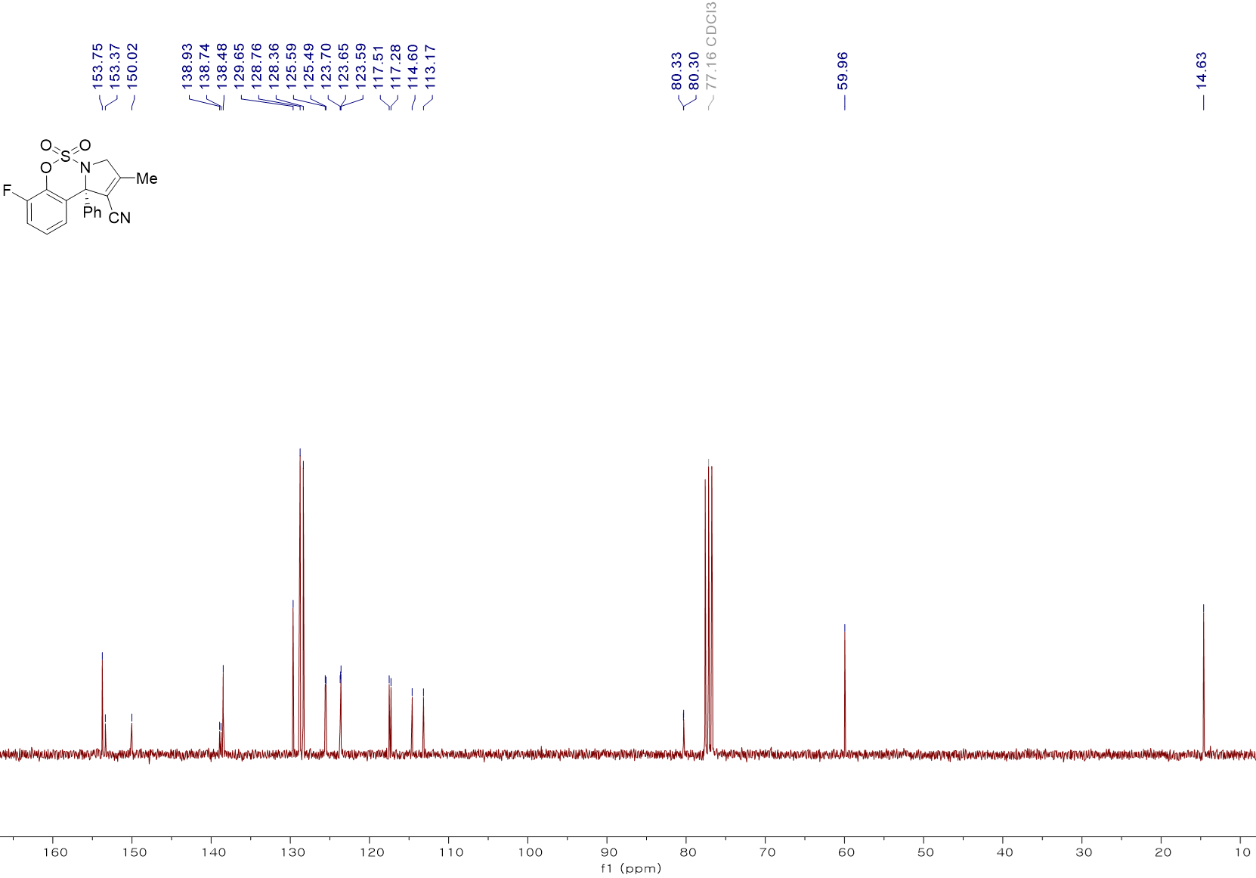


^13^C NMR spectrum of **4d**

_
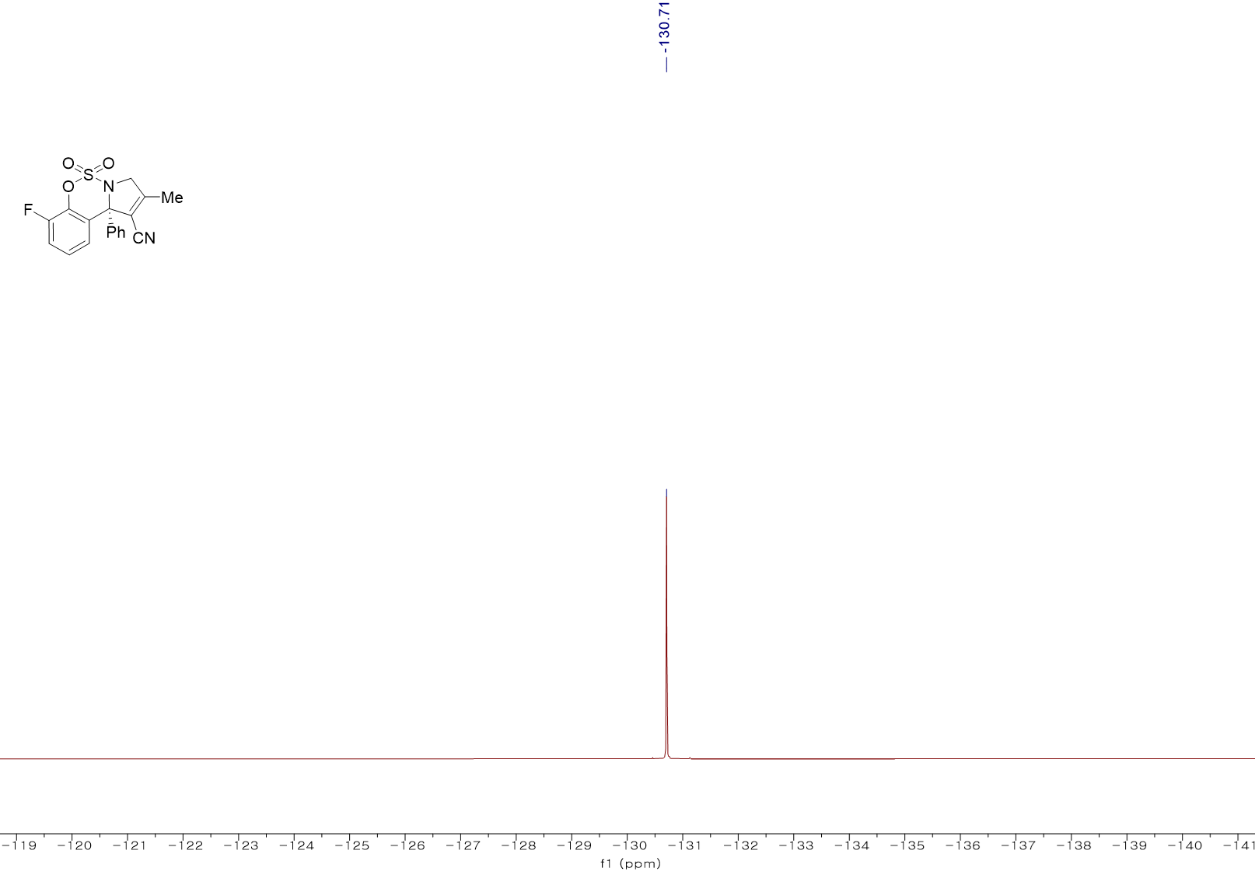
_

^19^F NMR spectrum of **4d**


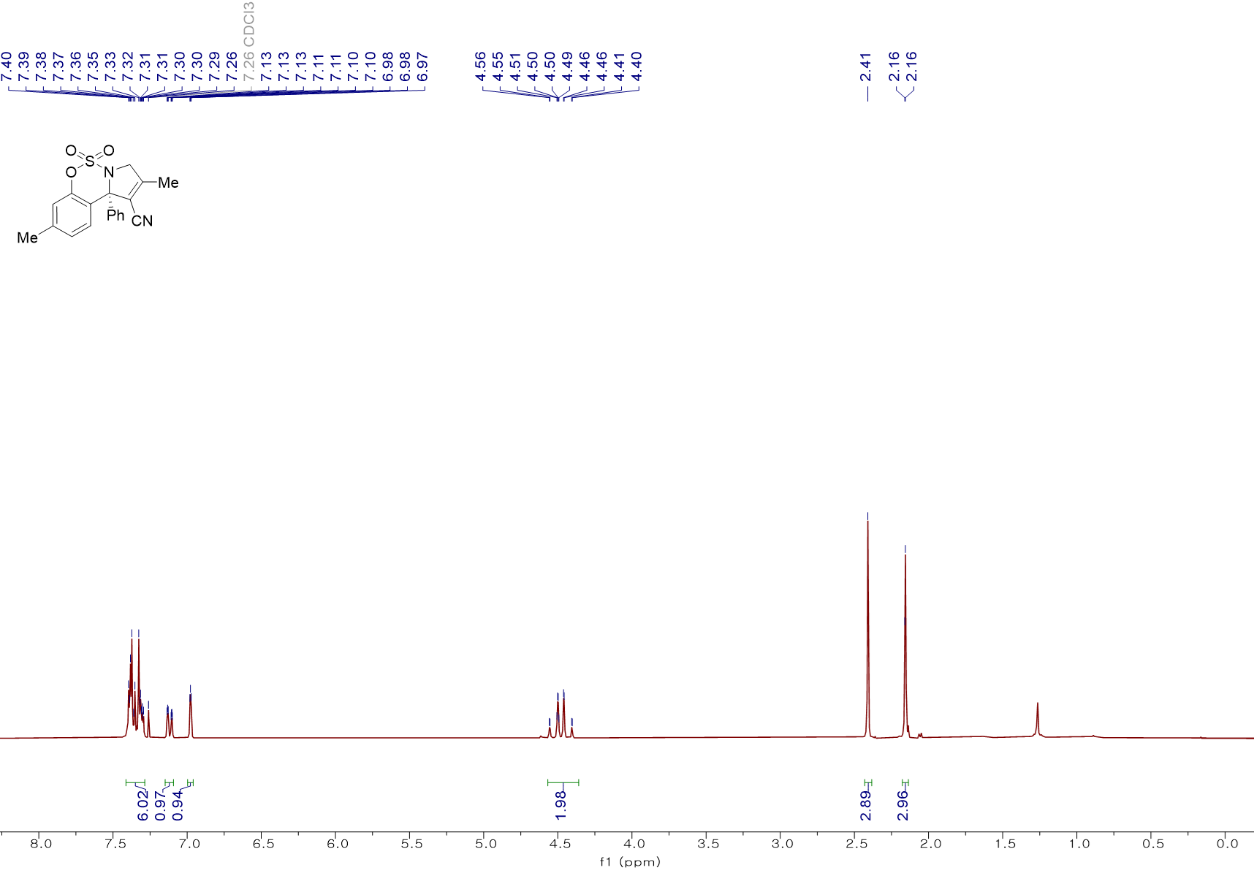


^1^H NMR spectrum of **4e**


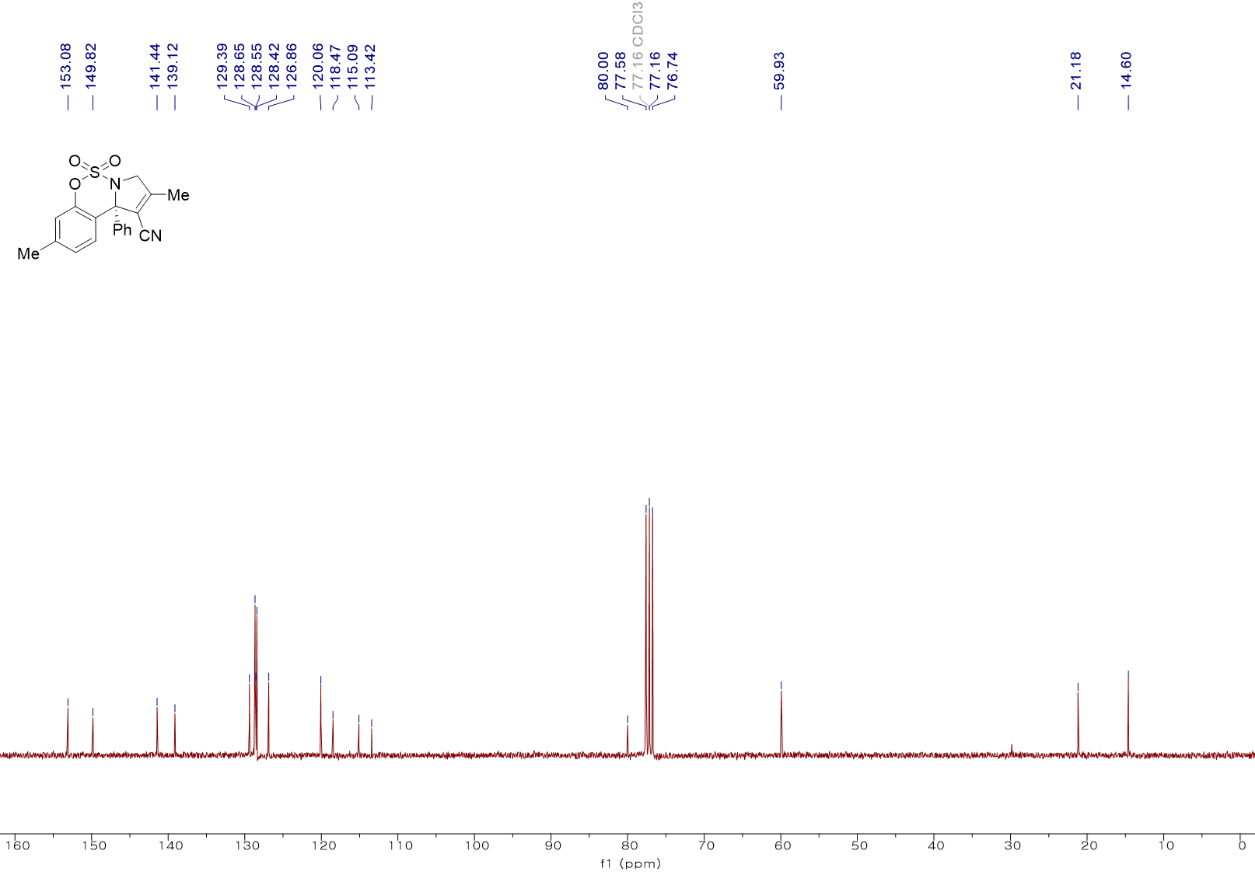


^13^C NMR spectrum of **4e**

**
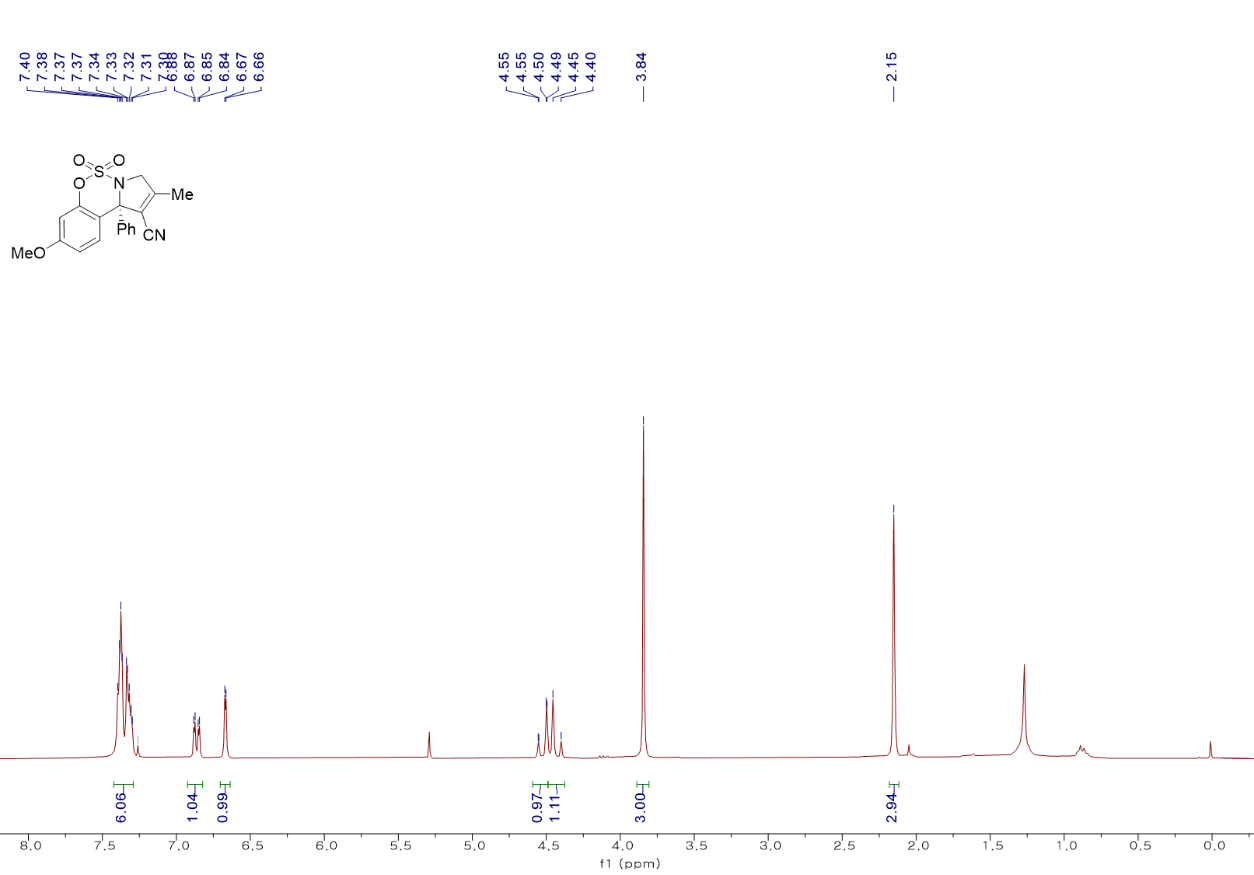
**

^1^H NMR spectrum of **4f**


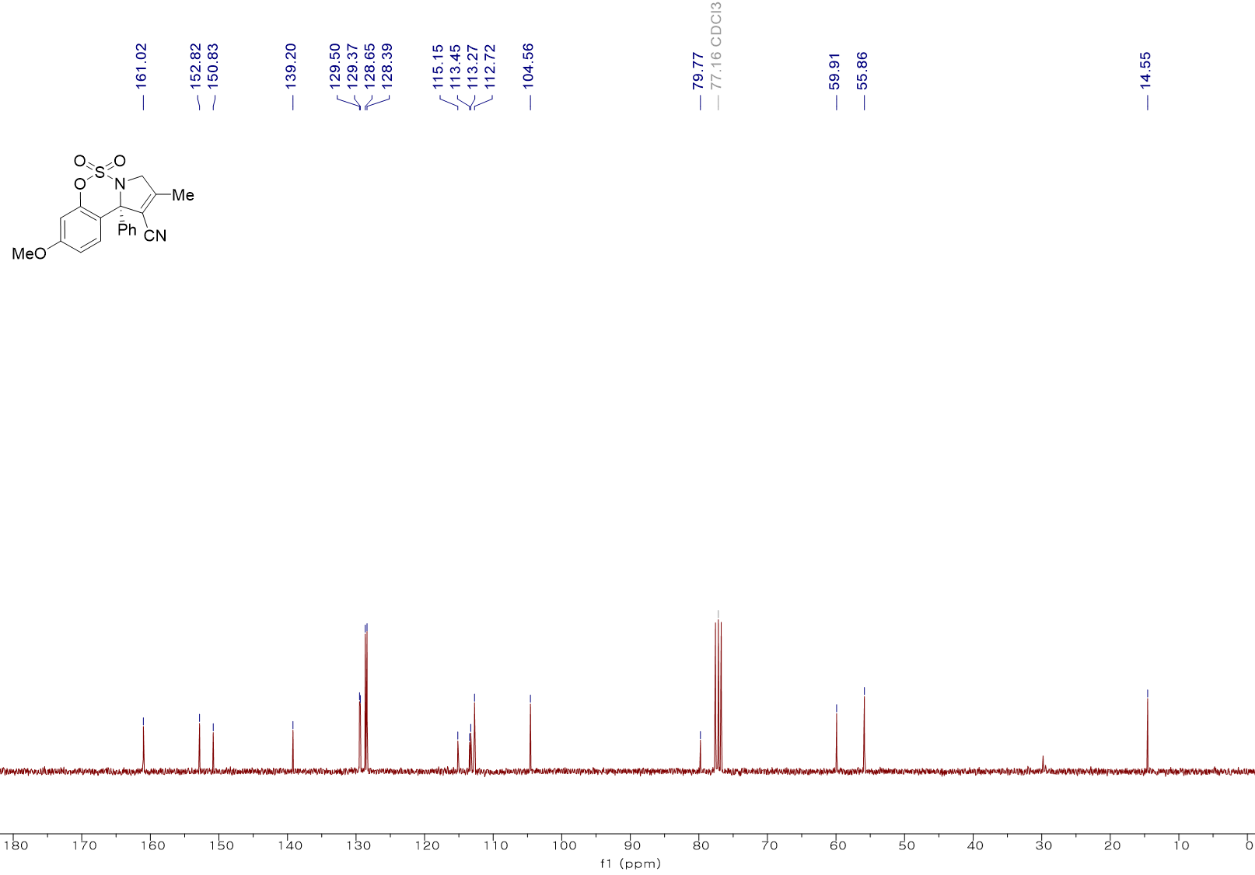


^13^C NMR spectrum of **4f**


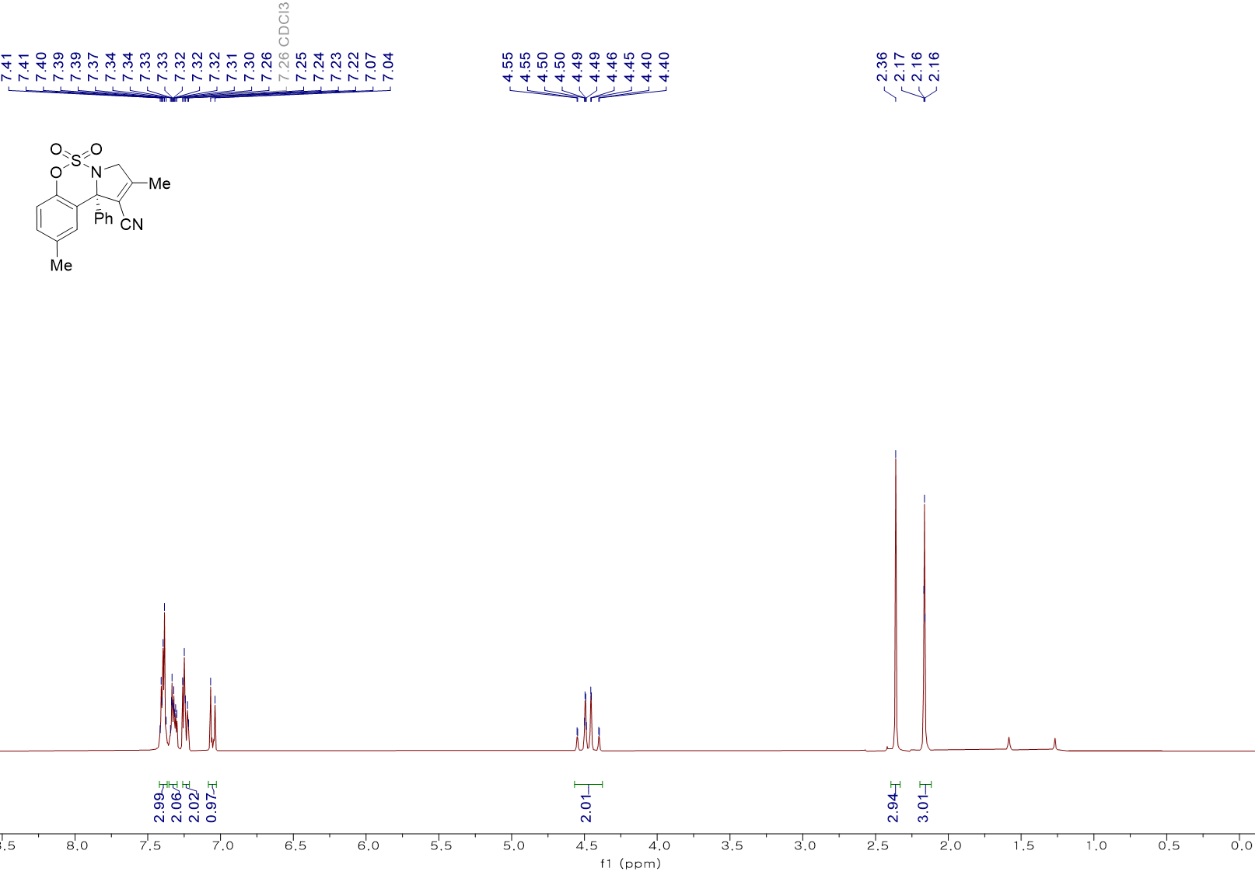


^1^H NMR spectrum of **4g**


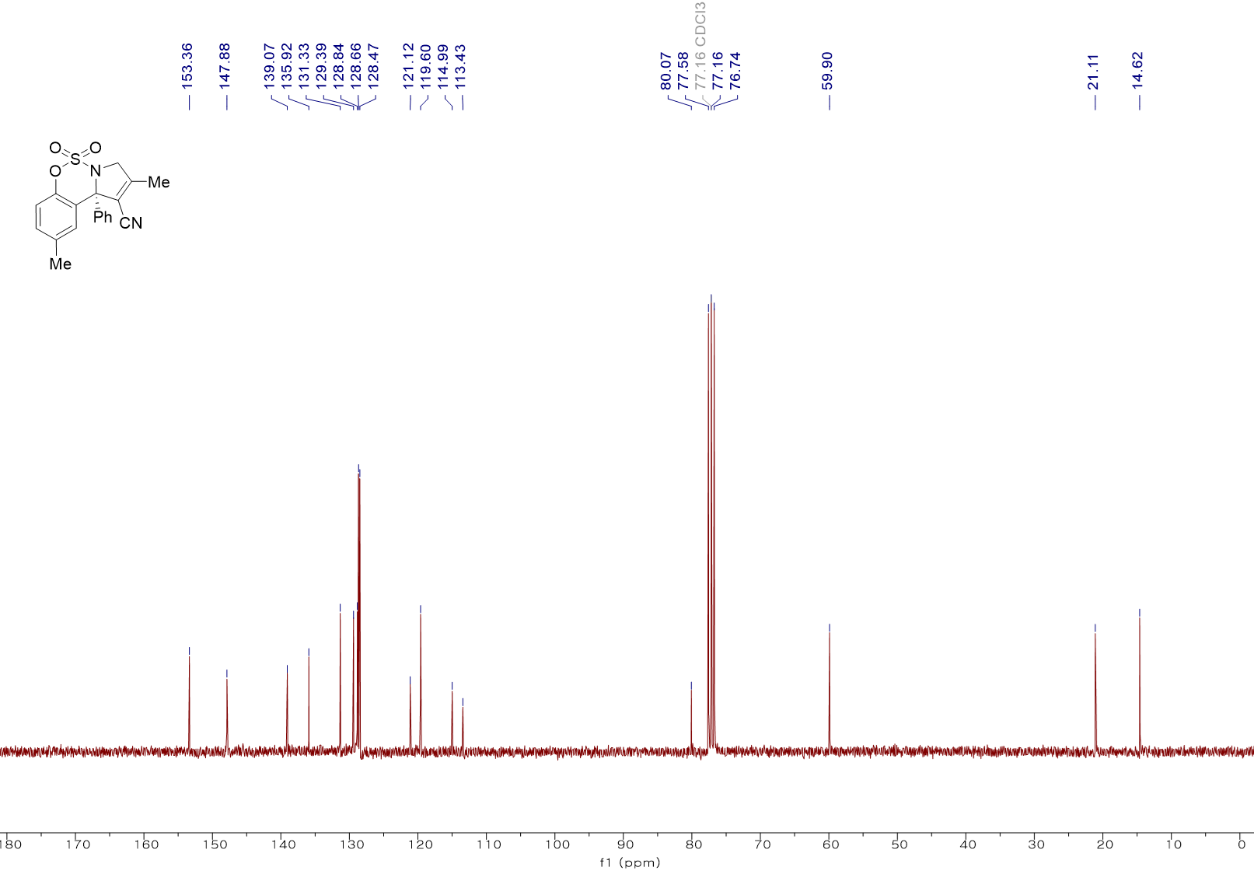


^13^C NMR spectrum of **4g**


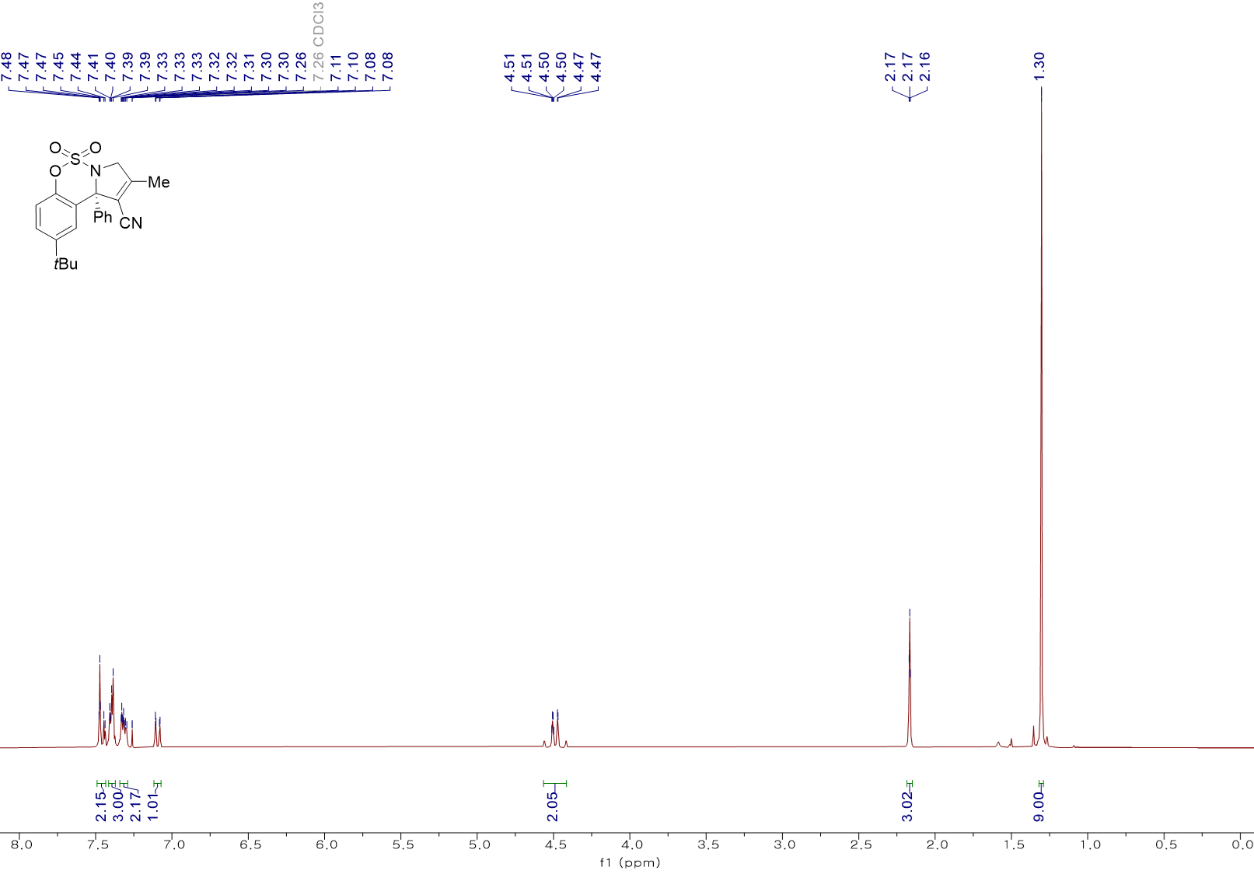


^1^H NMR spectrum of **4h**


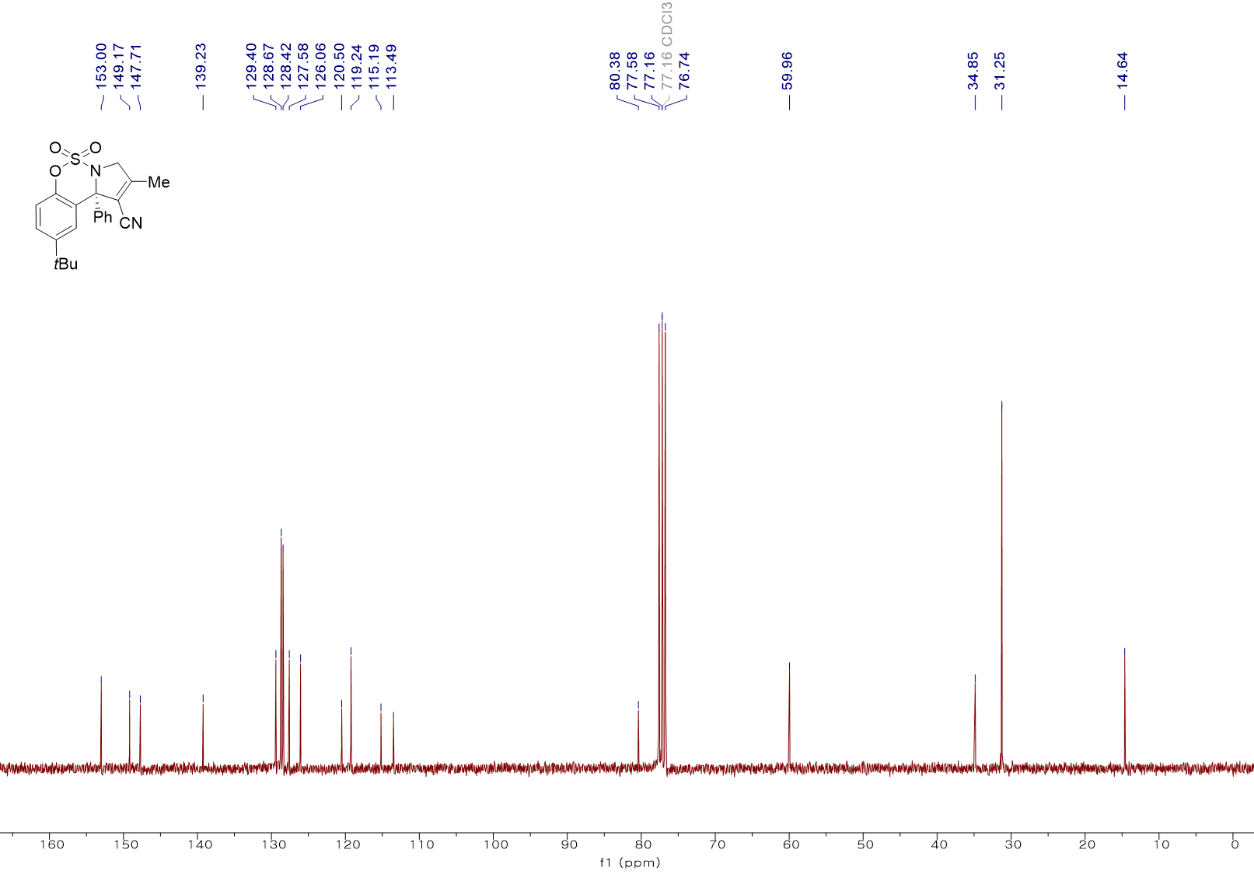


^13^C NMR spectrum of **4h**


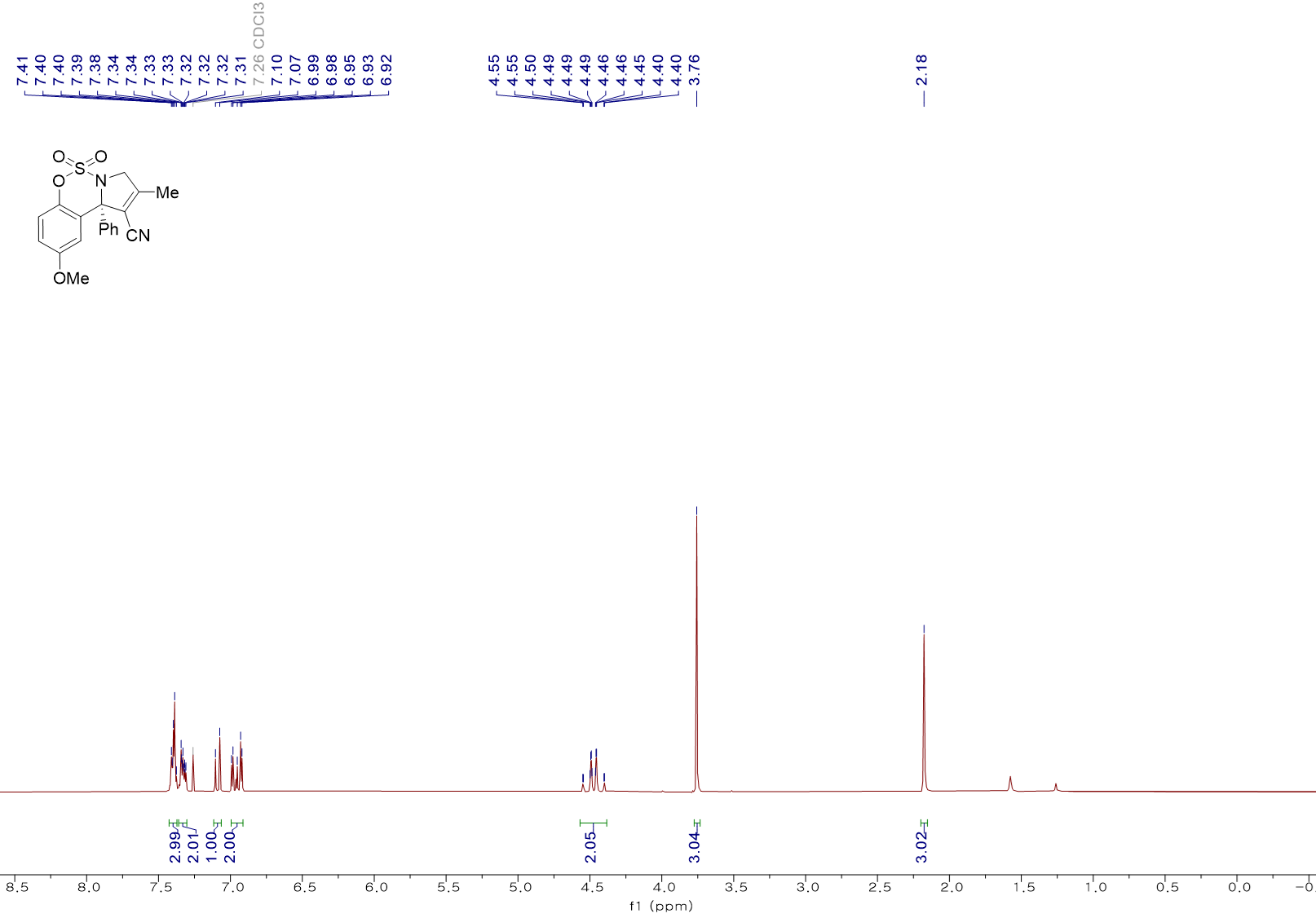


^1^H NMR spectrum of **4i**


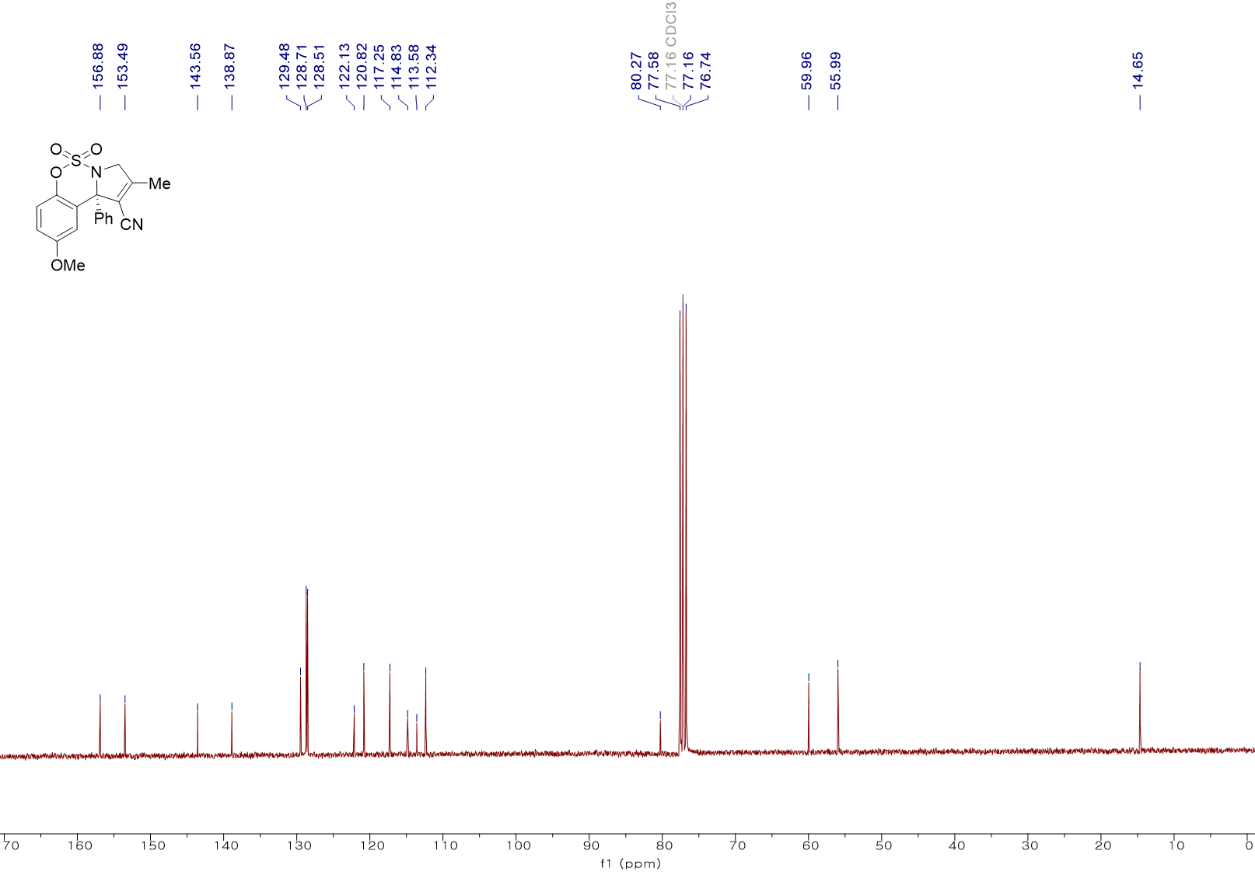


^13^C NMR spectrum of **4i**


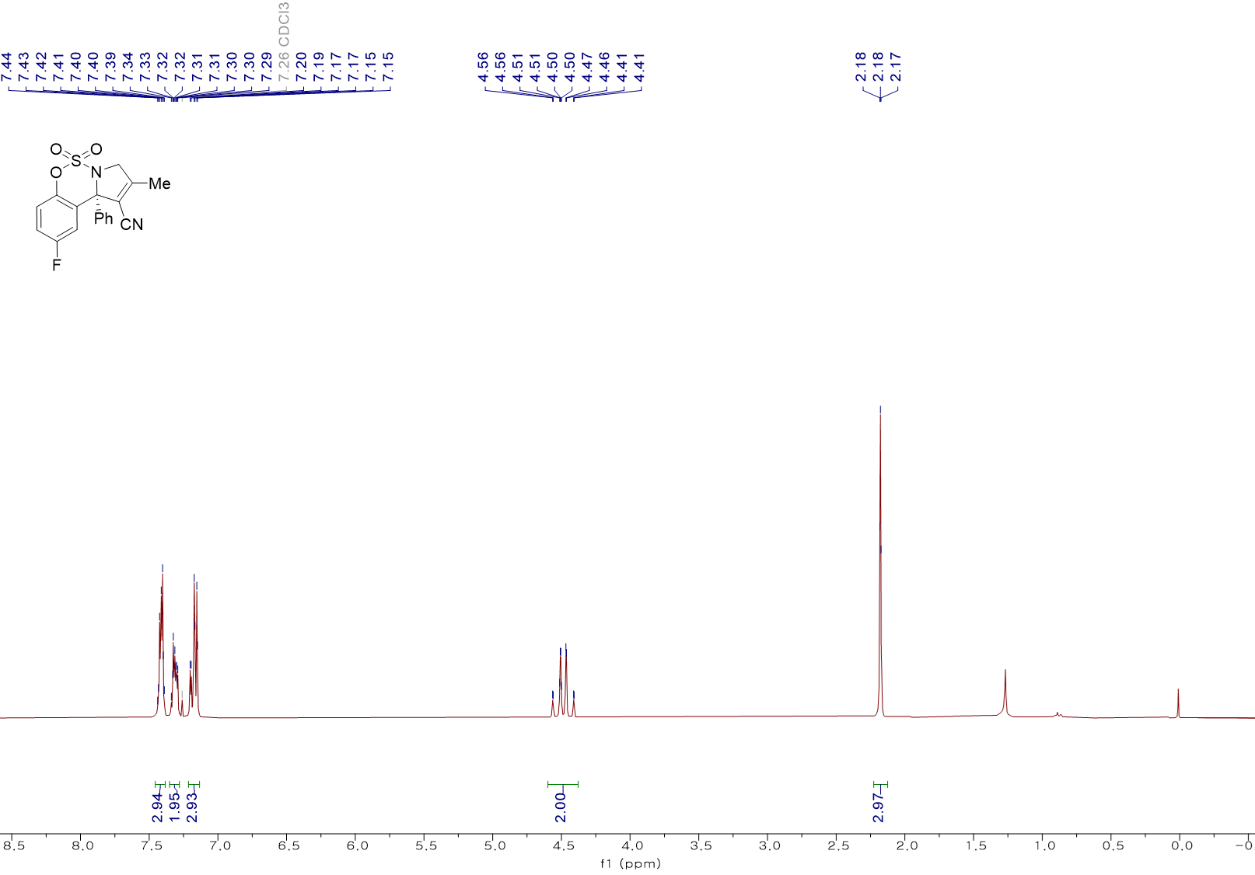


^1^H NMR spectrum of **4j**


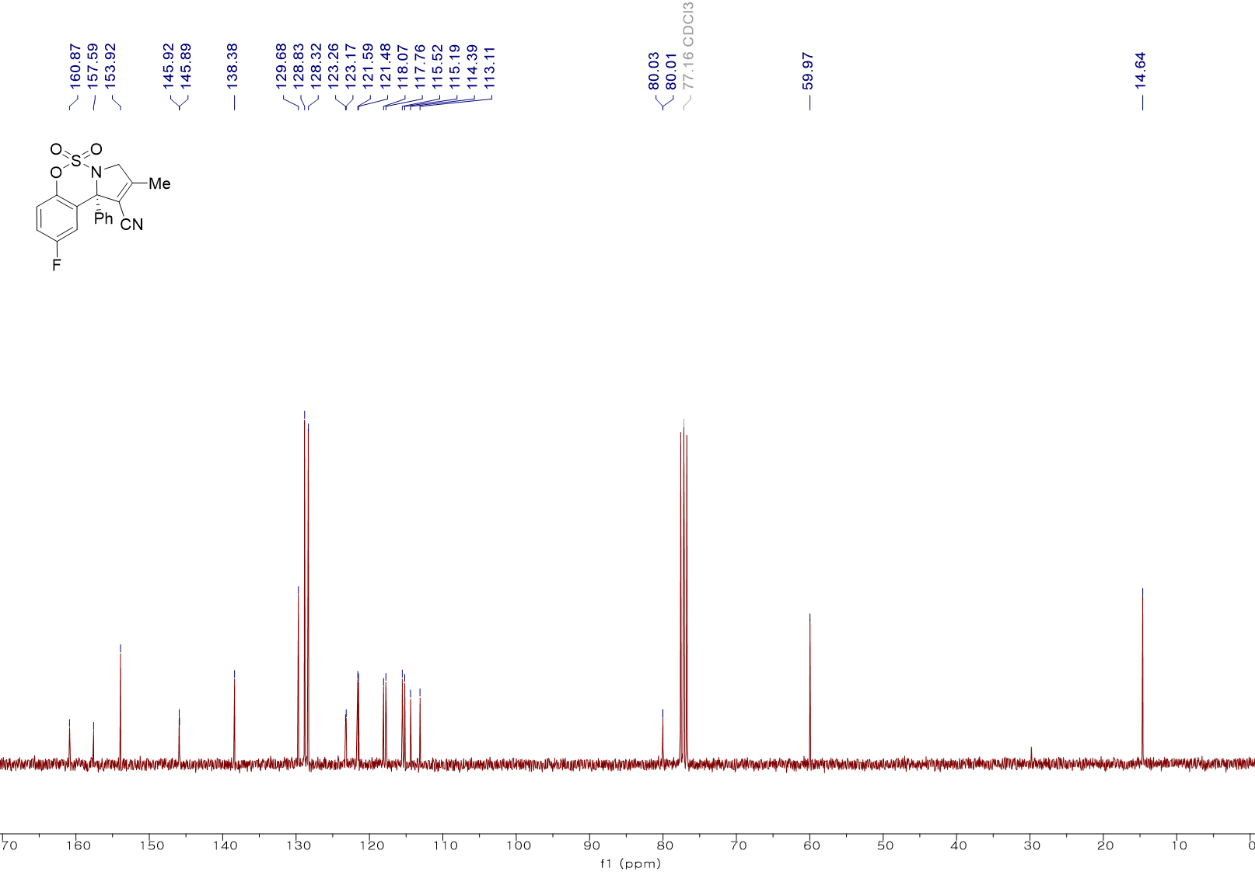


^13^C NMR spectrum of **4j**

_
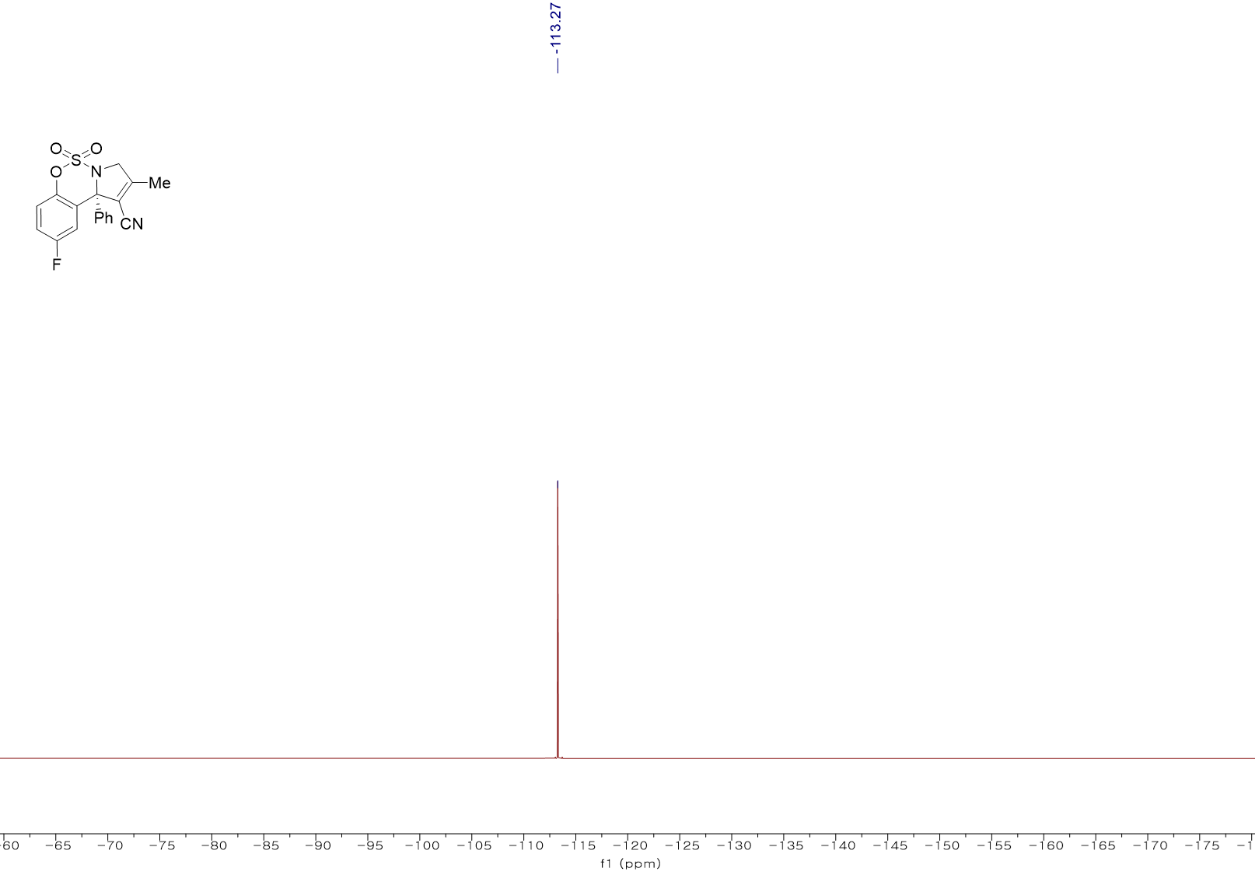
_

^19^F NMR spectrum of **4j**


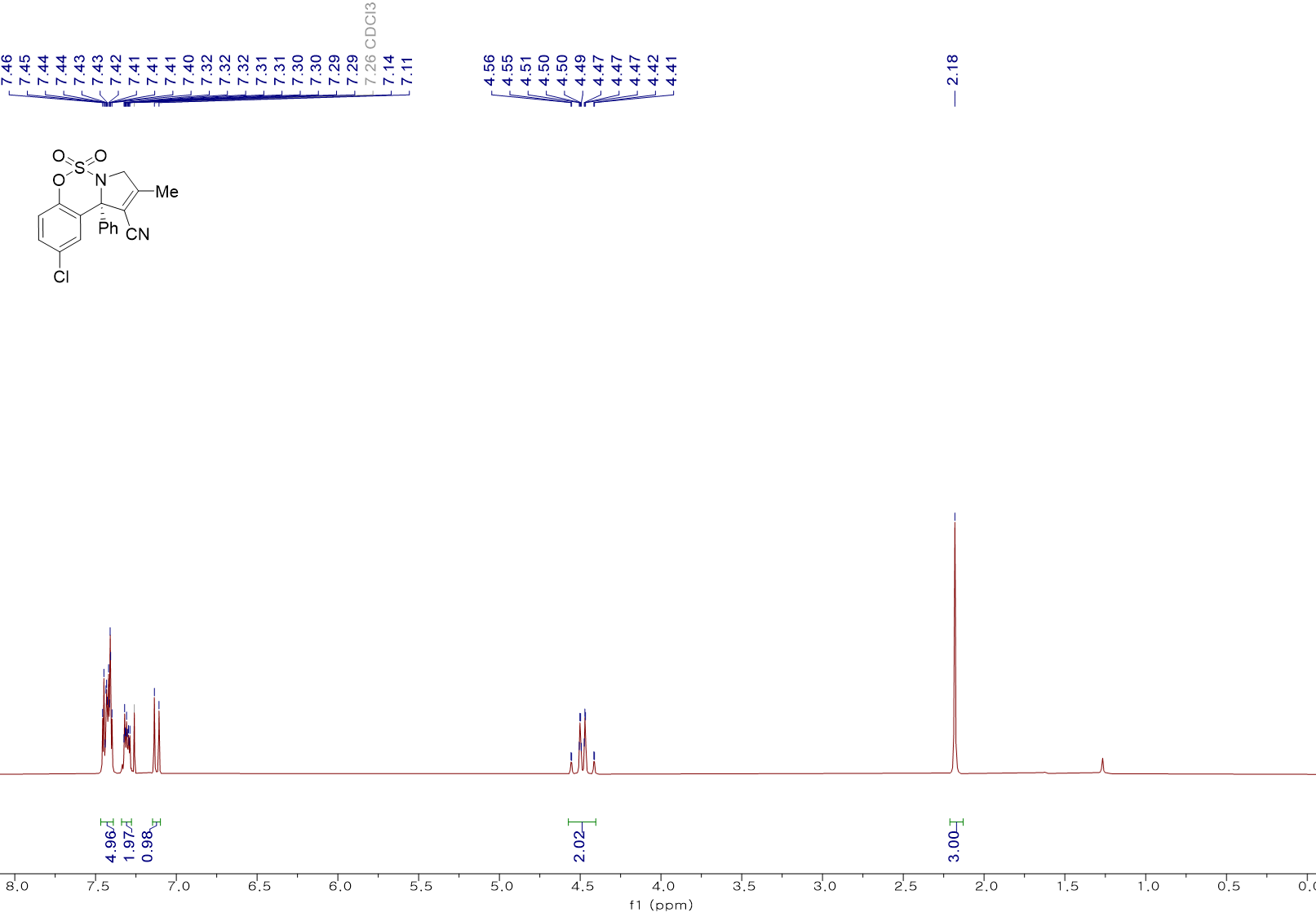


^1^H NMR spectrum of **4k**


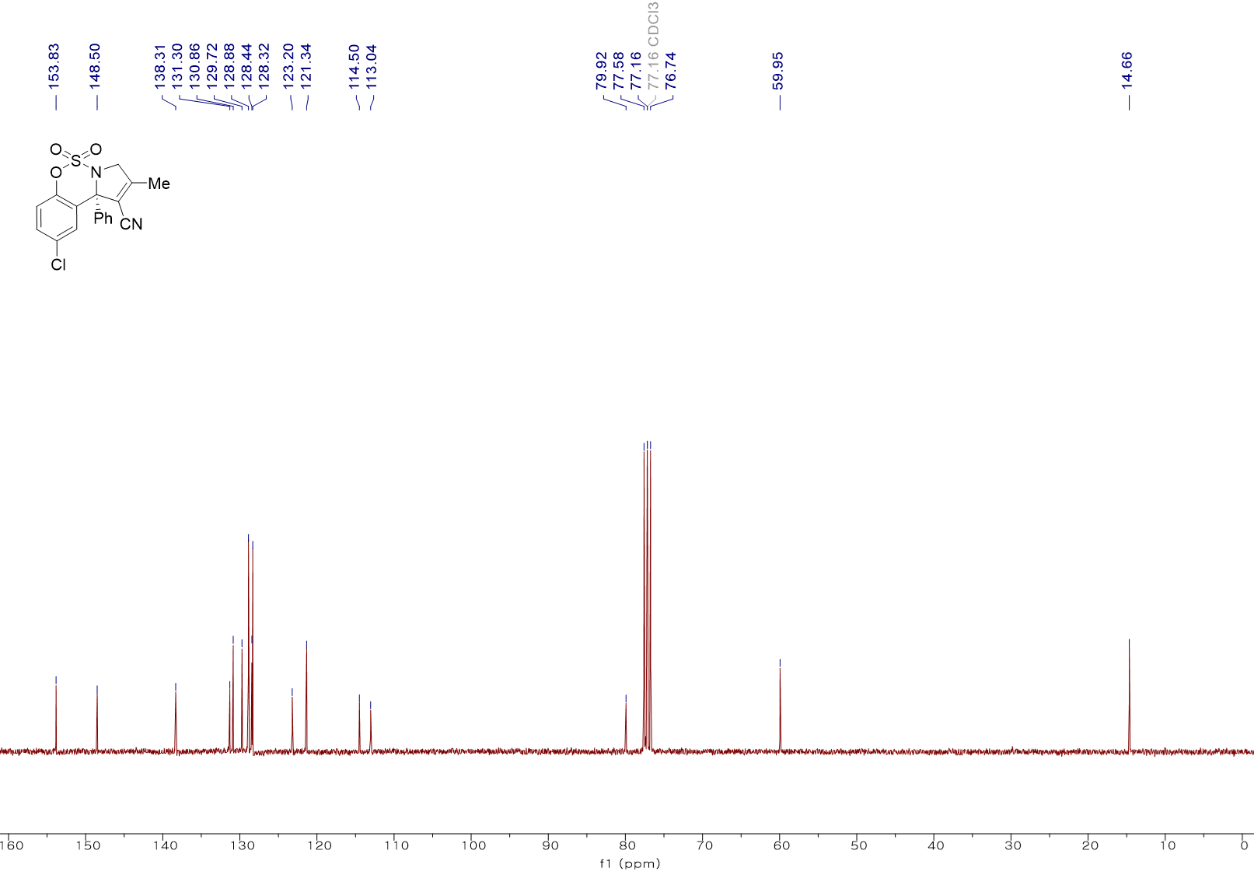


^13^C NMR spectrum of **4k**


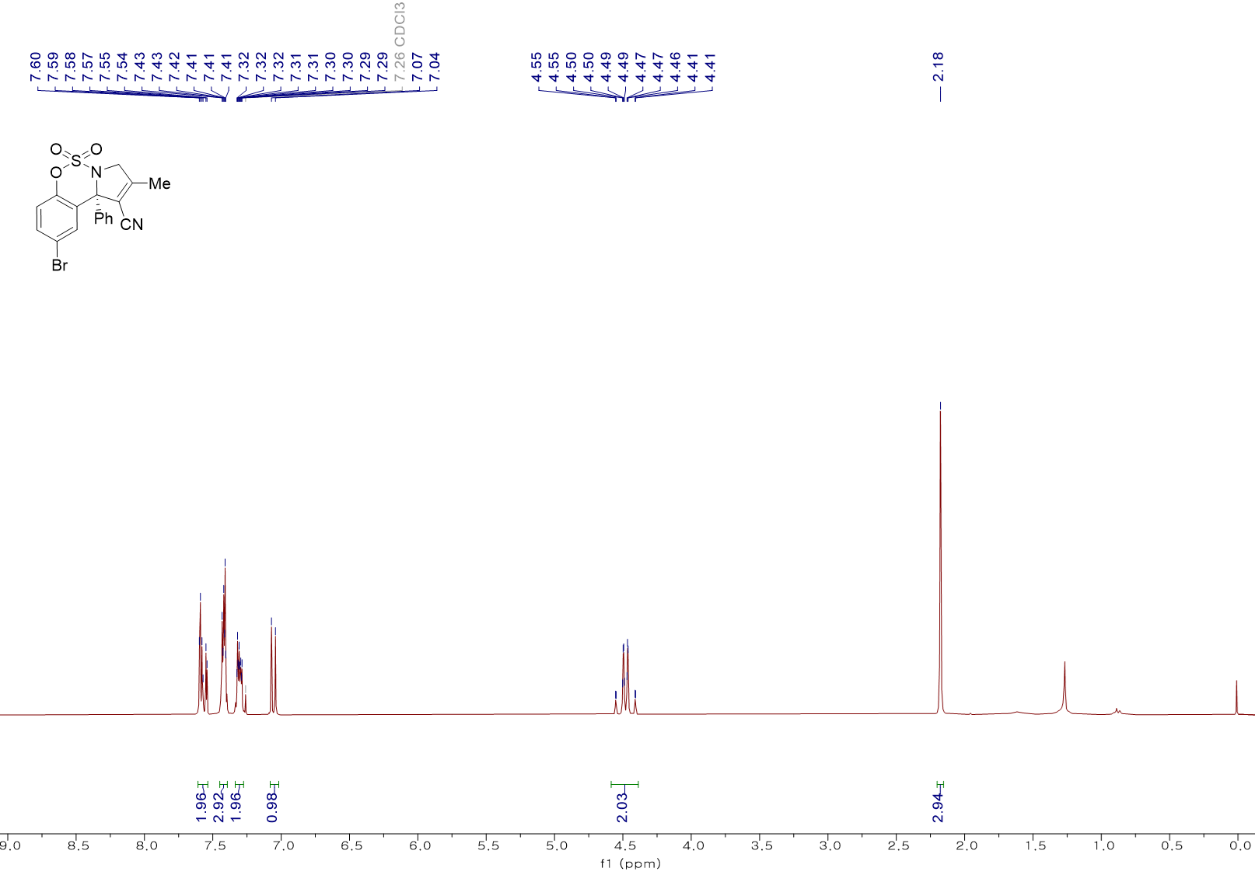


^1^H NMR spectrum of **4l**


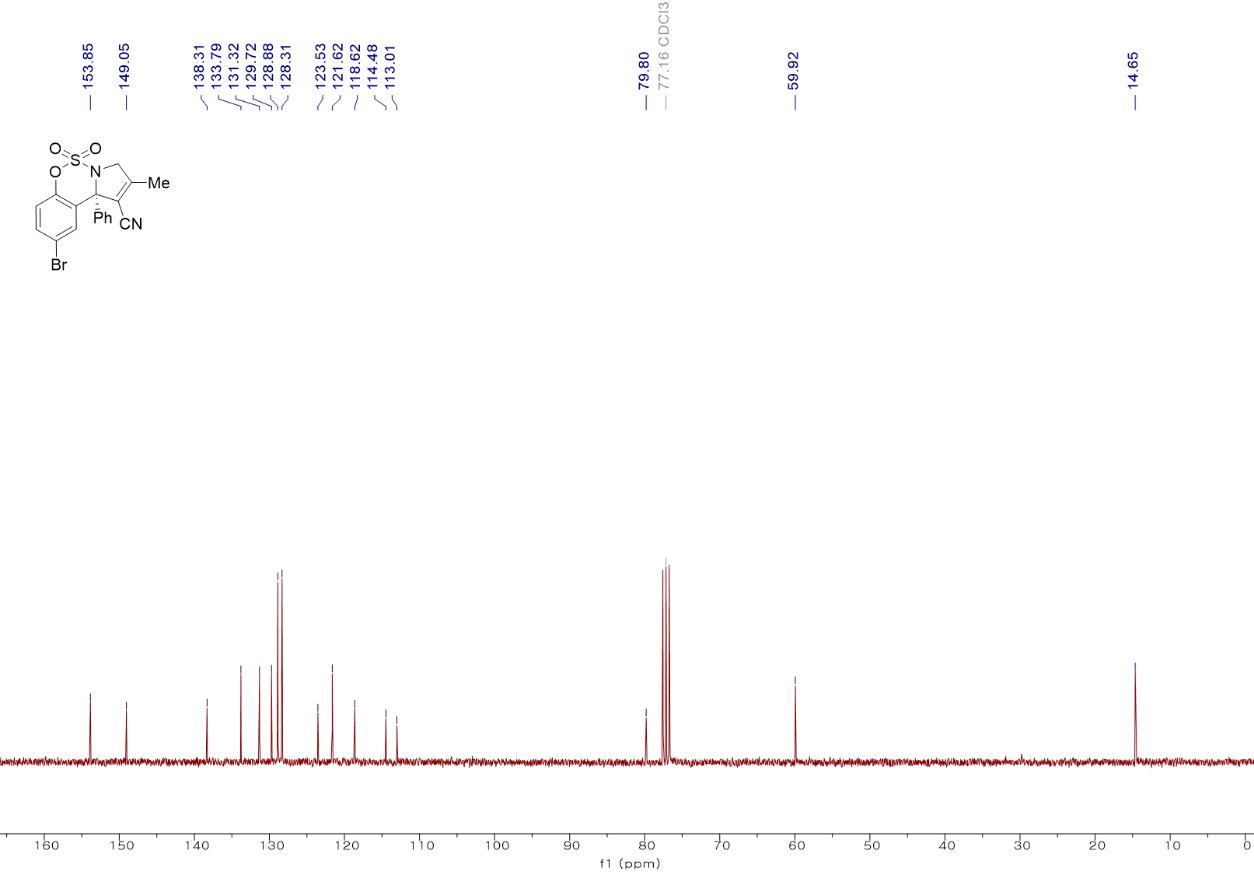


^13^C NMR spectrum of **4l**


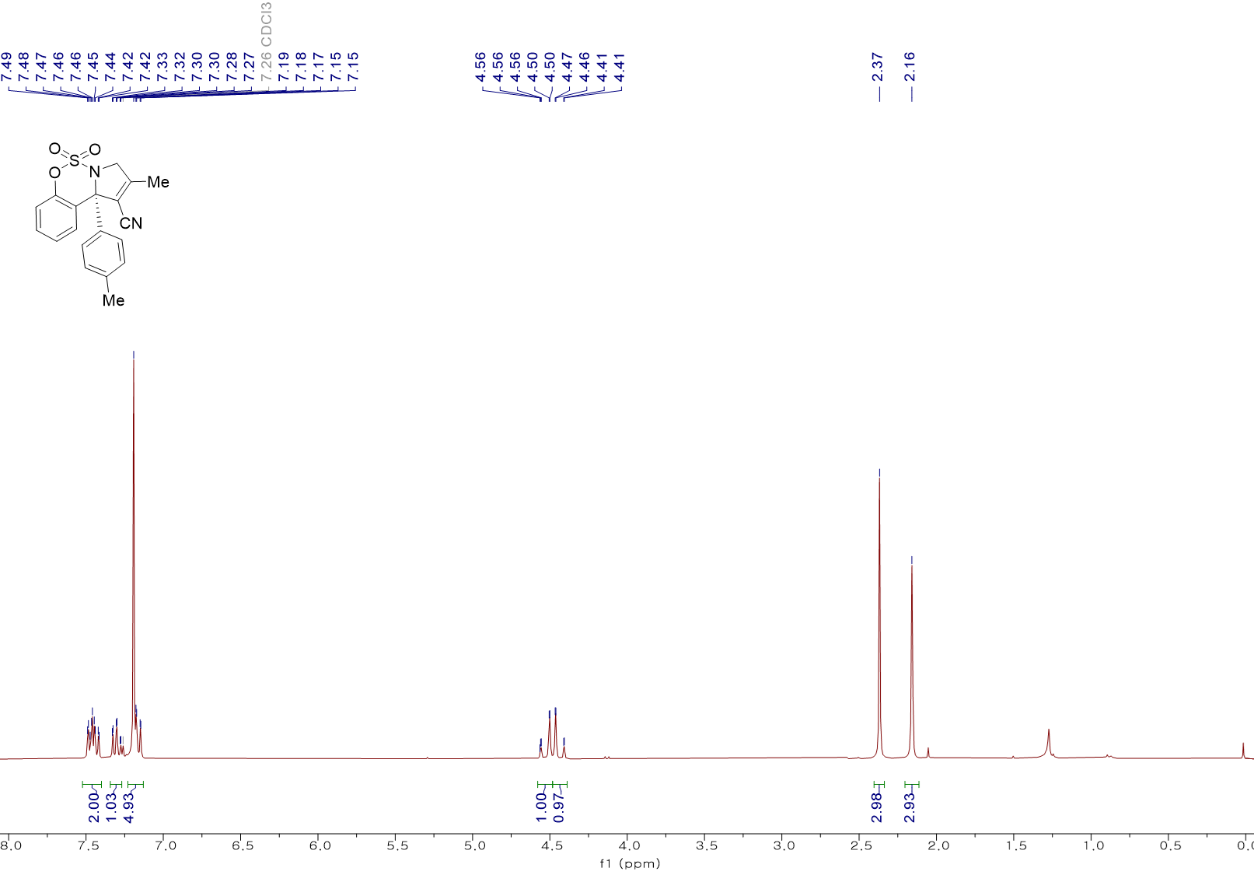


^1^H NMR spectrum of **4m**


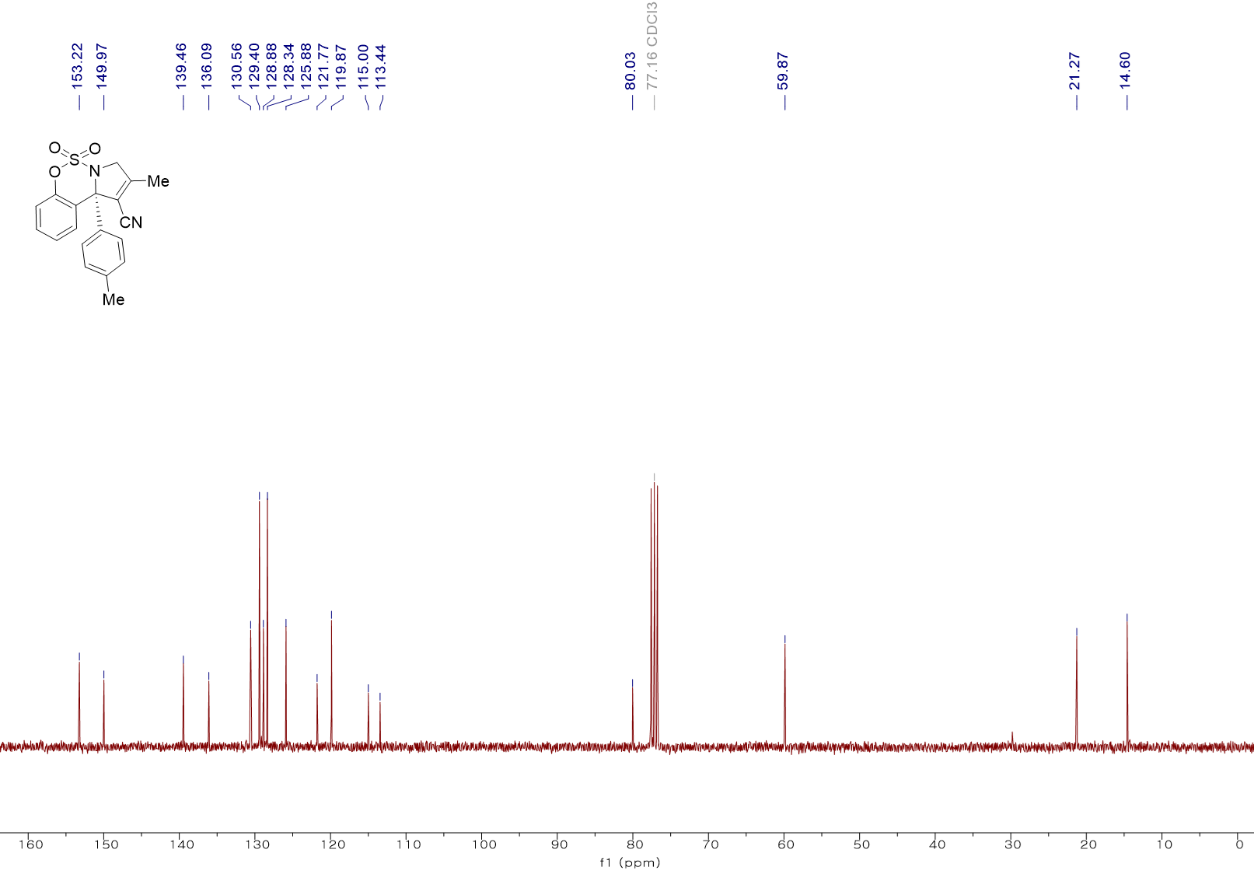


^13^C NMR spectrum of **4m**


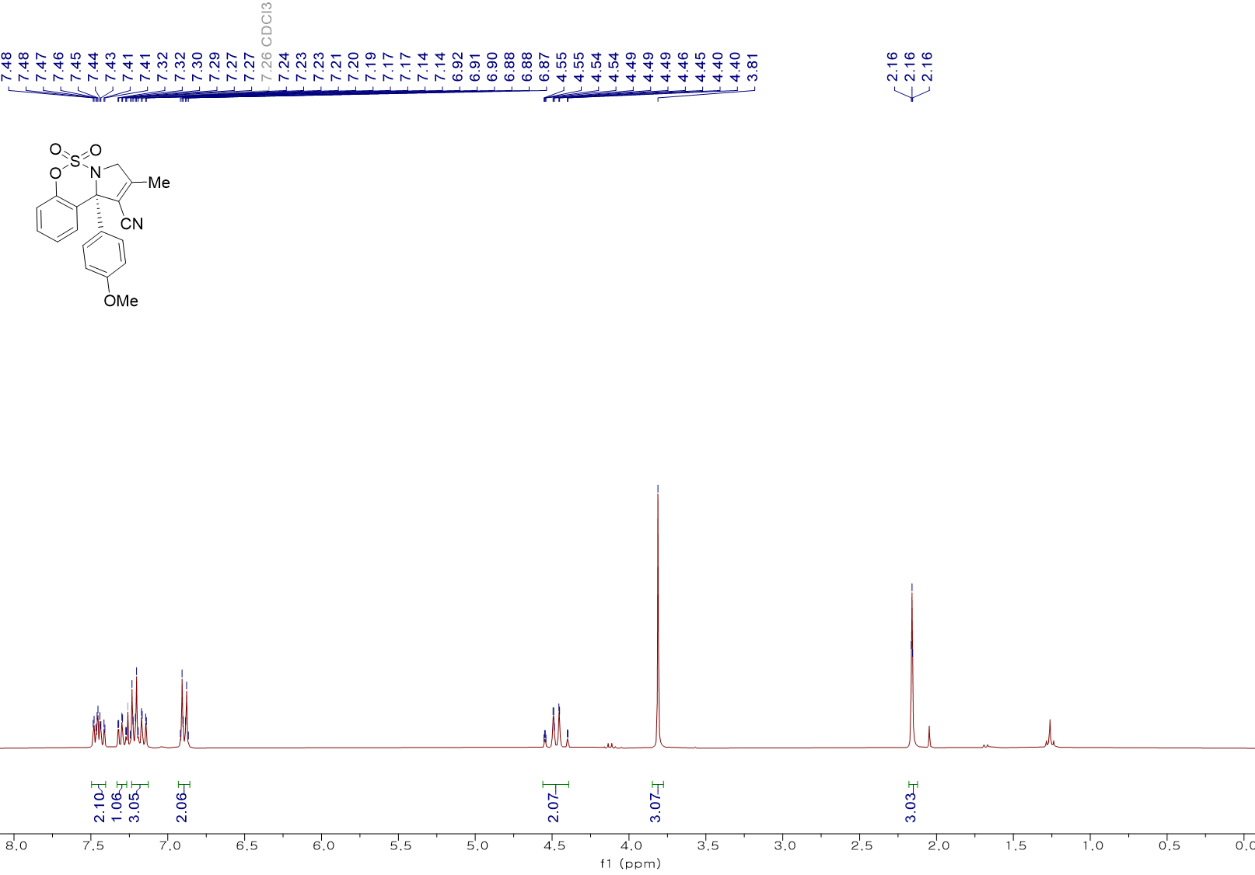


^1^H NMR spectrum of **4n**


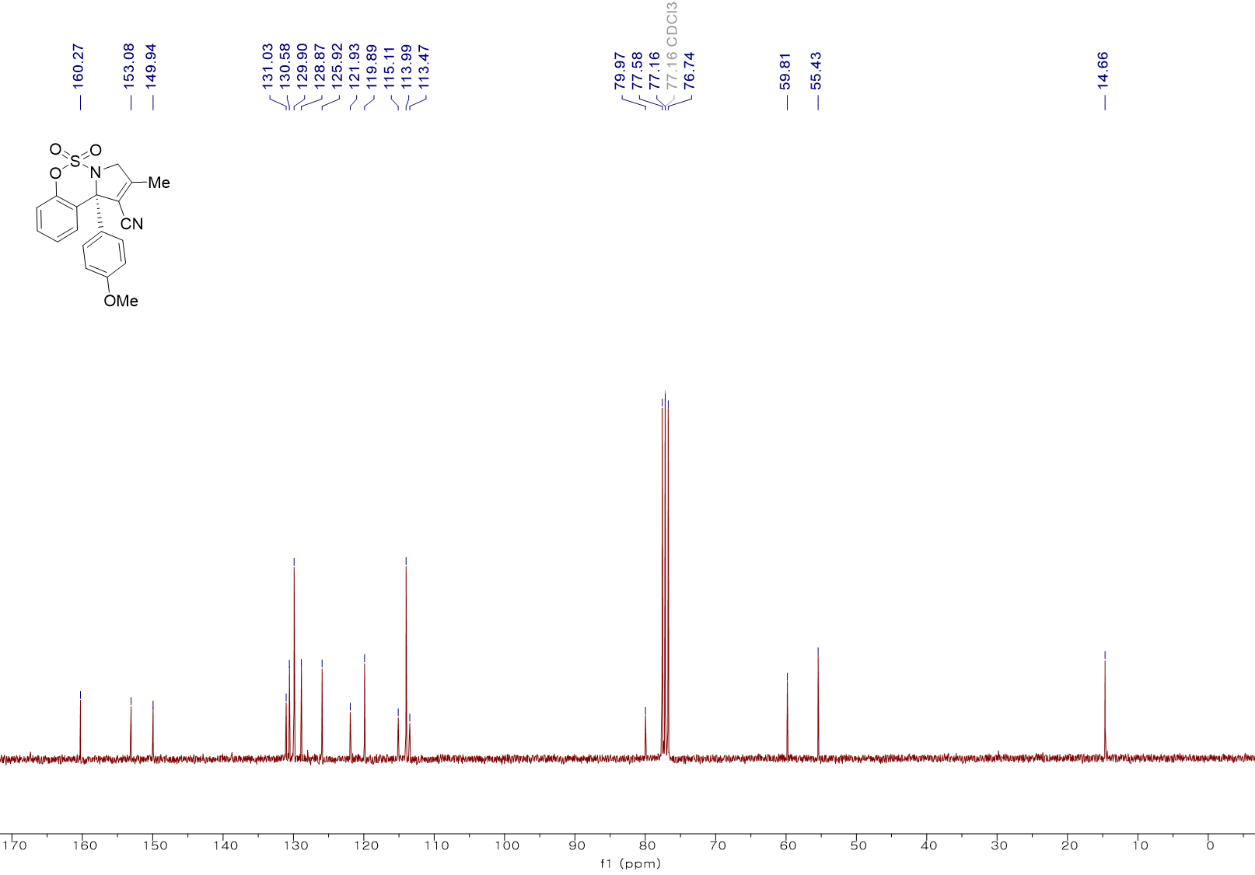


^13^C NMR spectrum of **4n**


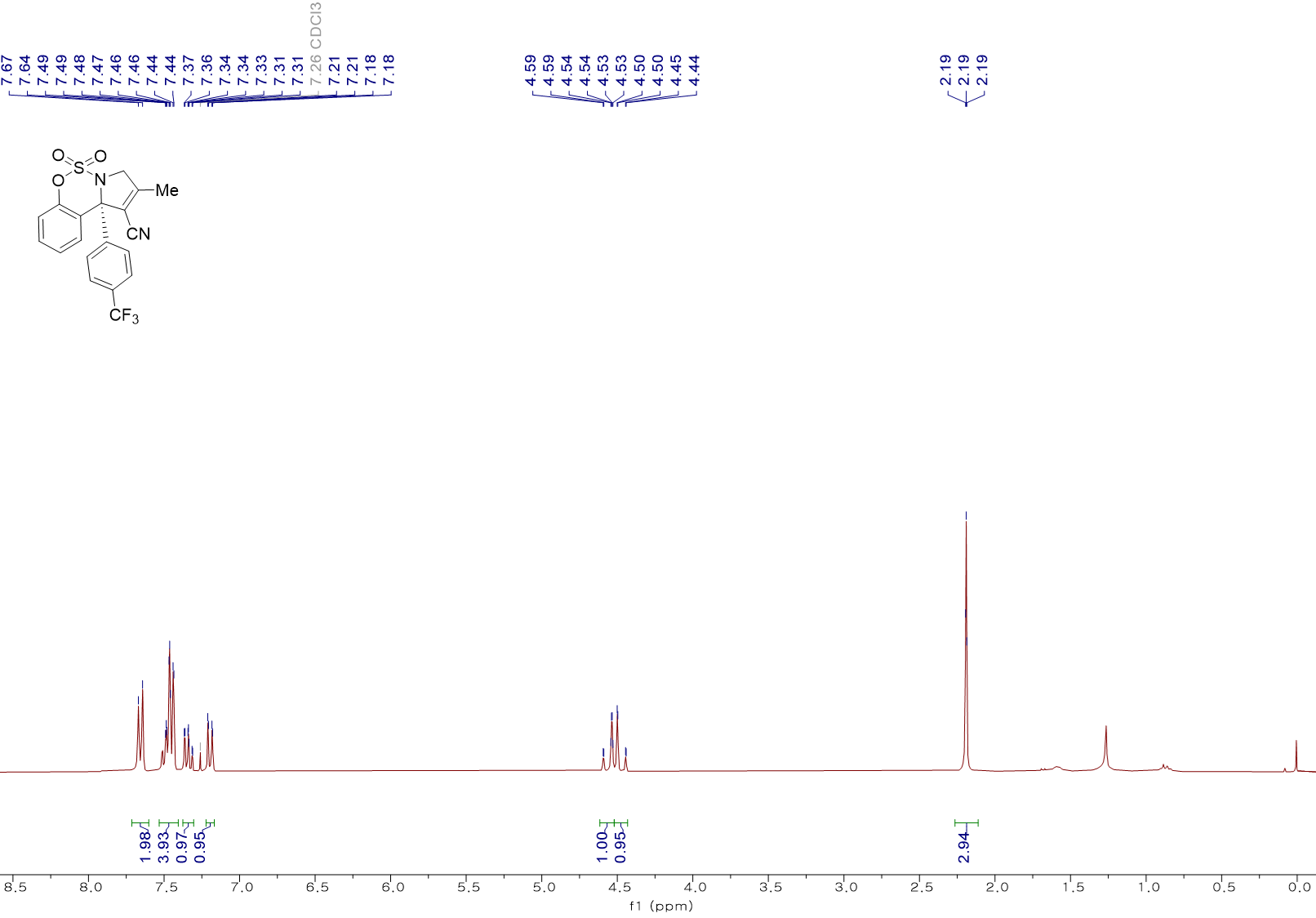


^1^H NMR spectrum of **4o**


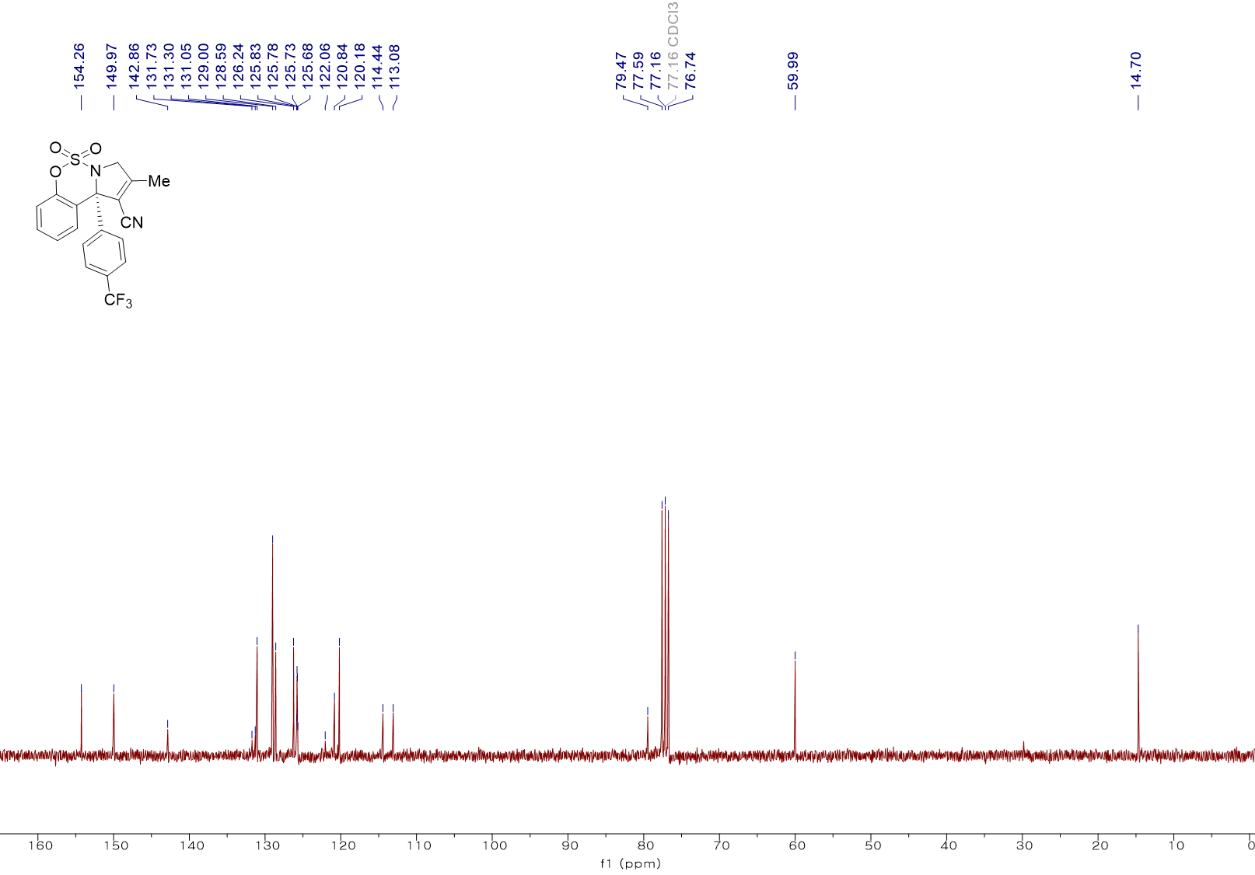


^13^C NMR spectrum of **4o**


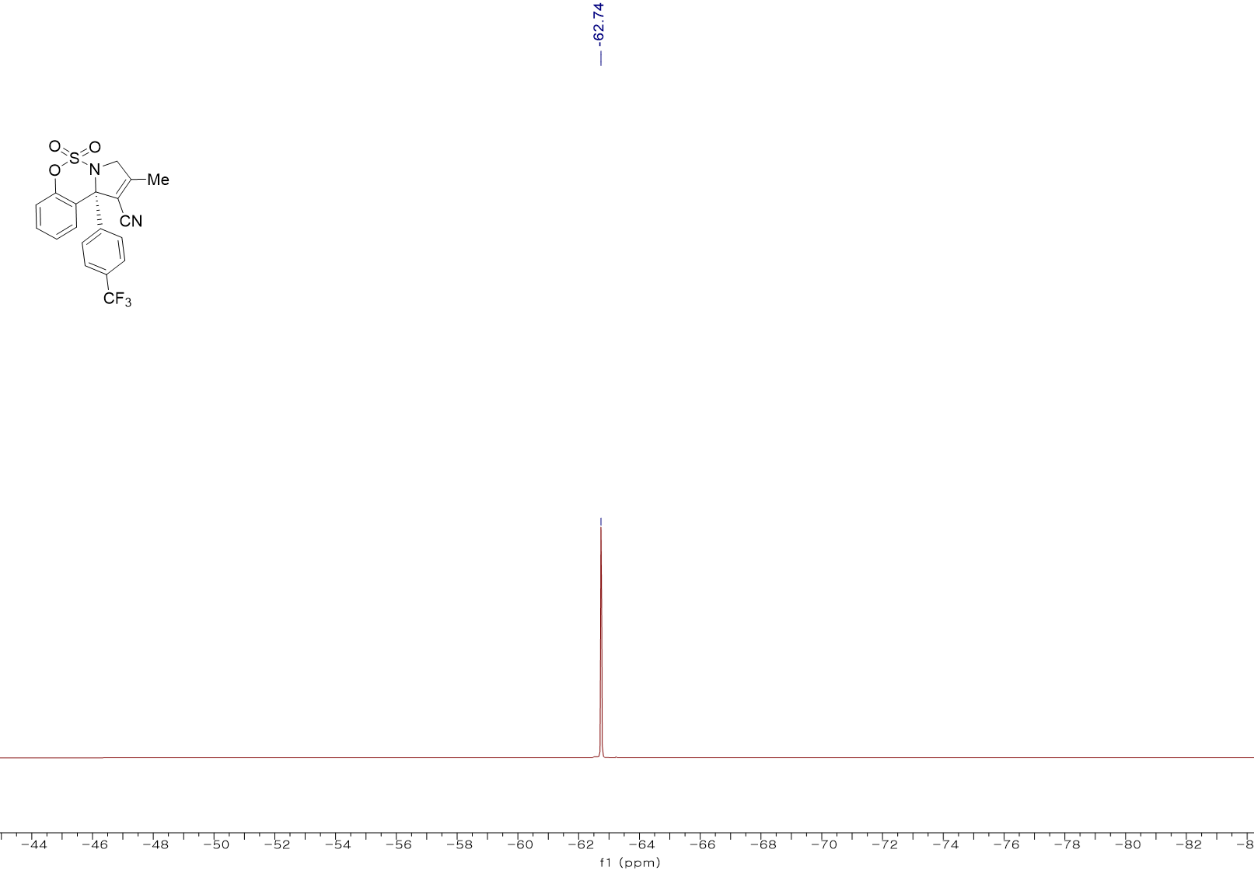


^19^F NMR spectrum of **4o**


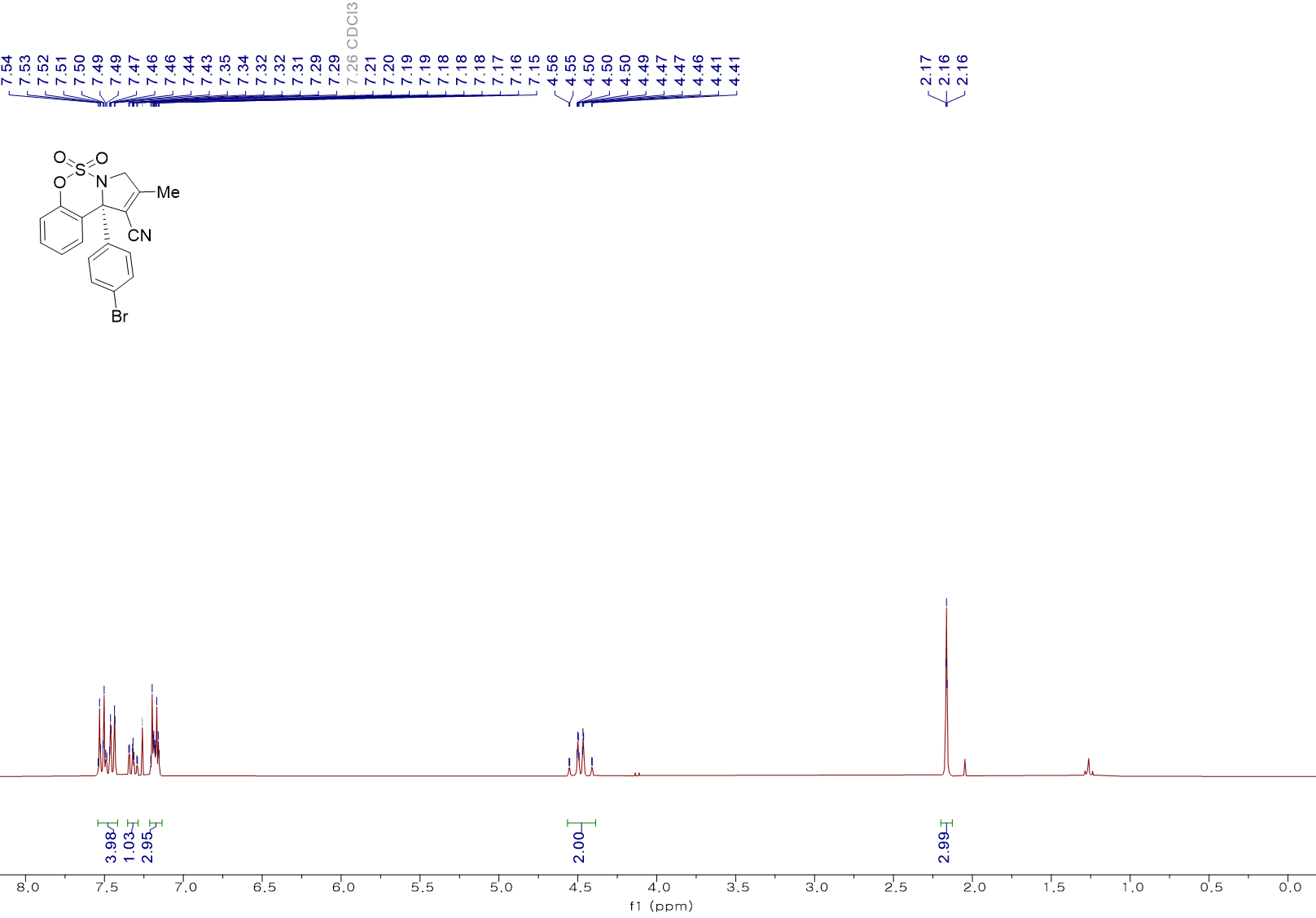


^1^H NMR spectrum of **4p**


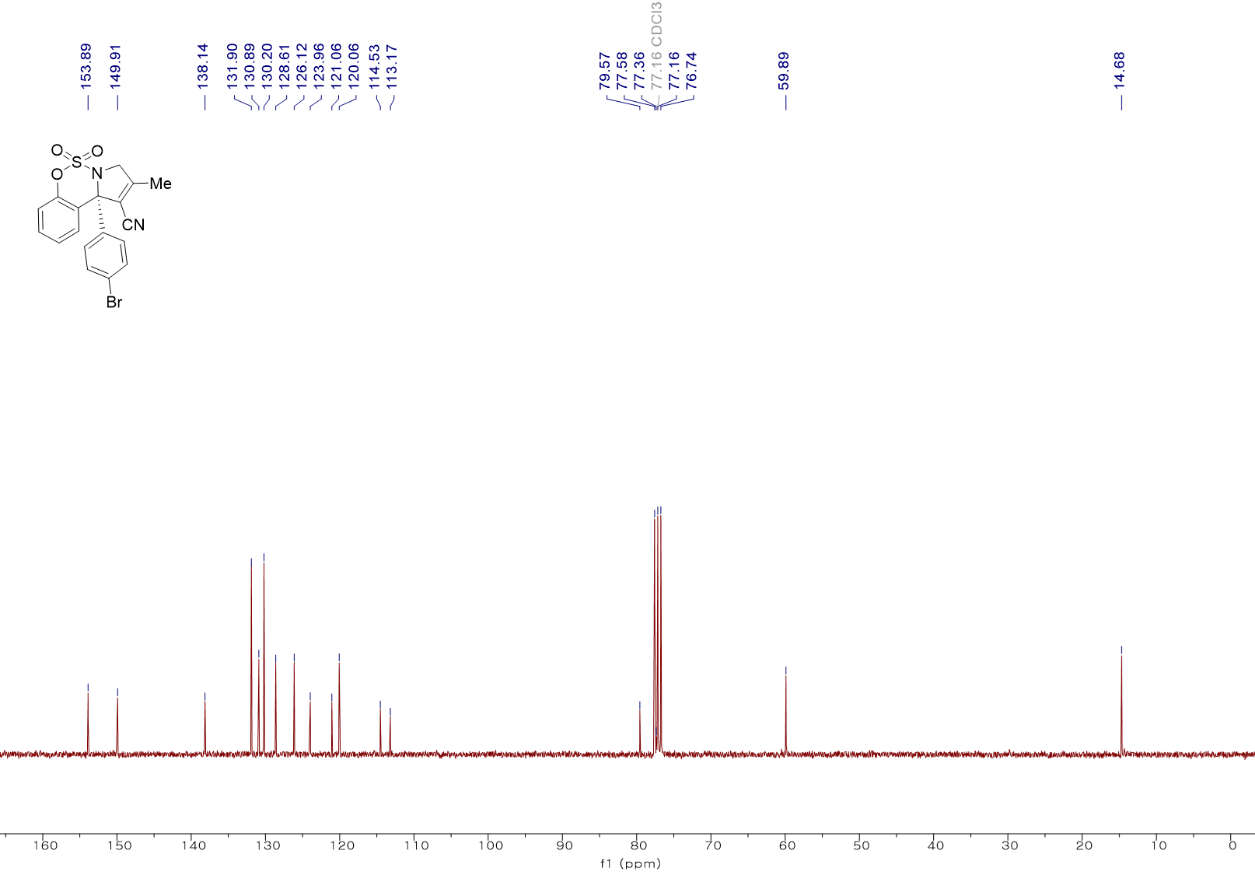


^13^C NMR spectrum of **4p**


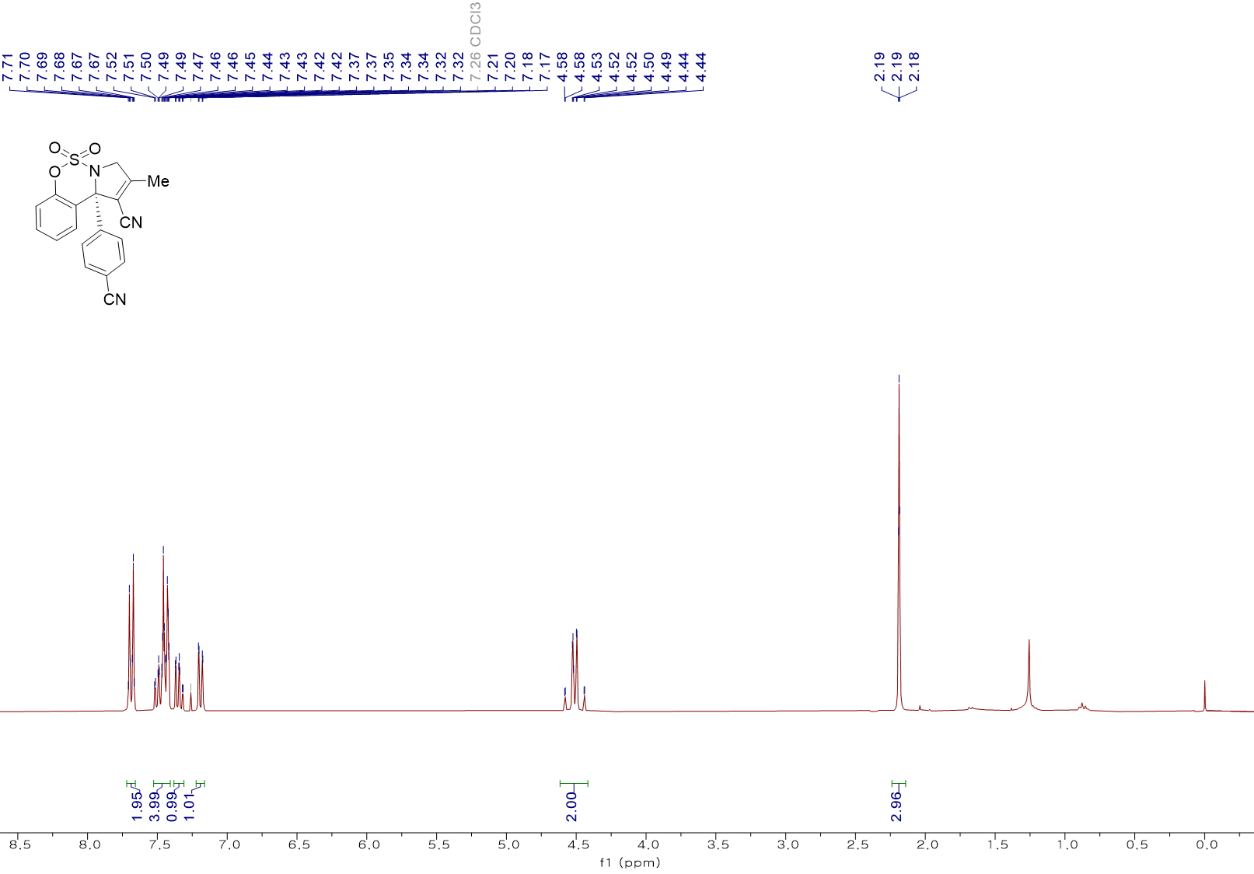


^1^H NMR spectrum of **4q**


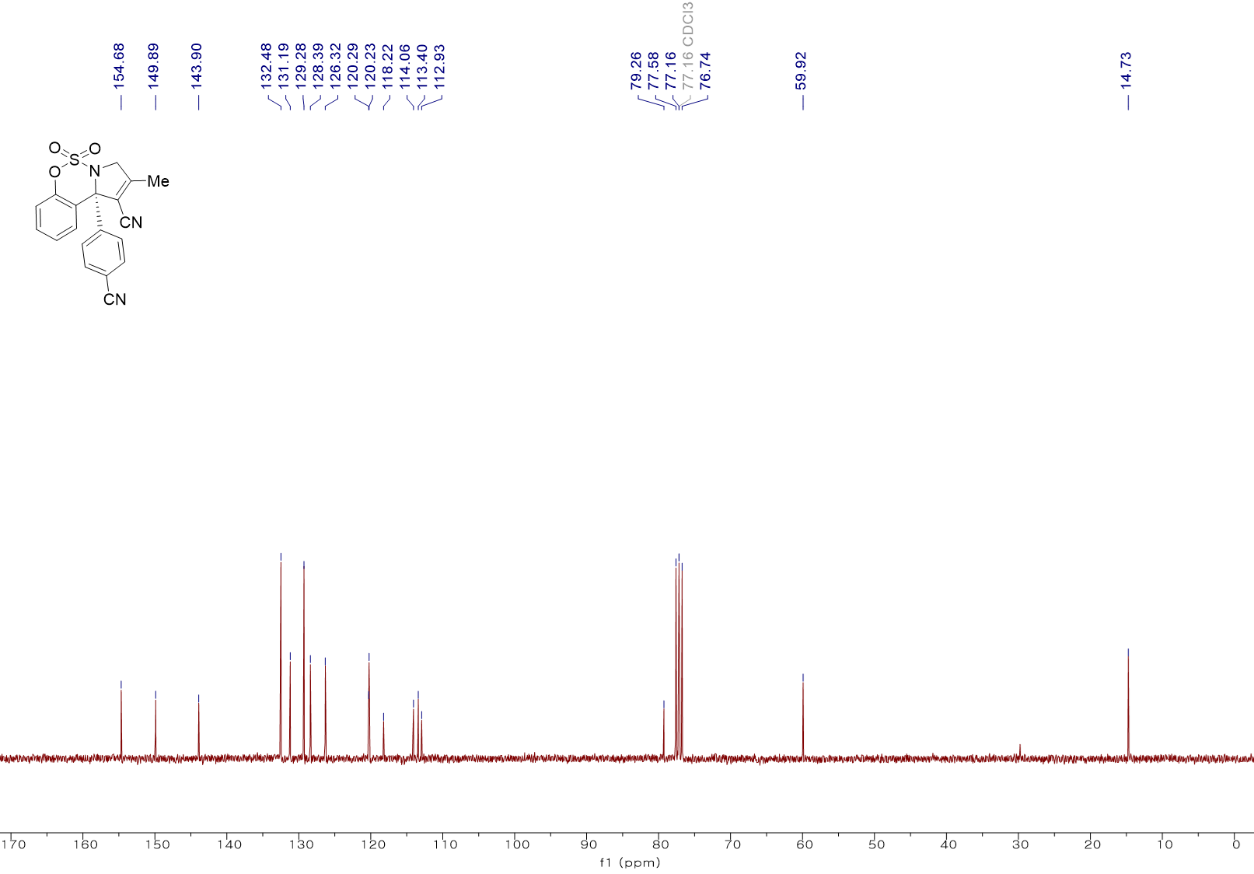


^13^C NMR spectrum of **4q**


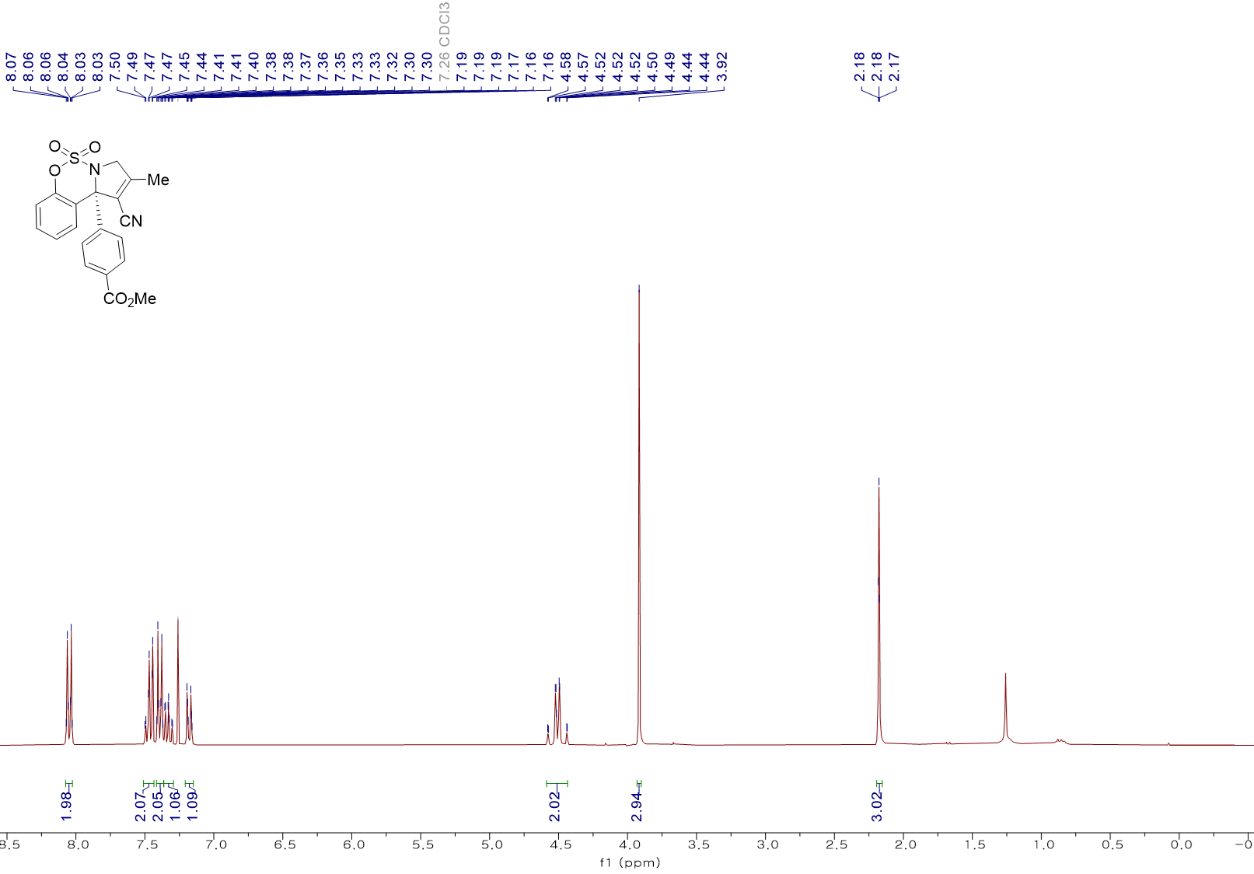


^1^H NMR spectrum of **4r**


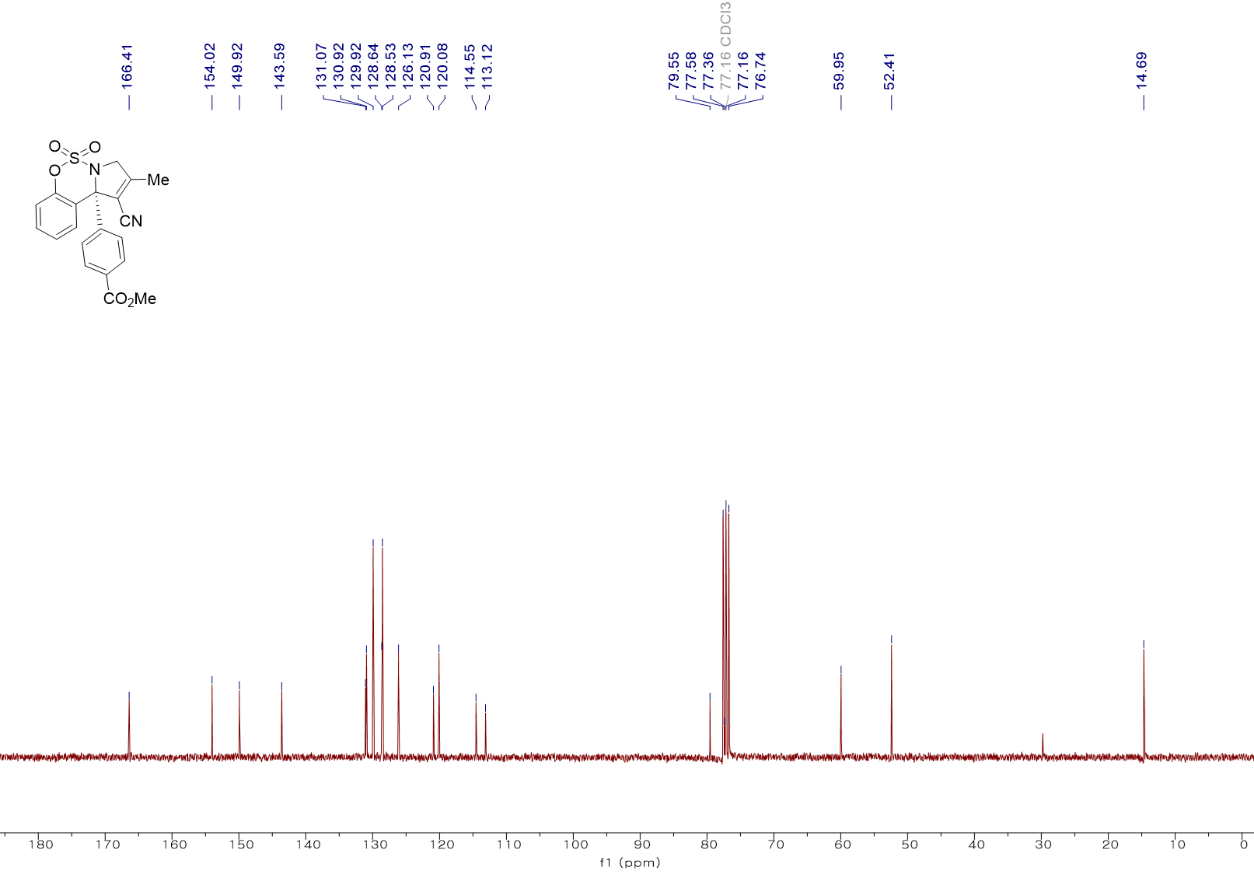


^13^C NMR spectrum of **4r**


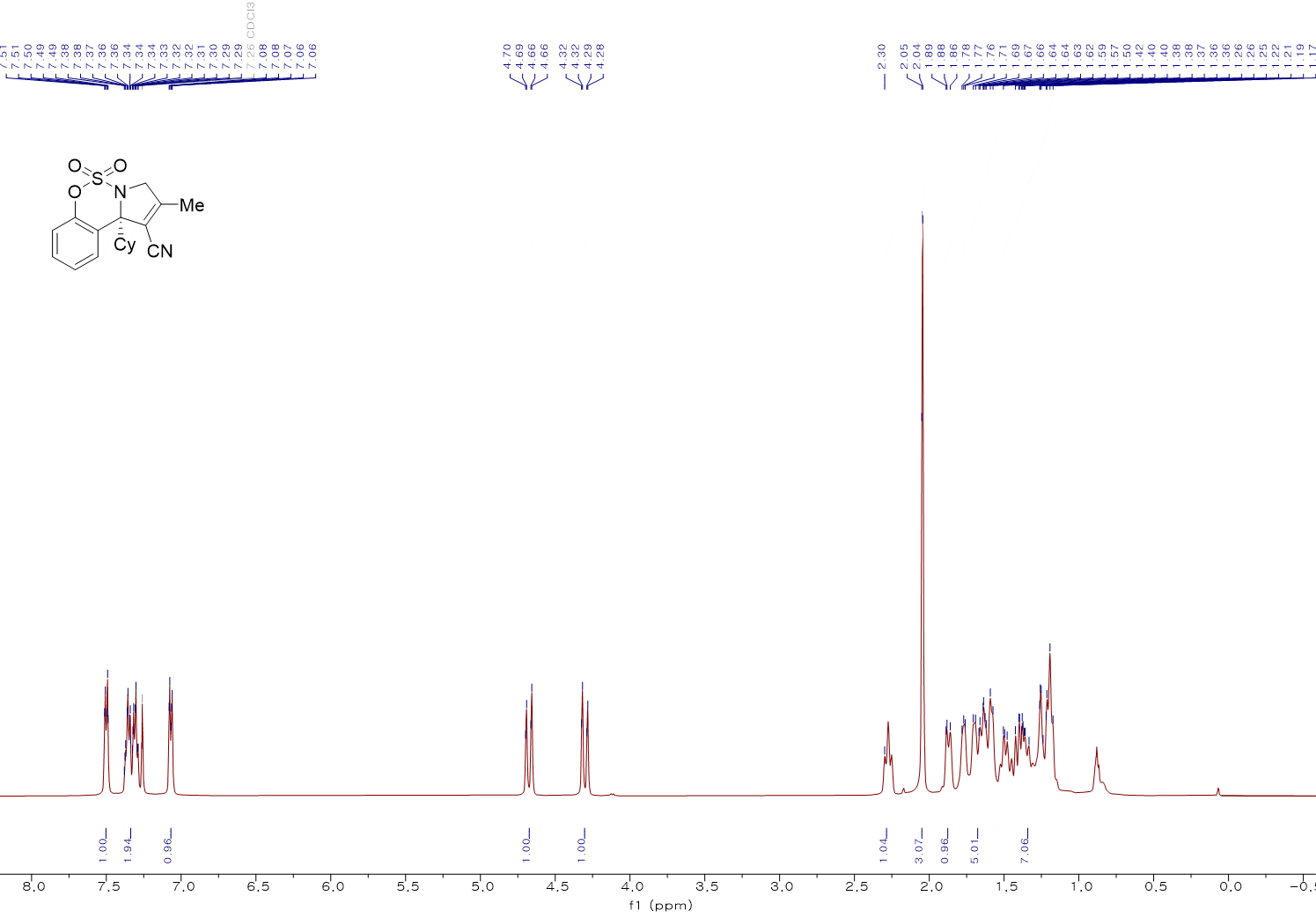


^1^H NMR spectrum of **4s**


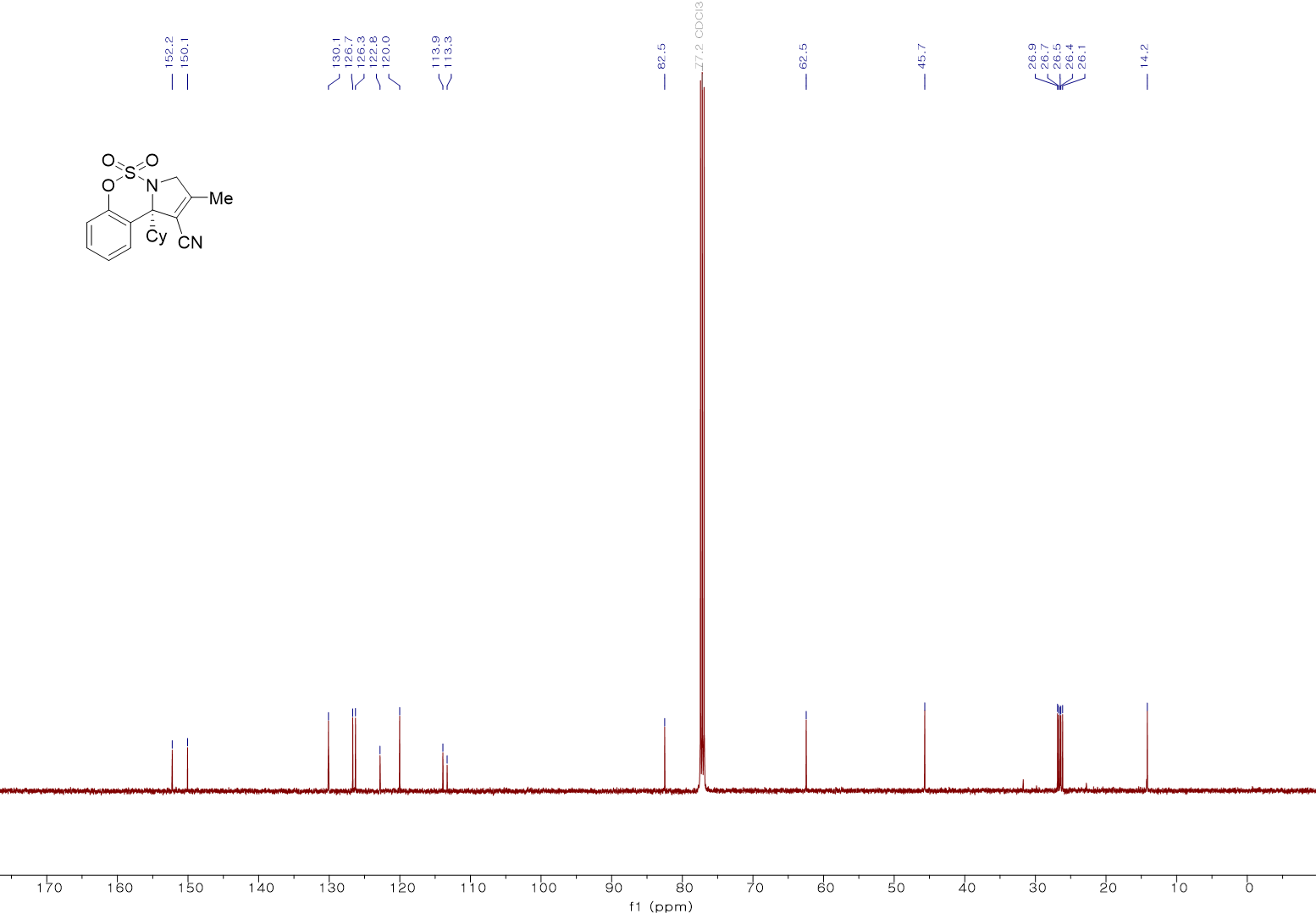


^13^C NMR spectrum of **4s**

**
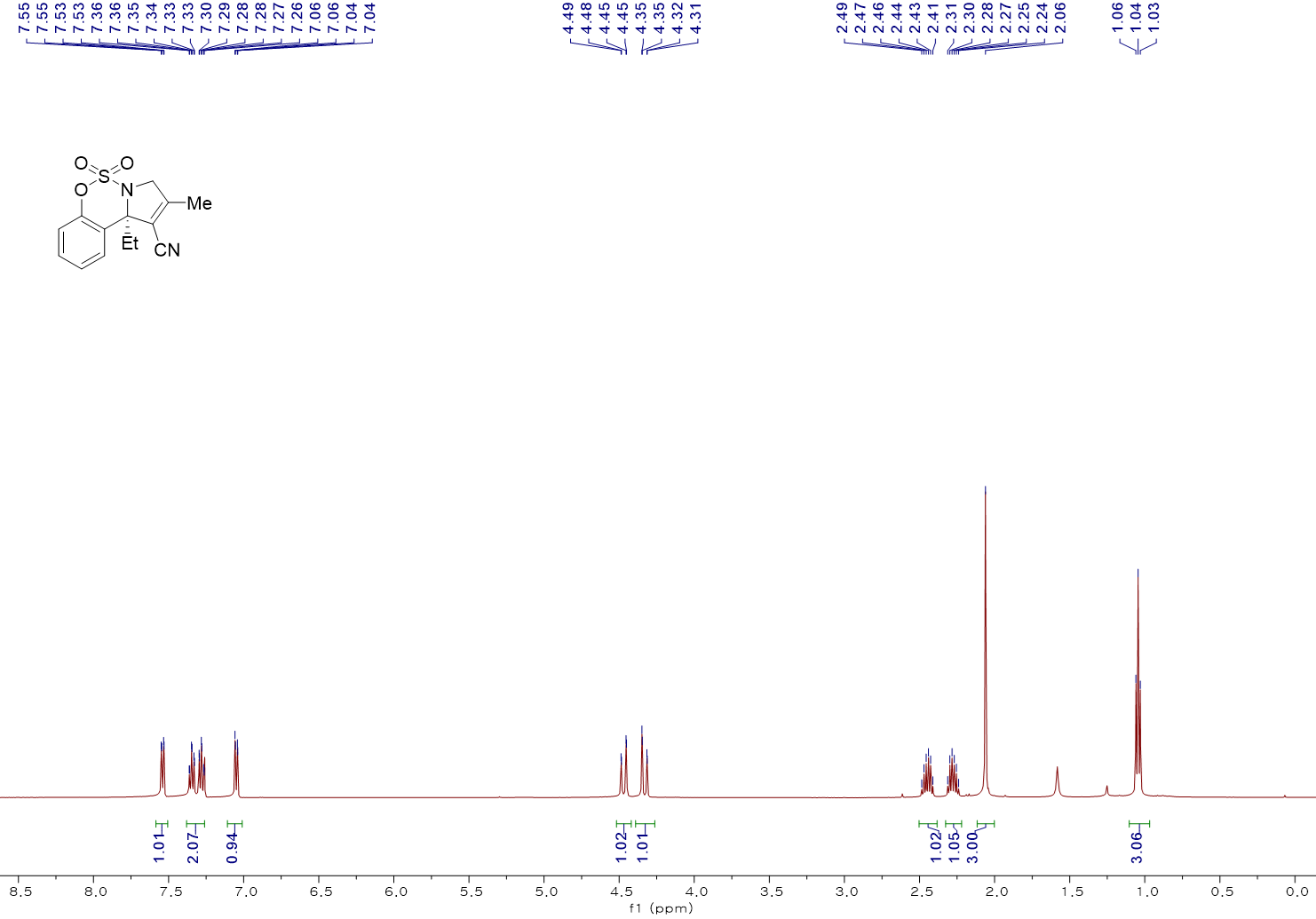
**

^1^H NMR spectrum of **4t**

**
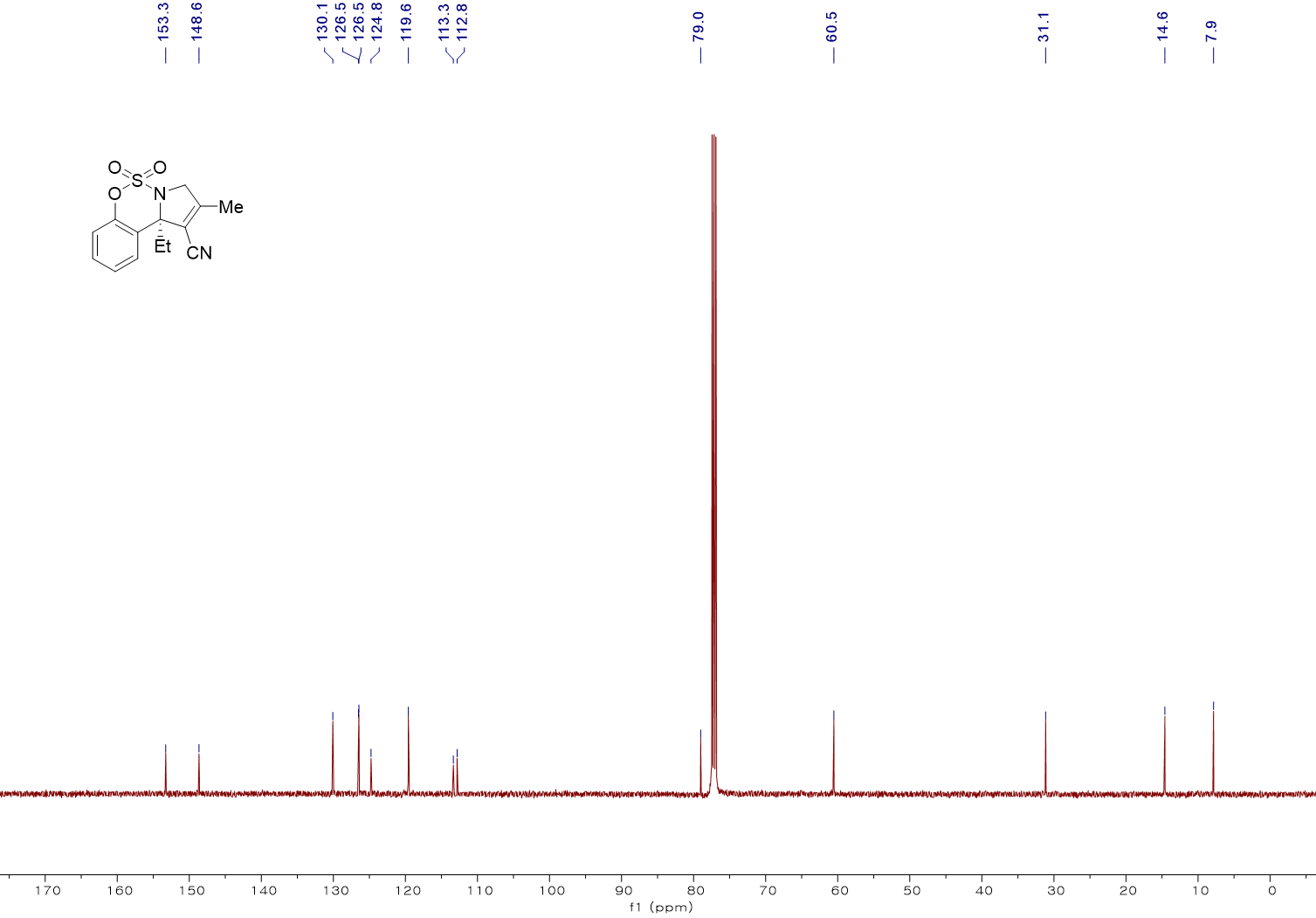
**

^13^C NMR spectrum of **4t**


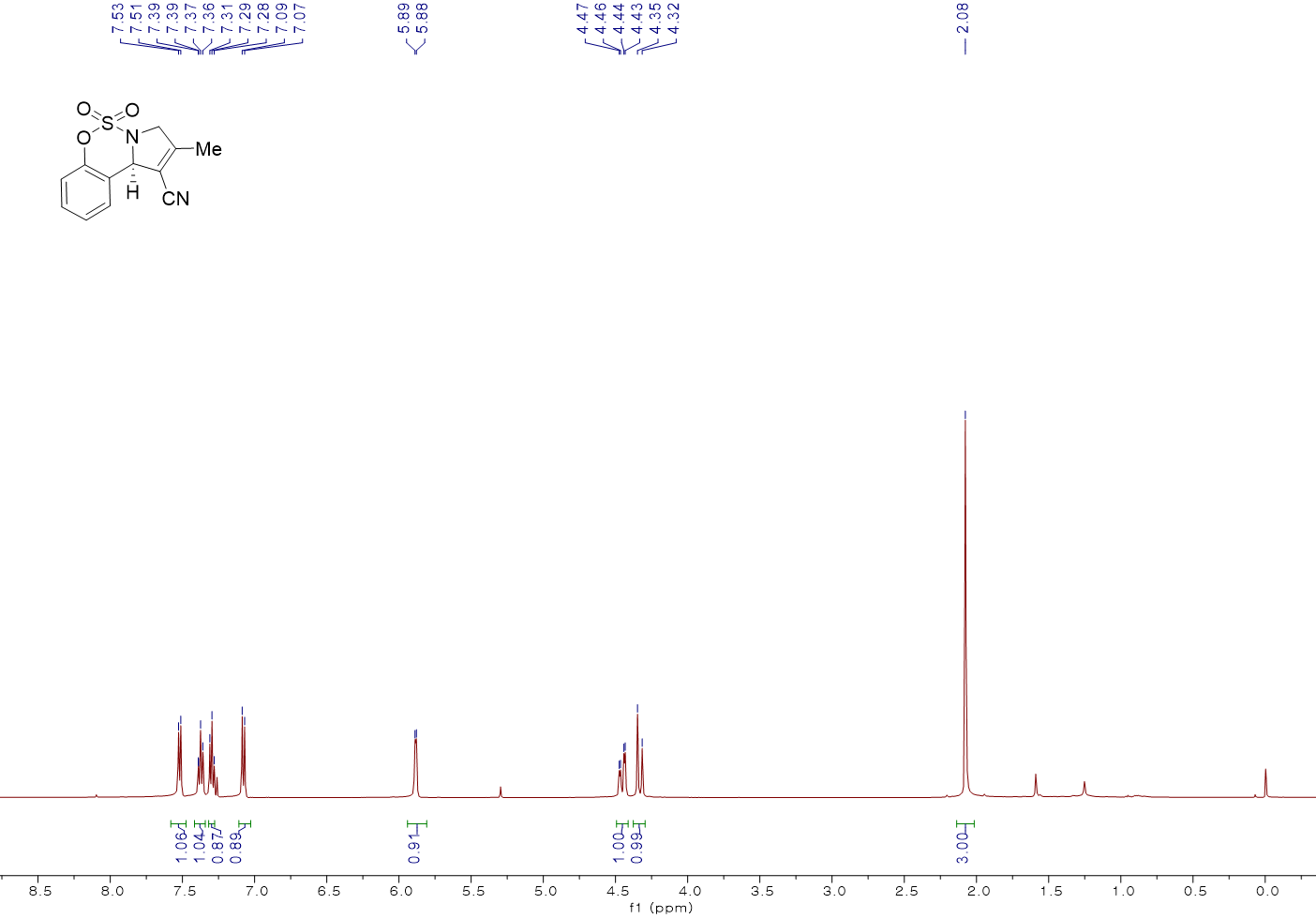


^1^H NMR spectrum of **4u**


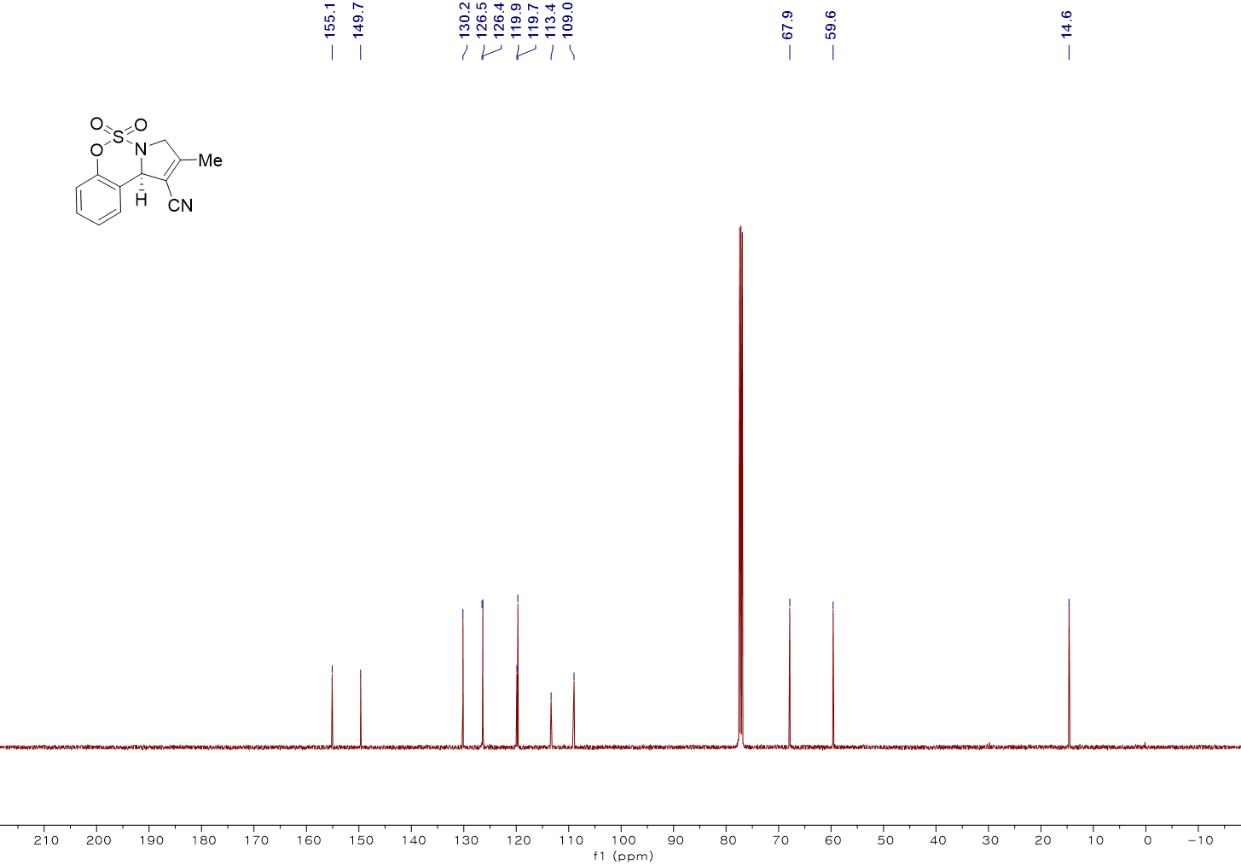


^13^C NMR spectrum of **4u**


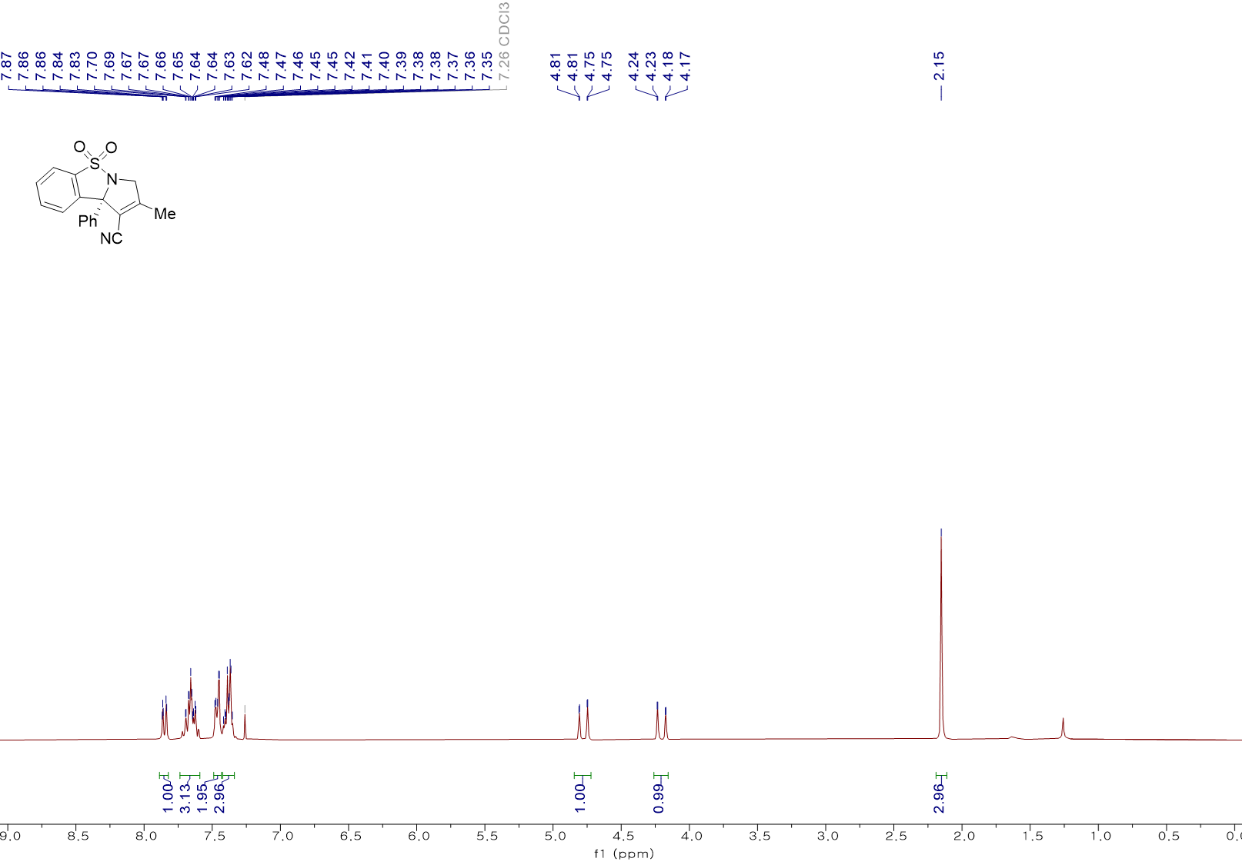


^1^H NMR spectrum of **4v**


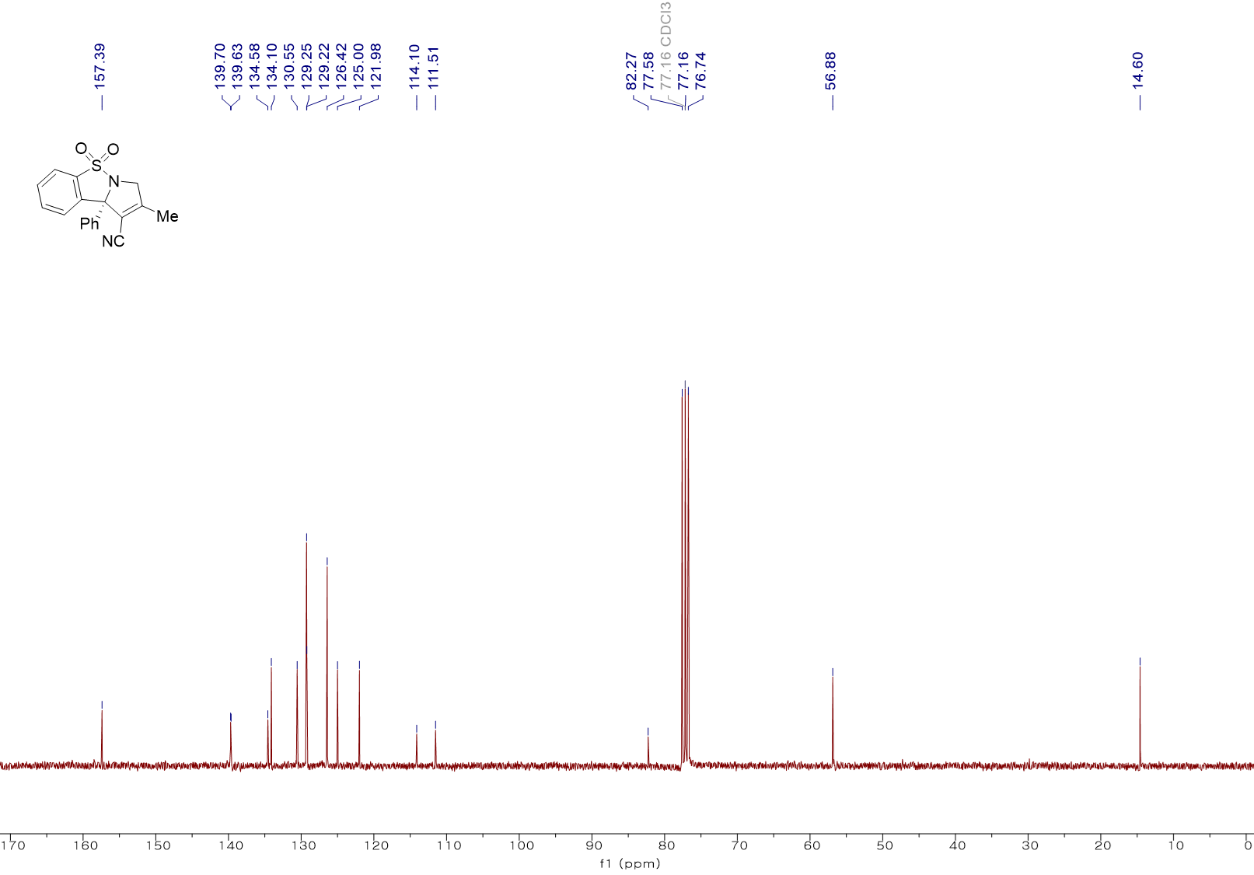


^13^C NMR spectrum of **4v**


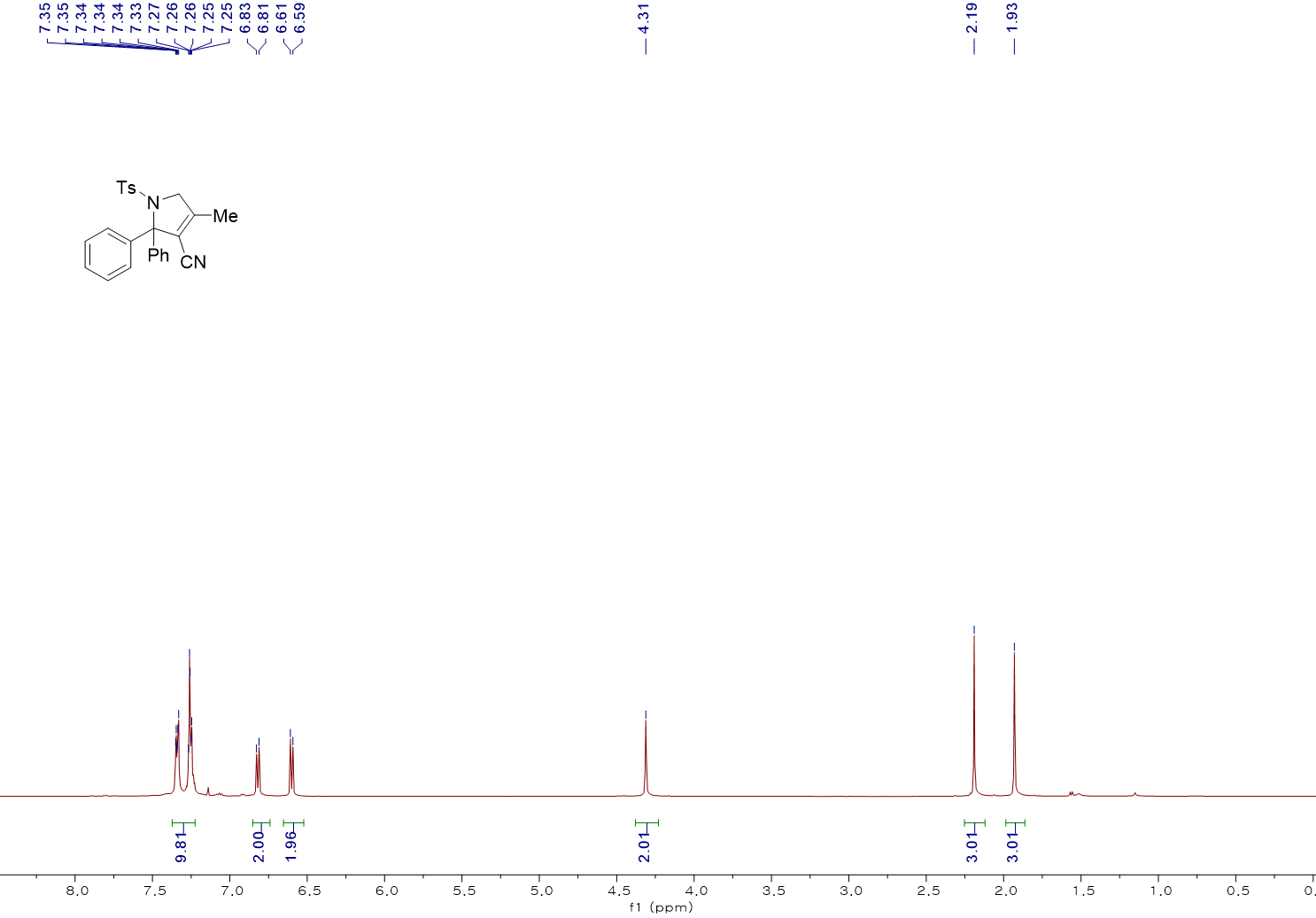


^1^H NMR spectrum of **4w**


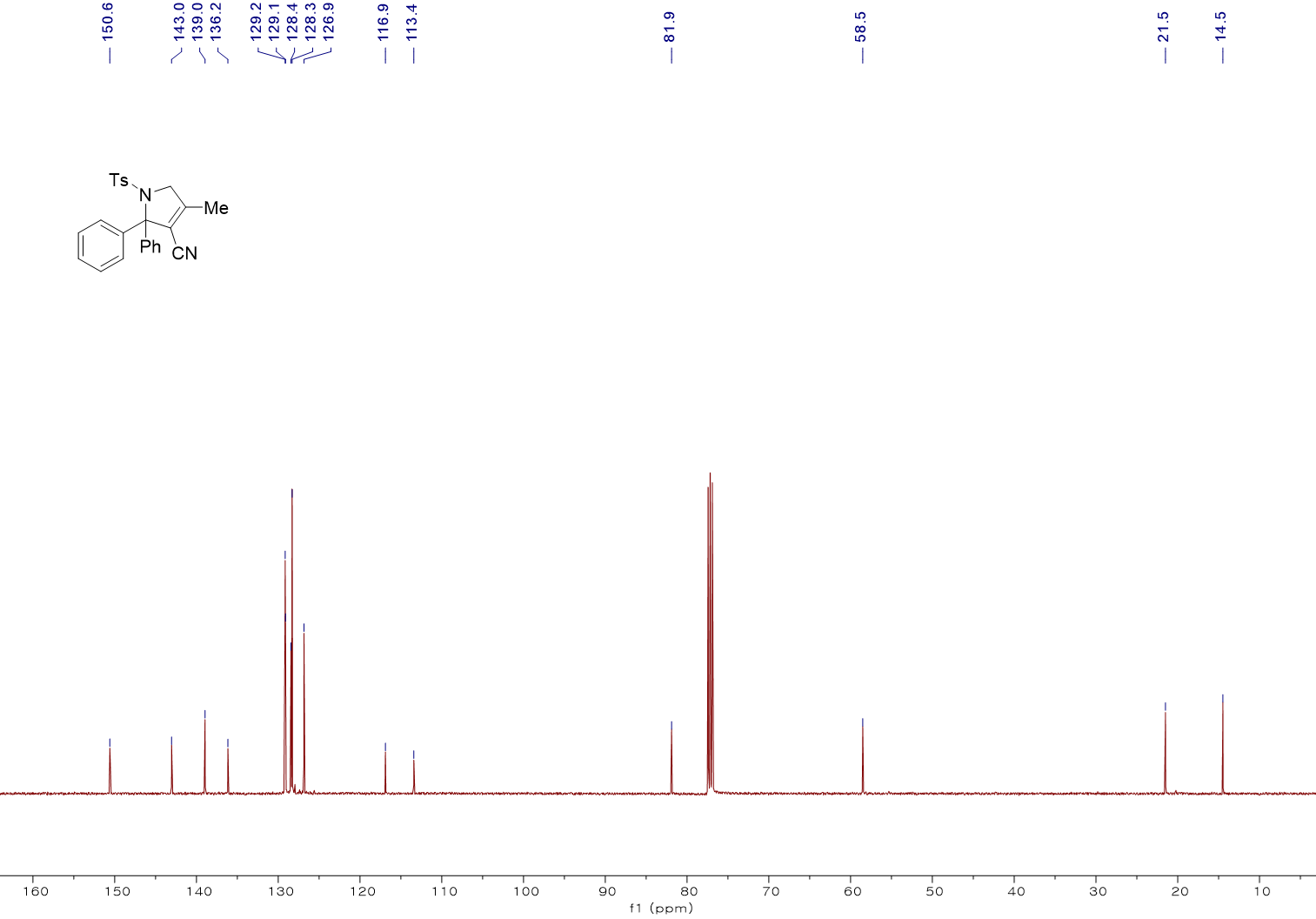


^13^C NMR spectrum of **4w**


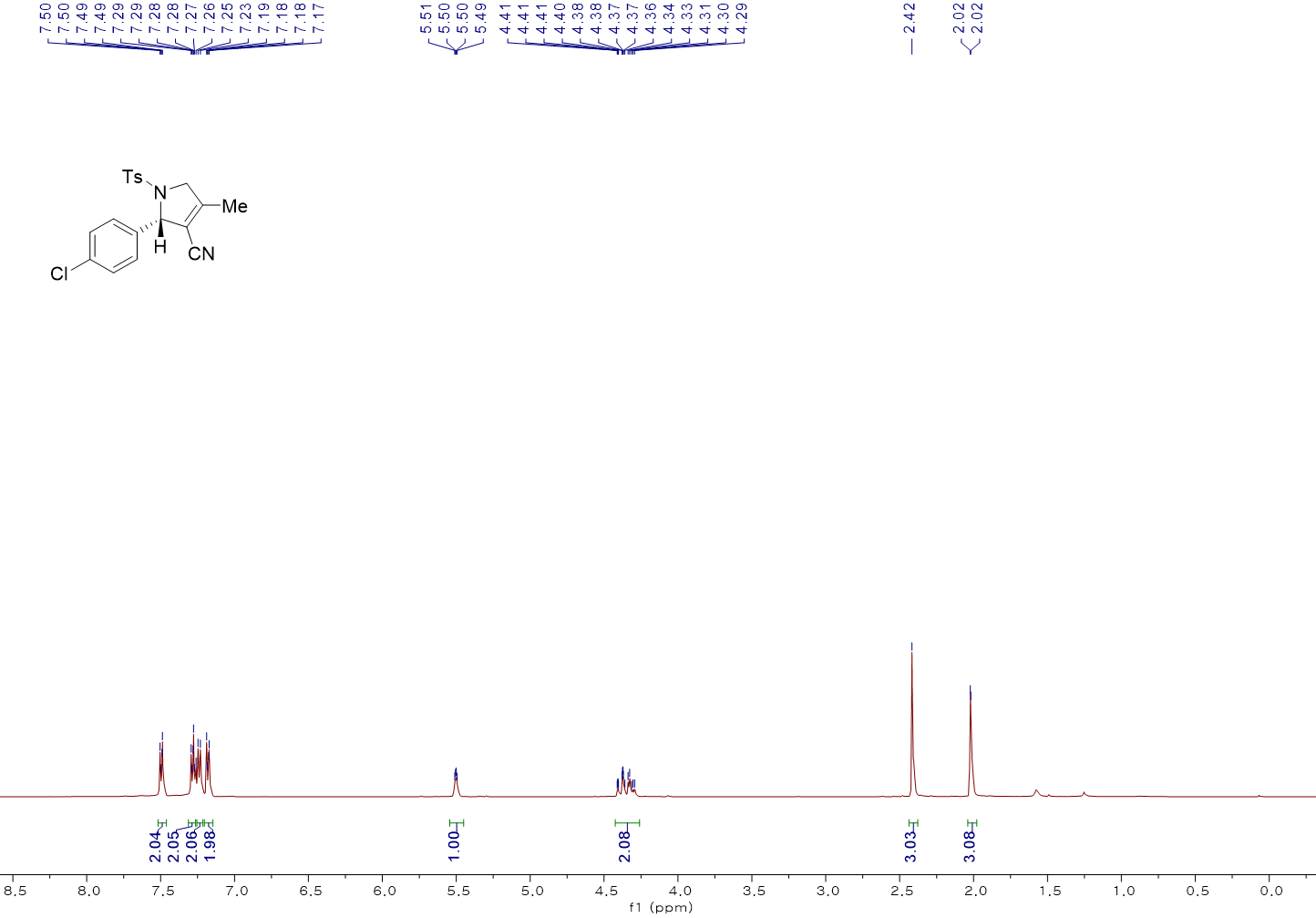


^1^H NMR spectrum of **4x**


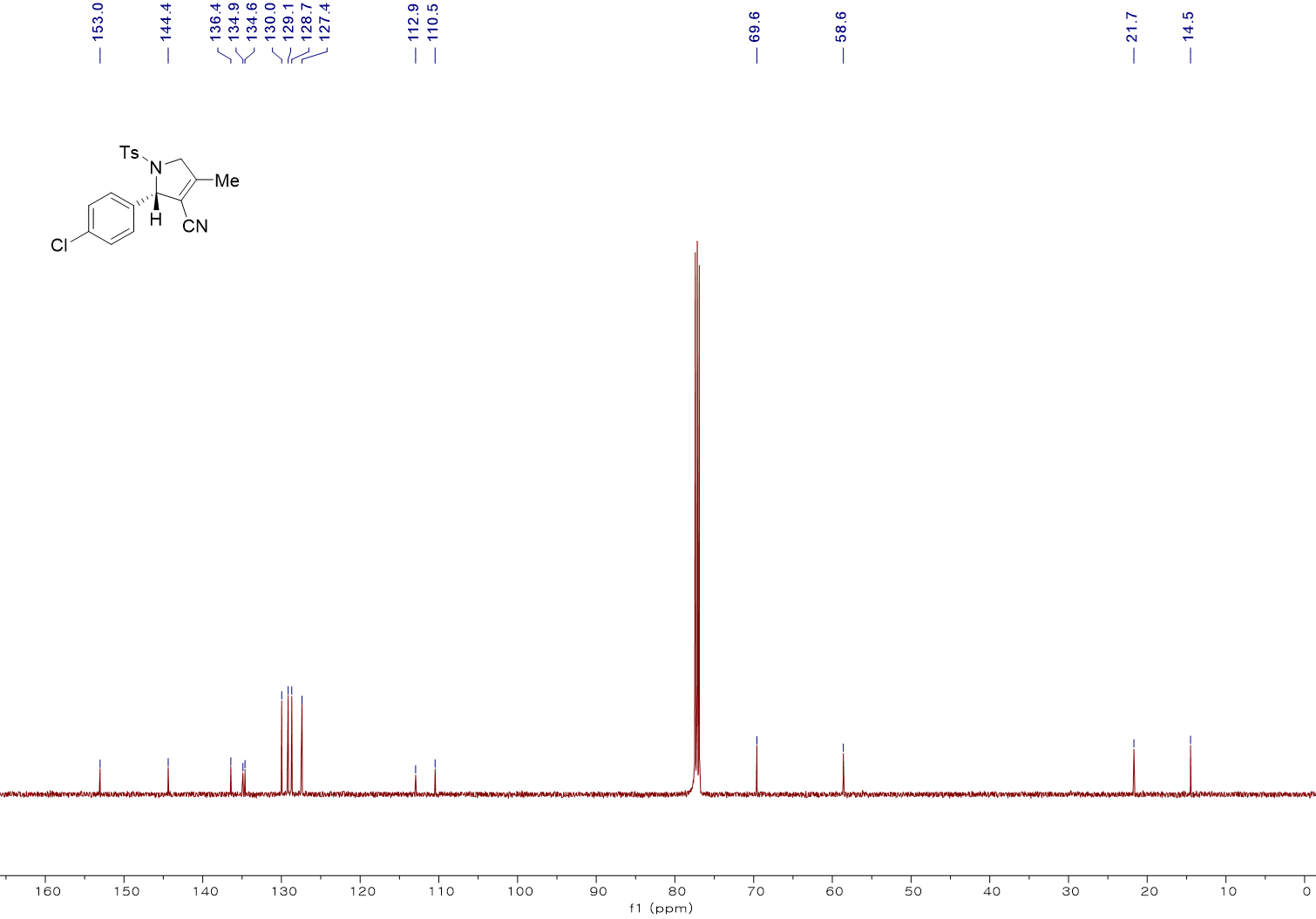


^13^C NMR spectrum of **4x**


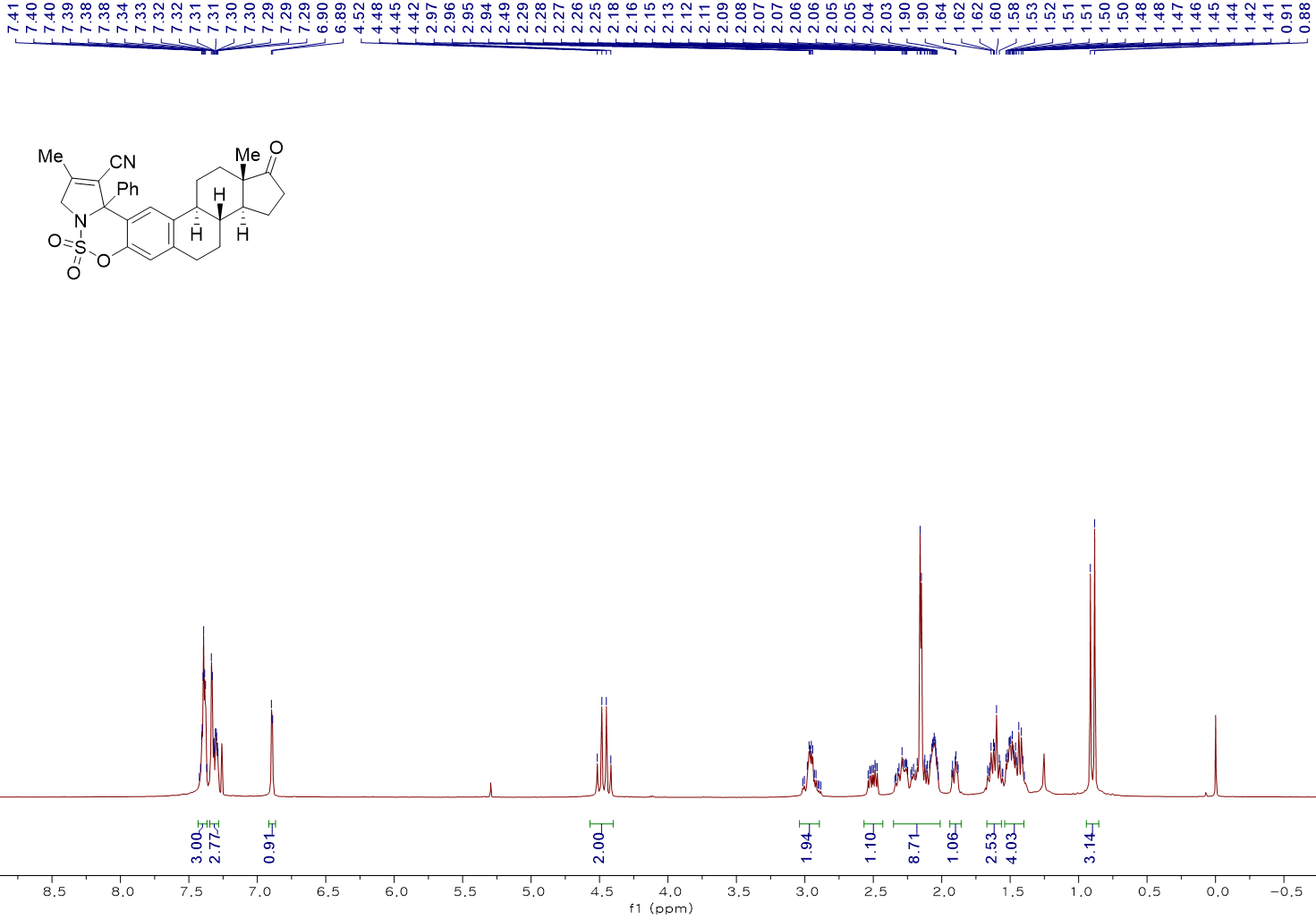


^1^H NMR spectrum of **4y**


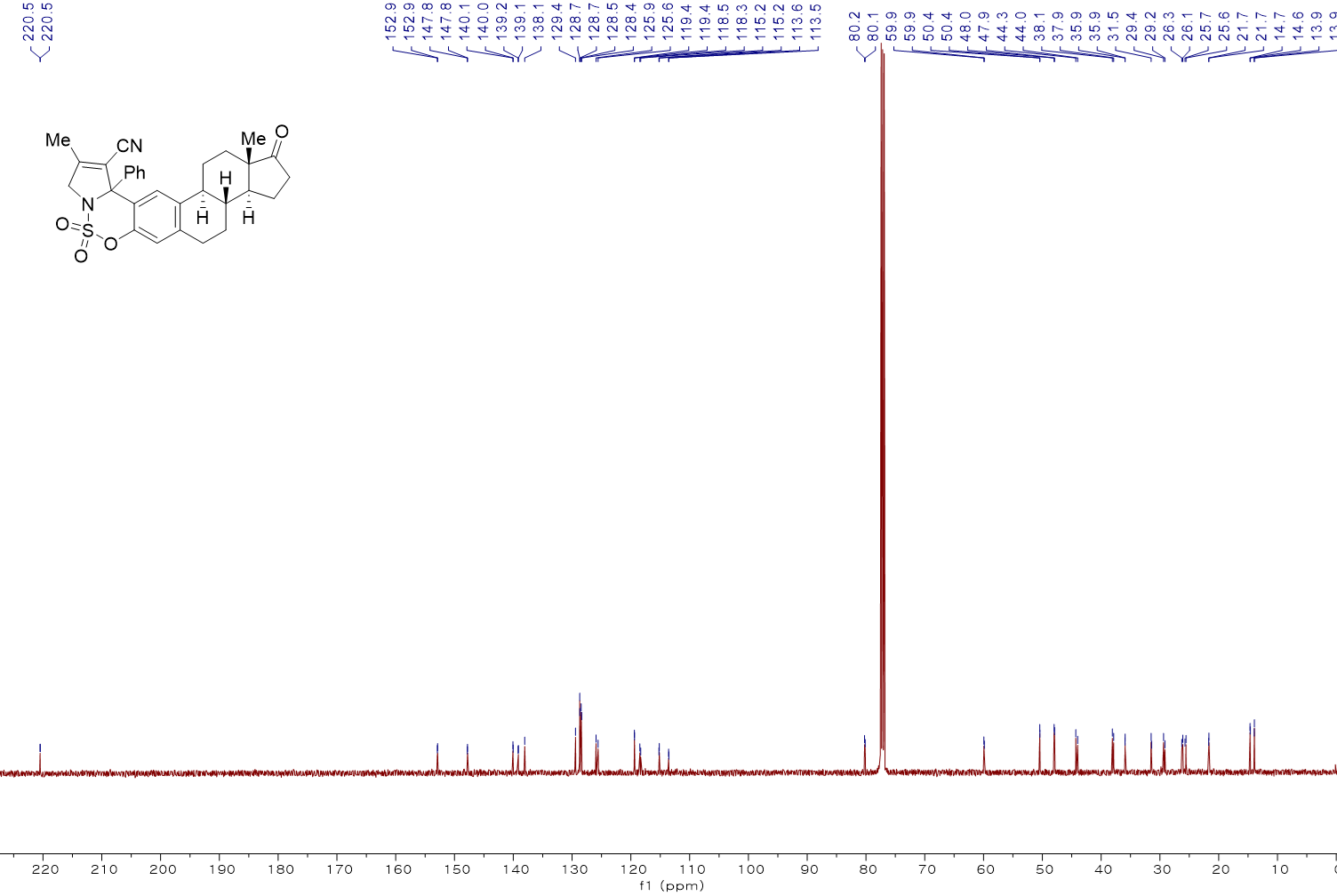


^13^C NMR spectrum of **4y**


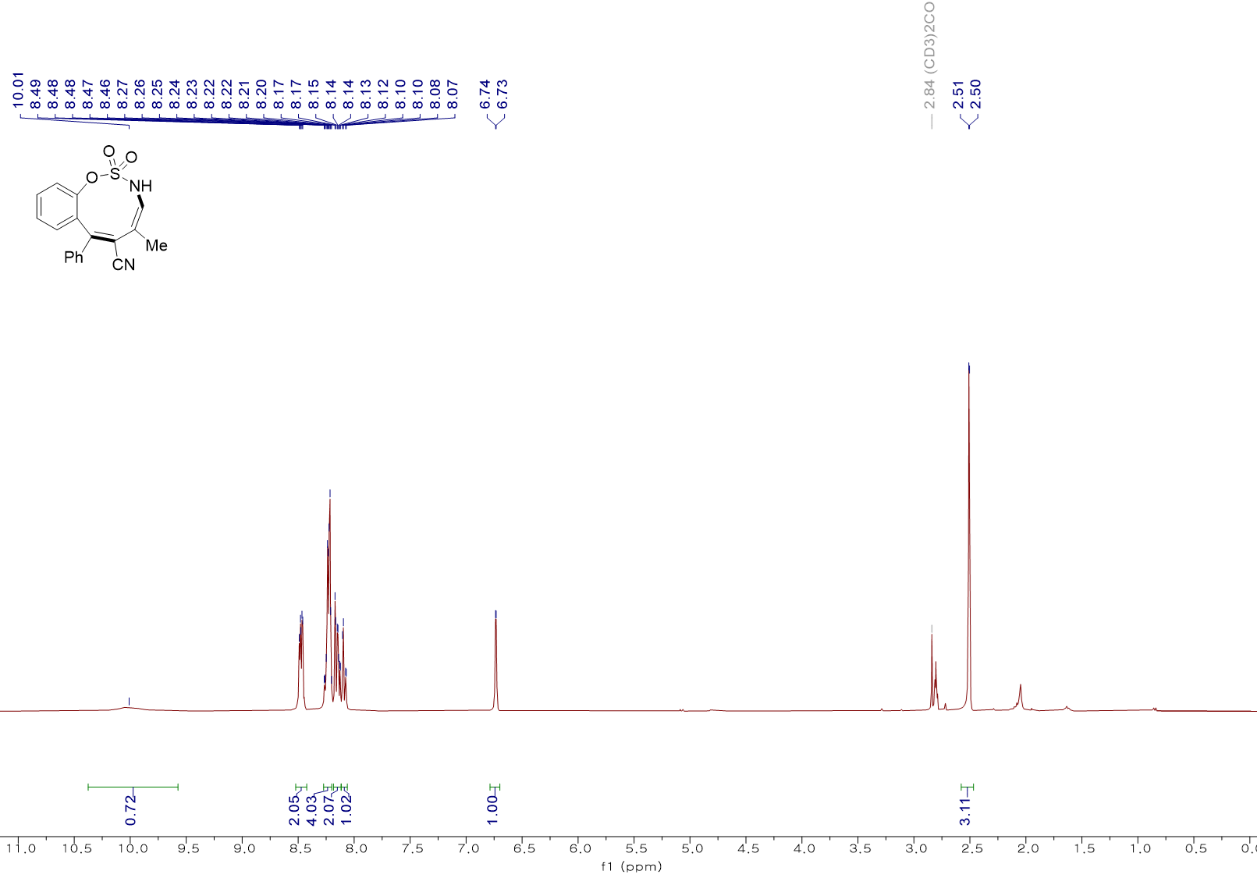


^1^H NMR spectrum of **5a**


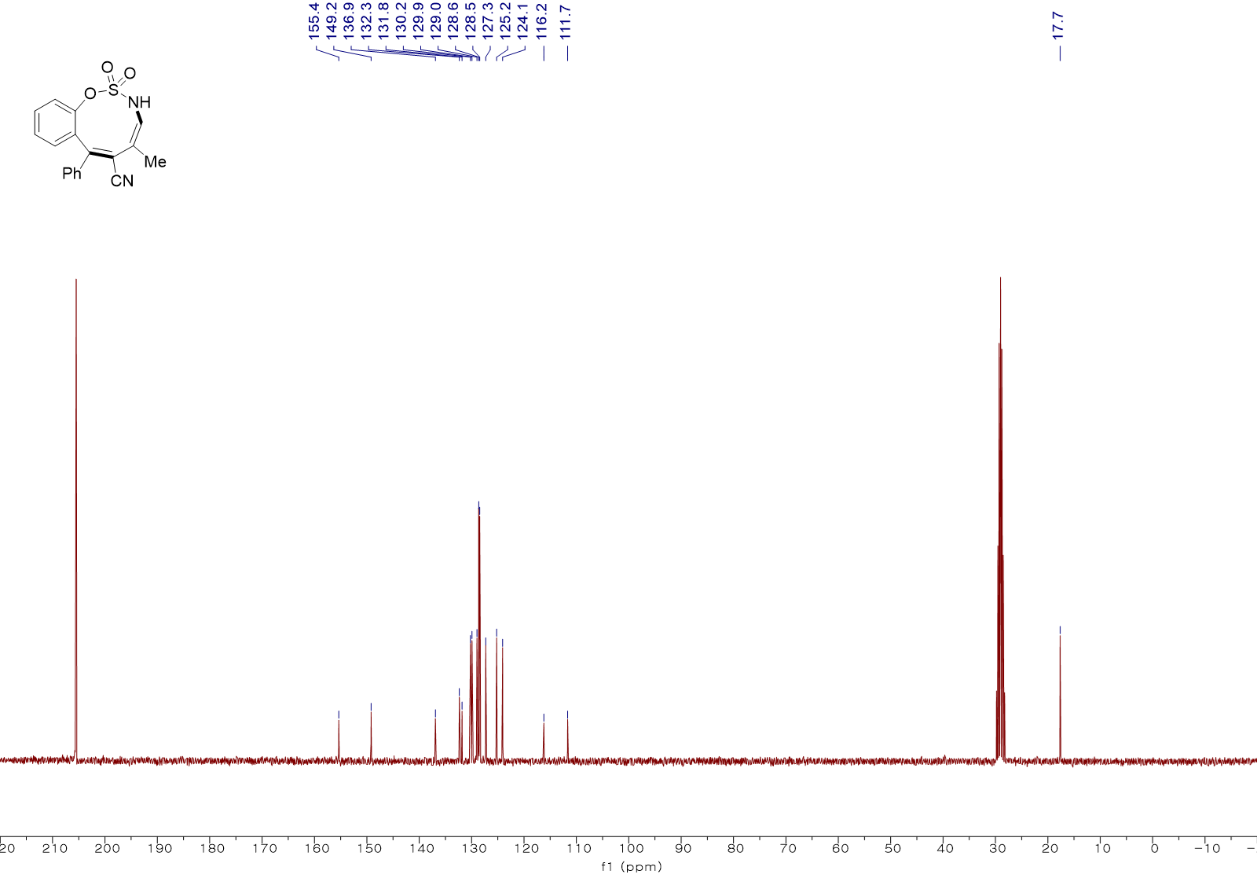


^13^C NMR spectrum of **5a**


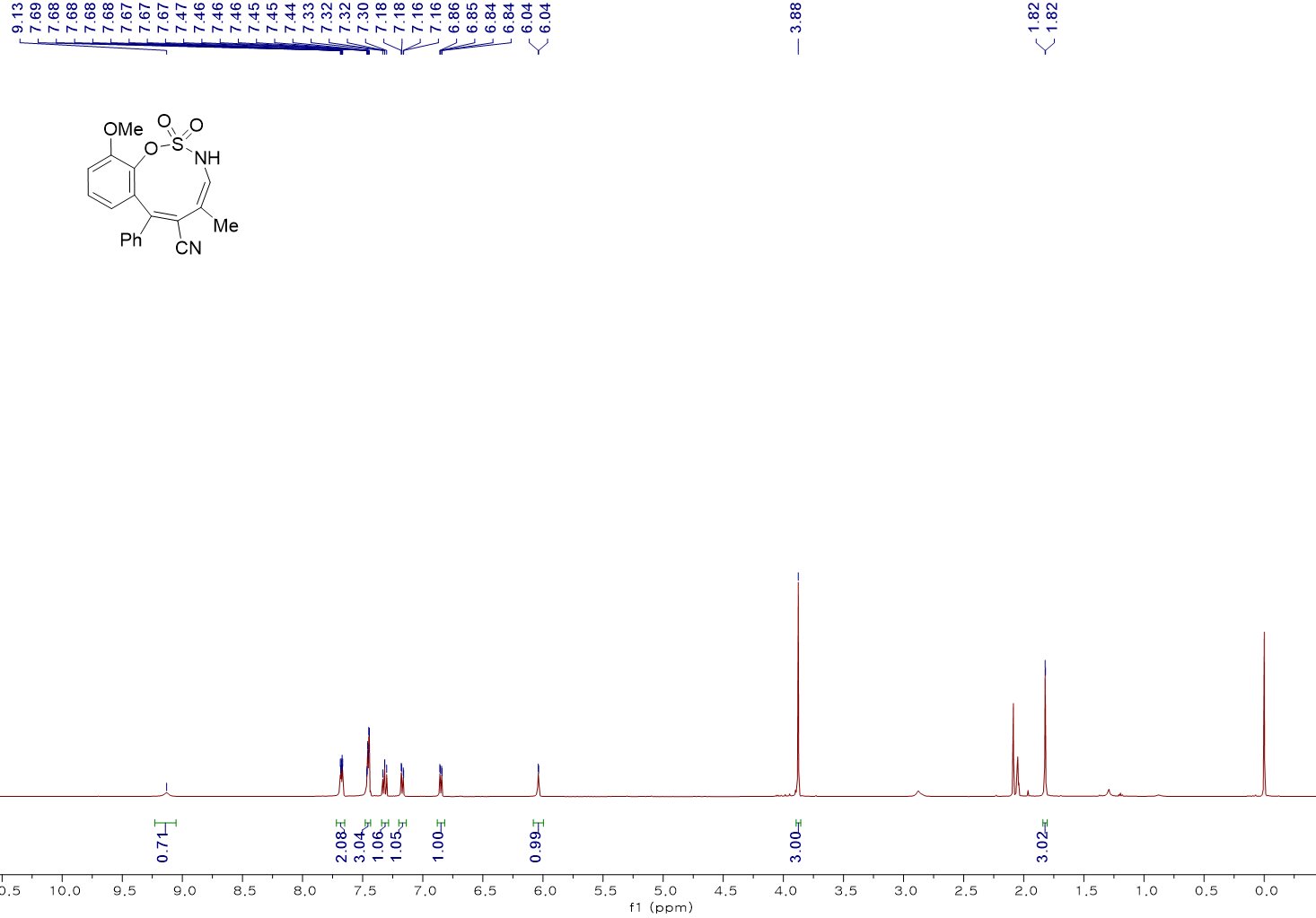


^1^H NMR spectrum of **5b**


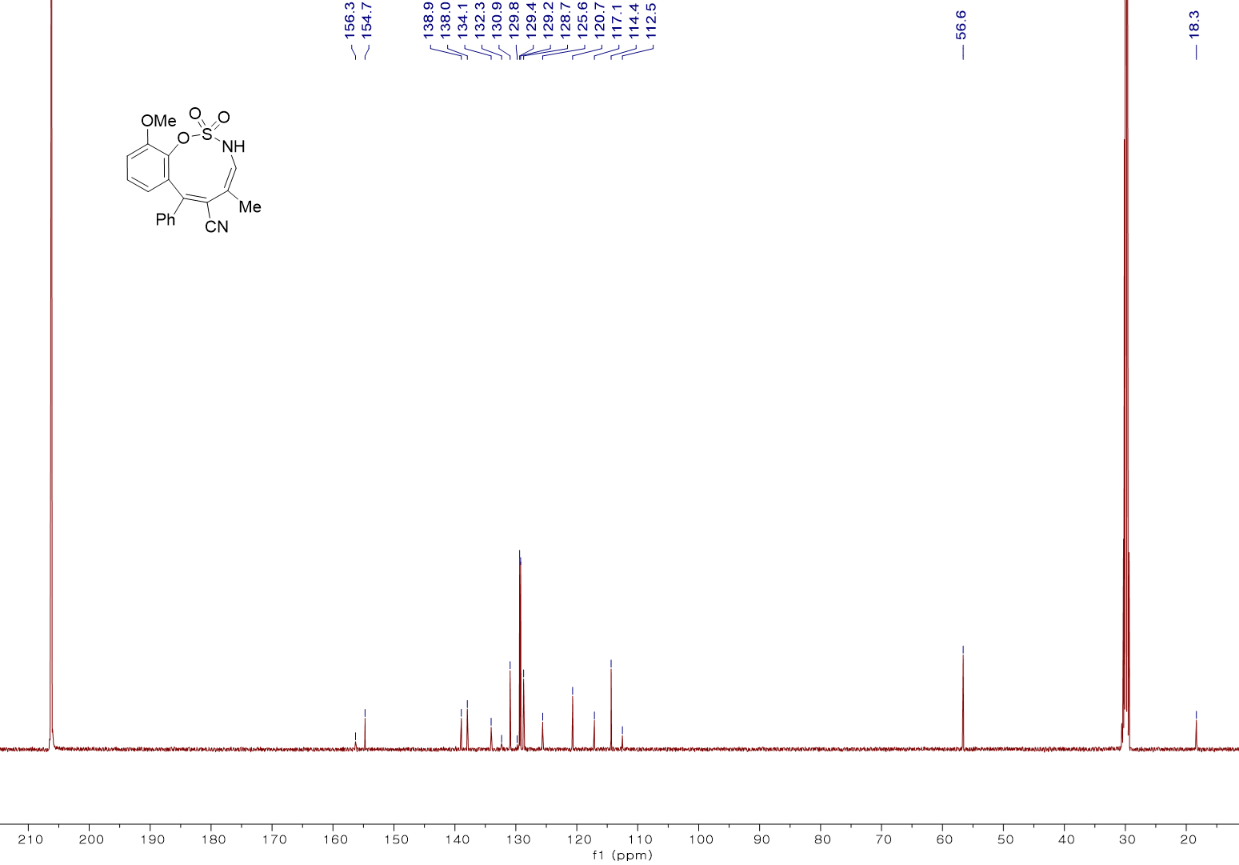


^13^C NMR spectrum of **5b**


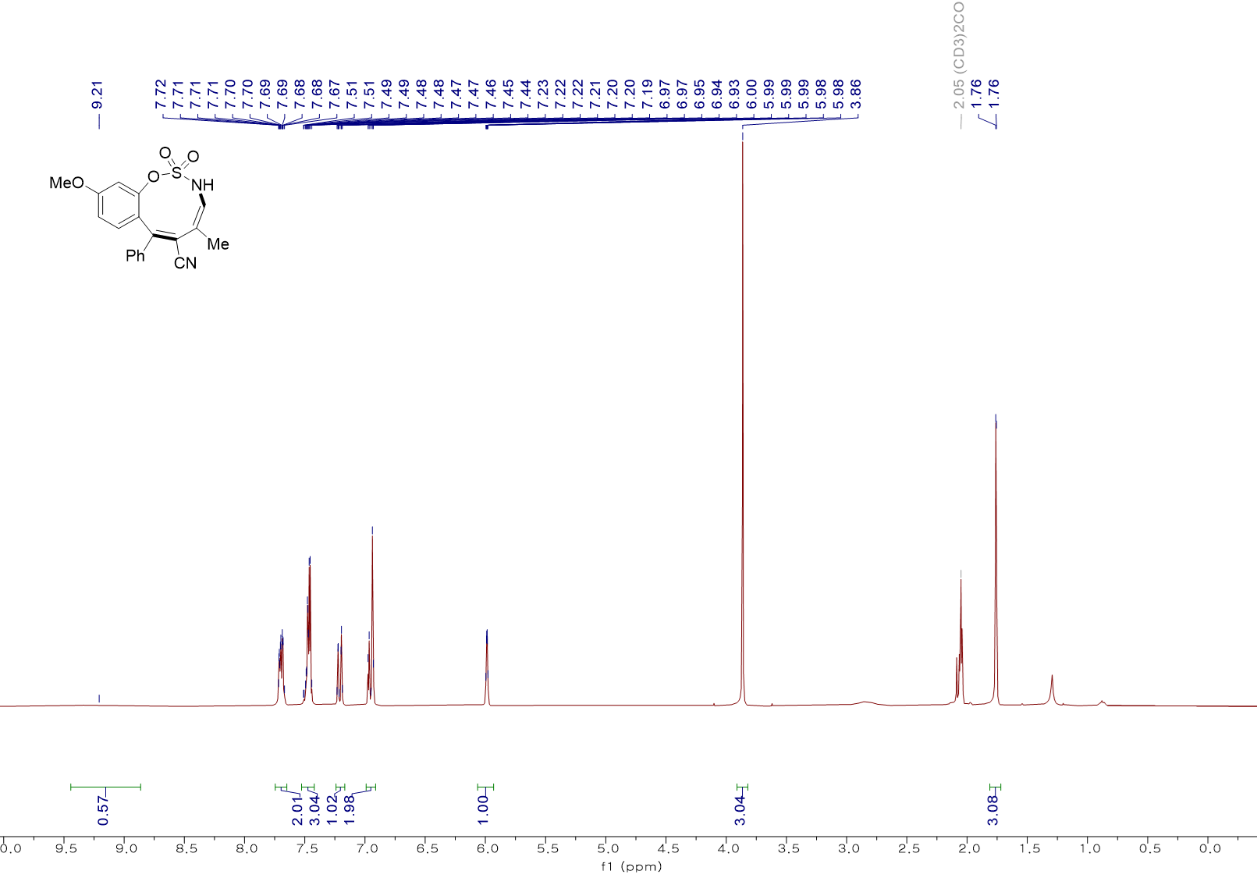


^1^H NMR spectrum of **5c**


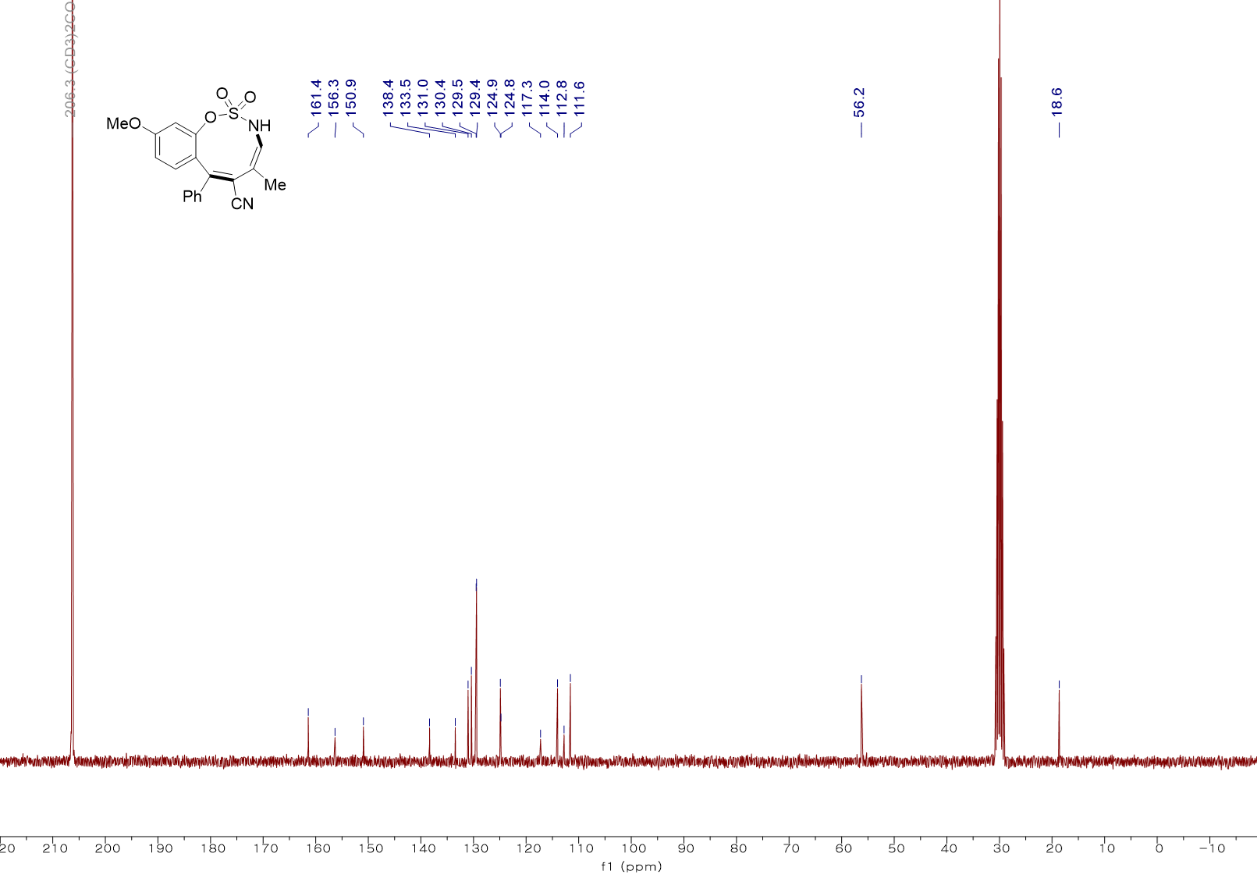


^13^C NMR spectrum of **5c**


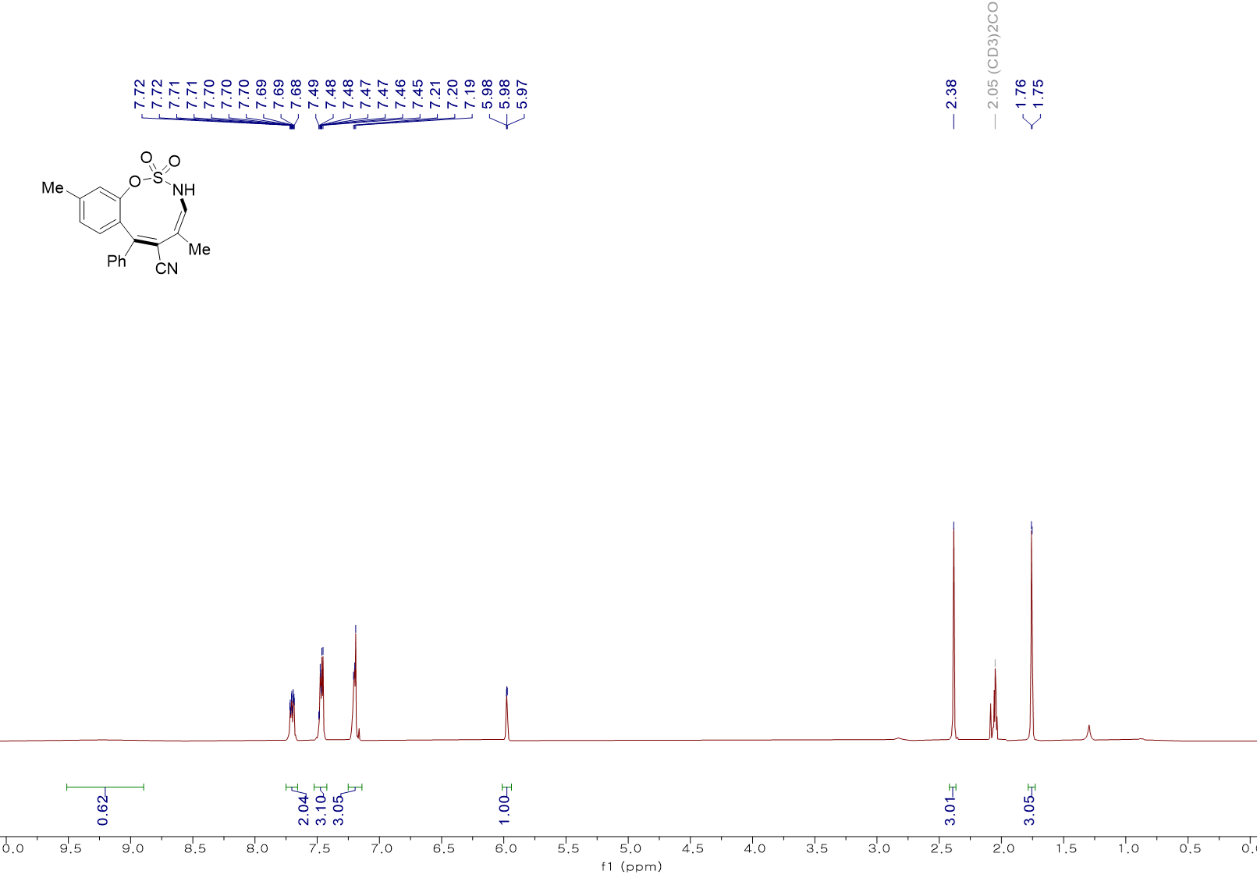


^1^H NMR spectrum of **5d**


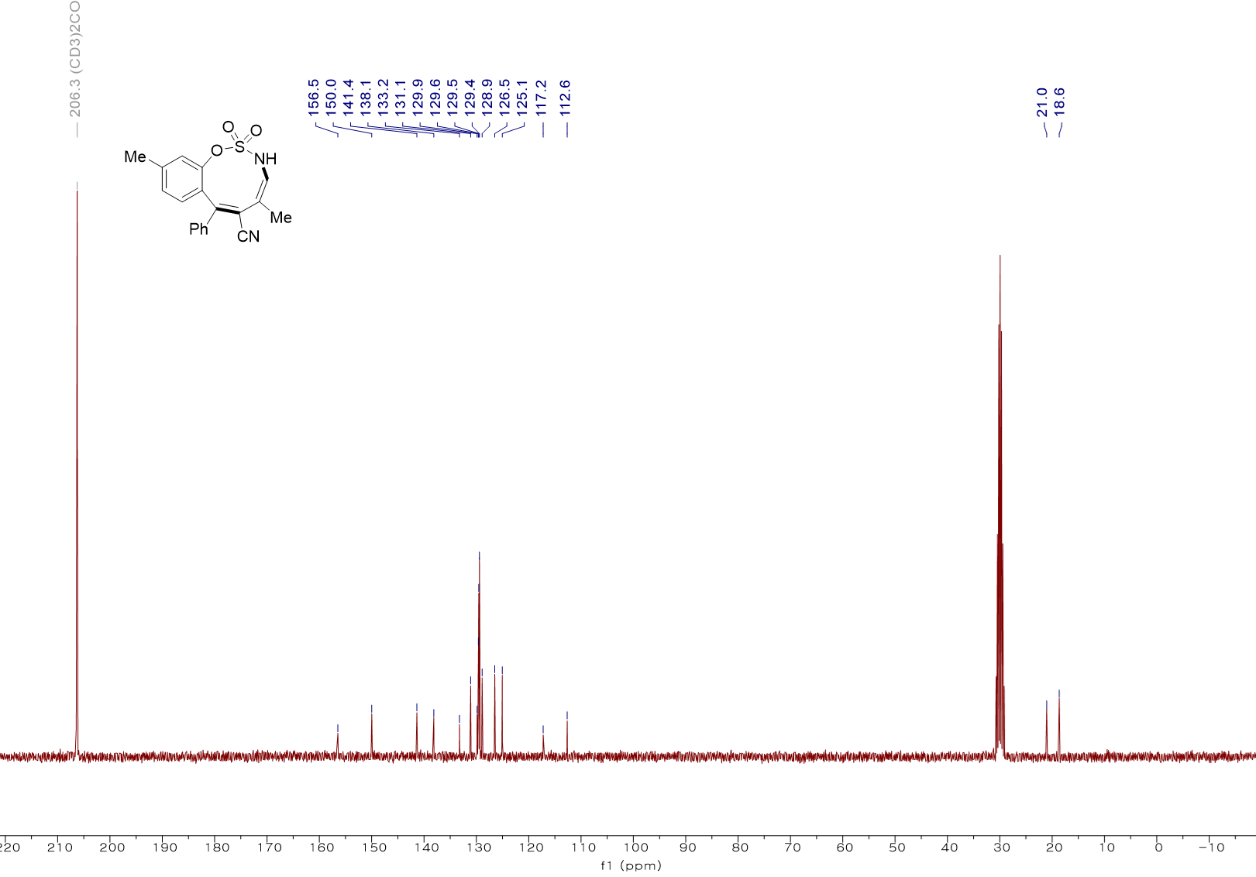


^13^C NMR spectrum of **5d**


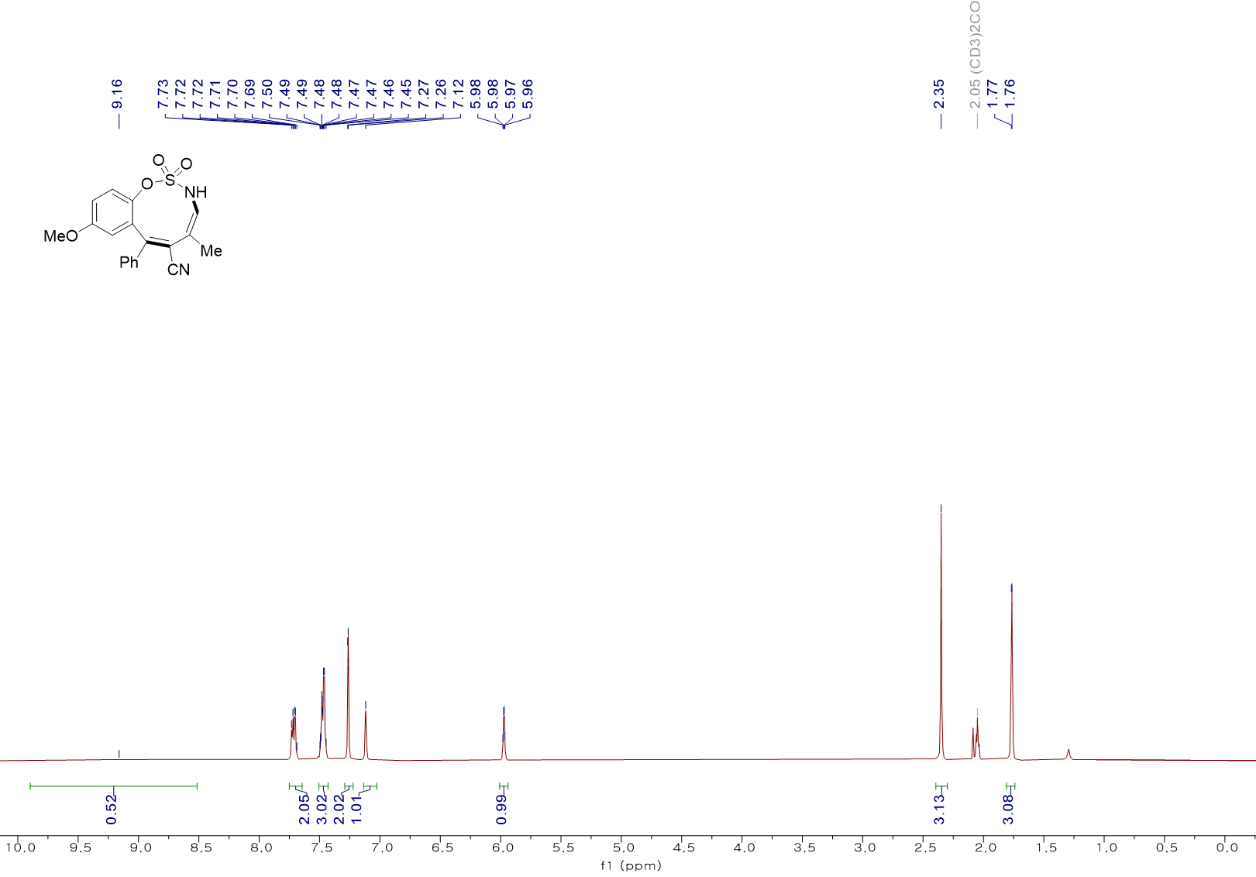


^1^H NMR spectrum of **5e**


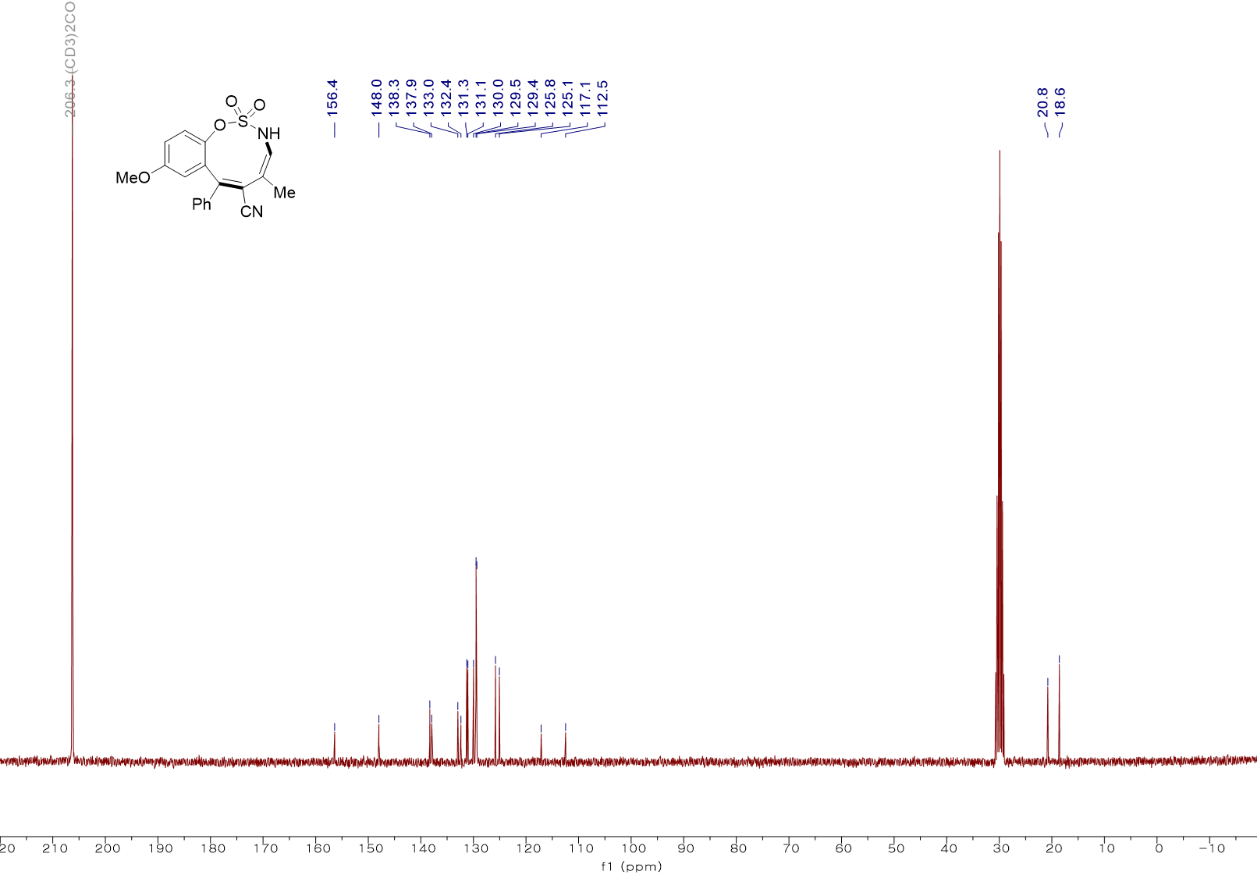


^13^C NMR spectrum of **5e**


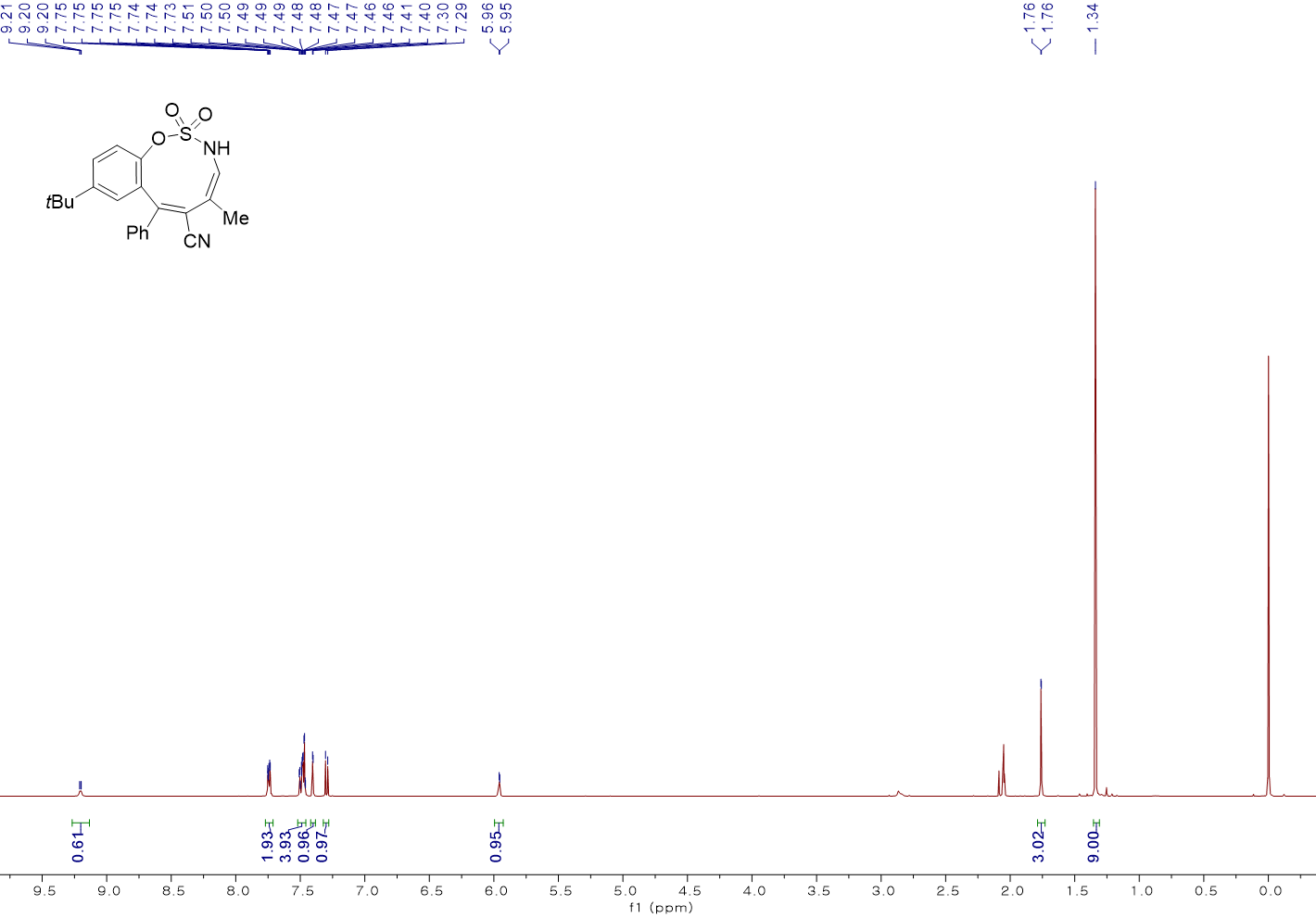


^1^H NMR spectrum of **5f**


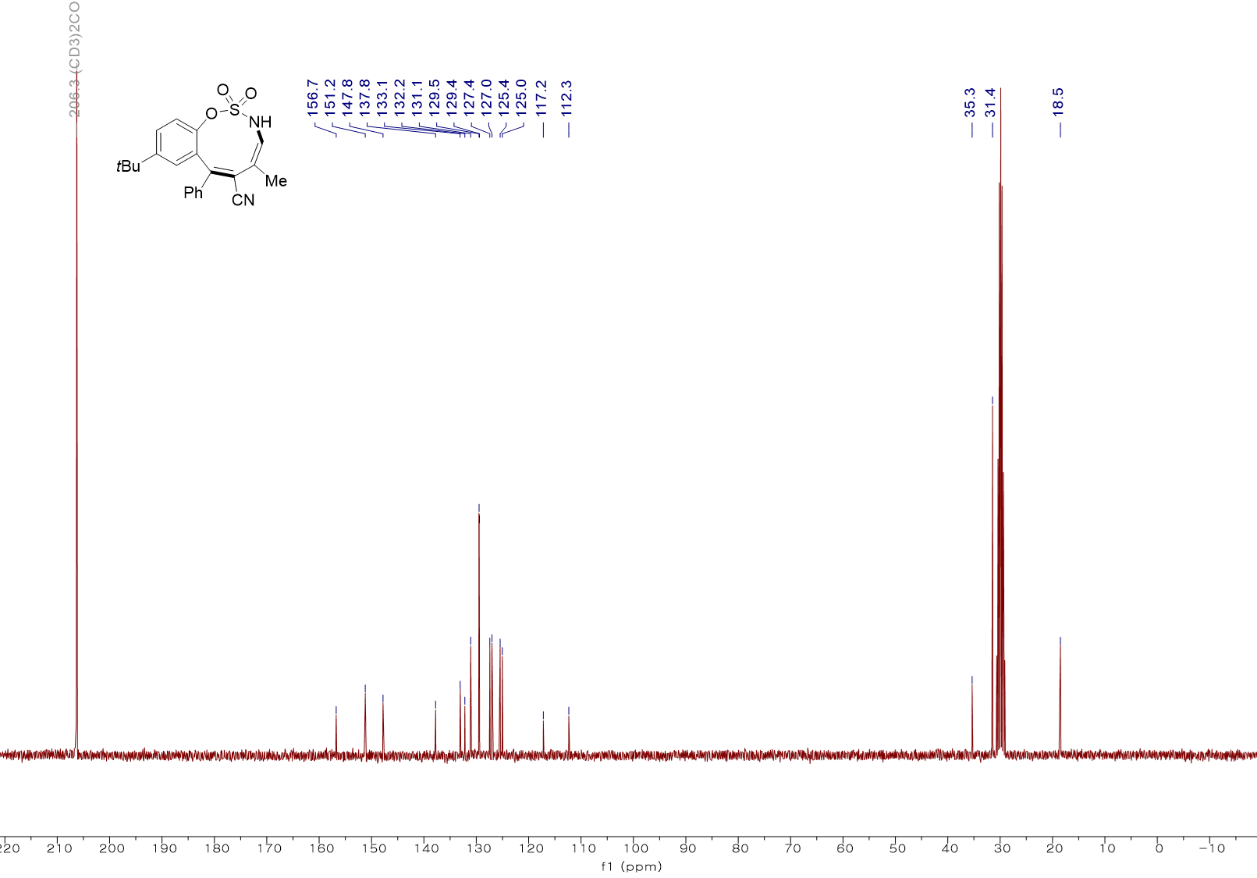


^13^C NMR spectrum of **5f**


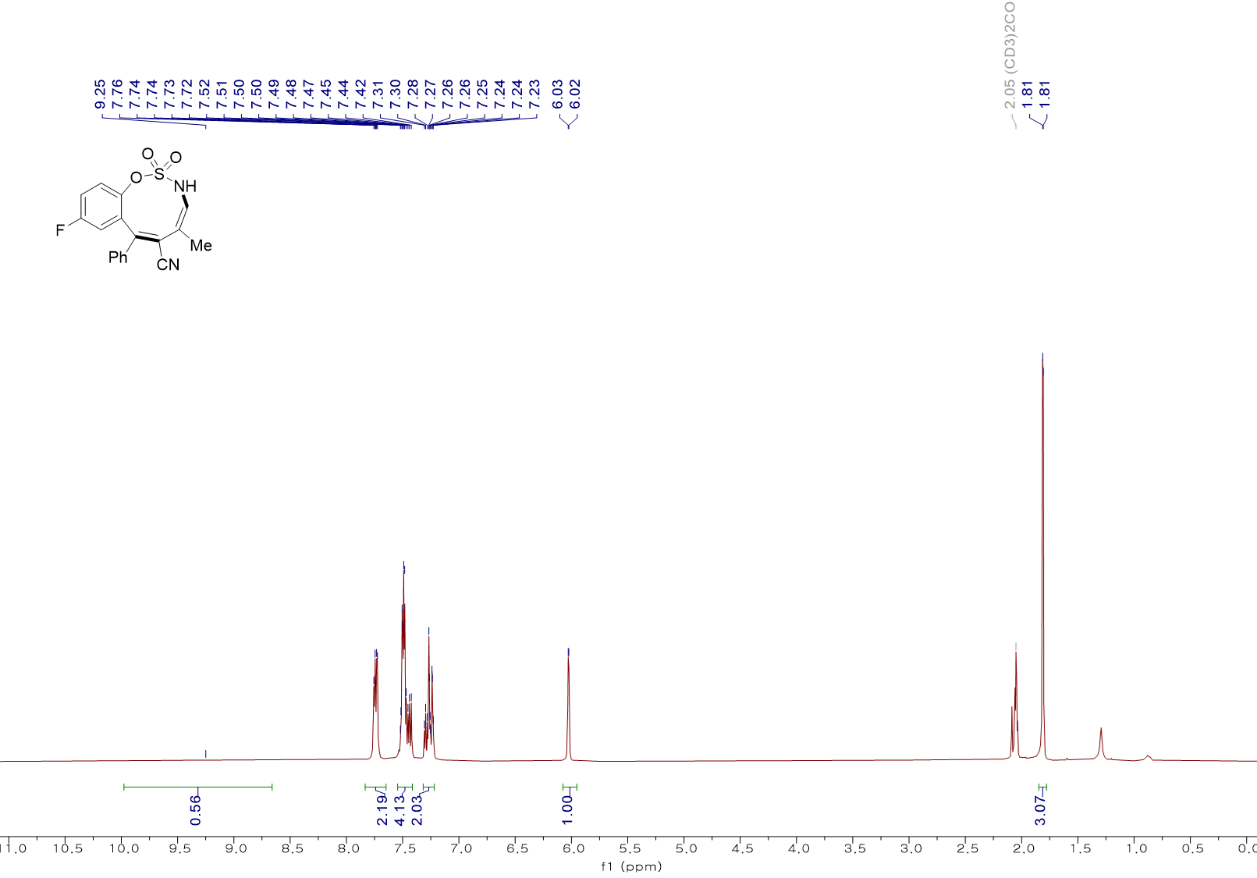


^1^H NMR spectrum of **5g**


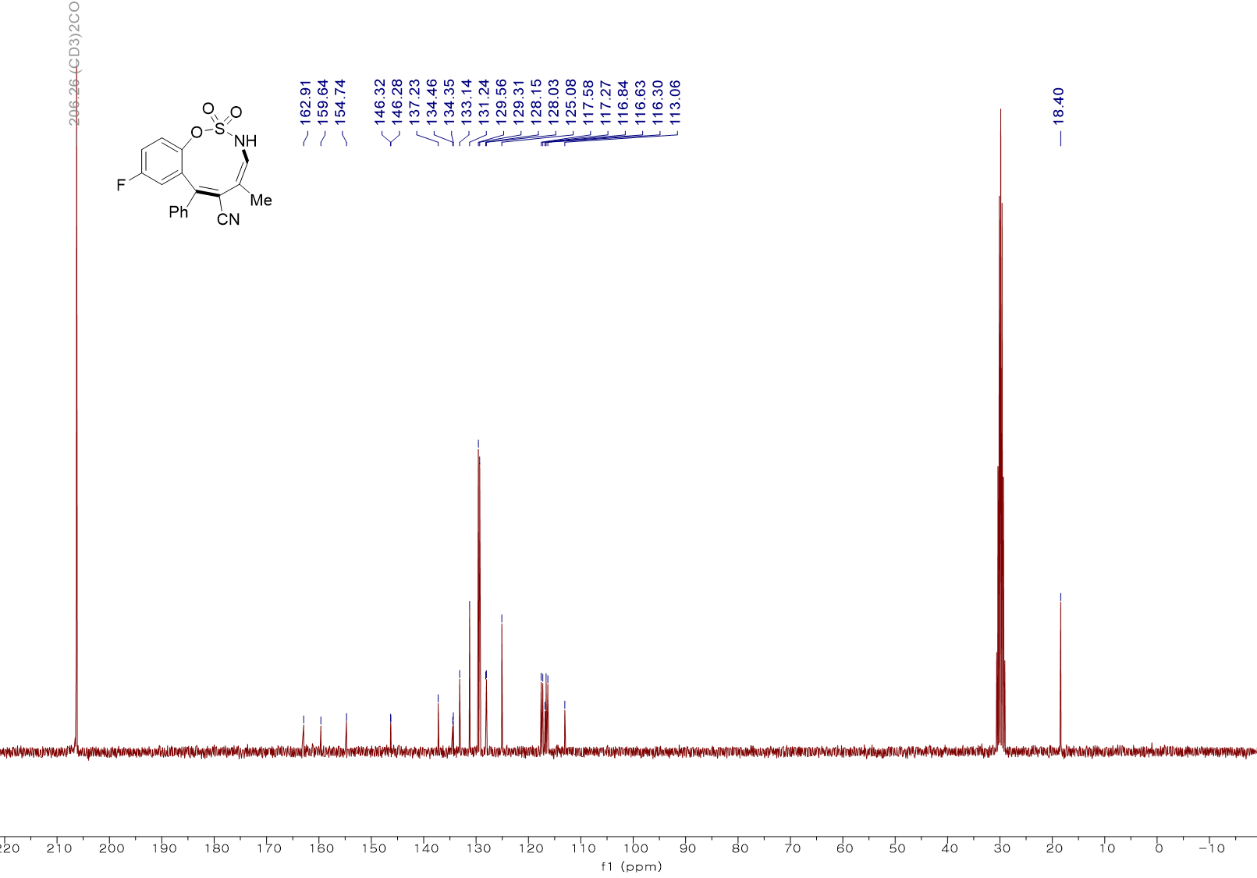


^13^C NMR spectrum of **5g**


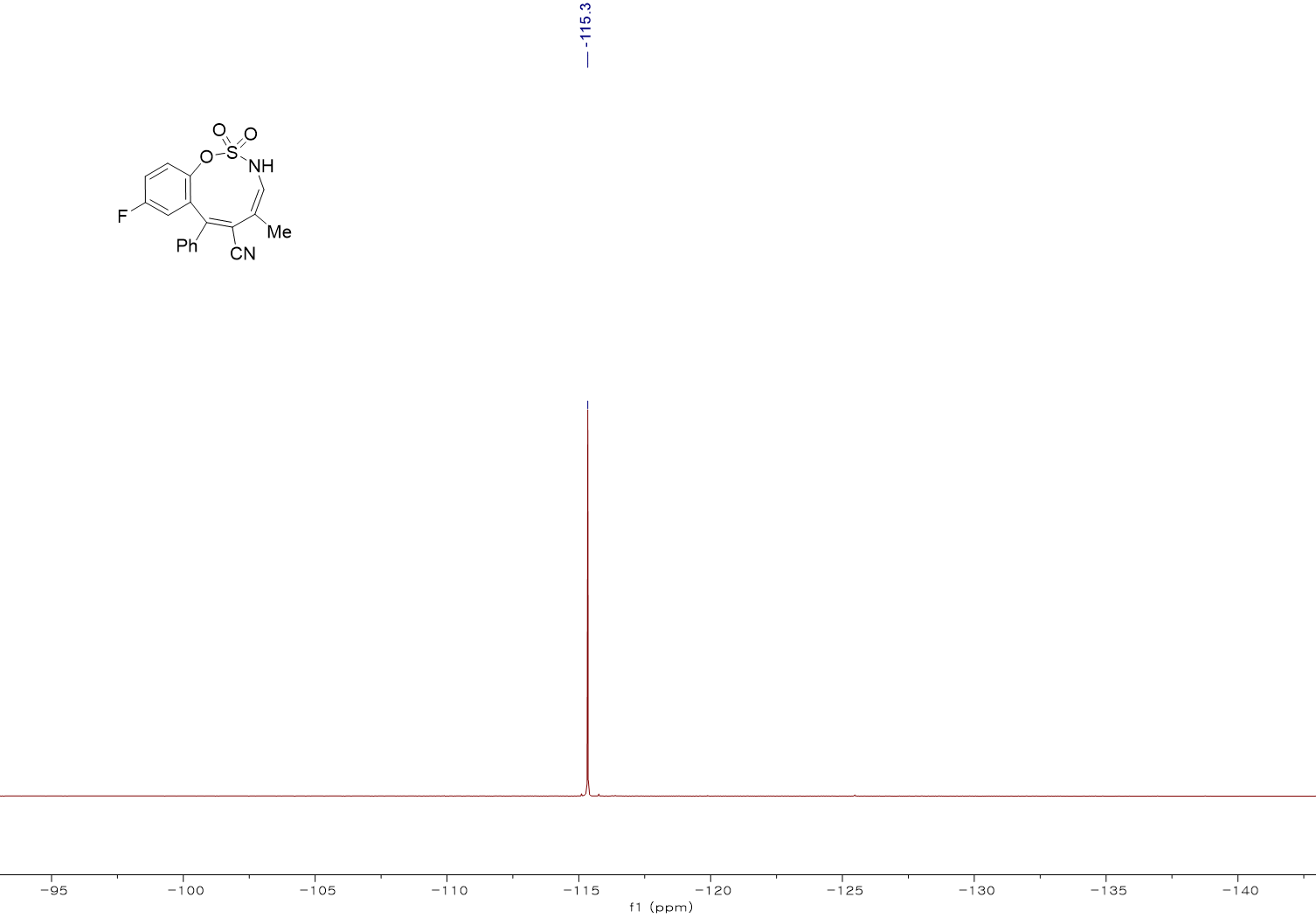


^19^F NMR spectrum of **5g**


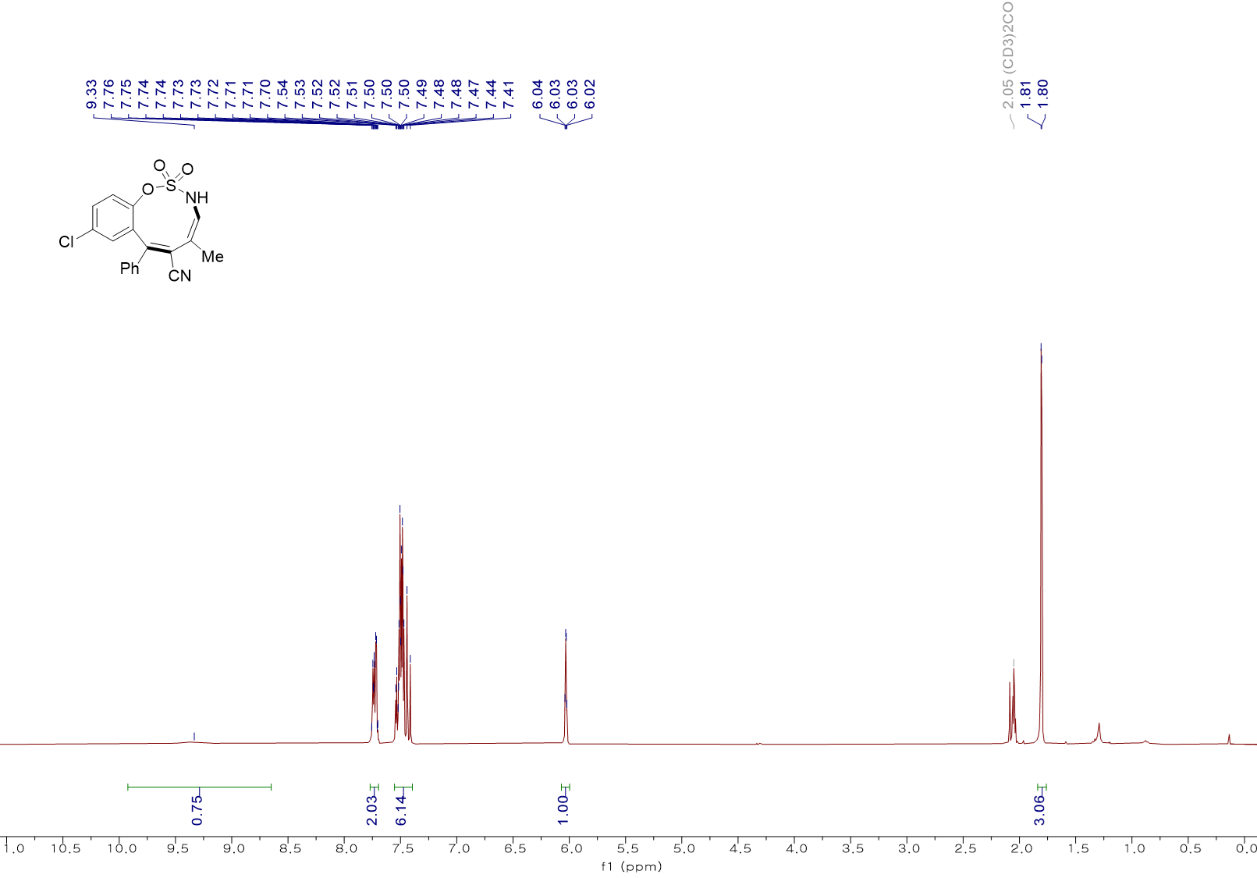


^1^H NMR spectrum of **5h**


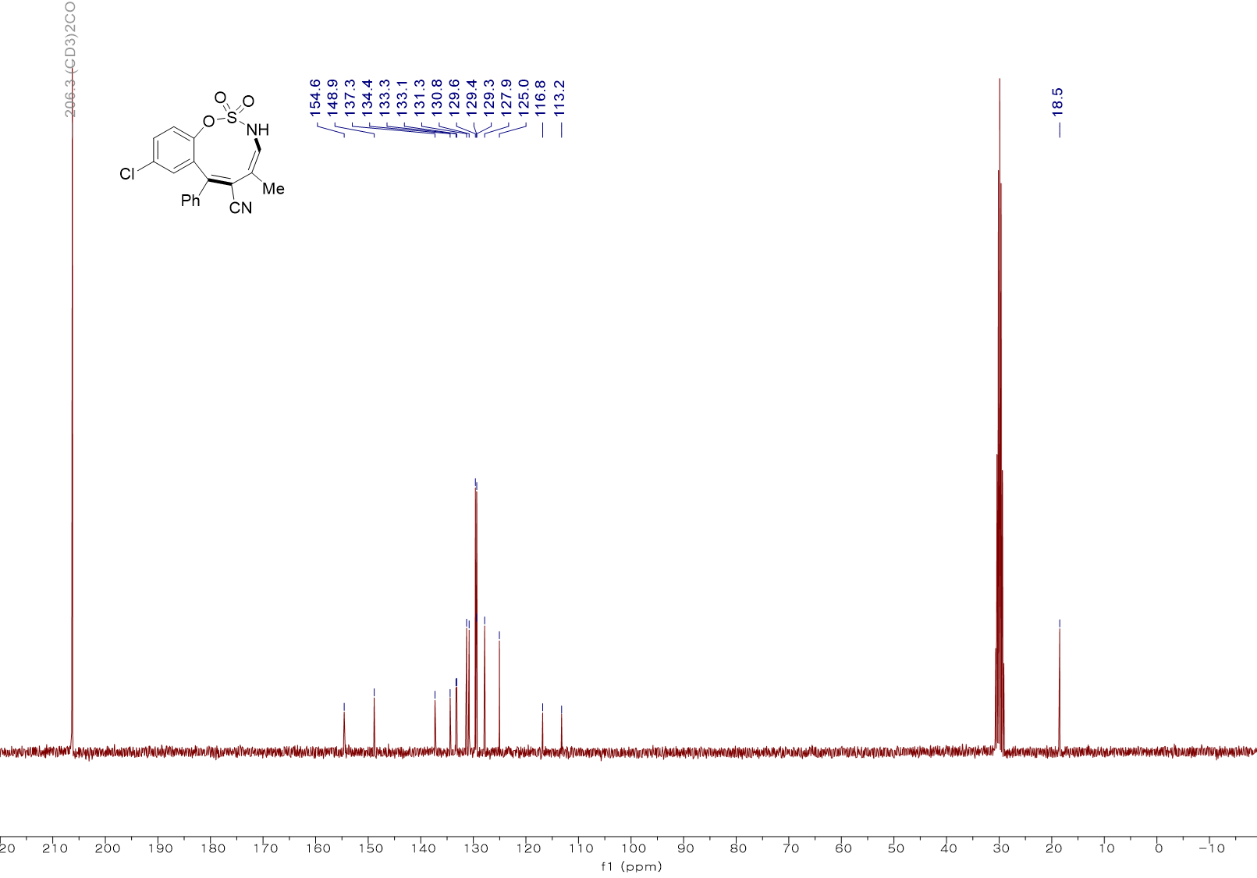


^13^C NMR spectrum of **5h**


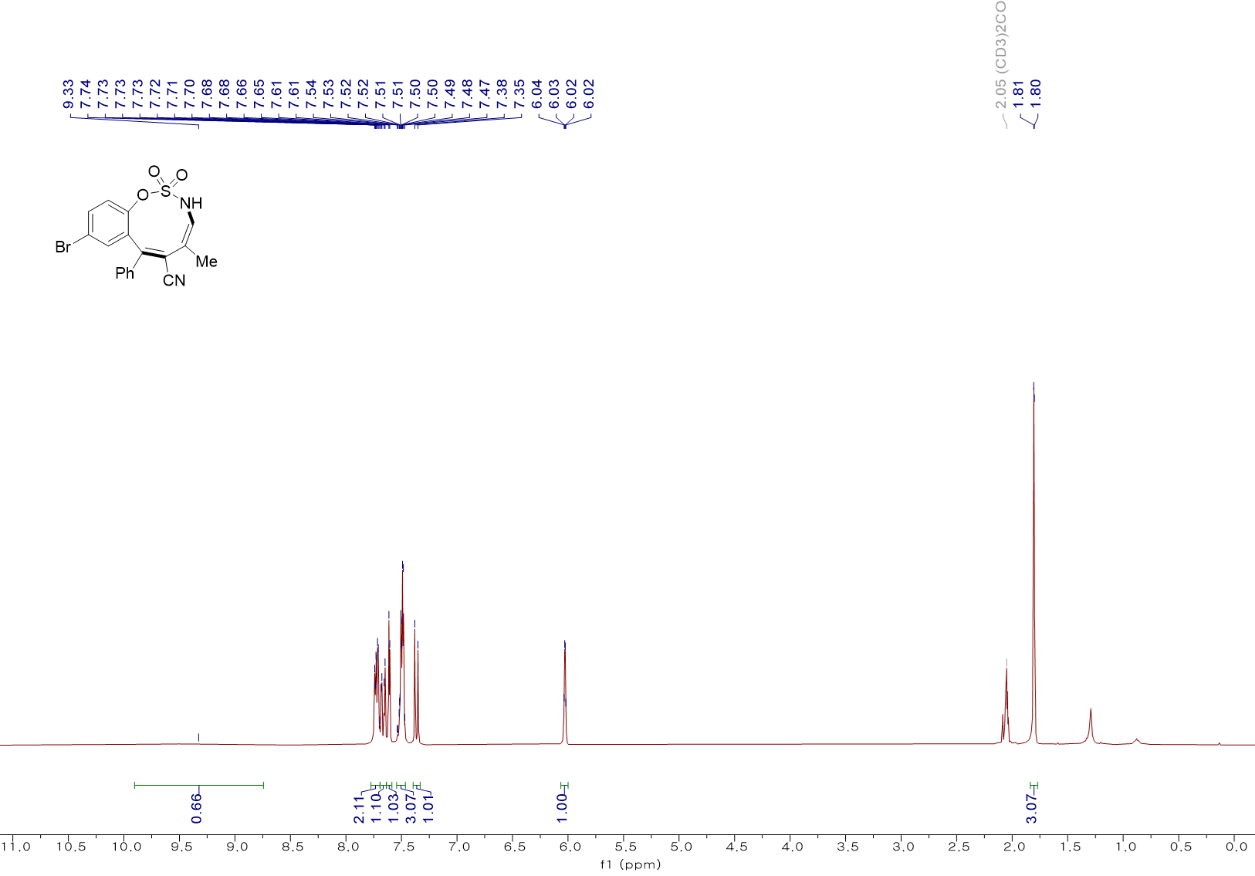


^1^H NMR spectrum of **5i**


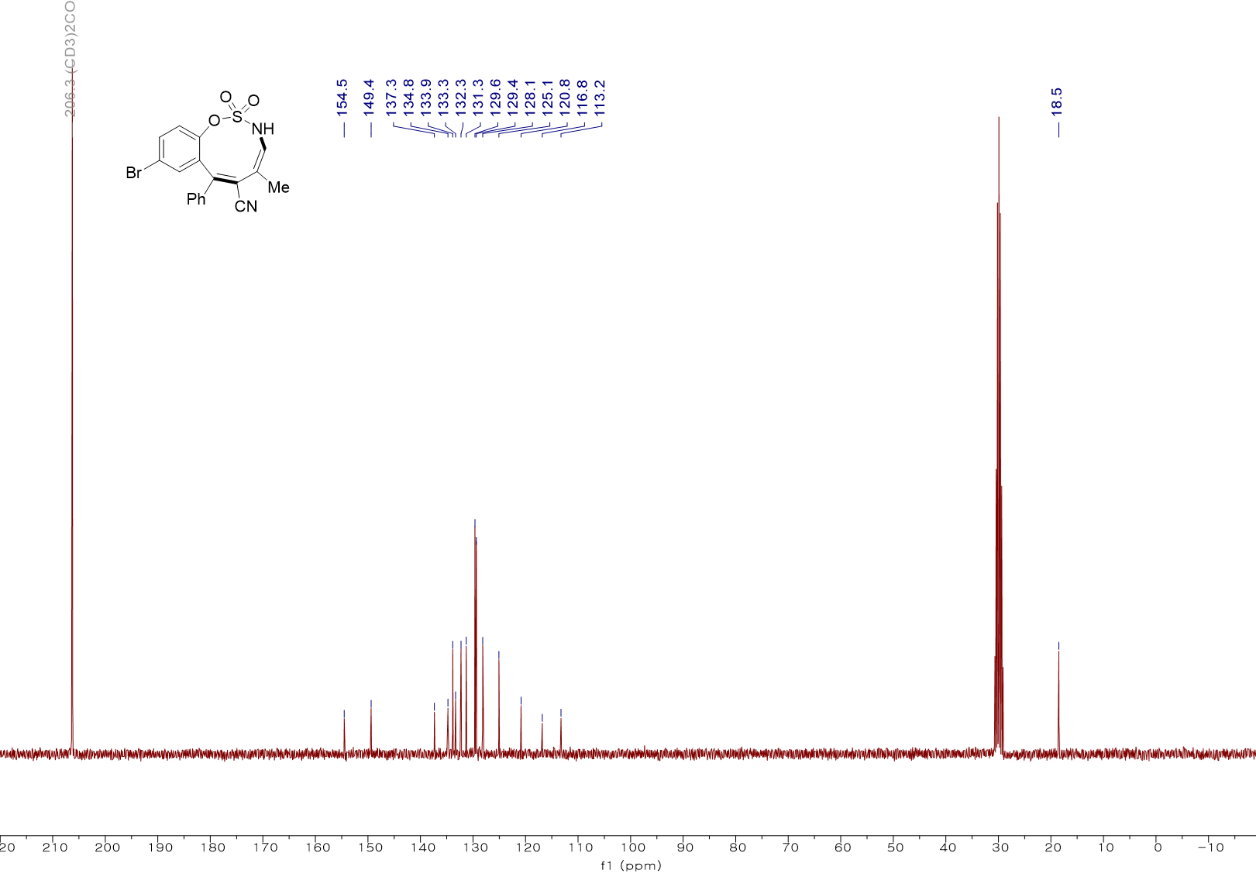


^13^C NMR spectrum of **5i**


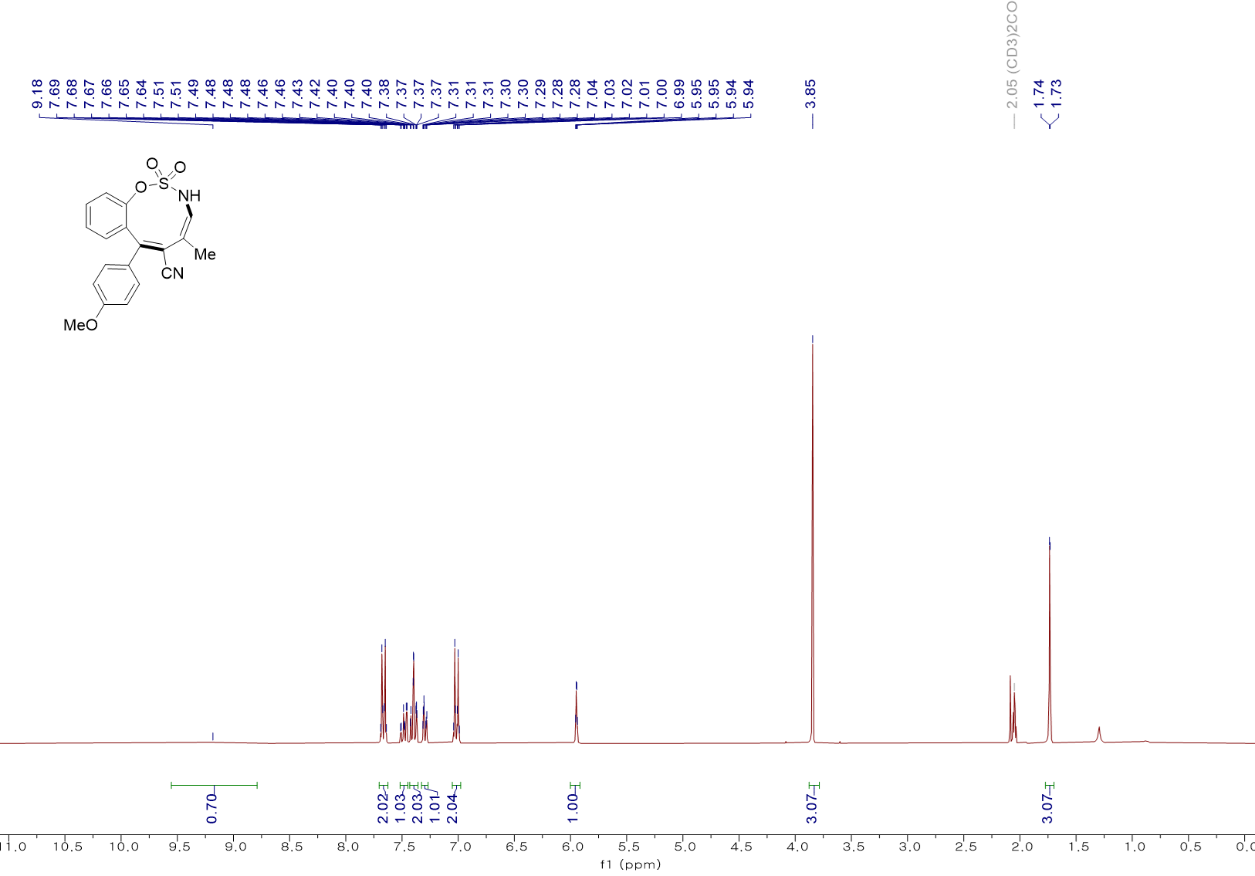


^1^H NMR spectrum of **5j**


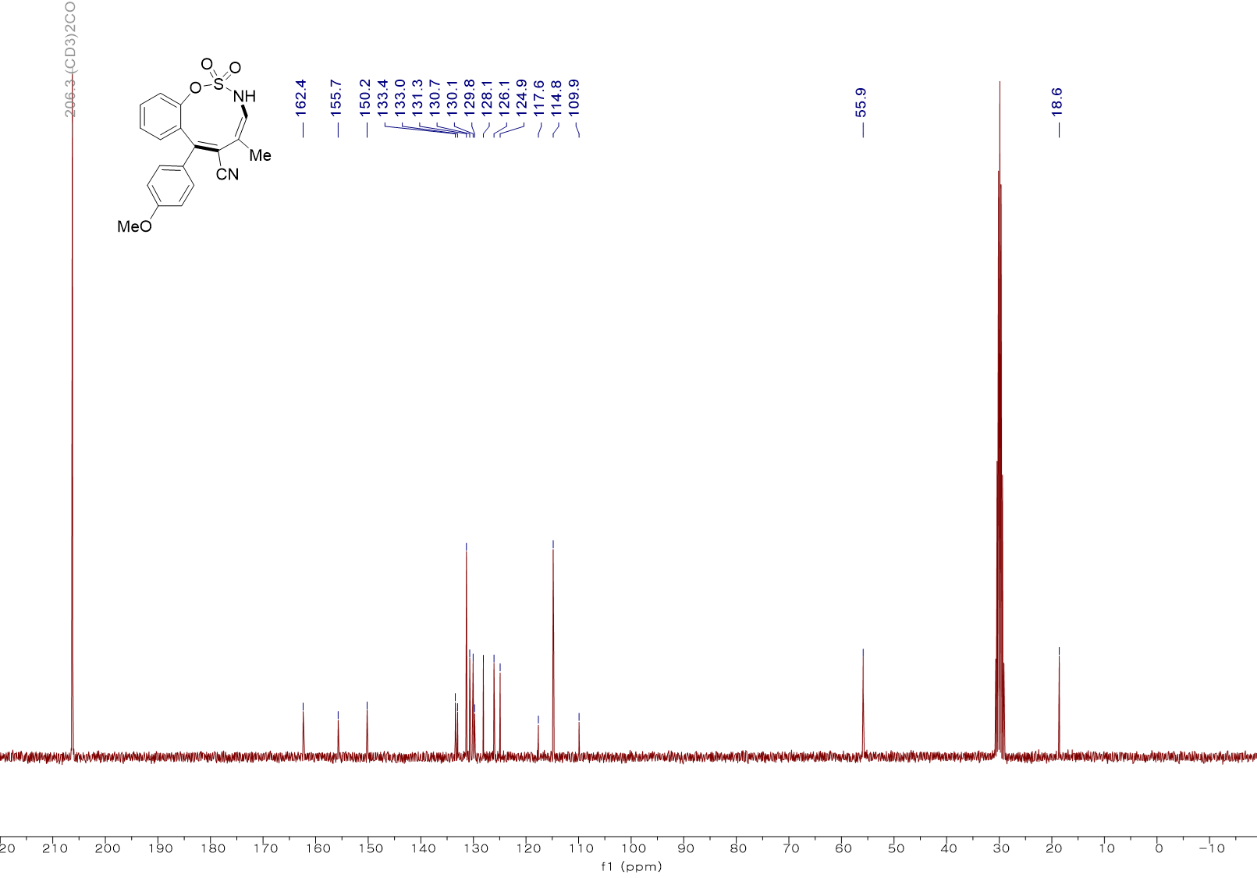


^13^C NMR spectrum of **5j**


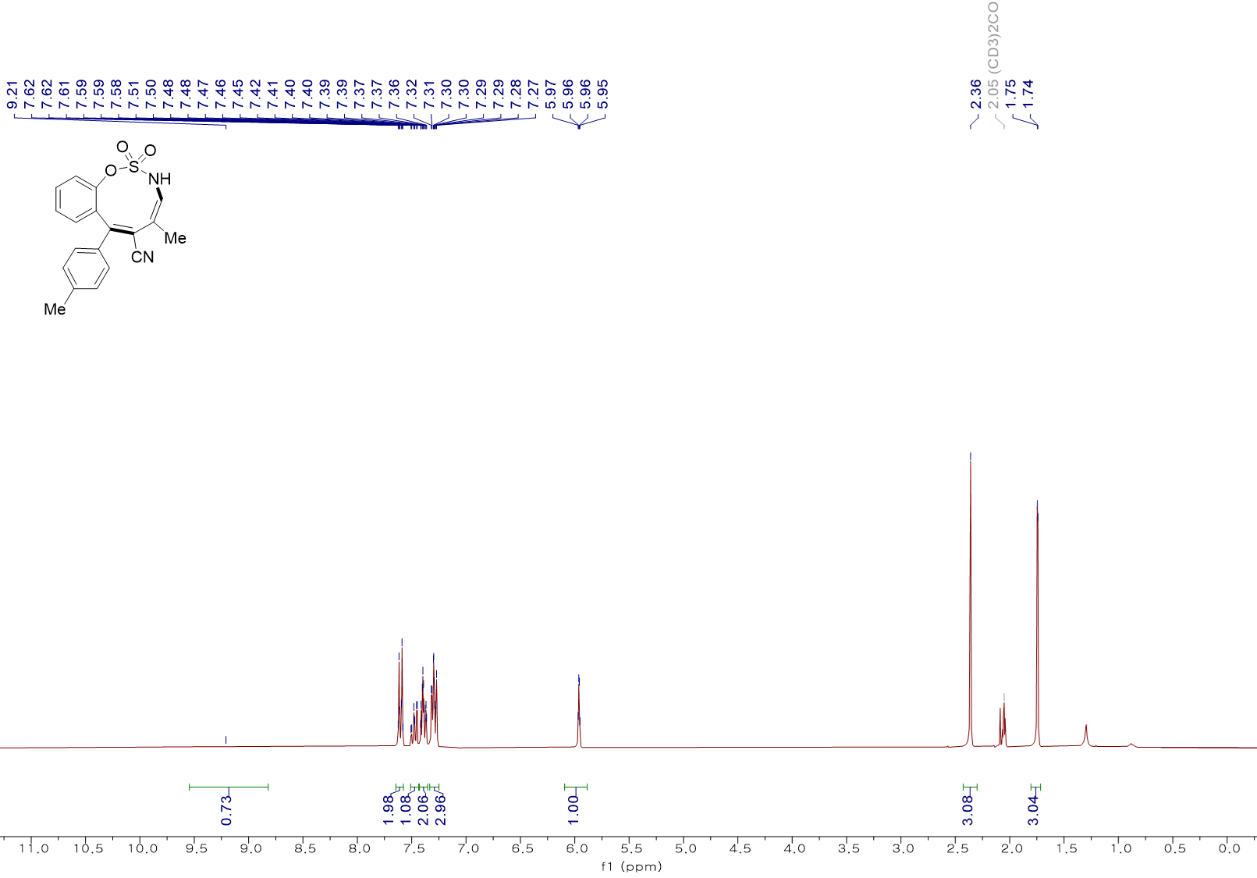


^1^H NMR spectrum of **5k**


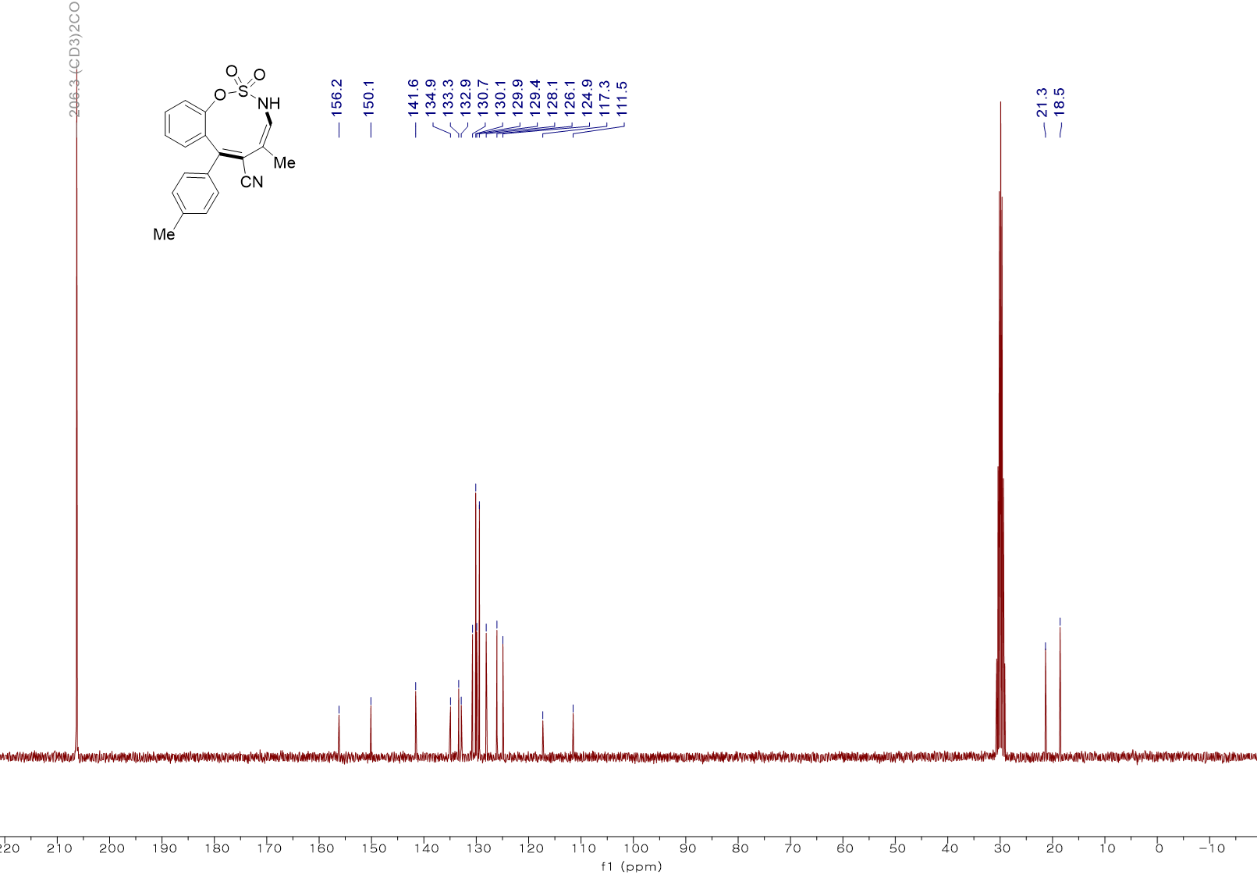


^13^C NMR spectrum of **5k**


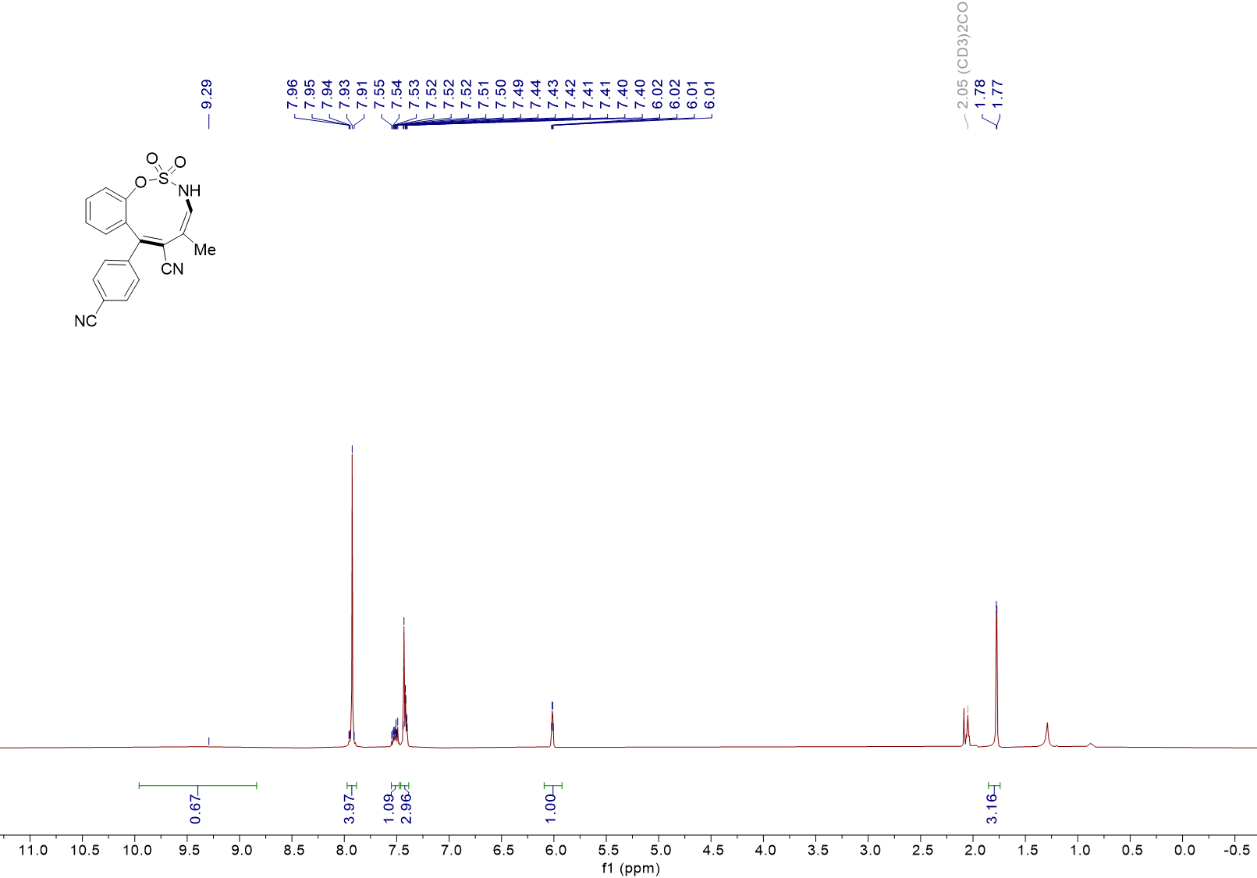


^1^H NMR spectrum of **5l**


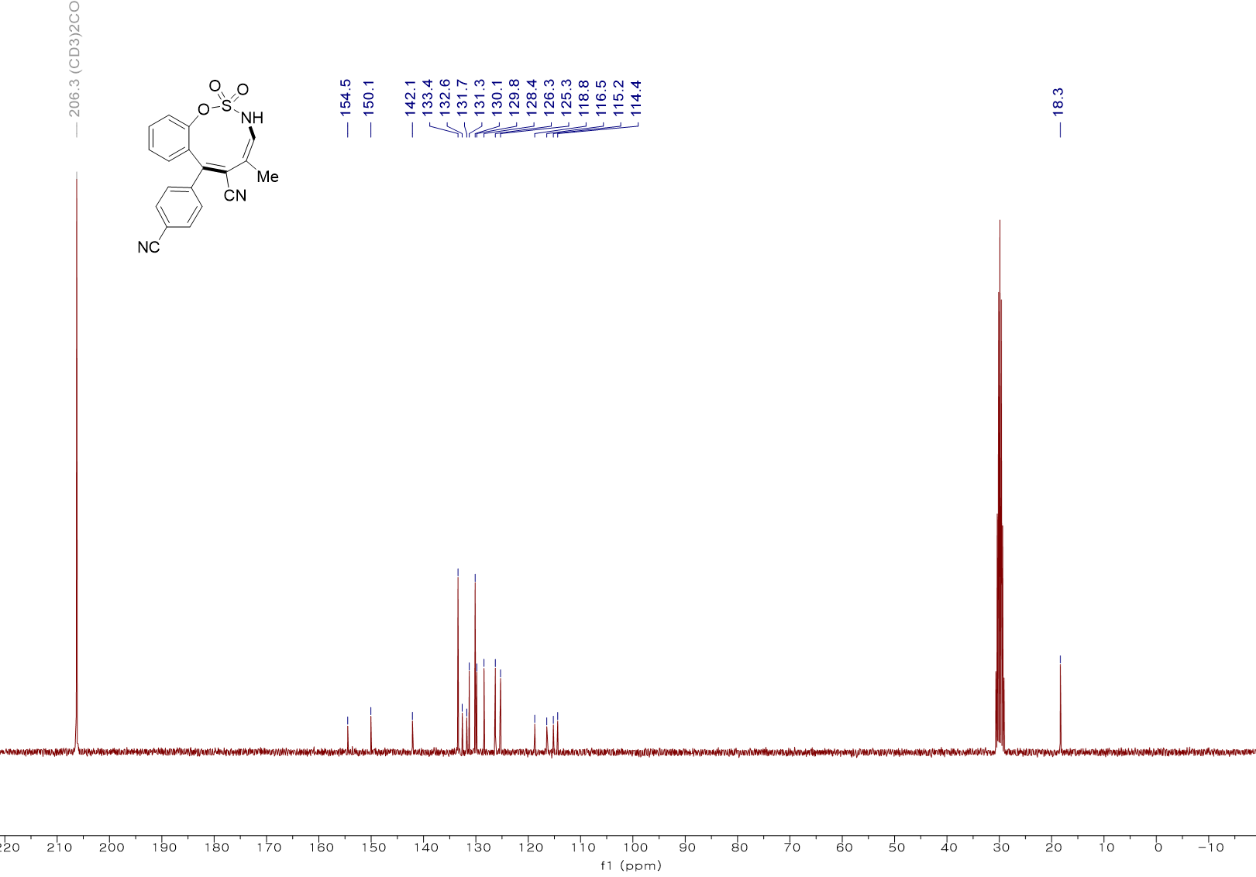


^13^C NMR spectrum of **5l**


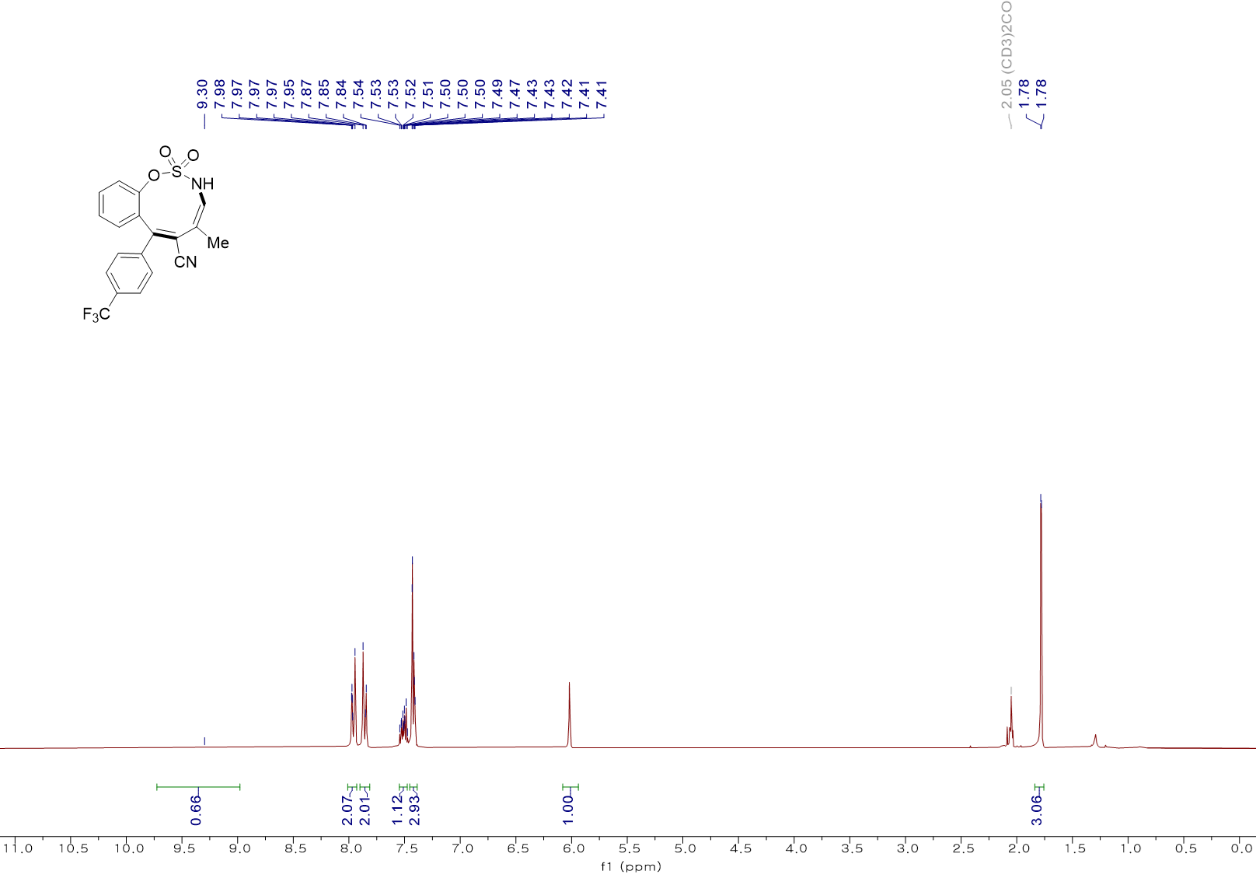


^1^H NMR spectrum of **5m**


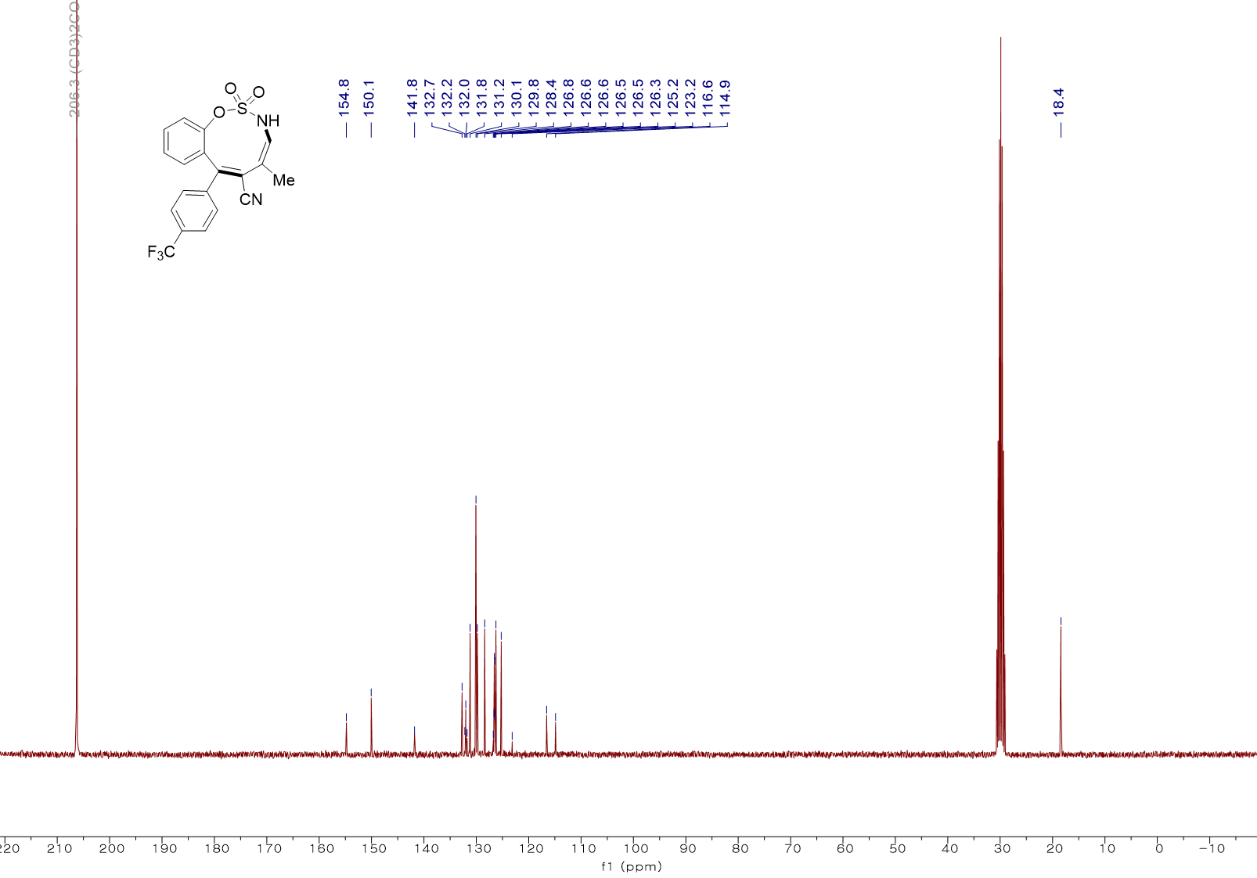


^13^C NMR spectrum of **5m**


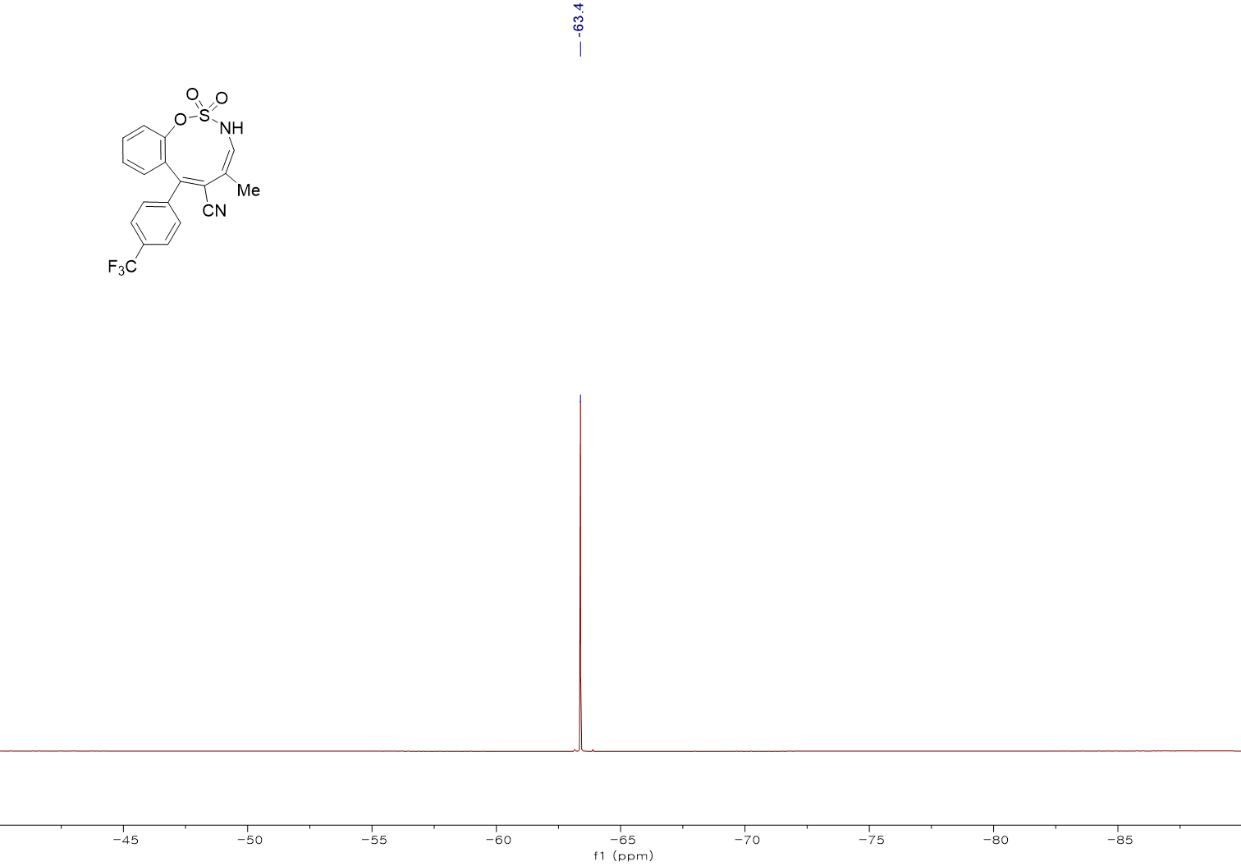


^19^F NMR spectrum of **5m**


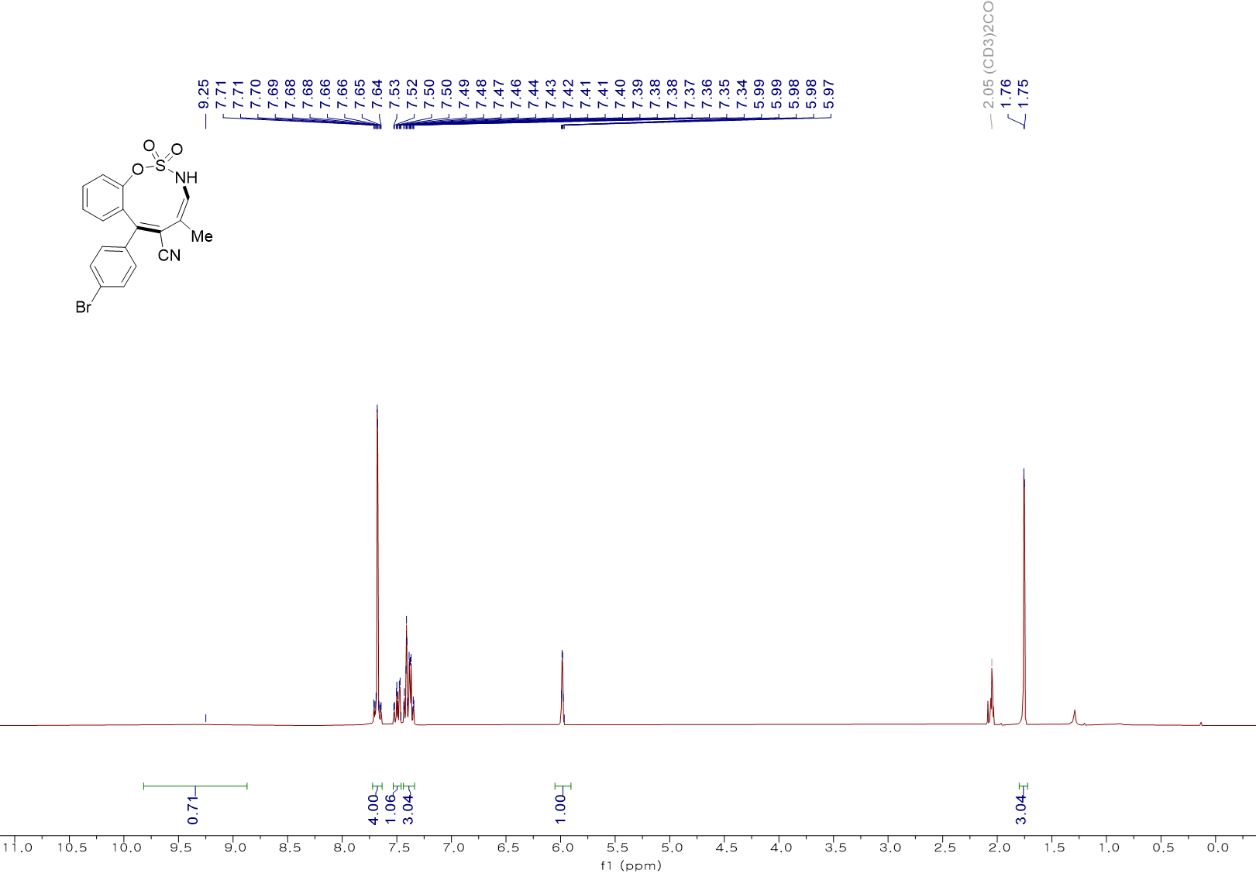


^1^H NMR spectrum of **5n**


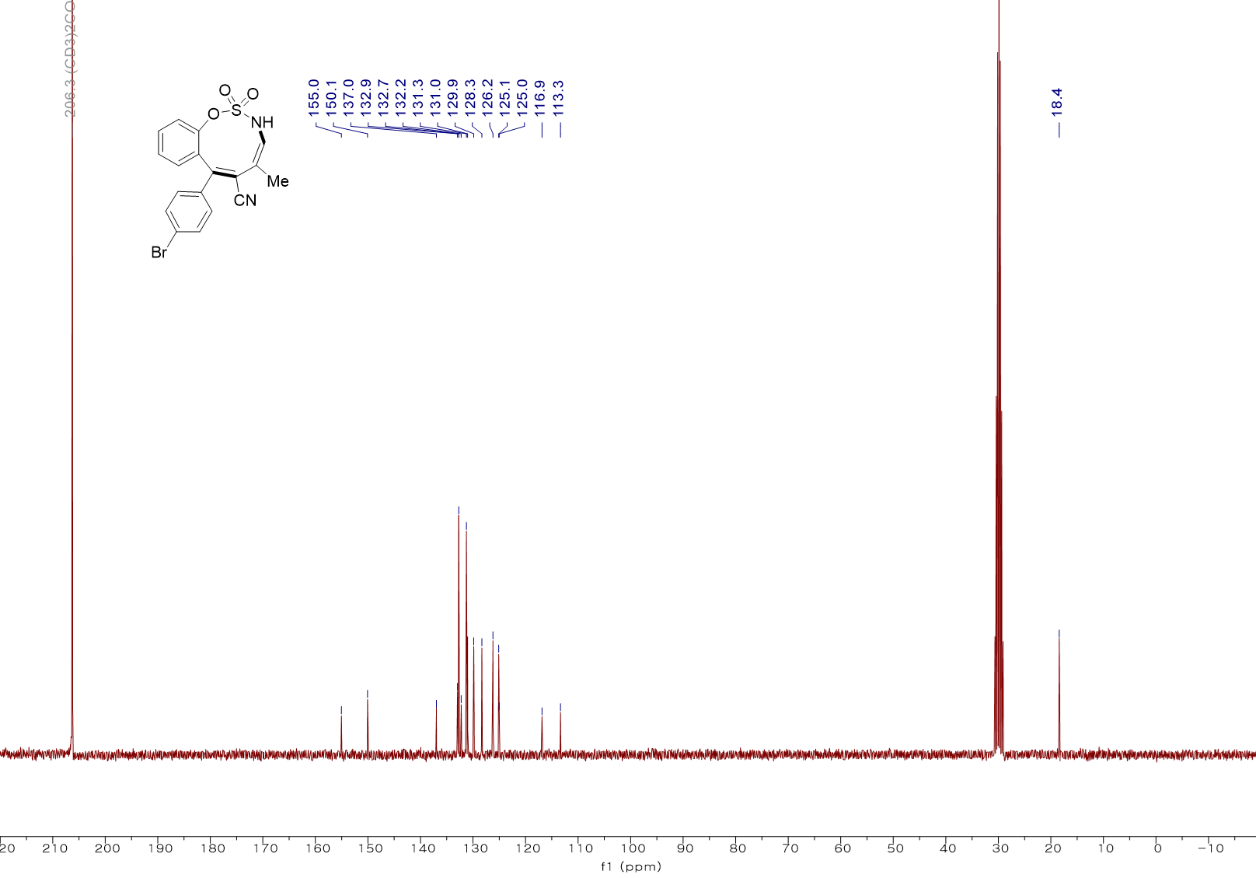


^13^C NMR spectrum of **5n**


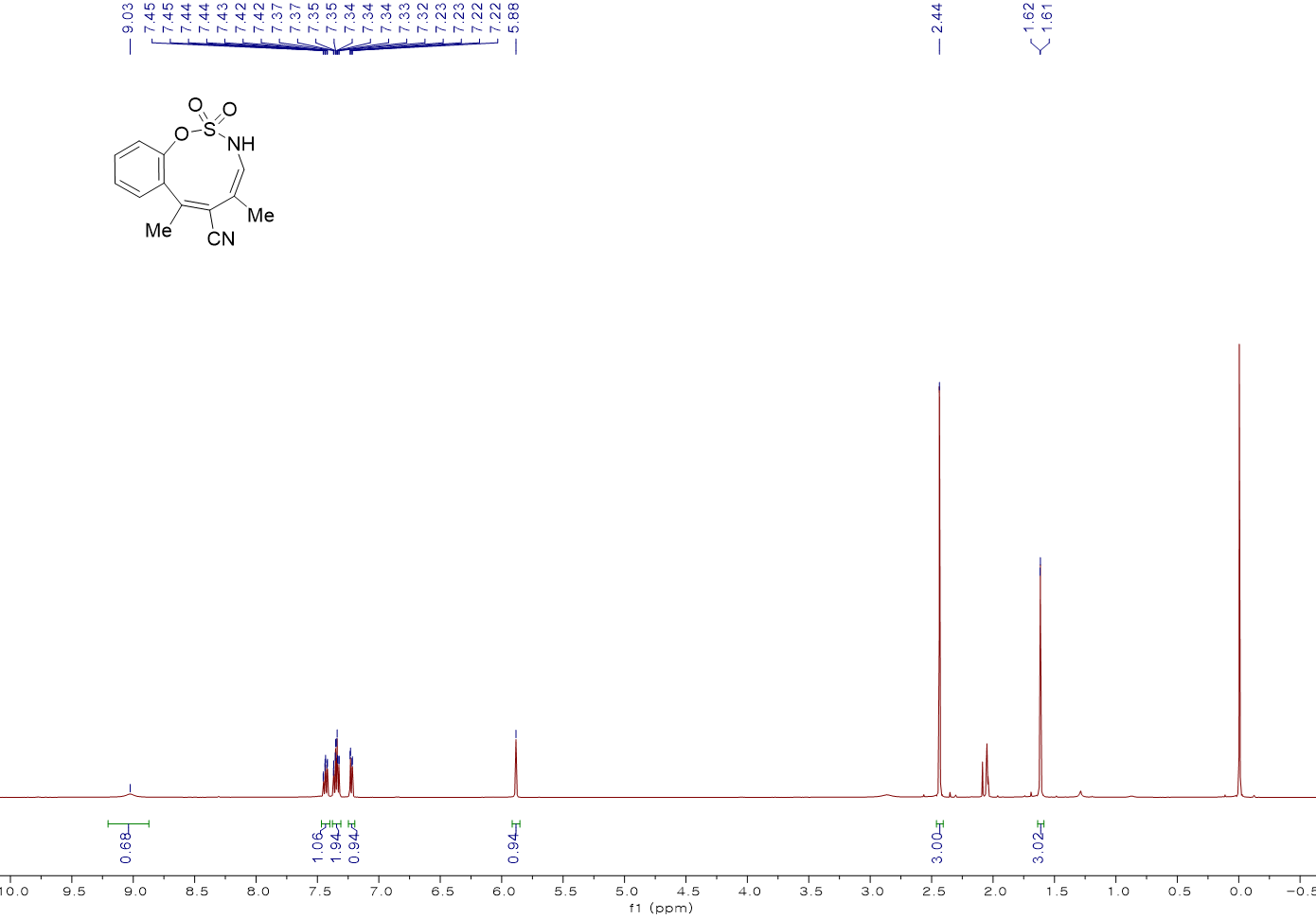


^1^H NMR spectrum of **5o**


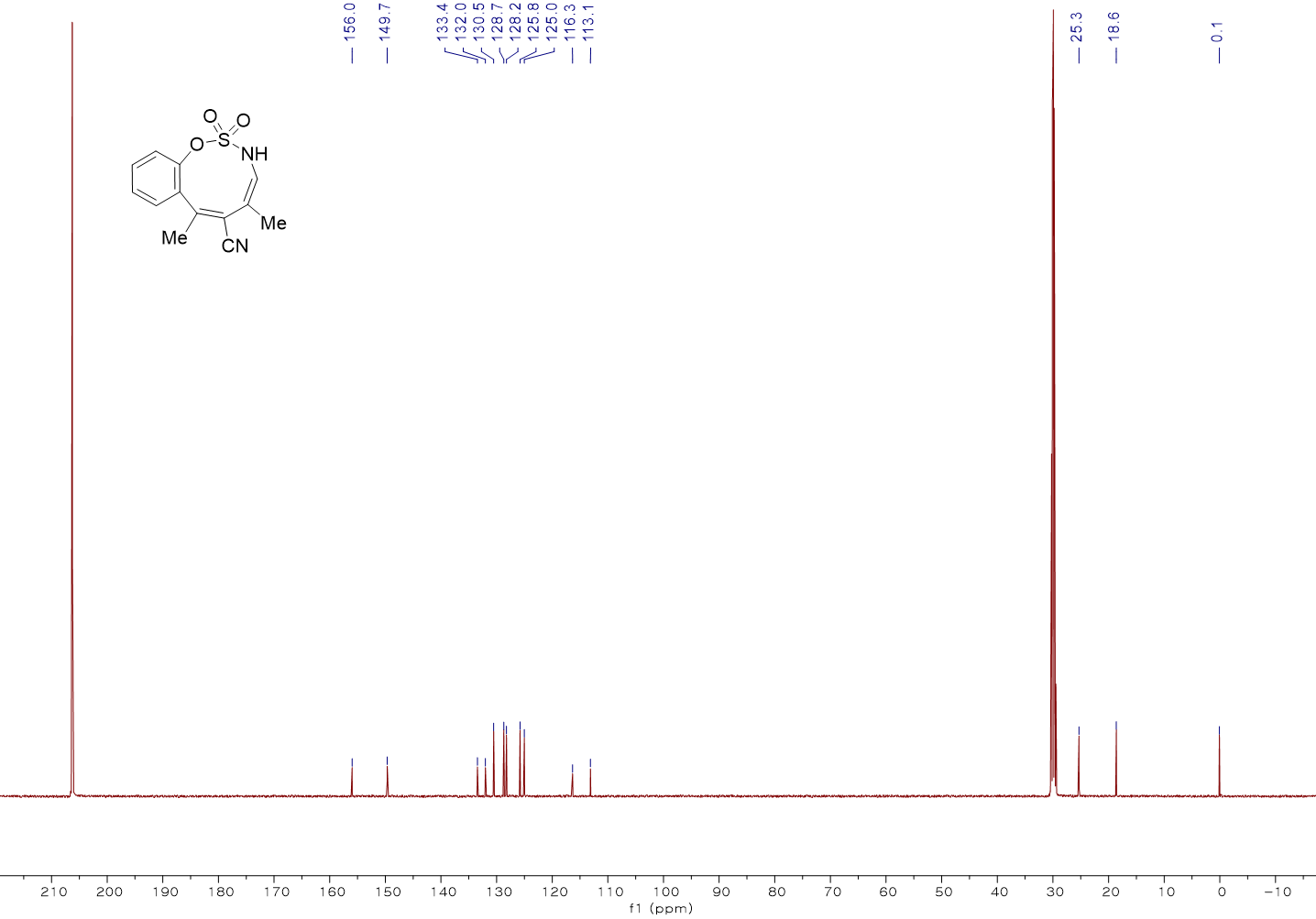


^13^C NMR spectrum of **5o**

**
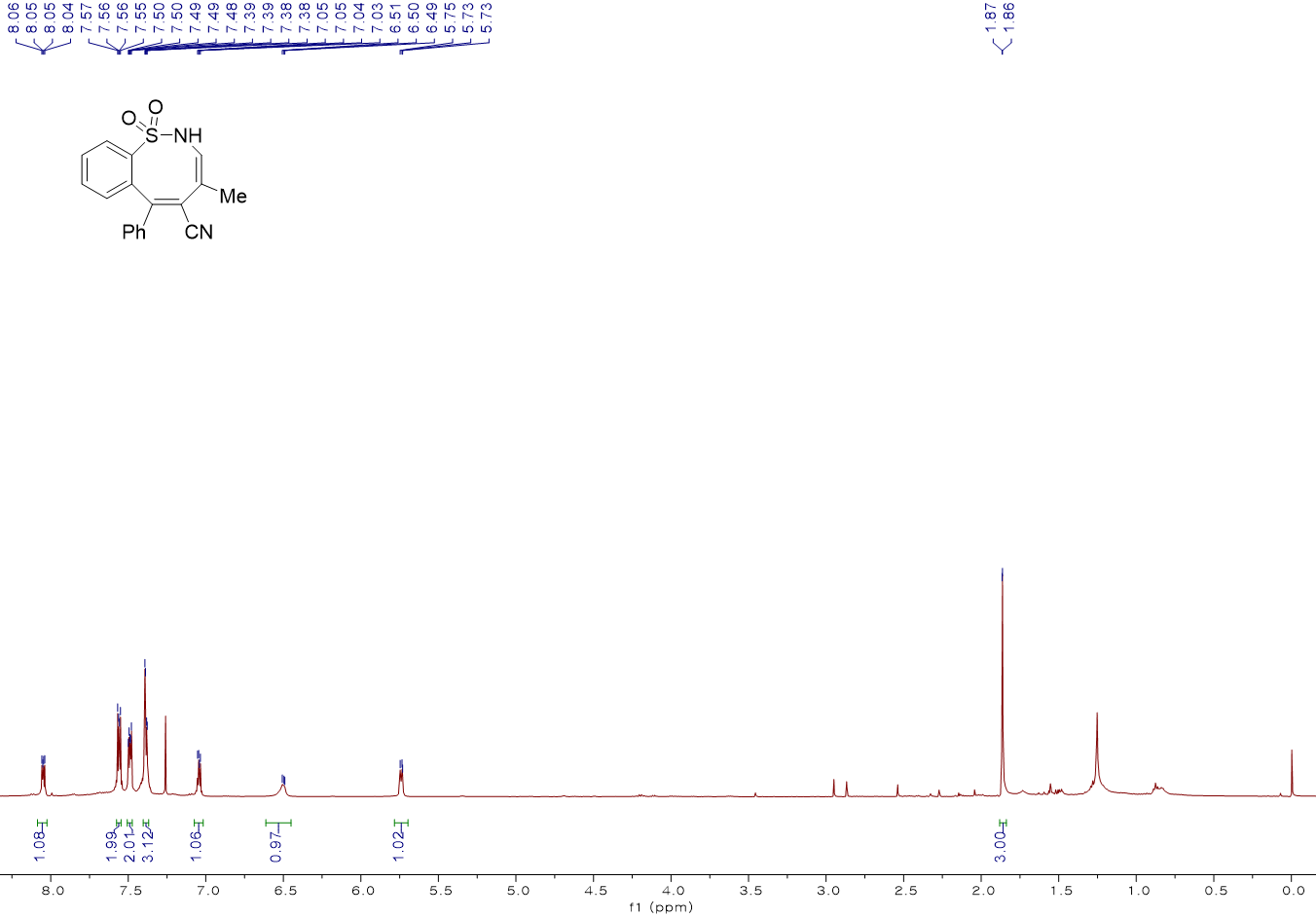
**

^1^H NMR spectrum of **5p**


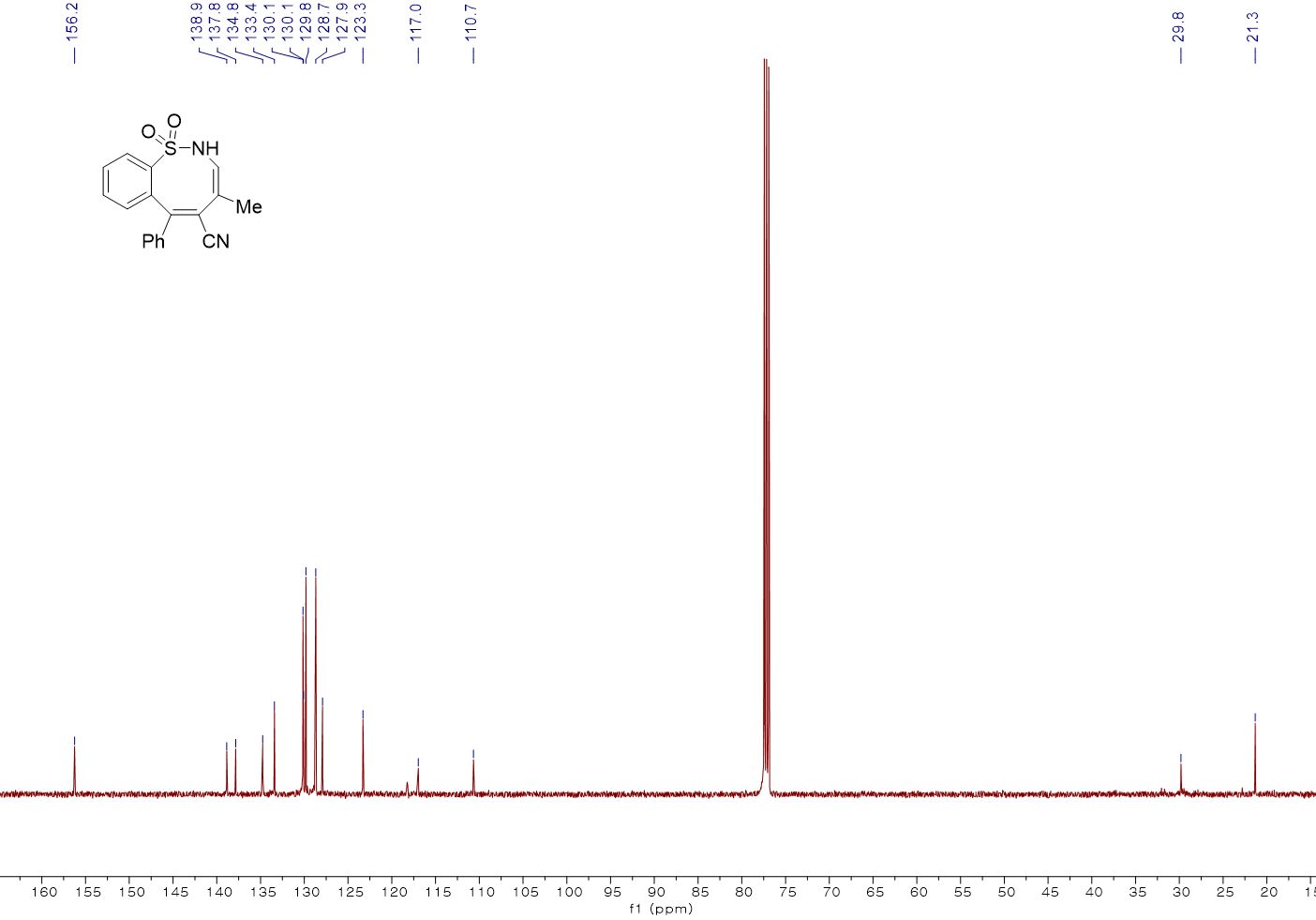


^13^C NMR spectrum of **5p**

**
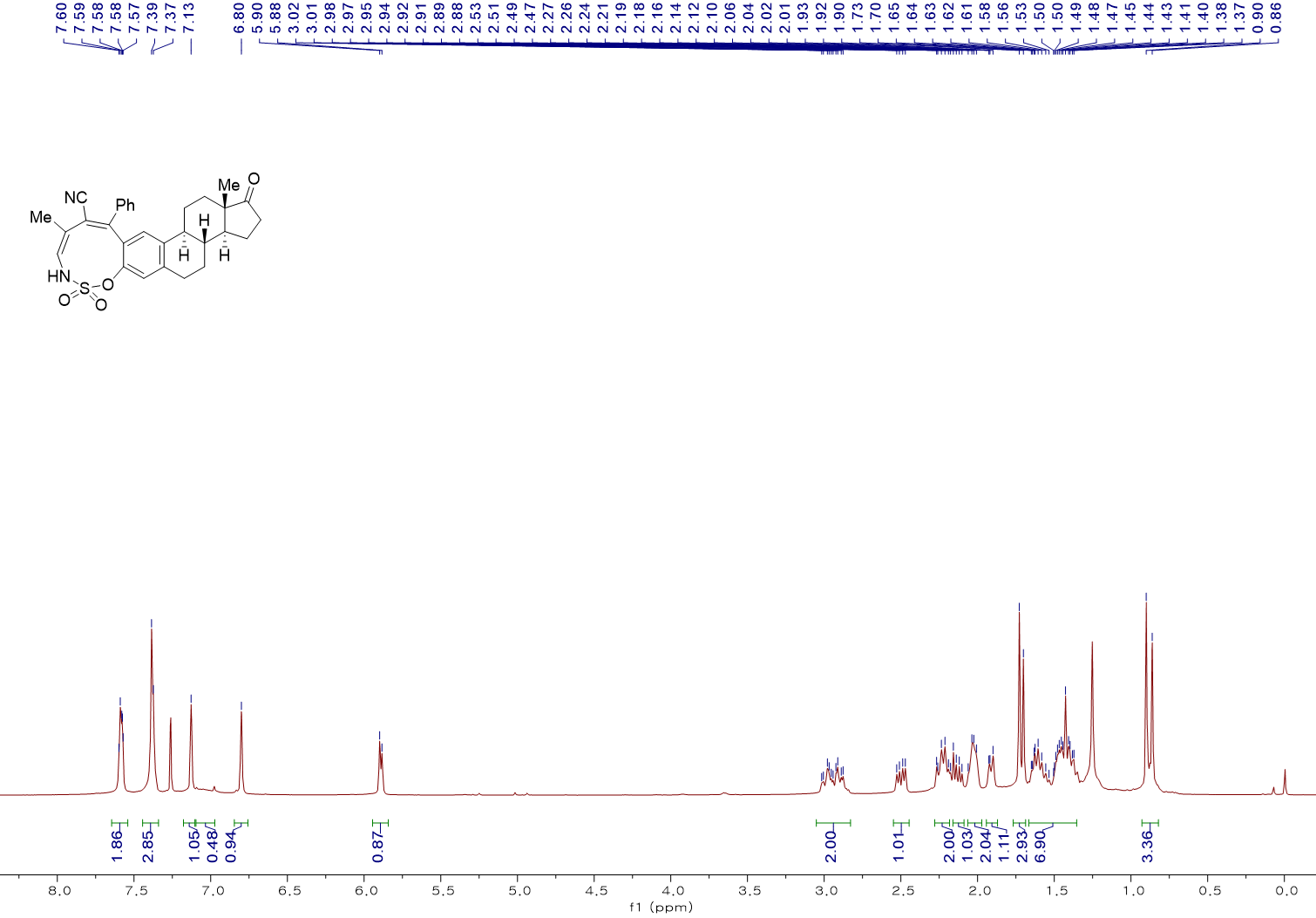
**

^1^H NMR spectrum of **5q**


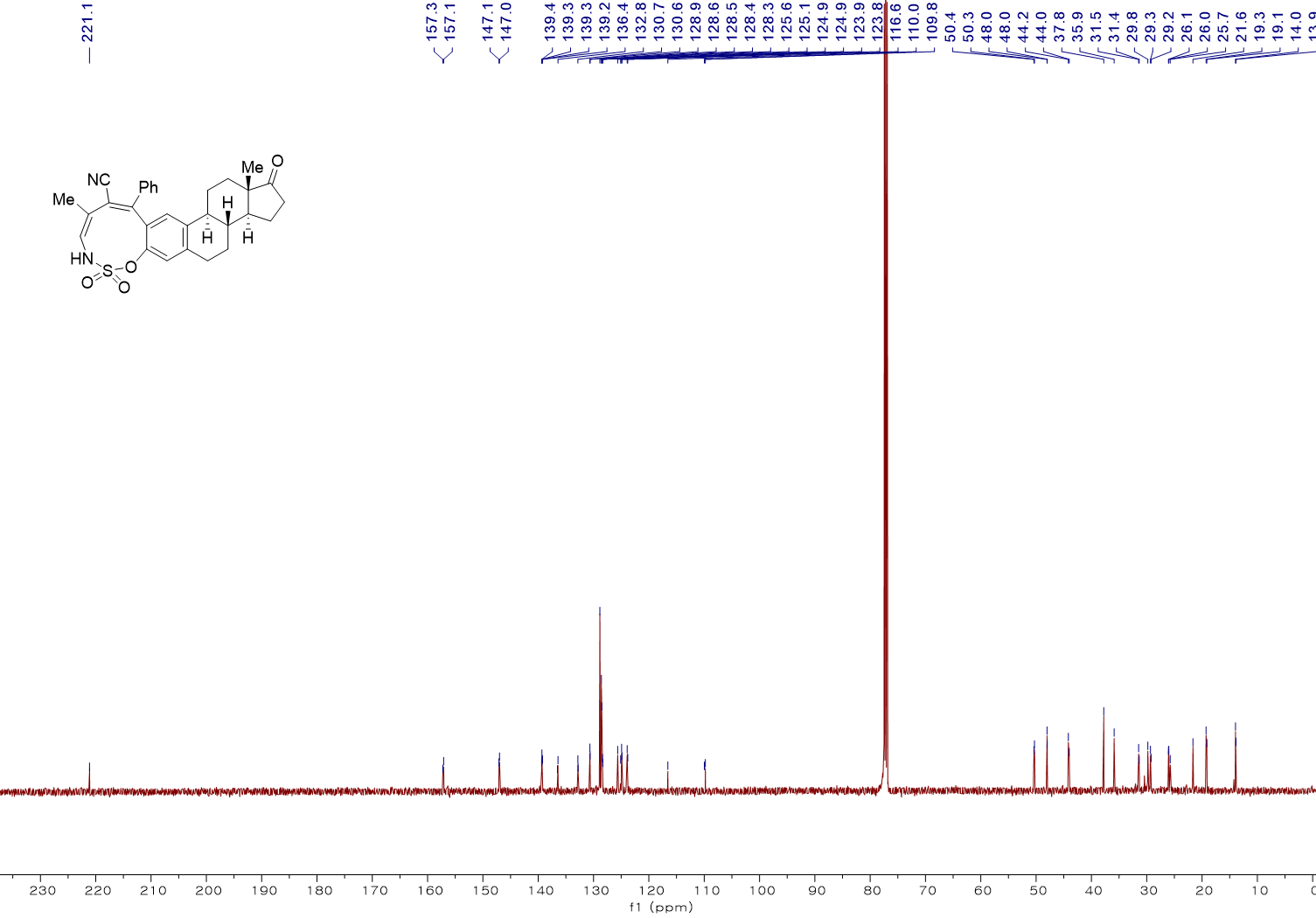


^13^C NMR spectrum of **5q**

^1^H NMR spectrum of **6a**

^13^C NMR spectrum of **6a**

^1^H NMR spectrum of **6b**

^13^C NMR spectrum of **6b**

^1^H NMR spectrum of **6c**

^13^C NMR spectrum of **6c**

^19^F NMR spectrum of **6c**

^1^H NMR spectrum of **6d**

^13^C NMR spectrum of **6d**

^1^H NMR spectrum of **6e**

^13^C NMR spectrum of **6e**

^1^H NMR spectrum of **6f**

^13^C NMR spectrum of **6f**

^1^H NMR spectrum of **6g**

^13^C NMR spectrum of **6g**

^1^H NMR spectrum of **6h**

^13^C NMR spectrum of **6h**

^19^F NMR spectrum of **6h**

^1^H NMR spectrum of **6i**

^13^C NMR spectrum of **6i**

^1^H NMR spectrum of **6j**

^13^C NMR spectrum of **6j**

^1^H NMR spectrum of **6k**

^13^C NMR spectrum of **6k**

^1^H NMR spectrum of **6l**

^13^C NMR spectrum of **6l**

^1^H NMR spectrum of **6m**

^13^C NMR spectrum of **6m**

^1^H NMR spectrum of **6n**

^13^C NMR spectrum of **6n**

^1^H NMR spectrum of **6o**

^13^C NMR spectrum of **6o**

^19^F NMR spectrum of **6o**

^1^H NMR spectrum of **6p**

^13^C NMR spectrum of **6p**

^1^H NMR spectrum of **6q**

^13^C NMR spectrum of **6q**

**13. HPLC Chromatograms of The Synthesized Compounds 4**

For racemic **4a**

For chiral **4a**

For racemic **4b**

For chiral **4b**

For racemic **4c**

For chiral **4c**

For racemic **4d**

For chiral **4d**

For racemic **4e**

For chiral **4e**

For racemic **4f**

For chiral **4f**

For racemic **4g**

For chiral **4g**

For racemic **4h**

For chiral **4h**

For racemic **4i**

For chiral **4i**

For racemic **4j**

For chiral **4j**

For racemic **4k**

For chiral **4k**

For racemic **4l**

For chiral **4l**

For racemic **4m**

For chiral **4m**

For racemic **4n**

For chiral **4n**

For racemic **4o**

For chiral **4o**

For racemic **4p**

For chiral **4p**

For racemic **4q**

For chiral **4q**

For racemic **4r**

For chiral **4r**

For racemic **4s**

For chiral **4s**

For racemic **4t**

For chiral **4t**

For racemic **4u**

For chiral **4u**

For racemic **4v**

For chiral **4v**

For racemic **4w**

For chiral **4w**

For racemic **4x**

For chiral **4x**

For racemic **4y**

For chiral **4y**
